# Extensive cellular multi-tasking within *Bacillus subtilis* biofilms

**DOI:** 10.1128/msystems.00891-22

**Published:** 2023-08-01

**Authors:** Sarah M. Yannarell, Eric S. Beaudoin, Hunter S. Talley, Alexi A. Schoenborn, Galya Orr, Christopher R. Anderton, William B. Chrisler, Elizabeth A. Shank

**Affiliations:** 1 Department of Microbiology and Immunology, University of North Carolina, Chapel Hill, North Carolina, USA; 2 Department of Biology, University of North Carolina, Chapel Hill, North Carolina, USA; 3 Department of Systems Biology, University of Massachusetts Chan Medical School, Worcester, Massachusetts, USA; 4 Environmental Molecular Sciences Laboratory, Pacific Northwest National Laboratory, Richland, Washington, USA; University of California, Berkeley, Berkeley, California, USA

**Keywords:** biofilms, cell types, confocal microscopy, flow cytometry, fluorescent reporters

## Abstract

*Bacillus subtilis* is a soil-dwelling bacterium that can form biofilms, or communities of cells surrounded by a self-produced extracellular matrix. In biofilms, genetically identical cells often exhibit heterogeneous transcriptional phenotypes, so that subpopulations of cells carry out essential yet costly cellular processes that allow the entire population to thrive. Surprisingly, the extent of phenotypic heterogeneity and the relationships between subpopulations of cells within biofilms of even in well-studied bacterial systems like *B. subtilis* remains largely unknown. To determine relationships between these subpopulations of cells, we created 182 strains containing pairwise combinations of fluorescent transcriptional reporters for the expression state of 14 different genes associated with potential cellular subpopulations. We determined the spatial organization of the expression of these genes within biofilms using confocal microscopy, which revealed that many reporters localized to distinct areas of the biofilm, some of which were co-localized. We used flow cytometry to quantify reporter co-expression, which revealed that many cells “multi-task,” simultaneously expressing two reporters. These data indicate that prior models describing *B. subtilis* cells as differentiating into specific cell types, each with a specific task or function, were oversimplified. Only a few subpopulations of cells, including surfactin and plipastatin producers, as well as sporulating and competent cells, appear to have distinct roles based on the set of genes examined here. These data will provide us with a framework with which to further study and make predictions about the roles of diverse cellular phenotypes in *B. subtilis* biofilms.

**IMPORTANCE:**

Many microbes differentiate, expressing diverse phenotypes to ensure their survival in various environments. However, studies on phenotypic differentiation have typically examined only a few phenotypes at one time, thus limiting our knowledge about the extent of differentiation and phenotypic overlap in the population. We investigated the spatial organization and gene expression relationships for genes important in *B. subtilis* biofilms. In doing so, we mapped spatial gene expression patterns and expanded the number of cell populations described in the *B. subtilis* literature. It is likely that other bacteria also display complex differentiation patterns within their biofilms. Studying the extent of cellular differentiation in other microbes may be important when designing therapies for disease-causing bacteria, where studying only a single phenotype may be masking underlying phenotypic differentiation relevant to infection outcomes.

## INTRODUCTION

Bacterial communities exist across diverse ecosystems. In these communities, genetically identical bacterial cells undergo differentiation that results in transcriptionally and functionally distinct cellular phenotypes (1). Such differentiation can result from nutrient availability (2), interspecies coculture interactions (3), stochastic effects (4), or specific microenvironments (5). It is thought that cells differentiate into phenotypically distinct subpopulations as a population survival strategy (2, 6 - 8). For many bacteria, the resulting phenotypic heterogeneity among genetically identical cells has implications for surface sensing (9, 10), virulence (11 - 13), and metabolism (14). For instance, some cell subpopulations may specialize in sugar incorporation (15) while others may release metabolic products (16). There is evidence of cross-feeding between glucose-fermenting and acetate-respiring subpopulations in *Escherichia coli* (17) and coordination between cells from the interior and periphery of *Bacillus subtilis* colony biofilms (18). Phenotypic differentiation is also implicated in antibiotic tolerance: *Pseudomonas aeruginosa* exhibits heterogeneity in its cellular metabolism due to oxygen gradients, which impacts how cells respond to antibiotics (19, 20). In *B. subtilis*, distinct subpopulations produce energetically costly compounds, like extracellular matrix components (21), extracellular proteases (22), or surfactin (23). This division of labor may have ecological benefits, since surfactin reduces surface tension to allow migration across solid surfaces (24) and extracellular matrix promotes plant root adherence (25).

Given these potential incentives for cellular differentiation, phenotypic heterogeneity is a hallmark of bacterial biofilms, which are complex communities of cells encased by a self-produced extracellular matrix (26). Many models of cellular differentiation exist, but *B. subtilis* is one of the best-characterized genetically tractable, biofilm-forming bacteria (27). Early cellular heterogeneity data initially led to *B. subtilis* being described as differentiating into six cellular phenotypes (cells that are motile, matrix producing, sporulating, cannibal, protease producing, and competent) (28, 29). This model was derived based on data from fluorescent transcriptional reporters that use the expression of a marker gene as a proxy for the cell’s transcriptional state (e.g., a flagellar protein is a marker for motility), with heterogeneous gene expression leading to the designation of these cell types. The explicit examination of phenotypic and transcriptional overlap between these putative cell types using strains containing two transcriptional reporters, however, has been conducted in only a handful of cases (30 - 35). These studies have added more nuance to our understanding of the overlap and exclusion of these phenotypes within individual cells, indicating that motile, matrix producing, and sporulating cells are spatiotemporally distinct within biofilms, as are matrix-producing cells from those that are competent or are producing the specialized metabolite surfactin (30 - 35). In contrast, matrix-producing and cannibal cells appear overlapping (23) while matrix-producing and protease-producing genes are co-expressed at certain times during growth (36). Mutagenesis studies have confirmed regulatory interactions between some of these genes, for instance, with the deletion of *hag* (motility) decreasing expression of the competence gene *comG* (37, 38). Beyond these few dual-reporter studies (30 - 35), however, the relationships between most other potential cellular subpopulations in *B. subtilis* are unknown.

In addition to these six canonical cellular phenotypes, metabolite-producing cell subpopulations also play important roles in *B. subtilis* biofilm populations (28, 39, 40). *B. subtilis* NCIB3610 produces at least 10 specialized metabolites, some of which act as cell–cell communication molecules and impact cellular differentiation (38). For instance, surfactin modulates biofilm formation by inducing the expression of biofilm-matrix genes under otherwise non-biofilm-forming conditions (31, 38, 41), while ComX has been shown to stimulate the expression of surfactin through a ComP-ComA signaling cascade (42, 43). Of the remaining *B. subtilis* metabolites, four others have had intraspecific signaling bioactivities ascribed to them (23, 38, 41, 44, 45). While the expression of a handful of these metabolites has been previously examined for heterogeneous expression patterns in *B. subtilis* (23, 31, 46 - 51), only three of these have been examined within *B. subtilis* colony biofilms (23, 31, 51, 52), and even fewer have been examined in terms of their spatial organization (51). Thus, our understanding of the gene expression relationships between these specialized metabolites and the other described *B. subtilis* phenotypes is fragmentary, with many studies examining cells grown under non-comparable growth conditions. Here, we aim to examine the expression patterns of all 10 metabolite biosynthetic genes and 6 phenotypic cell types (Table 1) under uniform biofilm-inducing conditions to obtain insights into the putative roles of these metabolites as cell–cell differentiation signals within *B. subtilis* colony biofilms on agar.

**TABLE 1:** Genes whose expression was monitored in this research, as well as the phenotype they are associated with

Gene	Phenotype
*aprF*	Protease
*bacA*	Bacilysin
*comG*	Competence
*comQ*	Pheromone
*dhbA*	Bacillibactin
*hag*	Motility
*pksC*	Bacillaene
*ppsA*	Plipastatin
*sboA*	Subtilosin
*sdpA*	Cannibal (SDP)
*skf*	Cannibal (SKF)
*skfAA*	Surfactin
*sspB*	Sporulation
*sunA*	Sublancin
*tapA*	Biofilm

Considering the many described cell types and metabolites known to exist within *B. subtilis* biofilms, the studies described above highlight that currently an incomplete understanding of *B. subtilis* cellular heterogeneity exists. We predict, based on the diverse gene expression relationships described so far, that substantial additional transcriptional multi-tasking occurs within *B. subtilis* biofilms. Here, we aim to determine the extent of both transcriptional multi-tasking and the spatial cellular coordination within *B. subtilis* biofilms using strains containing either one or two fluorescent transcriptional reporters for genes associated with specific cell types or specialized metabolites (Table 1). Using flow cytometry and fluorescence microscopy, we quantitatively measured the expression overlap between the expression of these genes within individual cells as well as visualizing their spatial distributions within *B. subtilis* biofilms. Overall, we determined that some genes examined were only expressed in a small subset of cells, while other cells multi-task, expressing multiple genes simultaneously. In addition, we observed that, overwhelmingly, most of the genes examined were expressed in distinct and repeatable spatial patterns across the biofilm, consistent with but expanding previously published work (51). We note that although gene expression can be substantially influenced by the age of the bacterial colony biofilm (30, 53), here we only examine a single time point from mature agar-colony biofilms. Overall, the data presented from this study provide a substantially improved, more comprehensive model of cellular heterogeneity within *B. subtilis* biofilms than currently exist.

## MATERIALS AND METHODS

### Bacterial strains and growth conditions

Table S1 lists the strains used in this study. For standard growth, *B. subtilis* strains were cultured on lysogeny broth (LB)-Lennox medium (10 g/L tryptone, 5 g/L yeast extract, 5 g/L NaCl, 1.5% agar) at 30°C for 16–18  h with antibiotics as necessary. TY (tryptone yeast) broth consisted of LB supplemented with 10 mM MgSO_4_ and 100 µM MnSO_4_ after autoclaving. Colony biofilms were grown on MSgg (Minimal Salts glutamate glycerol) medium [5  mM potassium phosphate (pH 7), 100 mM morpholine propanesulfonic acid (MOPS; pH 7), 2  mM MgCl_2_, 700 µM CaCl_2_, 50 µM MnCl_2_, 50 µM FeCl_3_, 1 µM ZnCl_2_, 2 µM thiamine, 0.5% glycerol, 0.5% glutamate] at with 1.5% agar at 30°C for 48 h. Antibiotics (final concentrations) were as follows unless noted otherwise: MLS (Macrolides, Lincosamides, Streptogramines: 1 µg/mL erythromycin, 25 µg/mL lincomycin) and chloramphenicol (5 µg/mL). *E. coli* strains were cultured in LB-Miller medium (10 g/L tryptone, 5 g/L yeast extract, 10 g/L NaCl, 1.5% agar). Final concentration of carbenicillin was 50 µg/mL.

### Colony morphology phase-contrast and fluorescence imaging and analysis

Macrocolony biofilm images were gathered using a Zeiss SteREO Discovery.V8 dissecting stereomicroscope (Zeiss, Oberkochen, Germany) with a 1 × 0.63 lens objective in three channels: brightfield, YPet, and mTurq. Fluorescence was generated using a Lumen Dynamics XCite 120 fluorescence lamp with either an YFP cube (KSC 295-823D; excitation: HQ500/20; dichroic: Q515LP; emission: HQ535/30) or a cyan cube (KSC 296-824D; excitation: D436/20; dichroic: 455DCLP; emission: D480/40). Images were exported as a .TIFF for image analysis at 1388 × 1040 pixels for Fig. S2. These images were indexed and grouped based on the strain and reporters the strain contained. Images were then imported into Matlab 2020b. For each strain, the location of the colony was determined by masking the image using the Laplassian of Gausian of a grayscale brightfield image. This method of detection was used because the agar was a single, relatively uniform value, while the colony was a substantially different, relatively uniform value. The center of the colony was determined using the Centroid function in Matlab. The edge of the colony was determined using bwboundries function in Matlab on the masked colony image. The average Euclidian distance, in number of pixels, between the center and edge of the colony was then determined. Each pixel’s “Euclidian distance from the center of the colony” was divided by the “average Euclidian distance from the center of the colony to the edge,” transforming each pixel’s distance into a percentage (with the edge of the colony being 100%) to allow for easier comparison between colony images. Each distance was rounded to 2 decimal places, then the mean signal in mTurq and YPet was taken for each unique distance at and graphed. Graphs were limited to 120% on the X-value to capture background fluorescence just beyond the edge of the colony. Y-values were normalized to an appropriate and consistent value for each reporter. The code can be found at DOI: 10.5281/zenodo.4624987.

### SPP1 phage transduction

Phage transduction was carried out as previously described (54). Briefly, we grew the *B. subtilis* donor strain at 37°C in TY broth until the culture reached an OD_600_ of 1.0. At that point, we infected cells with SPP1 phage stock and incubated for 15 min at 37°C. We then added 0.5% TY soft top agar to the cells and phage, overlaid the mixture on TY 1.5% agar plates, and incubated plates at 37°C for 8–16  h. *B. subtilis* donor phage plaques were collected and pelleted using a clinical centrifuge. We infected *B. subtilis* recipient cells with 300 μL of supernatant, and then plated the cell lysate on LB-Lennox with 10  mM citrate and antibiotic to which the donor strain was resistant. Plates were incubated at 37°C for 12–24  h. Four colonies were picked from each phage transduction and struck on LB-Lennox plates with antibiotic. After growth, strains were restruck 2 more times on LB-Lennox plates with antibiotic. Cells were spotted on MSgg and incubated at 30°C to ensure growth, which indicates that the cells have a 3610 rather than a 168 background (which is an amino acid auxotroph). Specifics of reporter construction are described below.

### Construction of *B. subtilis* reporter strains

The newly constructed transcriptional reporter plasmids (pES099—pES112) containing *YPet* were derived from pES045 (*amyE::*P*_spacC_-YPet*) (55, 56). To construct these plasmids, the *spacC* promoter was removed by digestion with EcoRI and HindIII and replaced with promoter sequences. Promoter sequences were amplified from *B. subtilis* wild-type genomic DNA (see primers in Table S1) and inserted into the base plasmid by isothermal assembly (also used for all subsequent constructions described in this section) (57) and transformed into *E. coli*.

A plasmid-containing mTurquoise2 (*mTurq*) was generated using primer ES395 and primer ES315 (see Table S1) to amplify *mTurq* from GL-FP-31. The fragment was cloned into plasmid pDR183 [*lacA*::(*mls*)] (58) digested with SalI and EcoRI. To create *mTurq* reporters, we amplified promoter sequences from *B. subtilis* wild-type genomic DNA (see primers in Table S1), digested with NheI and SalI, and inserted into the pDR183-*mTurq* base plasmid (pES069) using isothermal assembly. The assembled plasmids were transformed into *E. coli*.

Upon final construction, the plasmids were isolated from *E. coli*, linearized, and transformed into *B. subtilis* 168 cells grown to stationary phase. Cells containing *YPet* reporters were plated on Lennox-chloramphenicol to select for transformants. Cells containing *mTurq* reporters were plated on Lennox-MLS to select for transformants. Phage transduction was carried out as described previously (54) and above. *B. subtilis mTurq* reporters were used as the donor strains and grown to 37°C in TY broth until the culture reached an OD_600_ of 1.0. Cells were infected with SPP1 phage stock and plated on 0.5% TY soft top agar, overlaid on TY 1.5% agar plates, and incubated at 37°C for 8–16 h. *B. subtilis* donor phage plaques were collected and pelleted using a clinical centrifuge. Three hundred microliters of supernatant was used to infect *B. subtilis* 3610 wild-type and *B. subtilis YPet* reporter strains (recipient cells) to construct single- and dual-fluorescent reporters, respectively. The cell lysate was then plated on LB-Lennox with 10 mM citrate and MLS to which the donor *mTurq* reporter strains were resistant. Plates were incubated at 37°C for 12–24 h. Three colonies were picked from each phage transduction and struck on LB-Lennox plates with MLS and citrate to select for *B. subtilis* cells that contained *mTurq* reporters. For strains containing dual-fluorescent reporters, strains were then restruck on Lennox-chloramphenicol to select for strains containing both *mTurq* and *YPet* reporters. Cells were spotted on MSgg and incubated at 30°C to ensure growth, which indicates that the cells have a 3610 rather than a 168 background (which is an amino acid auxotroph). Colony morphology of reporter strains was compared with wild type, as morphology should be identical.

### Flow cytometry

*B. subtilis* strains were prepared and grown on MSgg as described above. After 48 h of growth, biofilms were collected and resuspended in 1 mL 1X phosphate buffered saline (PBS) using a 23G needle and syringe to shear the biofilm. Cells were pelleted by centrifugation at 16,000 × g, the supernatant was removed, and the cells were fixed in 200 µL of 4% (wt/vol) paraformaldehyde for 7 min. After the incubation, the cells were pelleted, washed in 1× PBS to remove residual paraformaldehyde, and resuspended in GTE buffer (1% glucose [wt/vol] and 5 mM EDTA in 1× phosphate buffer, pH 7.4). Samples were stored at 4°C until flow cytometry analysis. Prior to analysis, cells were sonicated for 12 pulses (1 s pulse with subsequent 1 s pause) and filtered through a 38 µm nylon mesh. We have previously confirmed that this sonication treatment does not lyse cells (by measuring the levels of the cytoplasmic protein σA in the cell pellet and supernatant via Western blotting) but yields single cells for flow cytometry (based on microscopic analysis of samples on agar pads) (45). *YPet* and *mTurq* fluorescence in dual-reporter strains was measured using the 488 and 457 lasers, respectively, of the Influx cell sorter (BD Biosciences). Single-color controls were used to confirm that the spectral overlap of these fluorophores was minimal and thus that compensation was not required. Gating with the non-fluorescent control strain (WT 3610) enabled us to set detection limits for cells expressing either of the two fluorophores.

### Thin-sectioning

The thin-sectioning protocol was adapted from Vlamakis et al. (30) and Marlow et al. (36). *B. subtilis* strains were cultured on MSgg as described above and vapor fixed with 8% paraformaldehyde (adapted from reference 59)). Biofilm-agar blocks were quartered, transferred to a 15 × 15 × 5 mm mold (Fisher; Cat: 22-363-553), and snap-frozen. The colony was then overlaid with 4% (wt/vol) agarose (Lonza; Cat: 50181) and frozen at −80°C for 15 min. The blocks were transferred to −20°C for 30 min to equilibrate. Colony blocks were mounted to the chuck with double-distilled H_2_O and sliced to 20-µm-thick cross-sections using a cryotome (Thermo Cryostar NX70). Sections were attached to VWR Superfrost Plus slides (Cat: 48311-703) and stored at −20°C.

### Confocal microscopy and image analysis

Sections for microscopy were overlaid with mounting medium ProLong Gold Antifade Mountant (ThermoFisher; Cat: P10144) unless otherwise noted and a 25 × 25 mm coverslip (Fisher; 12-548-C). Sections were imaged using a Zeiss 710 laser scanning confocal microscope equipped with a 20X EC Plan NEOFLUAR and 100X Plan APOCHROMAT oil objective.

### BiofilmQ analysis

We analyzed the biofilm gene expression using BiofilmQ (60). We selected voxels of 10 cubic pixels, or about 0.80 µm per side. Pearson correlation coefficients were derived for each voxel by looking at the intensity of each of the two reporters expressed by each strain. Each Pearson value was overlaid onto the centroid location of the voxel in the image.

## RESULTS

### Heterogeneous *B. subtilis* gene expression at the colony level

To monitor *B. subtilis* gene expression in colony biofilms, we constructed strains containing fluorescent transcriptional reporters for key *B. subtilis* genes. To do this, we introduced the fluorescent protein YPet (a brighter variant of yellow fluorescent protein) under the control of promoters for 15 genes of interest (Table 1) and incorporated them into the neutral *amyE* locus (61). These genes are involved in specialized metabolite production, extracellular matrix production, motility, sporulation, competence, protease production, and cannibal antibiotic production. Exploring this range of genes was intended to provide a more global view of cellular phenotypic variation in *B. subtilis* than previously examined as well as focus on potential intraspecific signals that could be stimulating cellular differentiation. We initially looked at the fluorescent transcriptional reporters for all 10 of the specialized metabolites but excluded sublancin from further experiments because initial results showed that the colony morphology of the P*_sun_-YPet* strain differed from that of wild-type *B. subtilis* (Fig. S1). In this manuscript, we, therefore, focused on the expression of 14 *B. subtilis* genes (Table 1).

**Fig 1:**
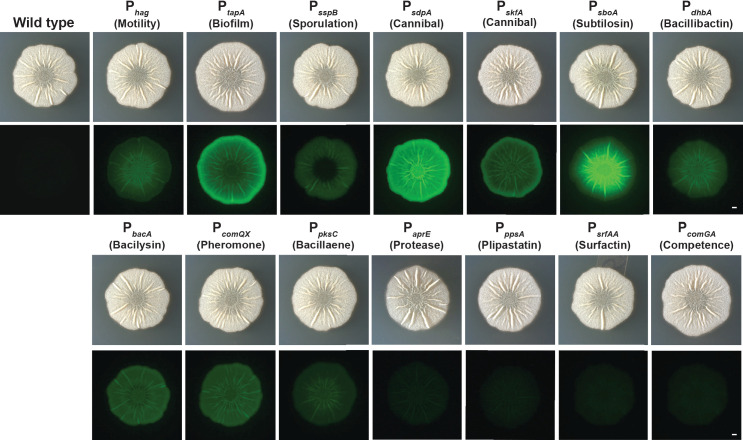
*Bacillus subtilis* exhibits heterogeneous gene expression patterns at the colony level. Wild-type *B. subtilis* and *B. subtilis* strains containing fluorescent reporters were grown at 30°C on the biofilm-inducing medium MSgg for 48 h. Brightness was linearly adjusted in the same way for each image using Fiji. Bar, 1 mm.

To obtain information about the relationships between the expression of these genes, we first asked how the expression of these fluorescent transcriptional reporters was localized and how intensely they expressed *YPet* within *B. subtilis* biofilms. We grew biofilm colonies from an OD_600_-normalized inoculum on MSgg, a *B. subtilis* biofilm-inducing media (62) and imaged colonies at 48 h (Fig. 1) using brightfield and fluorescence illumination. We calculated the average fluorescence intensity across the colony, averaged from the center outward (Fig. S2). At the colony level, we observed a range of reporter expression patterns. Two metabolite reporters (*sdpA* and *sboA*) were expressed at high levels; eight reporters (for genes encoding metabolite or structural products: *bacA, tapA, skfA, dhbA, comQX, hag, pksC,* and *sspB*) were expressed at mid-range levels; and the reporters for four genes (*ppsA, srfAA, comGA,* and *aprE*) appeared expressed at low or at near-background levels (Fig. 1 and Fig. S2). With regards to their localization, the *sdpA, bacA, comQX, ppsA, srfAA*, and *aprE* reporters seemed to be consistently expressed throughout the colony (Fig. 1 and Fig. S2). In contrast, *hag, sboA, dhbA,* and *pksC* were expressed primarily in the interior of the colony while *tapA*, *skfA*, and *sspB* were expressed mostly in the periphery (Fig. 1 and Fig. S2). Thus, even by simply comparing the fluorescence localization of different reporters at the colony level, we already observed some genes with similar spatial expression patterns and others with distinct expression patterns.

### Identifying colony-level gene expression relationships using dual-reporter strains

**Fig 2:**
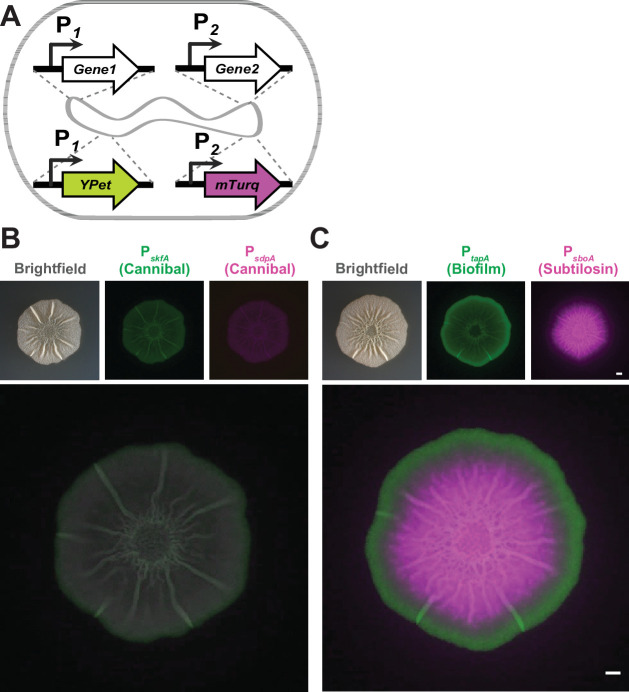
*Bacillus subtilis* dual-reporter strains allow for direct localization comparison of expression. (A) Dual-reporter construction schematic. (B) Individual and merged channels of a *B. subtilis* biofilm containing *sdpA* (cannibal) and *skfA* (cannibal) reporters and (C) a *B. subtilis* biofilm containing *tapA* (biofilm) and *sboA* (subtilosin) reporters grown on MSgg for 48 h. Colony images were taken from the top using a dissecting stereomicroscope. Bars, 1 mm.

We hypothesized that the regions of the *B. subtilis* colony where more than one fluorescent transcriptional reporter appeared expressed at the colony level could either be composed of highly heterogeneous populations of cells or else individual cells in those regions could be expressing multiple genes simultaneously, that is, multi-tasking. To better visualize and quantify gene expression co-localization within *B. subtilis* colonies, we generated strains that contained pairwise combinations of these 14 reporters at two neutral sites on the *B. subtilis* chromosome (one reporter at the *amyE* locus (61) and the other at the *lacA* locus (63), expressing either *YPet* or the brighter cyan fluorescent protein variant, *mTurq*) using phage transduction (Fig. 2A). In combining the reporter constructs for 14 genes of interest, we created 182 strains; this included all 91 possible pairwise combinations of the 14 genes in each of the two-color orientations (e.g., both P*_hag_-YPet::amyE*, P*_tapA_-mTurq::lacA* and P*_tapA_-YPet::amyE*, P*_hag_-mTurq::lacA* to control for differences in fluorescent protein expression levels). Only six such dual-reporter strain pairs (*hag-tapA; hag-sspB; tapA-sspB; srfAA-tapA; skfA-tapA*; and *tapA-comGA*) have been explicitly examined in the literature previously (23, 30, 31, 33, 34). All strains containing dual reporters grew similarly to wild-type *B. subtilis* (Fig. S3). In analyzing the fluorescence levels of the colony biofilms of these strains, we built a comprehensive picture of the spatial expression relationships between each of these gene pairs. We did so by growing the dual-reporter stains on MSgg and visualizing fluorescence within colonies at 48 h to directly compare their expression patterns. Some pairs, like *sdpA* (cannibal) and *skfA* (cannibal), exhibited high levels of co-localization (indicated by white color in false-colored overlay image, Fig. 2B). Conversely, a *B. subtilis* strain containing *tapA* (matrix-producing) and *sboA* (subtilosin) reporters exhibited little co-localization between the expression of these two genes (Fig. 2C). This colony-level fluorescence microscopy allowed us to identify areas of the colony that appeared to contain co-localized reporter gene expression.

### Analyzing gene expression in stratified, thin-sectioned colonies by confocal microscopy

**Fig 3:**
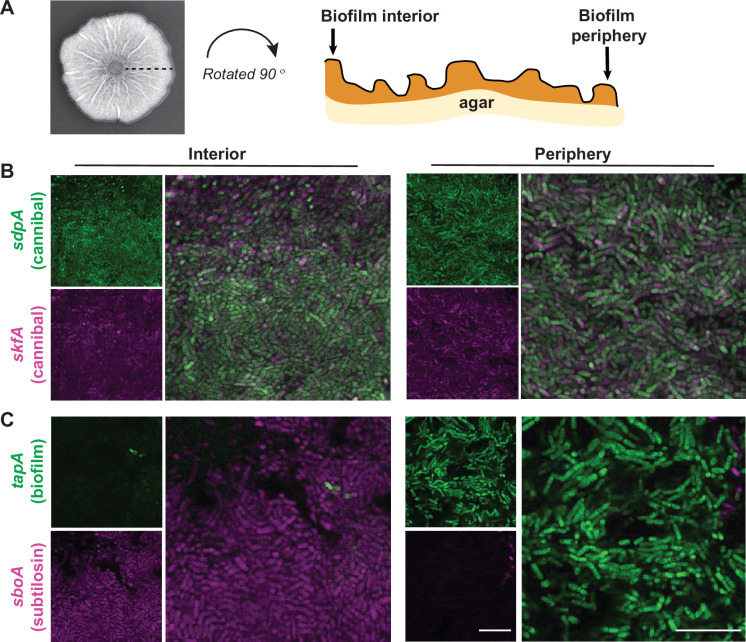
Specific phenotypic reporters display regions of co-localization and regions of distinct expression. (A) Schematic of *Bacillus subtilis* biofilm thin-section shown using a representative colony image. (B) Micrographs showing individual and merged channels of a *B. subtilis* biofilm containing *sdpA* (cannibal) and *skfA* (cannibal) reporters and (C) *B. subtilis* biofilm containing *tapA* (biofilm) and *sboA* (subtilosin) grown on MSgg for 48 h, thin-sectioned, and imaged using Airyscan confocal microscopy at 100× to image interior and periphery regions. For each reporter pair, the intensities were optimized to show differences between reporters (i.e., intensities of B and C are not comparable). Bars, 10 µm.

We delved further into the potential co-expression of genes across the biofilm using a confocal-laser-scanning microscope (CLSM) with an Airyscan detector. To gather spatial information not only from the surface of the colony but also from individual cells within the biofilm, we quartered and thin-sectioned the colonies to 20-µm thick and flipped the sections on their side for imaging (schematic in Fig. 3A). This approach enables a finer spatial visualization of fluorescence expression patterns within the depth of biofilms and provides information about the distributions of cells expressing different genes across the structure. For example, both *sdpA* (cannibal) and *skfA* (cannibal) reporters are present throughout the interior and peripheral regions of the colony and the reporters seem fairly well mixed in these regions, with some cells co-expressing both fluorescent reporters (white cells in Fig. 3B). In contrast, *sboA* (subtilosin) and *tapA* (matrix producing) are predominantly localized to the interior and the periphery, respectively, and these reporters seem to be mutually exclusive in individual cells in this area of the colony (Fig. 3C).

### Flow cytometry to quantify gene expression within individual cells

While the results from CLSM provided information about the spatial organization of gene expression across *B. subtilis* biofilm colonies, these data were not quantitative, and in some cases, it remained ambiguous whether (and the extent to which) genes were being co-expressed in the same cell. Therefore, we used flow cytometry to quantify gene expression and co-expression within *B. subtilis* biofilm cells using our dual-reporter strains. We harvested colonies of all 182 dual-reporter strains grown on MSgg and fixed the cells using paraformaldehyde to prevent changes in expression levels during processing. Samples were sonicated, filtered, and analyzed on a flow cytometer, where data from a minimum of 24,000 cells per strain were collected. We used *B. subtilis* wild-type, non-fluorescent control samples to set the flow cytometry fluorescence detection gates, which enabled us to differentiate cell populations from each strain that were: (i) not expressing either fluorophore, (ii) only expressing *mTurq*, (iii) only expressing *YPet*, or (iv) expressing both fluorophores (Fig. S4).

**Fig 4:**
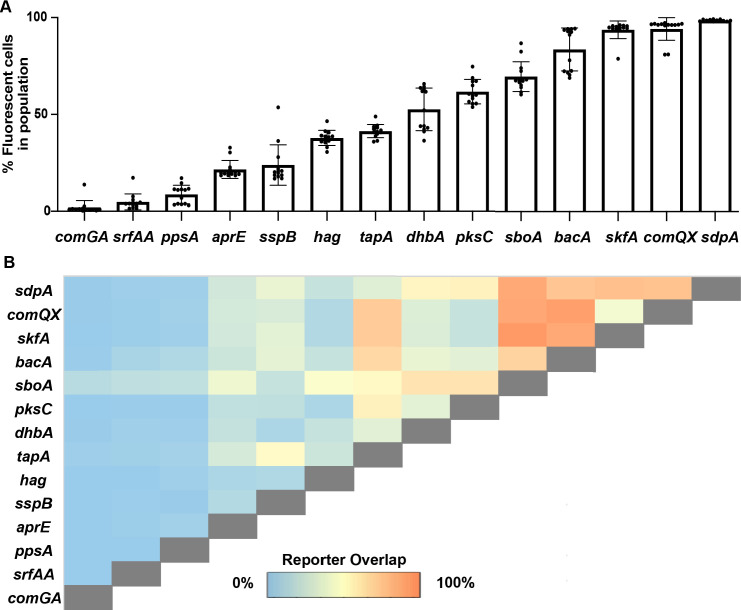
Many *Bacillus subtilis* genes exhibit co-expression at the individual cell level. (**A**) Percent of fluorescent cells in a *B. subtilis* biofilm population after 48-h growth on MSgg was determined by flow cytometry. (**B**) The percentage of cells co-expressing two reporters after 48-h growth on MSgg.

To understand how many cells in the overall biofilm were expressing each individual gene, we first quantified the total percentage of cells expressing *YPet* from each strain (Fig. 4A). (We used only the *YPet* signal from each dual-labeled strain for these calculations since the sensitivity of this fluorescent protein was superior to *mTurq* due to background cell fluorescence in the *mTurq* channel). With this analysis, we determined that most cells within the *B. subtilis* population (on average over 90%) express *sdpA*, *comQX,* and *skfA,* while on average less than 12% of the population expresses *ppsA*, *srfAA*, or *comGA* (Fig. 4A). The remaining 11 genes were expressed in between 25% and 75% of the cell population. Note that, although here we are using fluorescence as a proxy for gene expression, fluorescence levels do not necessarily correlate with higher levels of protein or subsequent metabolite production: some biosynthetic protein machines may be long lived within cells or their activity may be post-translationally regulated. Nevertheless, these intensity profiles were reproducible and, therefore, we expect may reflect real biology.

To understand the expression relationships between all of these genes, we then quantified the proportion of cells that expressed both *mTurq* and *YPet* in each dual-labeled strain. We observed a range of gene-expression relationships, from completely distinct to fully overlapping (Fig. 4B; flow cytometry plots can be found in the supplemental material, Fig. S4; the values reflected in Fig. 4 represent the maximum overlap values observed between in the matched Ypet-mTurq gene pairs). Our data set includes reporter pairs that corroborate results from previous studies [e.g., motility (*hag*) and sporulation (*sspB*) were not co-expressed (30) and biofilm matrix (*tapA*) and *skfA* (cannibal) reporters were overlapping (23)]. However, in our data set, >20% of the population co-express *tapA* and *sspB* reporters, which were originally described as distinct cell types (30). This overlap presumably represents cells transitioning between these two differentiation states, and our ability to detect this population likely reflects the brighter fluorophores used in our study compared to those originally available (30). Unexpectedly, *comGA*, *ppsA*, and *srfAA* had minimal expression overlap with any other genes (Fig. 4B) (with the exception of *sboA*, which was expressed in nearly all cells), indicating that cells expressing these genes may indeed represent more distinct cell types that are specializing in a particular task. This may be a result of the energetic costs required to generate these protein machines or due to shunting substrates they need away from other metabolic pathways. Beyond this, although most genes demonstrated some expression overlap with other genes (Fig. 4B and Fig. S4), a few pairs of genes appeared to have anticorrelated expression: *sspB* with *dhbA, aprE,* and *hag*, as well as *hag* with *aprE, comQX*, *skfA*, and *pksC* (Fig. 4B). Overall, the flow cytometry results revealed extensive multi-tasking occurring in cells in *B. subtilis* biofilms.

### Correlating flow cytometry and confocal fluorescence microscopy

**Fig 5:**
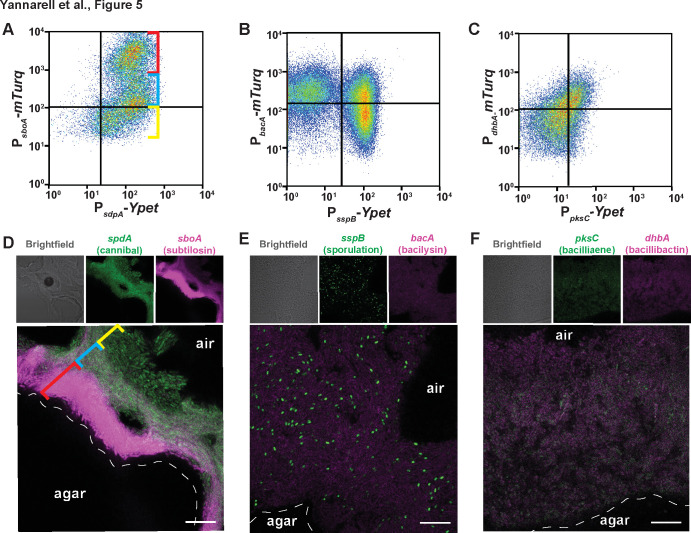
Corresponding reporter spatial arrangement with relationships displayed in flow cytometry data. Flow cytometry of the fluorescent intensities of *Bacillus subtilis* cells containing (A) *sdpA* and *sboA*, (B) *sspB* and *bacA*, and (C) *dhbA* and pksC reporters harvested from the 48-h timepoint. The gates were constructed from the non-fluorescent control sample from that experiment. A total of 24,000 cells were quantified for each sample. The *mTurq* reporter was detected using a 457-nm laser, and the *YPet* reporter was detected using a 488-nm laser. Confocal microscopy of *B. subtilis* biofilm thin-sections containing (D) *sdpA* and *sboA*, (E) *sspB* and *bacA*, and (F) *dhbA* and pksC reporters at 100×. For (D) and (E), propyl gallate mounting medium was used. For each reporter pair, the intensities were optimized to show differences between reporters and intensities are therefore not comparable across panels D, E, and F. Bars, 10 μm.

Because by nature flow cytometry data lack spatial information, we next wanted to investigate how cells expressing particular genes or gene pairs were spatially distributed within the colony. To do so, we used confocal fluorescence microscopy to spatially visualize the expression of gene pairs and observed diverse co-expression patterns (Fig. 5 and Fig. S5). The flow cytometry data from the *sdpA-sboA* reporter pair indicate that almost all *sboA*-expressing cells also express *sdpA* but that there is also a subset of *sdpA*-only expressing cells (Fig. 5A, yellow bracket). The cells expressing both reporters have a bimodal expression, where some express *sboA* at lower levels, and others at very high levels (Fig. 5A, blue and red bracket, respectively). Interestingly, the spatial organization of these subpopulations of cells is not random: *sboA* expression is largely missing from the top interior area of the biofilm, while the brighter *sboA* population exists at the colony–agar interface and the *sdpA-*only subpopulation of cells are found at the colony–air interface (Fig. 5D). This expression pattern of *sboA* is consistent with recent mass spectrometry imaging data showing that subtilosin A is primarily distributed at the bottom of the colony and in the agar underneath it (51).

The *sspB* gene, which is expressed late during sporulation (30), also exhibited a bimodal expression pattern, being either on or off in our micrographs (Fig. 5B). This distribution of expression is consistent with previous reports of the expression of *sspB*; at 48 h of biofilm growth on MSgg a subset of the population has begun to sporulate (30). The cells expressing *sspB* are also small and punctate in our fluorescence micrographs, consistent with cells undergoing sporulation (Fig. 5E) (64, 65); notably, the true population of spores is likely underrepresented based on the expression of P*_sspB_-YPet*, which will not be active in mature spores (30). In this field of view, we see little overlap of *sspB* with *bacA* (bacilysin)-expressing cells, which corresponds to the lower right quadrant of the flow cytometry data (Fig. 5E); cells co-expressing *bacA* and *sspB* must, therefore, reside elsewhere in the colony or else be diffusely spread across the entire colony, since they are not visible here (Fig. 5E). Conducting flow cytometry from more finely resolved spatial regions of the colony would enable future work to better resolve the position of this subset of cells within the biofilm.

Finally, for a handful of reporters, we observed partially overlapping gene expression patterns in our flow cytometry data (e.g., Fig. 4B). The flow cytometry plot for reporter pair *dhbA* (bacillibactin) and *pksC* (bacillaene) displays this partial overlap, with each reporter expressed alone in a subset of cells as well as in some cells simultaneously (Fig. 5C). Regions of co-localization in the center interior of the biofilm correlate to the observed co-expression in flow cytometry data (Fig. 5F). We have qualitatively summarized the spatial distributions of the different reporters obtained from fluorescence microscopy to better synthesize the range of expression patterns and facilitate an understanding of potential multi-tasking across the biofilm (Fig. S6).

**Fig 6:**
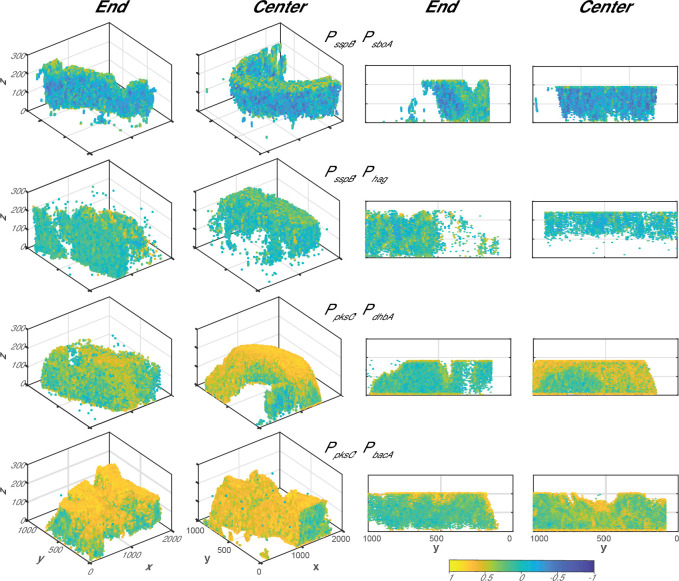
Correlations between pairs of reporters from confocal microscopy. Pearson correlation coefficients were determined between the fluorescent intensities of the two genes expressed in the dual-reporter strains of *Bacillus subtilis*; the reporters are indicated in the center of each row. “End” indicates the periphery (edge) of the colony while “Center” indicates the center of the biofilm colony. The left two panels are three-dimensional representations of the colony slice and the right two panels are cross-sections of these images. Yellow indicates highly correlated fluorescence between the two reporters within each strain, while dark blue indicates anti-correlated fluorescence. Areas with no fluorescence are not represented in these images.

### Correlating reporters to each other using microscopy

We then examined the spatial relationships between different fluorescent reporters within each reporter strain of *B. subtilis* based on the confocal fluorescence micrographs (Fig. S5). This was done as a voxel-based analysis using BiofilmQ (60). We derived Pearson correlation coefficients for the expression intensity of the two expressed genes for each strain and displayed this value onto the centroid location of the voxel. A range of distinct relationships were present between different reporter pairs (Fig. 6), supporting the diversity of relationships present between each set of genes as observed in the flow cytometry data. The expression of *sspB* and *sboA* was anti-correlated, while *pksC* and *bacA* expression was correlated in the center and on the edge of the biofilm. Interestingly, *pksC* and *dhbA* expression was correlated in the center but somewhat anticorrelated in the periphery, suggesting that a *pksC-* and *dhbA-*active cell type is present in the center of the biofilm (Fig. 6). These cross-sections provide an even more nuanced view of how different gene pairs are related to one another throughout the biofilm colony.

## DISCUSSION

Historically, researchers have classified *B. subtilis* into several cell states or subpopulations that were identified based on the gene expression inferred by a handful of fluorescent transcriptional reporters (28). In this study, we have built upon these foundational studies and generated 182 strains containing all pairwise combinations (91 reporter pairs in both color combinations) of a suite of 14 fluorescent transcriptional reporters that report on the gene expression of described cell physiologies as well as genes that encode specialized metabolite machinery in *B. subtilis*. By analyzing the spatial and co-expression relationships of genes controlling these critical phenotypes and the production of cell–cell metabolite signals within *B. subtilis* biofilms, we have uncovered which genes are simultaneously expressed within *B. subtilis* cells, enhancing our understanding of how extensively cells within these biofilm cell populations are multi-tasking. Furthermore, the expression patterns of these genes are spatially distributed in a repeatable way across biofilms [shown here and in Ref. (51)]. Our ambitious evaluation of these many strains by both flow cytometry and microscopy has led to a substantially more nuanced and thorough view of cellular heterogeneity within *B. subtilis* biofilms.

Our study reveals a substantial level of multi-tasking within *B. subtilis* biofilm cells. Given the diverse reasons that cells differentiate (e.g., division of labor in producing extracellular matrix or in generating cells tolerant to antibiotics, etc.), it is possible that *B. subtilis* cells may multi-task to provide an additional layer of “bet-hedging” in the face of environmental stress. We frequently observed subpopulations of cells that co-expressed two reporters as well as subpopulations that only expressed one of the two reporters (Fig. 4 and Fig. S4). Our data indicate that 66% (60/91) of the phenotypes examined here are co-expressed in at least some *B. subtilis* biofilm cells (Fig. 4). Some unexpected co-expression patterns were revealed, which could lead to a better understanding of the spatial organization and regulation of cellular behavior within *B. subtilis* biofilms. For instance, *sspB* was also co-expressed with a substantial (16%) population of highly fluorescent *sdpA-*expressing cells and smaller proportions (~3%–7%) of *skf, aprE, comQX, sboA*-expressing cells (Fig. S4). These observations may merely reflect fluorophore overlap during differentiation transitions from one cell state to another, or the co-expression of proteases and specialized metabolites during sporulation may be facilitating sporulation success via their ability to lyse and cannibalize neighboring bacteria (39, 66, 67). In addition, the intensity of *sdpA* expression is not uniform throughout the biofilm. *hag* was co-expressed with lower-intensity *spdA* cells while most other genes were co-expressed with brighter-intensity *sdpA* cells (Fig. S4). Prior work indicates that SDP collapses the proton motive force of the *B. subtilis*’ cell wall, delays sporulation, and kills a variety of other bacterial species (44). Non-motile *B. subtilis* cells may benefit from higher levels of SDP production when they encounter or need to kill other bacteria. Some other low-intensity *sdpA-*expressing cells (in the interior of the biofilm; Fig. S5) instead co-express *aprE*; protease production possibly compensates for lower levels of *sdpA* expression in these areas of the biofilm.

Further work interrogating the broader, genome-wide gene expression patterns of individual cells within biofilms would be informative here. The global transcriptome of *B. subtilis* has been analyzed previously (68, 69), but data about the specific transcriptional profile of multi-tasking cells are masked when biofilms are harvested and analyzed in bulk. Previous studies have coupled fluorescence-activated cell sorting followed by RNA sequencing to examine competent (*comG*+) and non-competent subpopulations (70) and succinate co-A ligase (*sucC*+) populations (16) in *B. subtilis*. In addition, a recent study demonstrated the possibility of obtaining single-cell RNA-seq data from *B. subtilis* strain 168, albeit under very different growth conditions (71). We anticipate that such approaches, used in conjunction with our suite of dual-labeled fluorescent reporter strains, could be used to isolate and determine the transcriptome of specific subpopulations of *B. subtilis* cells. Expanding the current collection of reporters to include other genes implicated in cell–cell communication and biofilm formation (72 - 75) would provide an even more refined view of cellular transcriptional heterogeneity within bacterial biofilms.

*B. subtilis* and many other bacteria dedicate a large portion of their genome to specialized metabolite gene clusters (76, 77); many of the metabolites produced by these biosynthetic genes act as cell–cell signals (38). Many of the biosynthesis genes for specialized metabolites examined here were co-expressed and co-localized with other specialized metabolite and physiological reporter genes. This overlap suggests that the expression of specialized metabolite biosynthesis machinery may regulate the generation of other cellular phenotypes. In general, it appears that *comQX, sdpA, skfA,* and *bacA* are expressed in many cells throughout the biofilm. Nevertheless, in some areas of the biofilm, their expression intensity is non-uniform, and in some cases, the intensity co-varies across the genes (see patterns in Fig. S6). It is possible that these abundant transcripts are correlated for trivial reasons (e.g., those subsets of cells are merely more metabolically active) but they may instead be functionally co-regulated. Single-cell RNA-seq (71) or single-molecule fluorescence *in situ* approaches (78) would help uncover potential co-regulation of these genes within individual cells. Because little is known about the role of many metabolites within *B. subtilis* biofilms, it is challenging to speculate what those co-regulated functions might be, although our recent work showing that ComX (*comQX*) is critical for biofilm formation, and bacilysin (*bacA*) has a role in sporulation (38) suggests that their co-expression could facilitate the overall progression of biofilm differentiation.

This work advances our understanding of the heterogeneity of cellular gene expression and transcriptional multi-tasking that exists within the biofilms of the model bacterium *B. subtilis*. However, the spatial transcriptional complexity we describe here is only a single snapshot of the potential cellular heterogeneity of which *B. subtilis* is likely capable over the lifetime of these biofilms, which we expect may be rooted in early biofilm differentiation processes. Better understanding the relationships between the different multi-tasking cell states described here and their co-regulation will require additional integrative multi-modal studies. In addition to traditional mutagenesis to dysregulate gene expression and dissect gene regulation systems (23, 30, 31, 37, 38), optogenetic control of gene expression (79) would enable the spatial manipulation of transcription in particular spatial regions of the biofilm. Bulk single-cell transcriptomics (71) of biofilms would reveal additional possible transcriptional cell states while *in situ* and spatial single-molecule transcriptomic approaches (78) would enable those cells to be spatially assigned to specific regions of the biofilm. Integration of spatial metabolic and proteomic imaging techniques (51, 80, 81) can resolve how far potential metabolite signals physically diffuse from the producing cells. Finally, modeling approaches that integrate these diverse data sets would enable the generation of an even more comprehensive and predictive spatial model of specialized metabolite signaling in bacterial biofilms.

Beyond the diversity of intraspecies gene expression observed within this single-species *B. subtilis* biofilm, we also anticipate that these gene expression patterns will be further modified by interactions with other bacteria and fungi. We know that multiple other bacteria can affect *B. subtilis* physiology based on single-gene reporter constructs (45, 82, 83) and predict that these changes are representative of shifts in the balance of transcriptional heterogeneity that are propagated across many other genes. The tools and approaches we implemented here could similarly be utilized to address the question of cellular transcriptional heterogeneity within multi-species communities and how their spatial organization may shift in response to environmental stressors. This research, which provides an unusually complete depiction of how *B. subtilis* differentiates within biofilms, provides a foundation for exploring more complex metabolic and regulatory interactions between cells within environmentally and agriculturally important microbial communities.

## Data Availability

The code for spatial quantification of fluorescence over the bacterial colonies can be found at https://doi.org/10.5281/zenodo.4624987. Images of all flow cytometry plots are available in the supplemental material section as Fig. S4. Images of a single Z-section of confocal microscopy images are included in the supplemental material as Fig. S5. All data used to assemble the manuscript figures, as well as flow cytometry data and colony and confocal fluorescence microscopy images, are available at https://doi.org/10.5061/dryad.f4qrfj71n.

## SUPPLEMENTAL MATERIAL

FIG S1 msystems.00891-22-s0001.pdf Colony morphology of *B. subtilis* wild-type and *B. subtilis* PsunA-YPet grown at 30°C on the biofilm-inducing medium MSgg for 48 h. This same phenotype was apparent when both mTurq and YPet versions of the reporter were used, and across three colonies from each phage transformation. Colony images were taken from the top using a dissecting stereomicroscope. Bar, 1 mm.

FIG S2 msystems.00891-22-s0002.pdf Average fluorescent pixel intensity of *B. subtilis* biofilms containing single *YPet* reporters depicted in Fig. 1. Intensity is plotted (black line) from the center (x-axis = 0) to the edge of the colony (x-axis = 100). The baseline autofluorescence detected in the agar is displayed by the dashed red line. 95% confidence intervals are indicated by grey dashed lines.

FIG S3 msystems.00891-22-s0003.pdf Growth of wild-type and a representative subset of single- and dual-reporter *B. subtilis* strains measured by OD600 measurements over time. Baseline corrected using blank MSgg medium.

FIG S4 msystems.00891-22-s0004.pdf Flow cytometry profiles of *B. subtilis* reporters pairs. (A) Histograms from single-color reporters and (B) fluorescence plots from dual-reporter strains.

FIG S5 msystems.00891-22-s0005.pdf Microscopy images of biofilm thin-sections were taken at two or three fields of view across the biofilm for each strain (see Table S1 for complete list of micrographs collected from which strains) at the approximate positions noted in the biofilm schematic in Fig. S6. Images shown here are single Z-sections of confocal microscopy images that have had their levels linearly adjusted using identical settings (across all panels) in Photoshop to better enable the visualization of the bright pixels in these images. Raw microscopy files can be downloaded as described in the manuscript Data Availability section.

FIG S6 msystems.00891-22-s0006.pdf Schematic summary of distribution patterns of the fluorescent reporters from three radial regions of the biofilm from micrographs in Fig. S5. The intensity of each fluorophore is independent from the others.

TABLE S1 msystems.00891-22-s0007.xlsx Tab 1—Strains lists the primers, plasmids, and strains used in this study. Tab 2—Microscopy Data lists the strains from the study and indicates those for which microscopy images were taken. Tab 3—FC Data lists the strains for which flow cytometry plots are available and notes which Supplemental Figure they are included in.

ASM does not own the copyrights to Supplemental Material that may be linked to, or accessed through, an article. The authors have granted ASM a non-exclusive, world-wide license to publish the Supplemental Material files. Please contact the corresponding author directly for reuse.
