# Supplementary figures and images for: Extensive cellular multi-tasking within Bacillus subtilis biofilms
Source: mSystems. 2023 Aug 1;8(4):e00891-22. doi: 10.1128/msystems.00891-22 (PMC10469600; doi:10.1128/msystems.00891-22)

**Wild type**

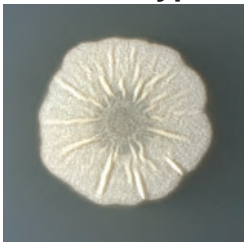

***sunA***

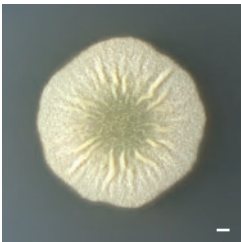

Supplement: FIG S1 — Colony morphology of B. subtilis wild-type and B. subtilis PsunA-YPet grown at 30°C on the biofilm-inducing medium MSgg for 48 h. This same phenotype was apparent when both mTurq and YPet versions of the reporter were used, and across three colonies from each phage transformation. Colony images were taken from the top using a dissecting stereomicroscope. Bar, 1 mm. [file msystems.00891-22-s0001.pdf]

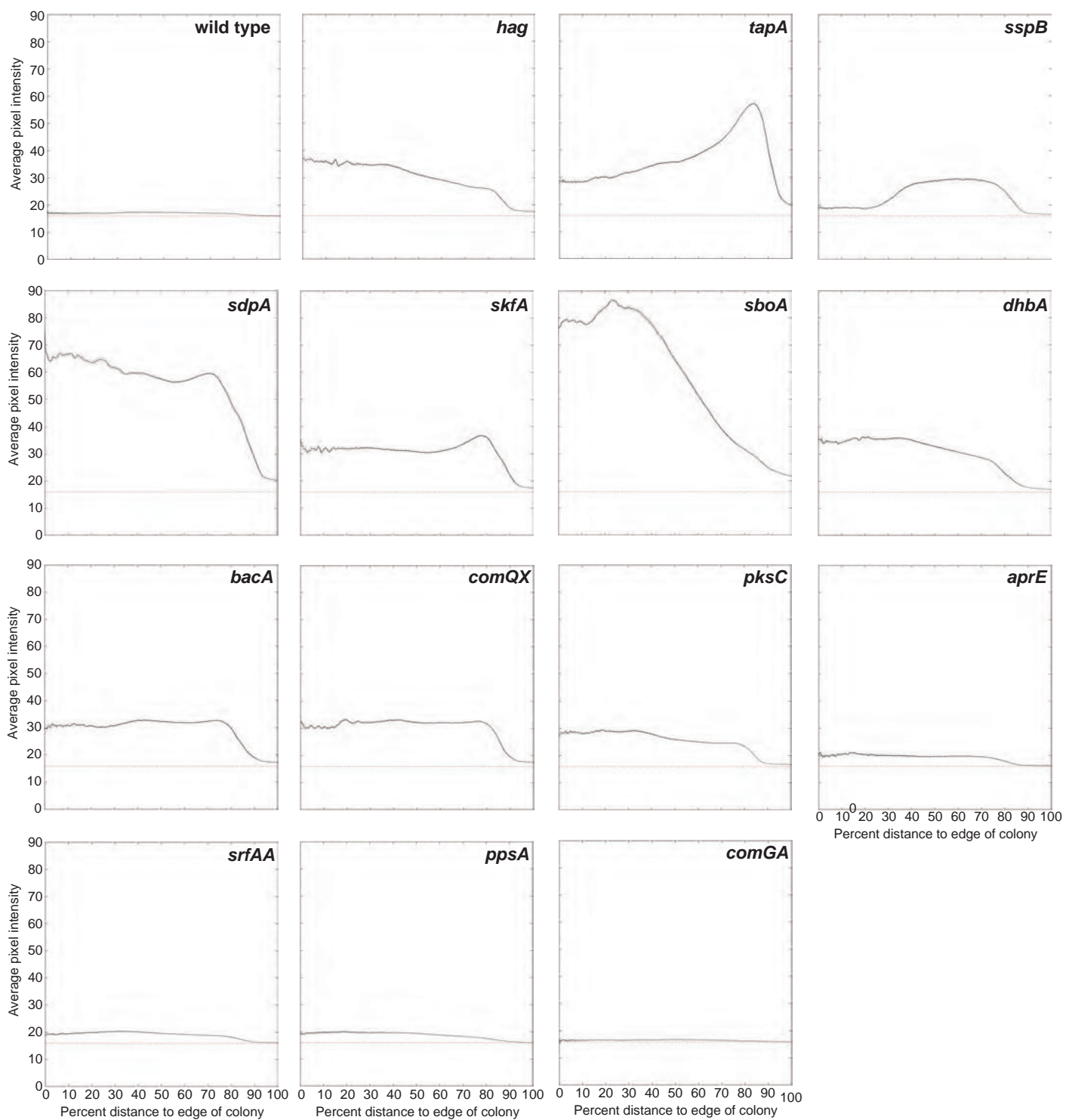

Supplement: FIG S2 — Average fluorescent pixel intensity of B. subtilis biofilms containing single YPet reporters depicted in Fig. 1. Intensity is plotted (black line) from the center (x-axis = 0) to the edge of the colony (x-axis = 100). The baseline autofluorescence detected in the agar is displayed by the dashed red line. 95% confidence intervals are indicated by grey dashed lines. [file msystems.00891-22-s0002.pdf]

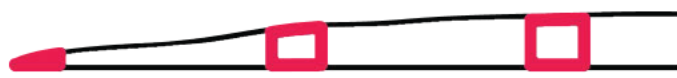

Periphery

Middle

Interior

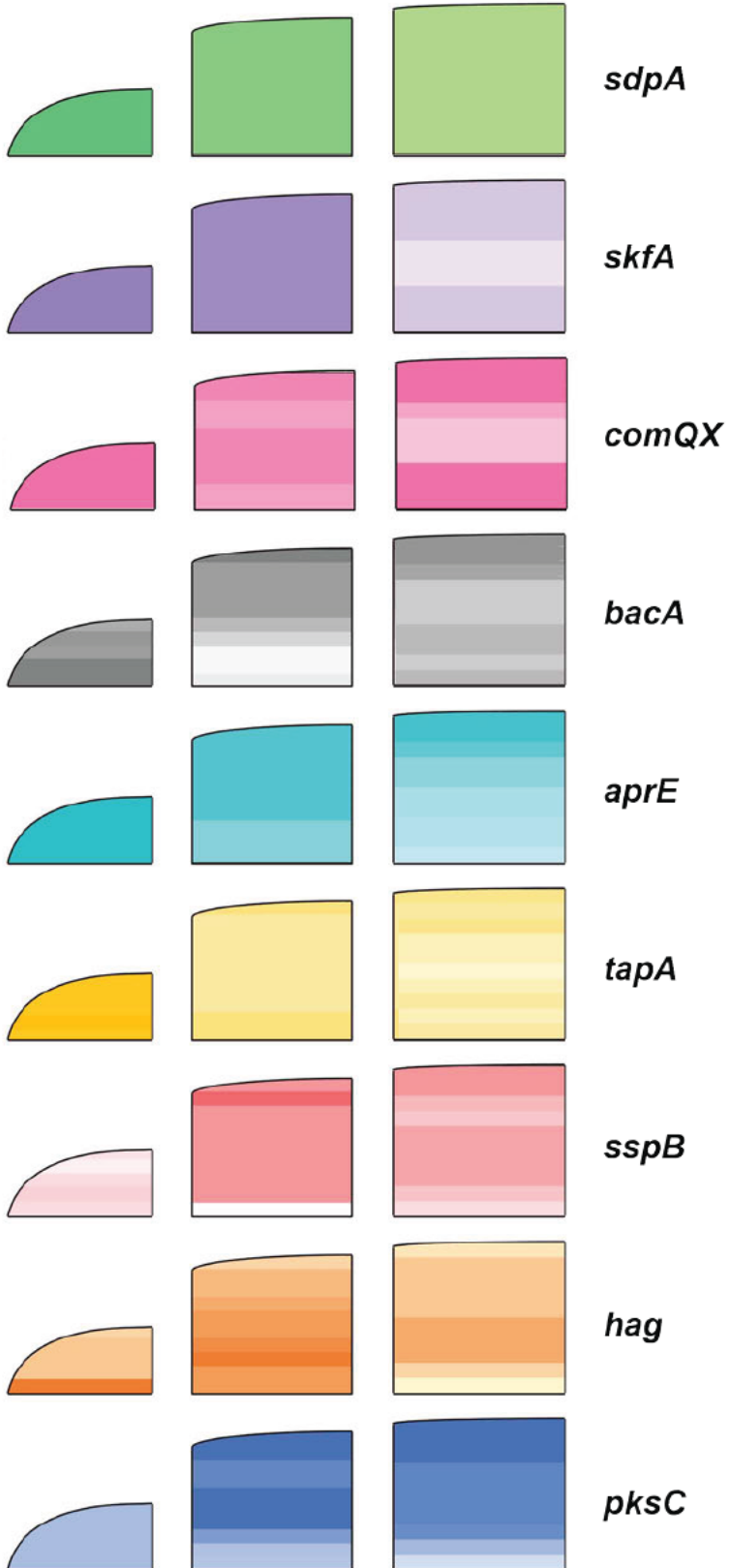

Supplement: FIG S6 — Schematic summary of distribution patterns of the fluorescent reporters from three radial regions of the biofilm from micrographs in Fig. S5. The intensity of each fluorophore is independent from the others. [file msystems.00891-22-s0006.pdf]
