# Supplementary material for: Extensive cellular multi-tasking within Bacillus subtilis biofilms
Source: mSystems. 2023 Aug 1;8(4):e00891-22. doi: 10.1128/msystems.00891-22 (PMC10469600; doi:10.1128/msystems.00891-22)
Supplement: FIG S3 — Growth of wild-type and a representative subset of single- and dual-reporter B. subtilis strains measured by OD600 measurements over time. Baseline corrected using blank MSgg medium. [file msystems.00891-22-s0003.pdf]

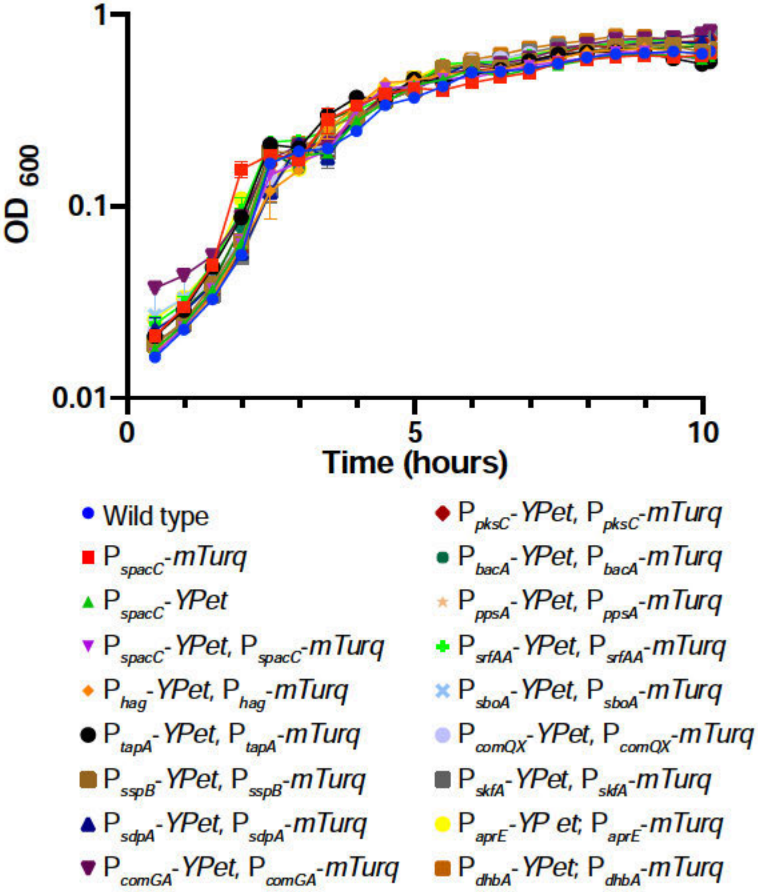

1
0.1
0.01
OD 600
0
5
10
Time (hours)
Wild type
P_{spacC}-mTurq
P_{spacC}-YPet
P_{spacC}-YPet, P_{spacC}-mTurq
P_{hag}-YPet, P_{hag}-mTurq
P_{tapA}-YPet, P_{tapA}-mTurq
P_{sspB}-YPet, P_{sspB}-mTurq
P_{sdpA}-YPet, P_{sdpA}-mTurq
P_{comGA}-YPet, P_{comGA}-mTurq
P_{pksC}-YPet, P_{pksC}-mTurq
P_{bacA}-YPet, P_{bacA}-mTurq
P_{ppsA}-YPet, P_{ppsA}-mTurq
P_{srfAA}-YPet, P_{srfAA}-mTurq
P_{sboA}-YPet, P_{sboA}-mTurq
P_{comQX}-YPet, P_{comQX}-mTurq
P_{skfA}-YPet, P_{skfA}-mTurq
P_{aprE}-YP et; P_{aprE}-mTurq
P_{dhbA}-YPet; P_{dhbA}-mTurq
