# Supplementary material for: Extensive cellular multi-tasking within Bacillus subtilis biofilms
Source: mSystems. 2023 Aug 1;8(4):e00891-22. doi: 10.1128/msystems.00891-22 (PMC10469600; doi:10.1128/msystems.00891-22)
Supplement: FIG S4 — Flow cytometry profiles of B. subtilis reporters pairs. (A) Histograms from single-color reporters and (B) fluorescence plots from dual-reporter strains. [file msystems.00891-22-s0004.pdf]

(A)

ES2090; AS128; amyE::PtapA-Ypet (cam)

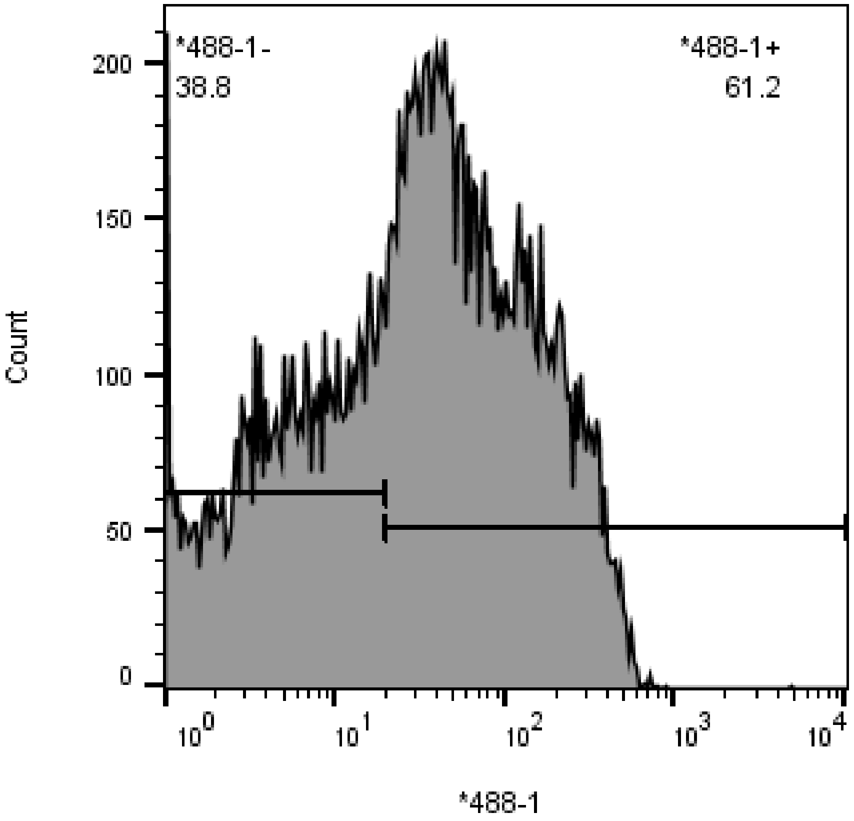

ES2091; AS129; amyE::PsdpA-Ypet (cam)

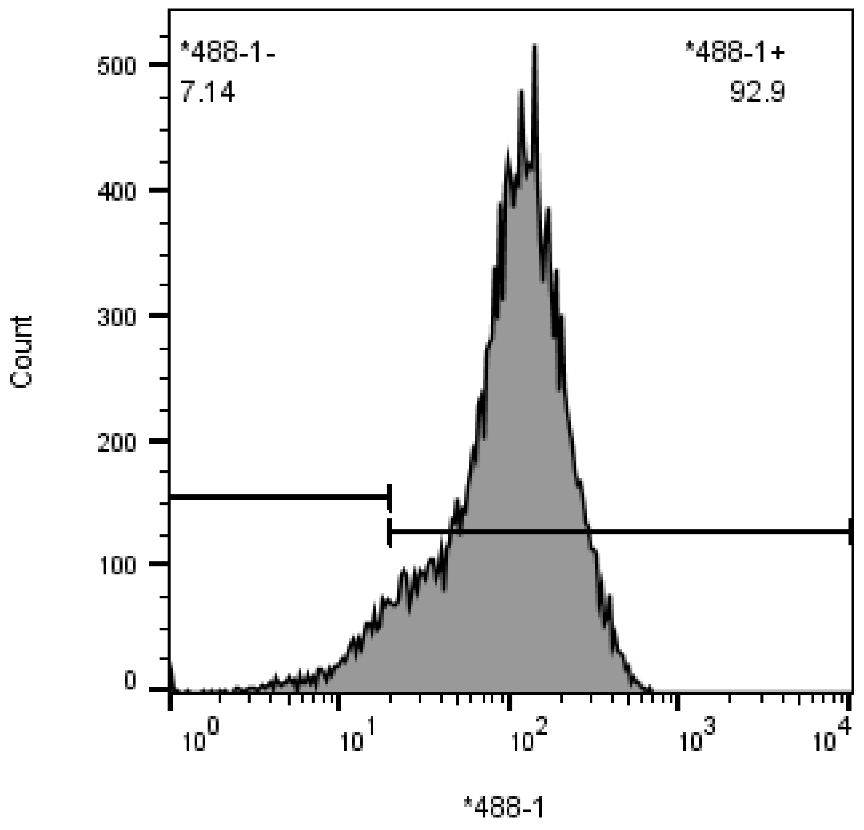

ES2092; AS130; amyE::PsspB-Ypet (cam)

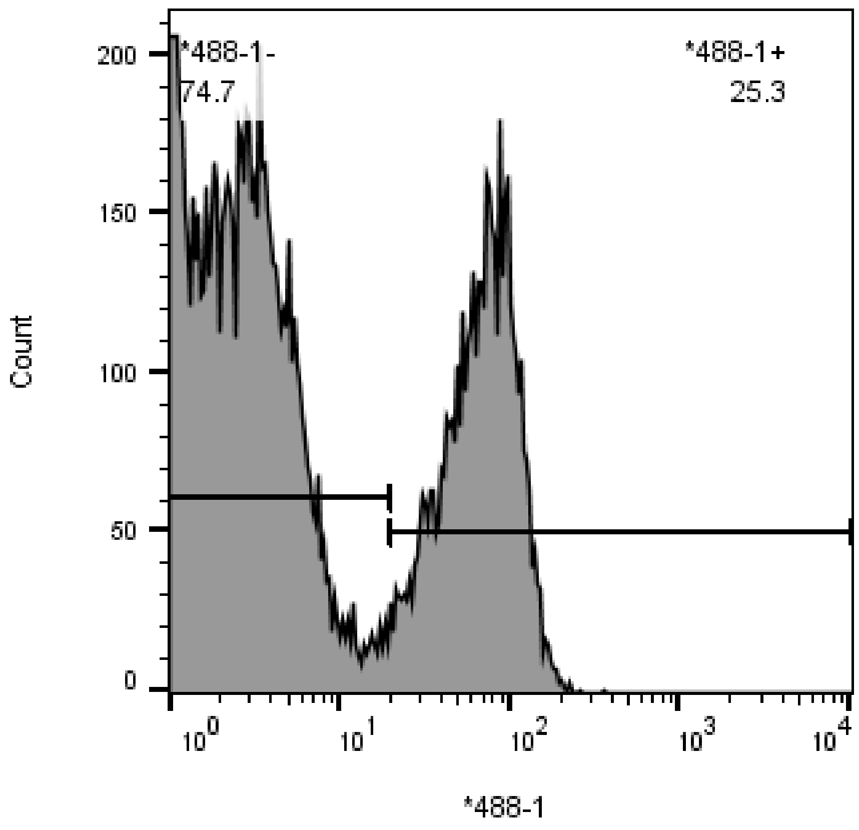

ES2093; AS131; amyE::PcomGA-Ypet (cam)

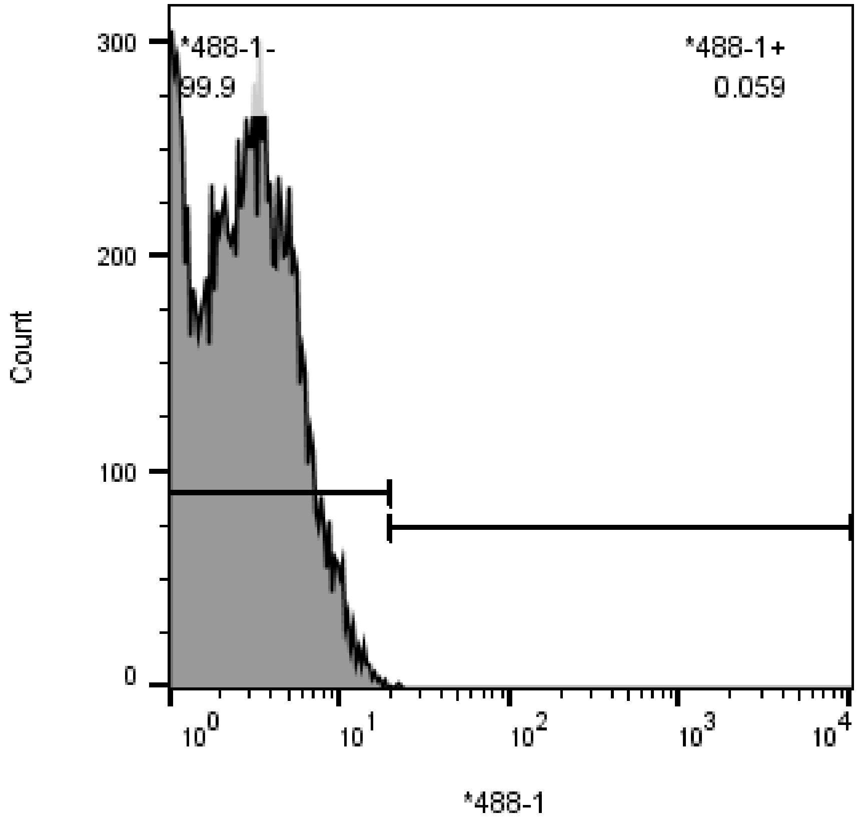

ES2095; AS133; amyE::PpksC-Ypet (cam)

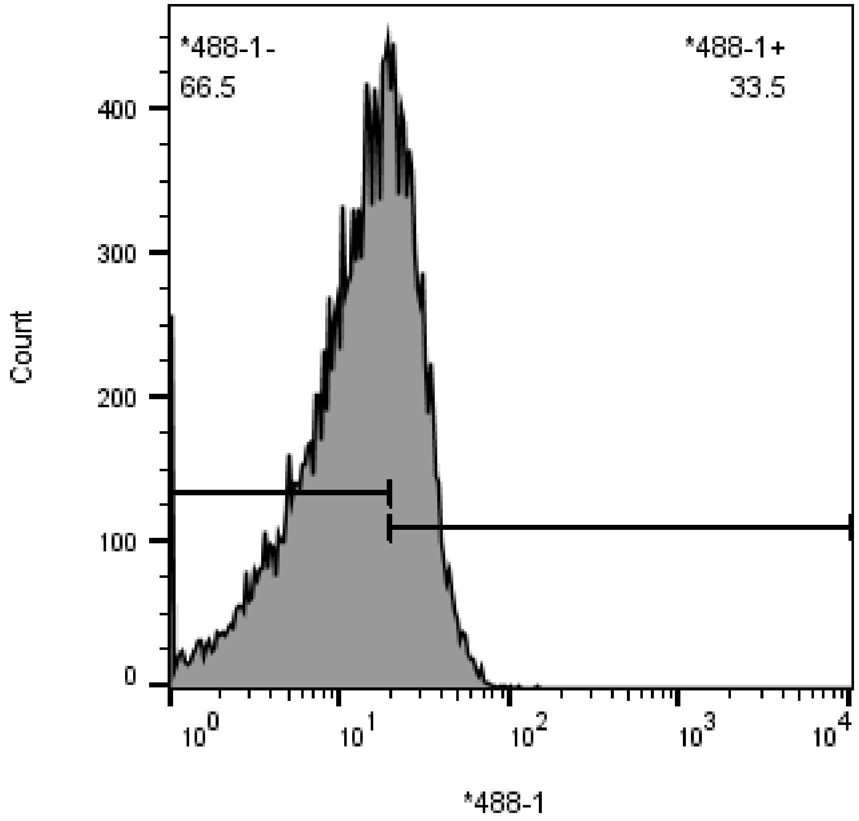

ES2096; AS134; amyE::PdhbA-Ypet (cam)

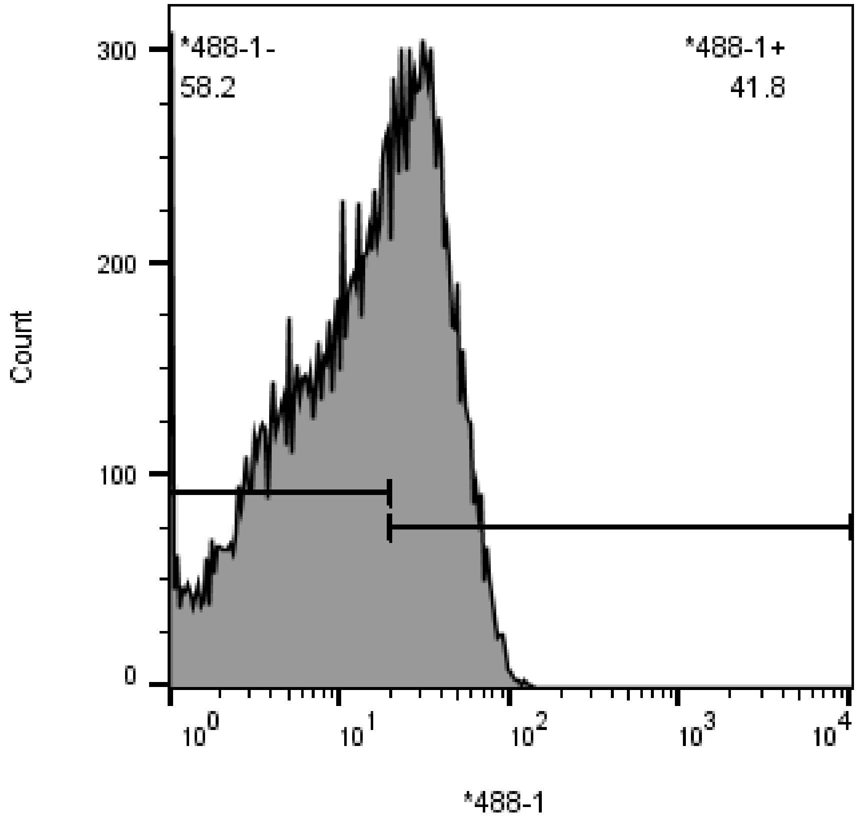

ES2097; AS135; amyE::PbacA-Ypet (cam)

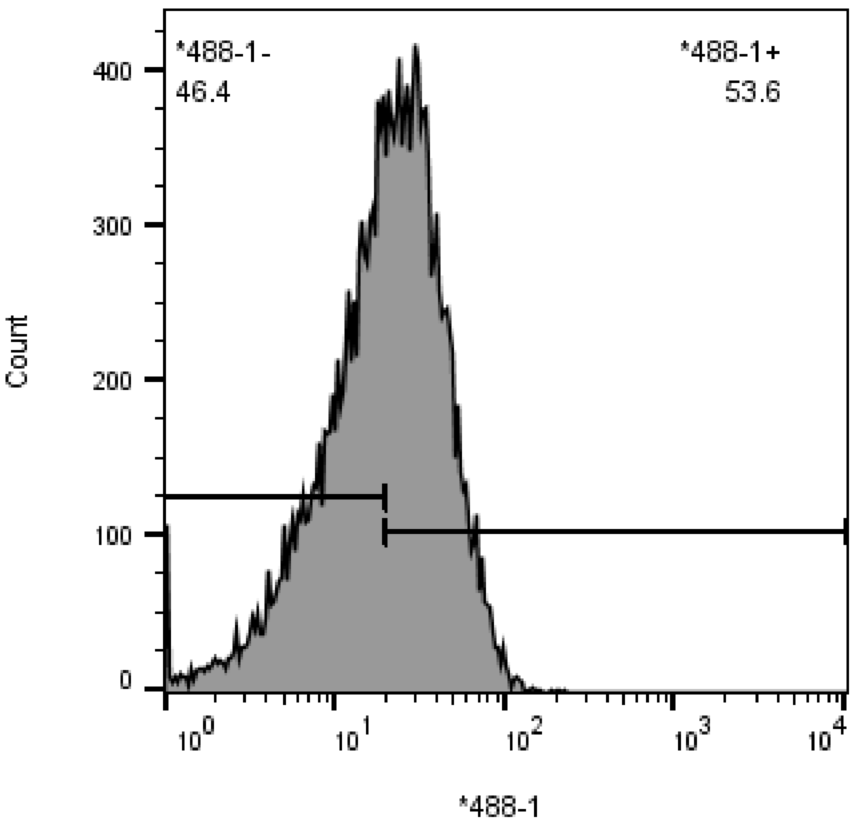

ES2098; AS136; amyE::PppsA-Ypet (cam)

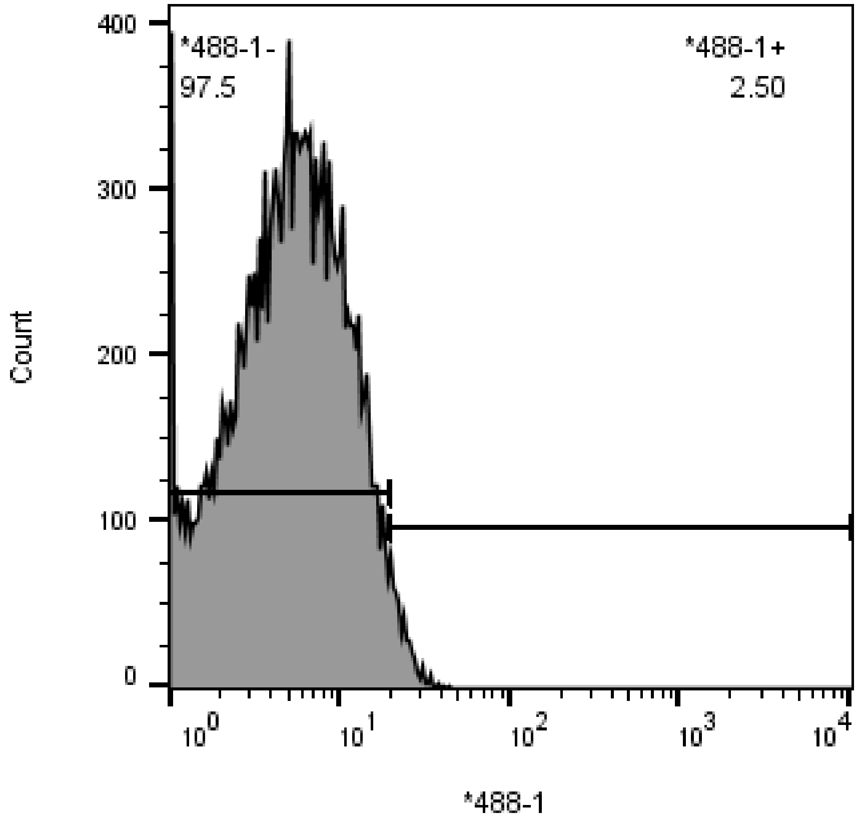

ES2099; AS137; amyE::PsrfAA-Ypet (cam)

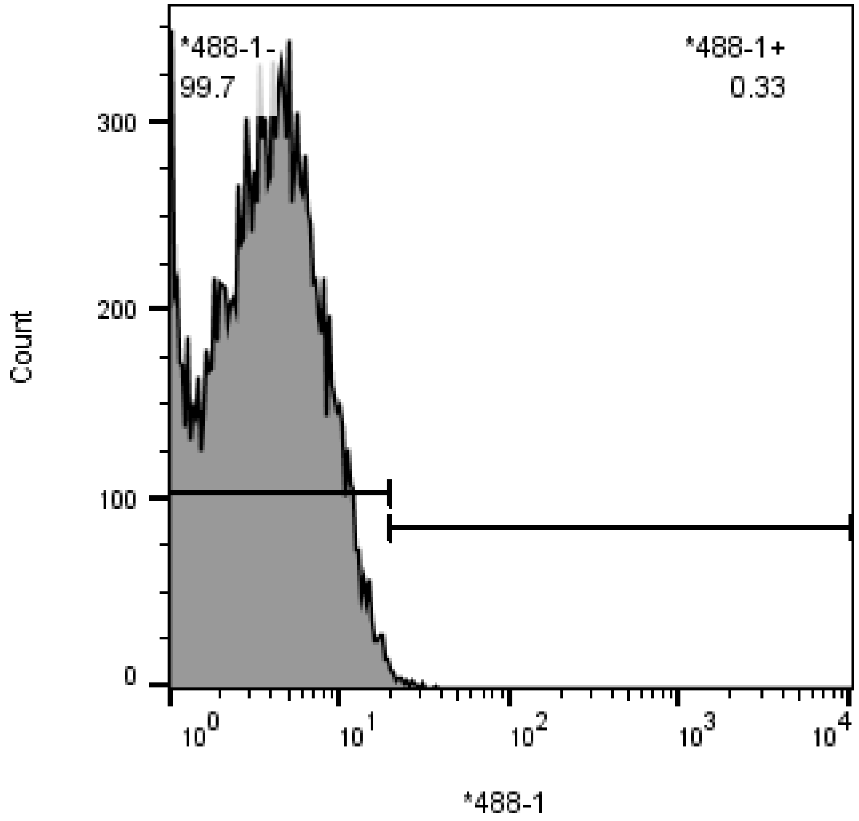

ES2100; AS138; amyE::PsboA-Ypet (cam)

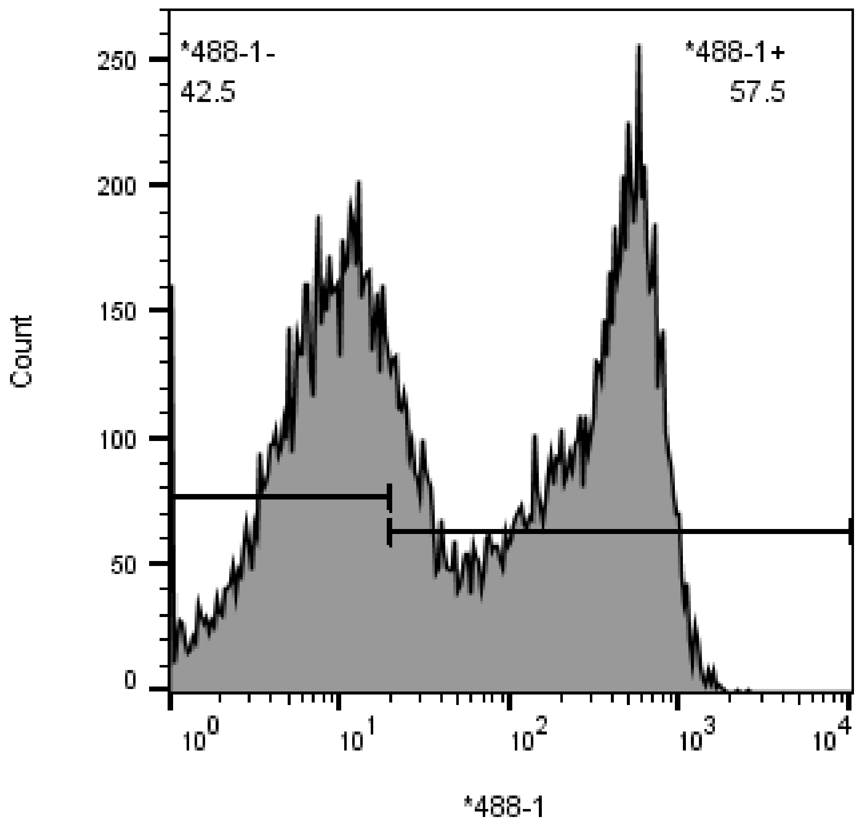

ES2102; AS140; amyE::PcomQX-Ypet (cam)

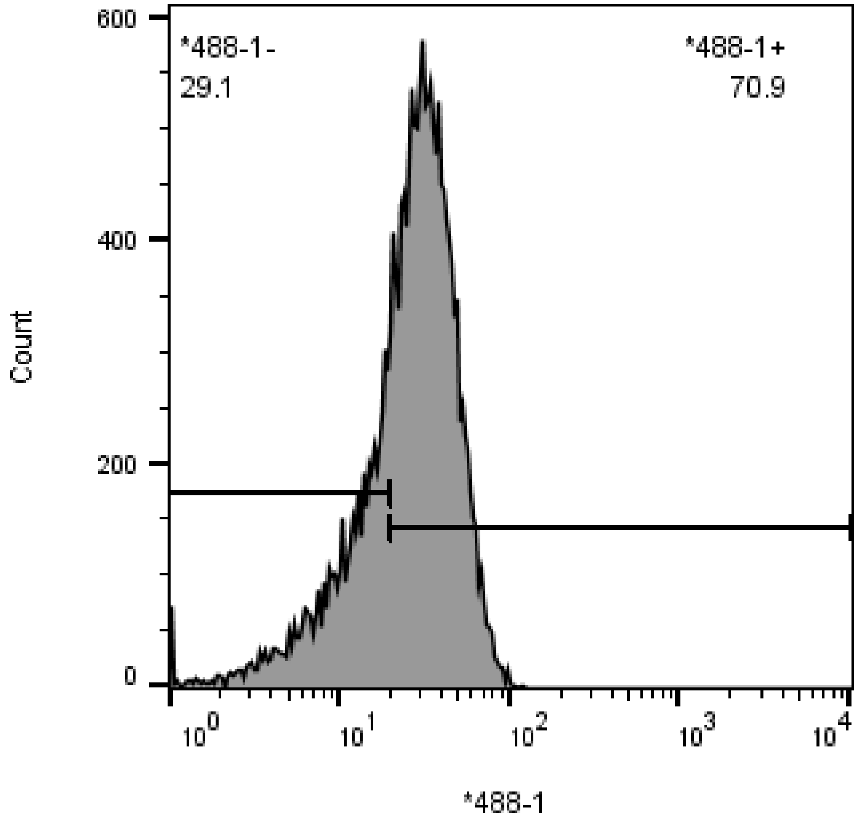

ES2142; SY297; AS158; amyE::PskfA-Ypet (cam)

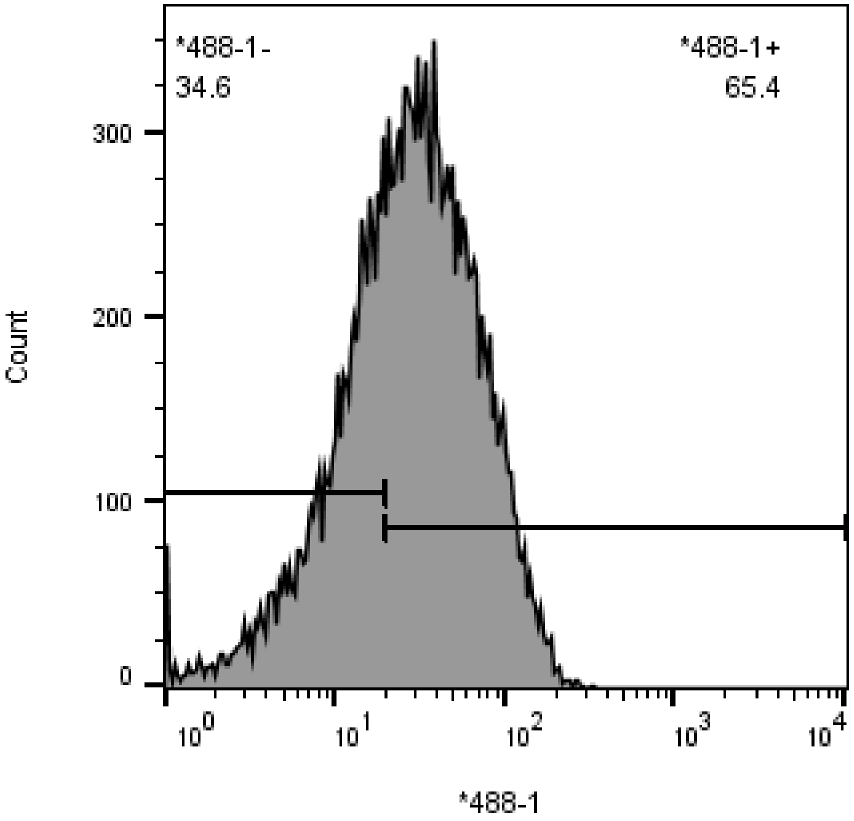

ES2059; SY252; lacA::Phag-mTurq (erm)

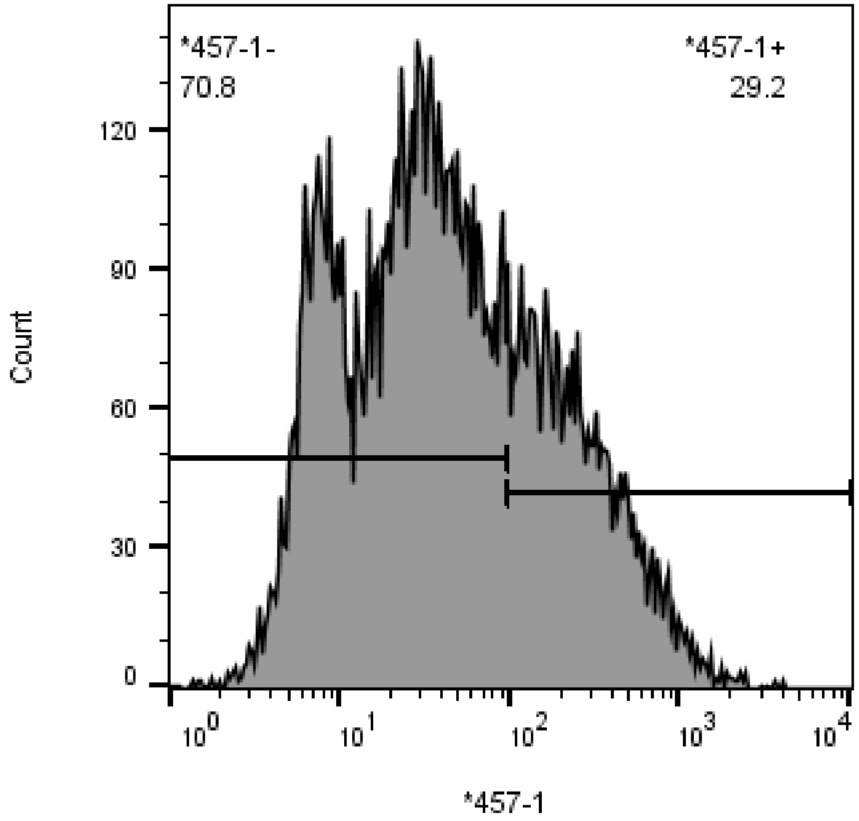

(B)

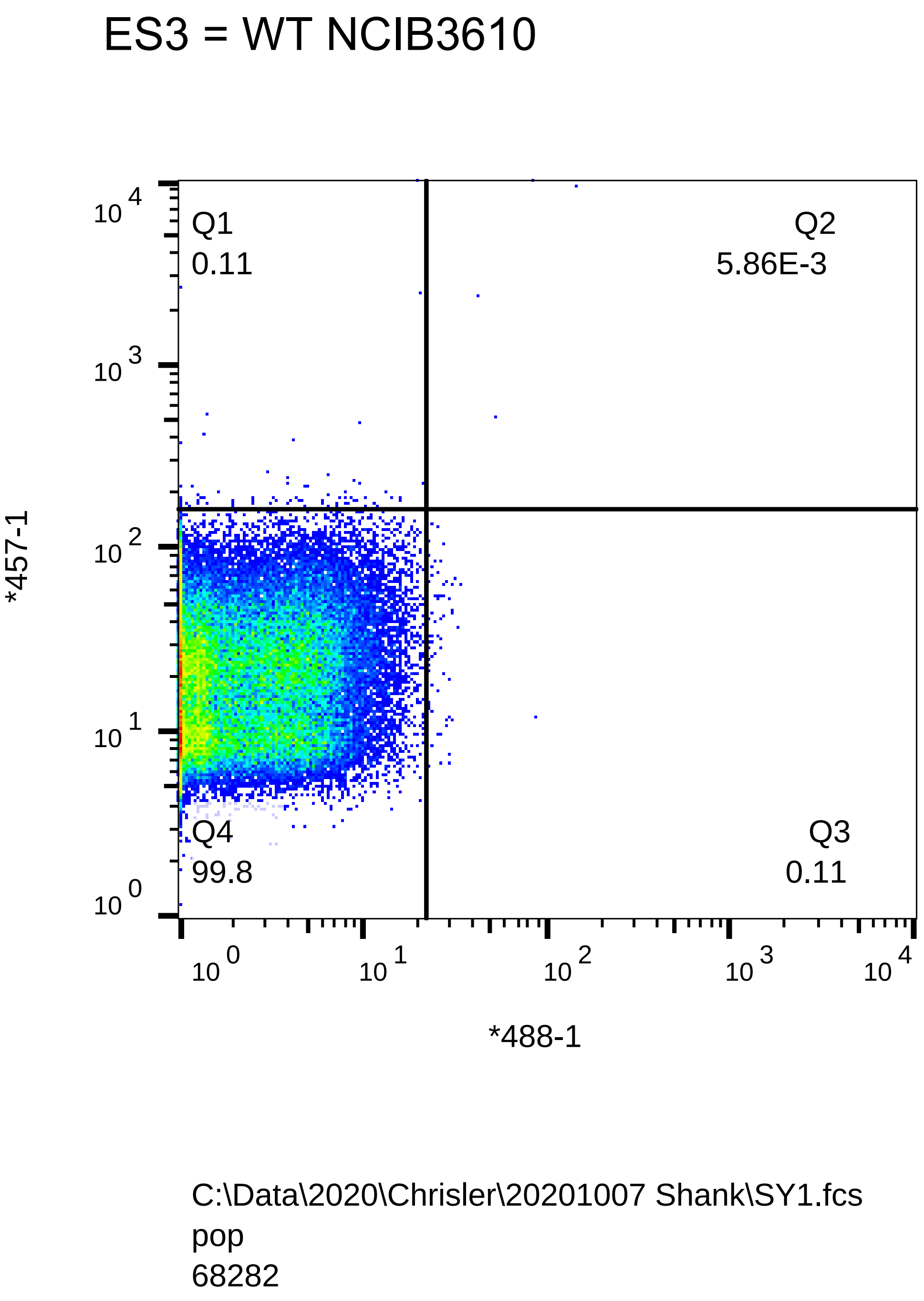
ES3 = WT NCIB3610
Q1
0.11
Q2
5.86E-3
Q4
99.8
Q3
0.11
*457-1
*488-1
C:\Data\2020\Chrisler\20201007 Shank\SY1.fcs
pop
68282

ES3 = NCIB3610 = WT

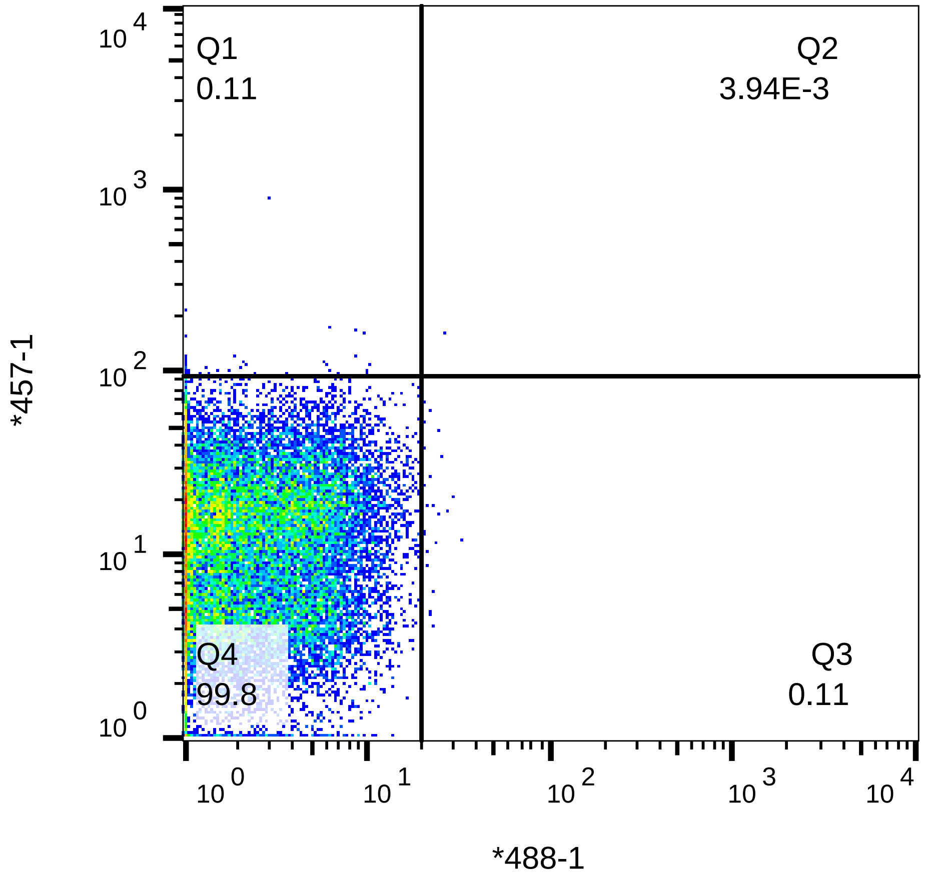

C:\Data\2019\Chrisler\20191219 Shank\No Fl Control.fcs
All pop
25353

ES2184; SY311; amyE::PtapA-Ypet (cam); lacA::Phag-mTurq (erm)

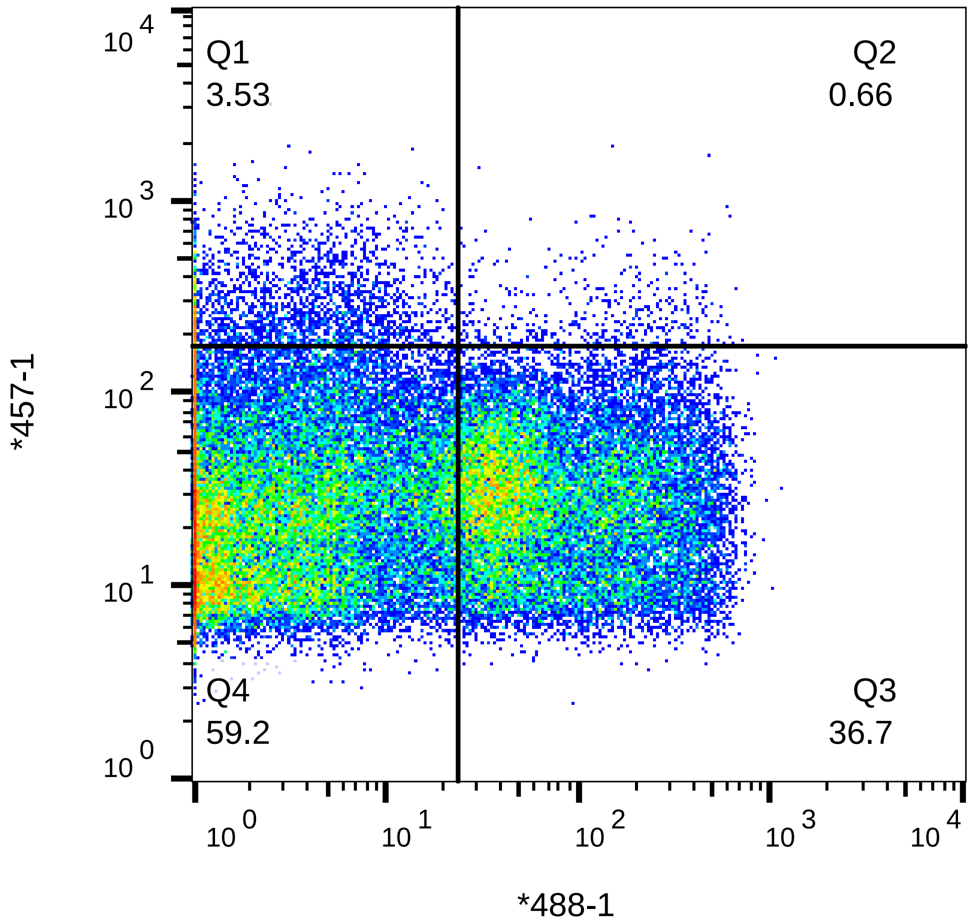

C:\Data\2020\Chrisler\20201007 Shank\SY311.fcs
pop
68573

ES2185; SY312; amyE::PtapA-Ypet (cam); lacA::PdhbA-mTurq (erm)

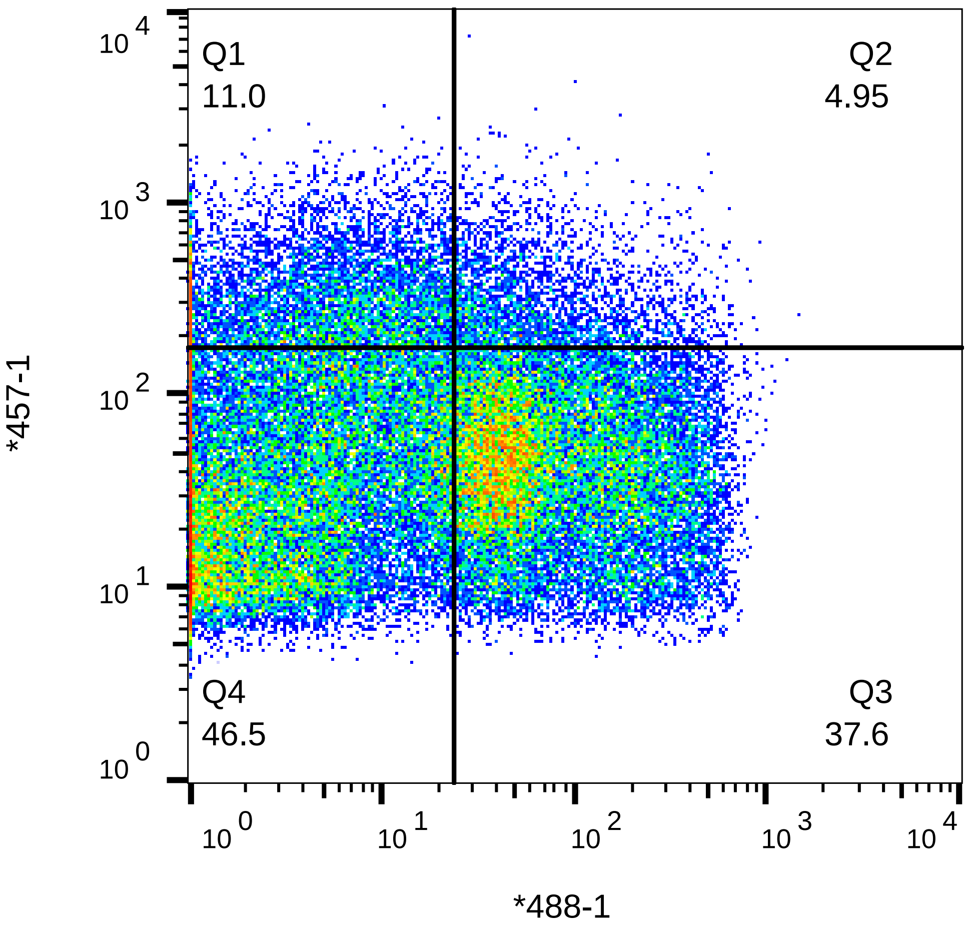

C:\Data\2020\Chrisler\20201007 Shank\SY312.fcs
pop
67690

ES2186; SY313; amyE::PtapA-Ypet (cam); lacA::PsdpA-mTurq (erm)

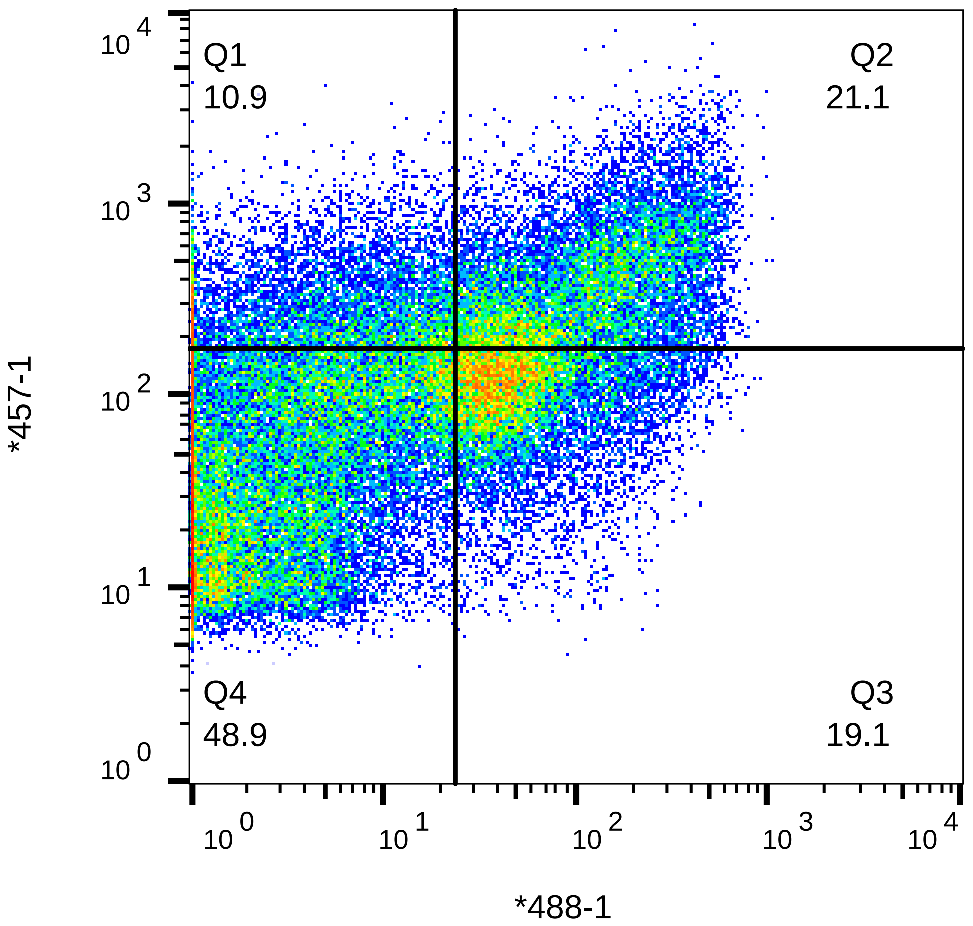

C:\Data\2020\Chrisler\20201007 Shank\SY313.fcs
pop
65602

ES2187; SY314; amyE::PtapA-Ypet (cam); lacA::PsspB-mTurq (erm)

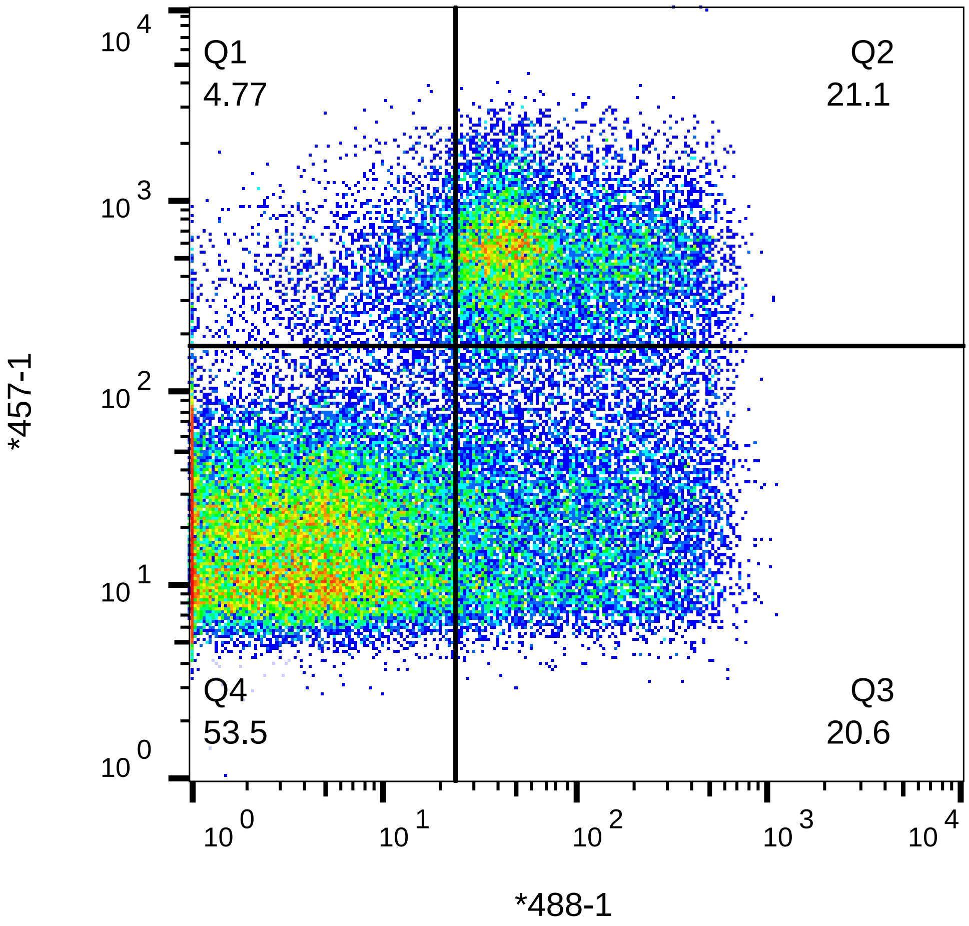

C:\Data\2020\Chrisler\20201007 Shank\SY314.fcs
pop
64487

ES2188; SY315; amyE::PtapA-Ypet (cam); lacA::PcomGA-mTurq (erm)

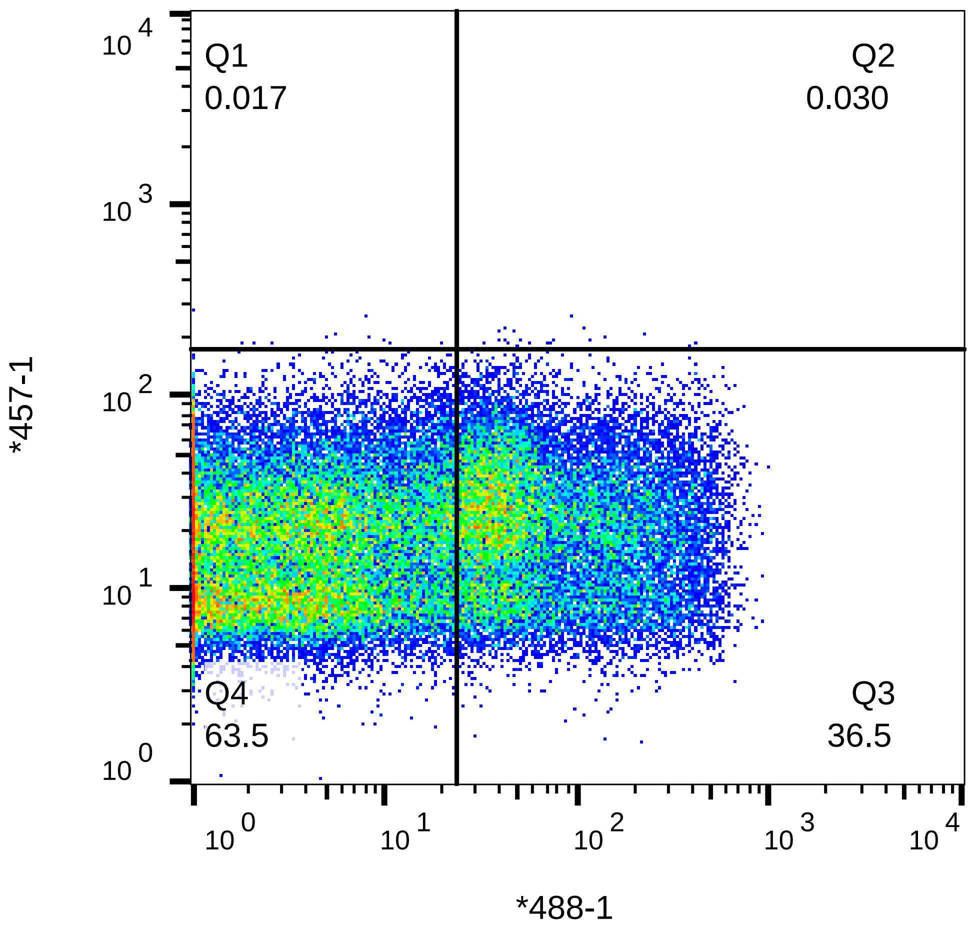

C:\Data\2020\Chrisler\20201007 Shank\SY315.fcs
pop
66206

ES2190; SY317; amyE::PtapA-Ypet (cam); lacA::PpksC-mTurq (erm)

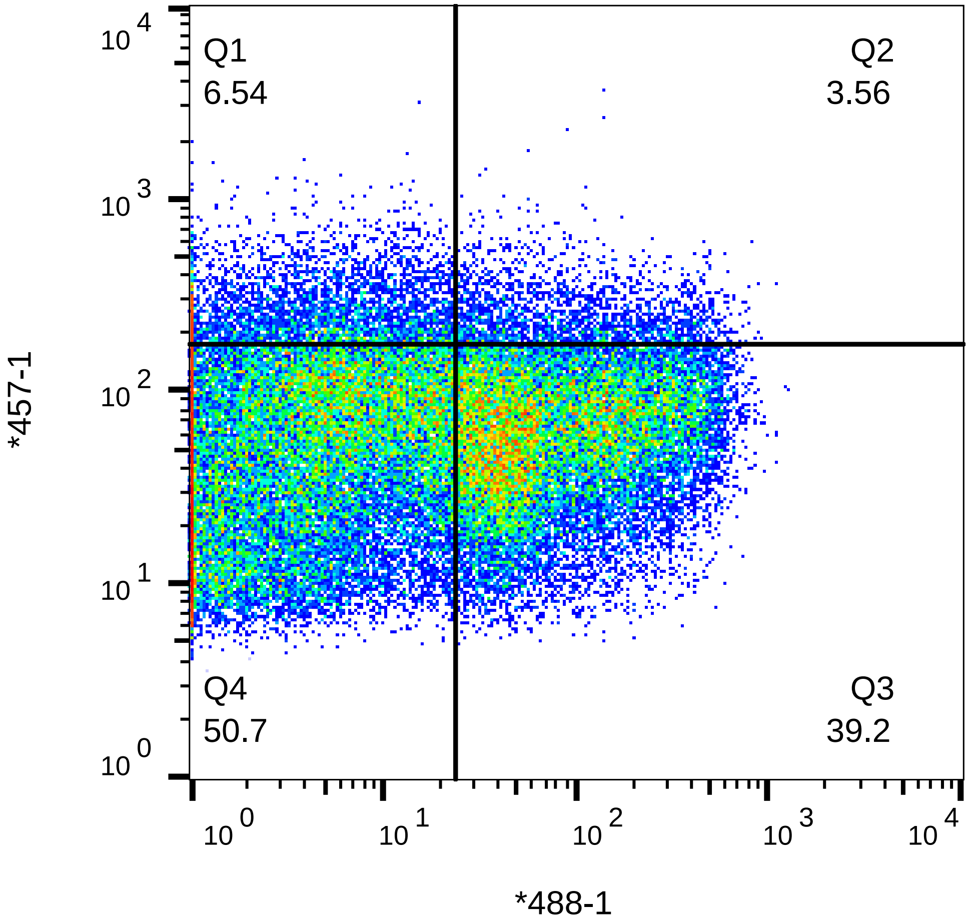

C:\Data\2020\Chrisler\20201007 Shank\SY317.fcs
pop
65009

ES2191; SY318; amyE::PtapA-Ypet (cam); lacA::PbacA-mTurq (erm)

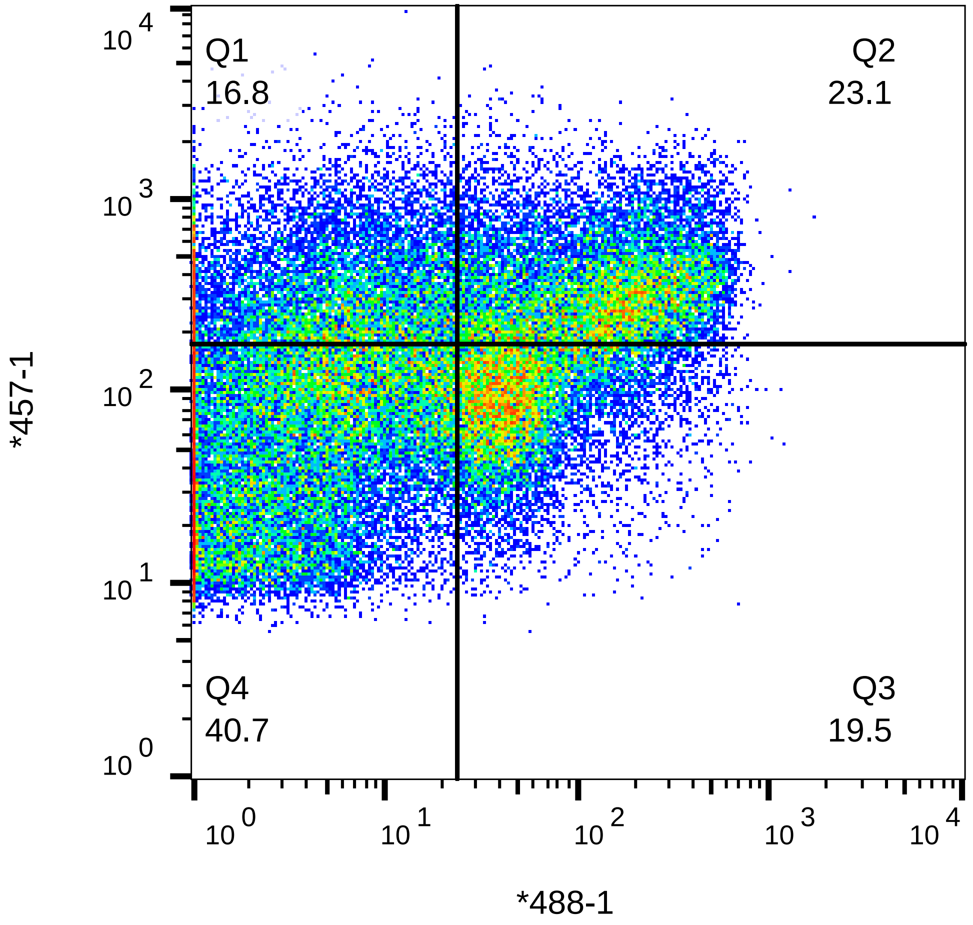

C:\Data\2020\Chrisler\20201007 Shank\SY318.fcs
pop
66193

ES2192; SY319; amyE::PtapA-Ypet (cam); lacA::PppsA-mTurq (erm)

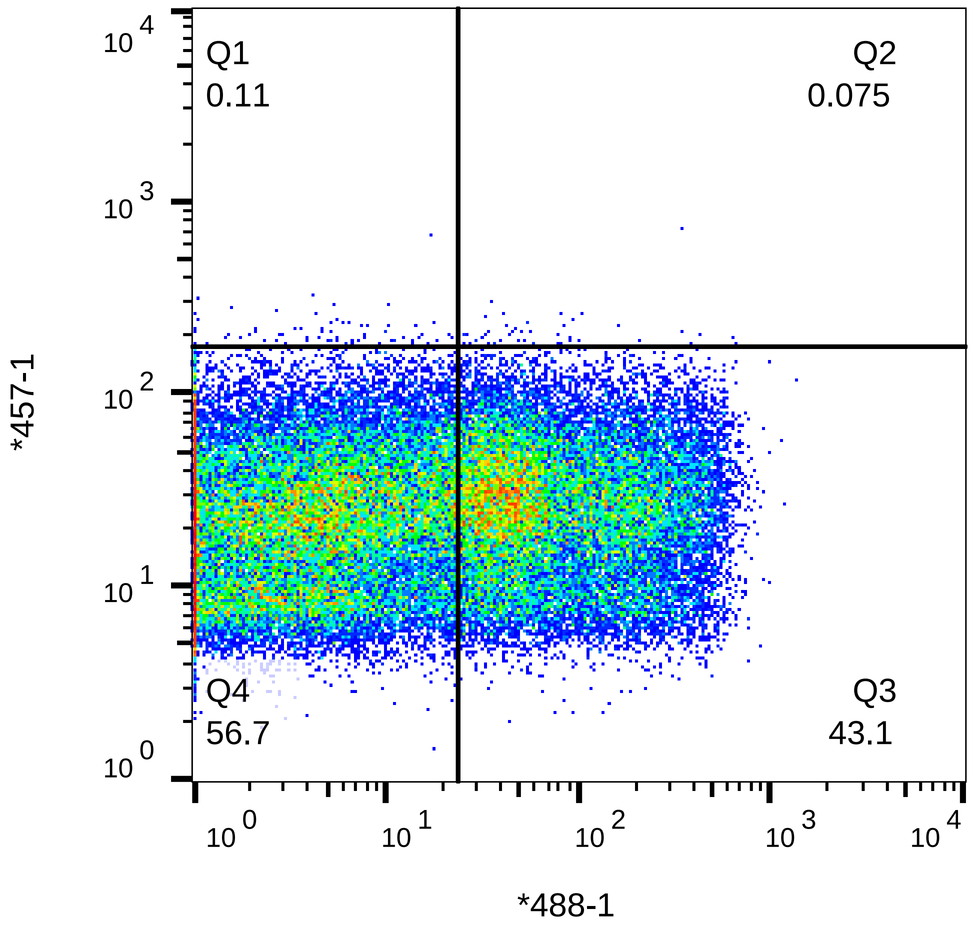

C:\Data\2020\Chrisler\20201007 Shank\SY319.fcs
pop
64429

ES2193; SY320; amyE::PtapA-Ypet (cam); lacA::PsrfAA-mTurq (erm)

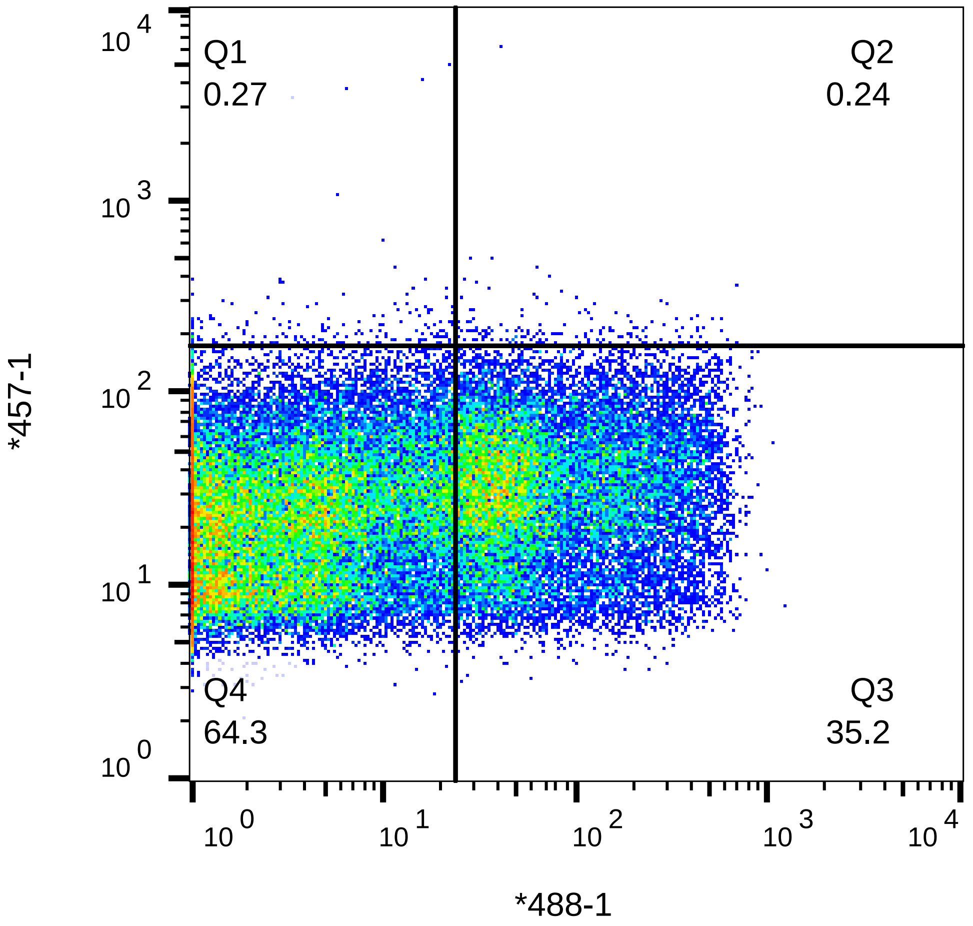

C:\Data\2020\Chrisler\20201007 Shank\SY320.fcs
pop
65751

ES2194; SY321; amyE::PtapA-Ypet (cam); lacA::PsboA-mTurq (erm)

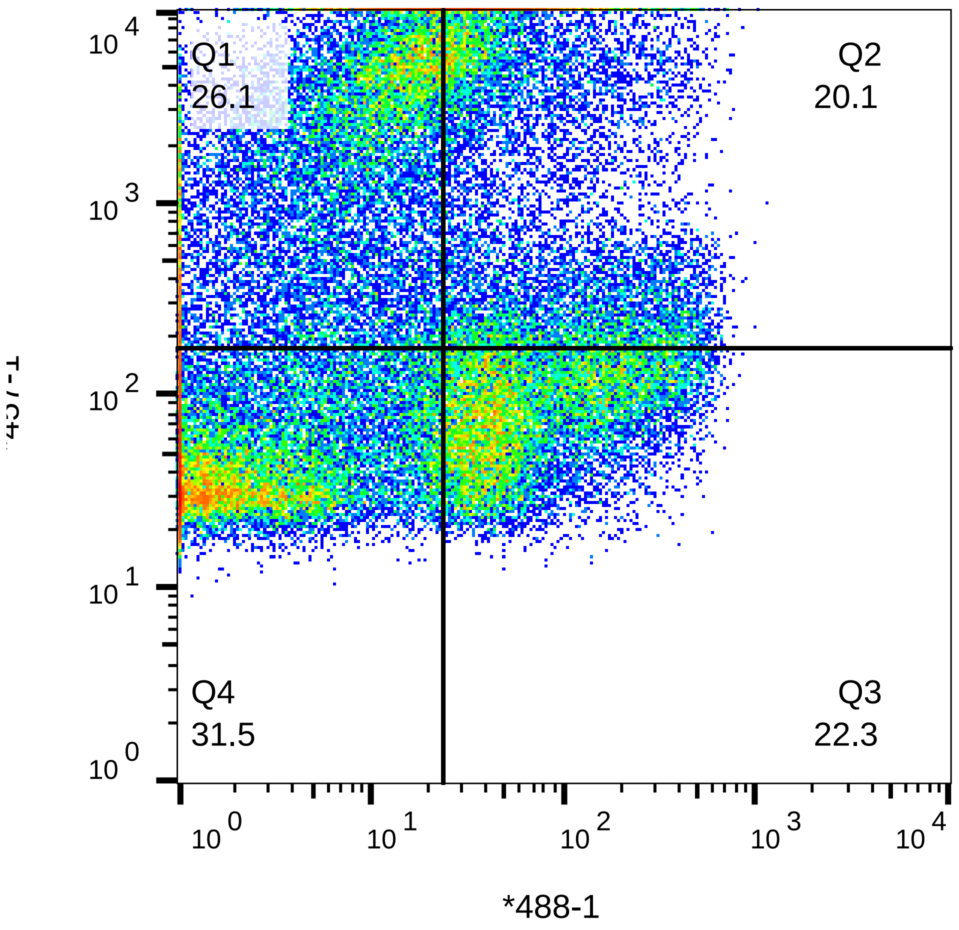

C:\Data\2020\Chrisler\20201007 Shank\SY321.fcs
pop
65475

ES2196; SY323; amyE::PtapA-Ypet (cam); lacA::PcomQX-mTurq (erm)

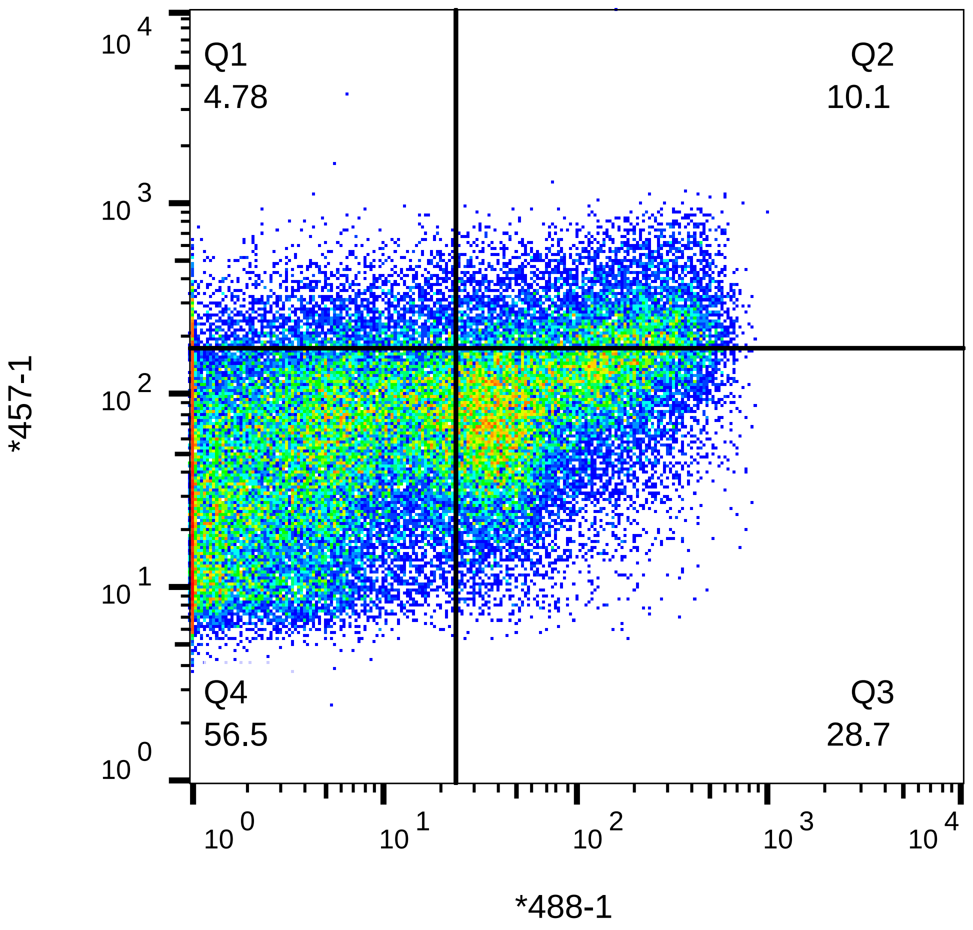

C:\Data\2020\Chrisler\20201007 Shank\SY323.fcs
pop
65052

ES2200; SY327; amyE::PtapA-Ypet (cam); lacA::PskfA-mTurq (erm)

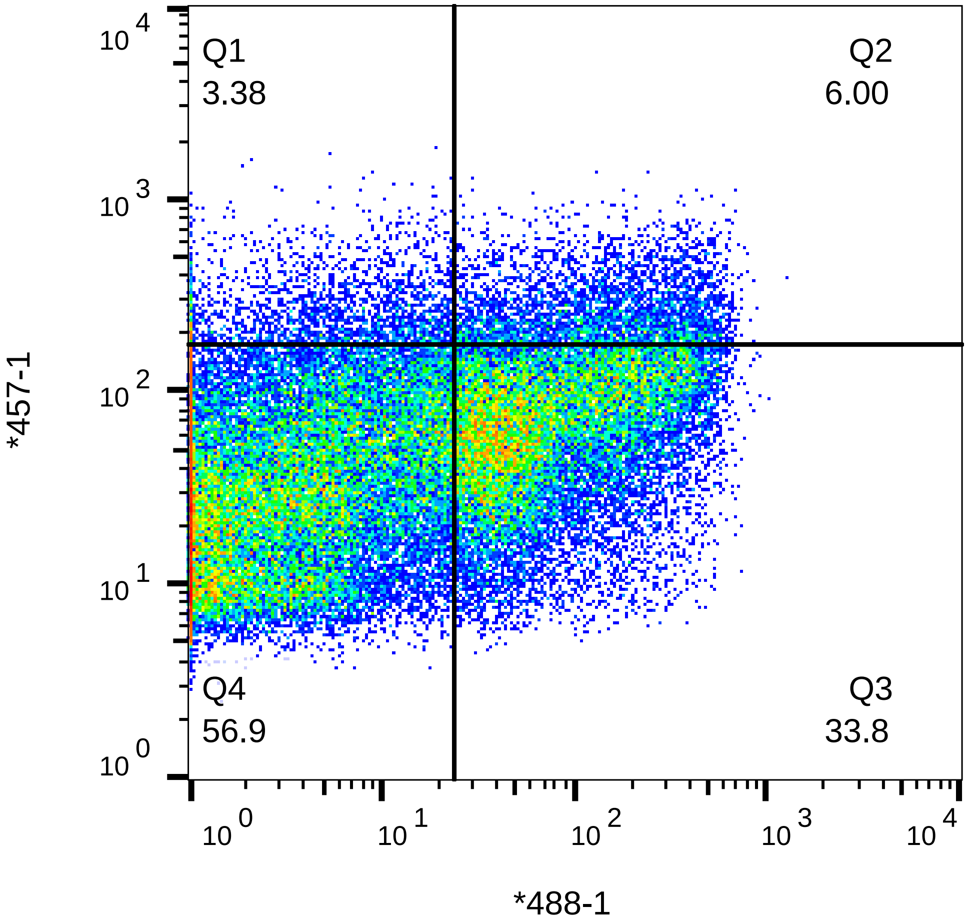

C:\Data\2020\Chrisler\20201007 Shank\SY327.fcs
pop
66176

ES2201; SY328; amyE::PtapA-Ypet (cam); lacA::PaprE-mTurq (erm)

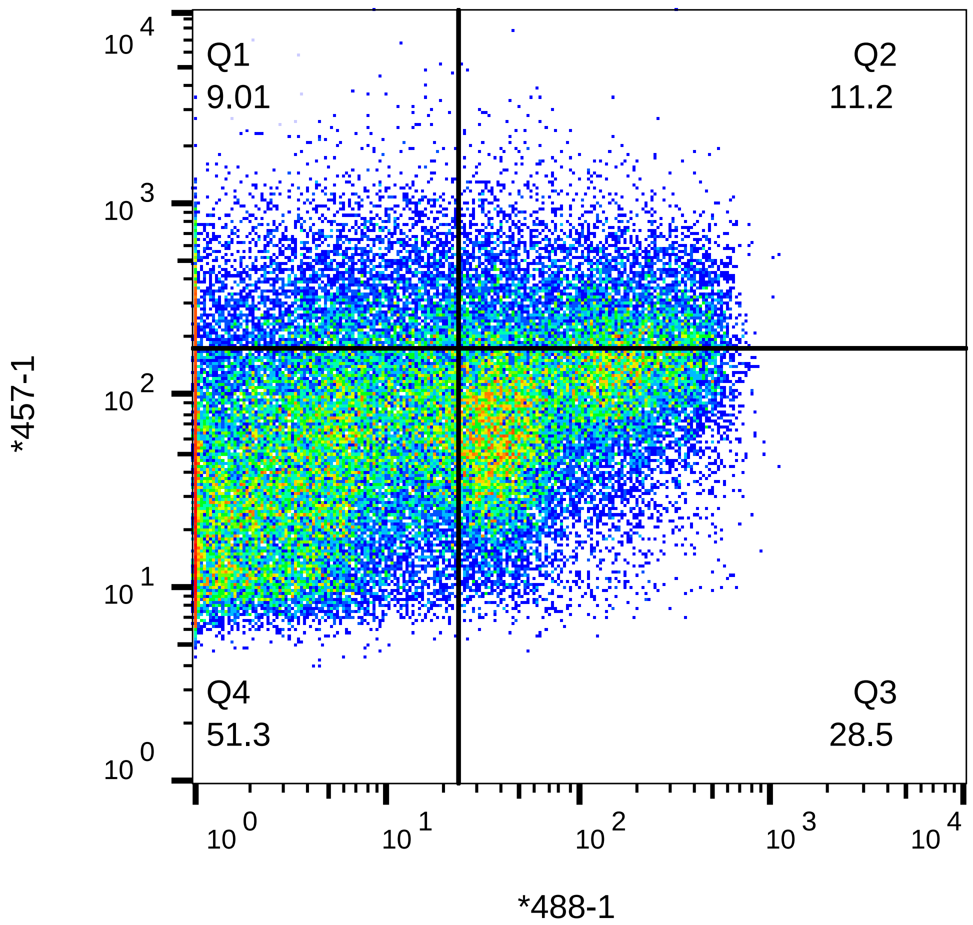

C:\Data\2020\Chrisler\20201007 Shank\SY328.fcs
pop
65260

ES2202; SY329; amyE::PsspB-Ypet (cam); lacA::Phag-mTurq (erm)

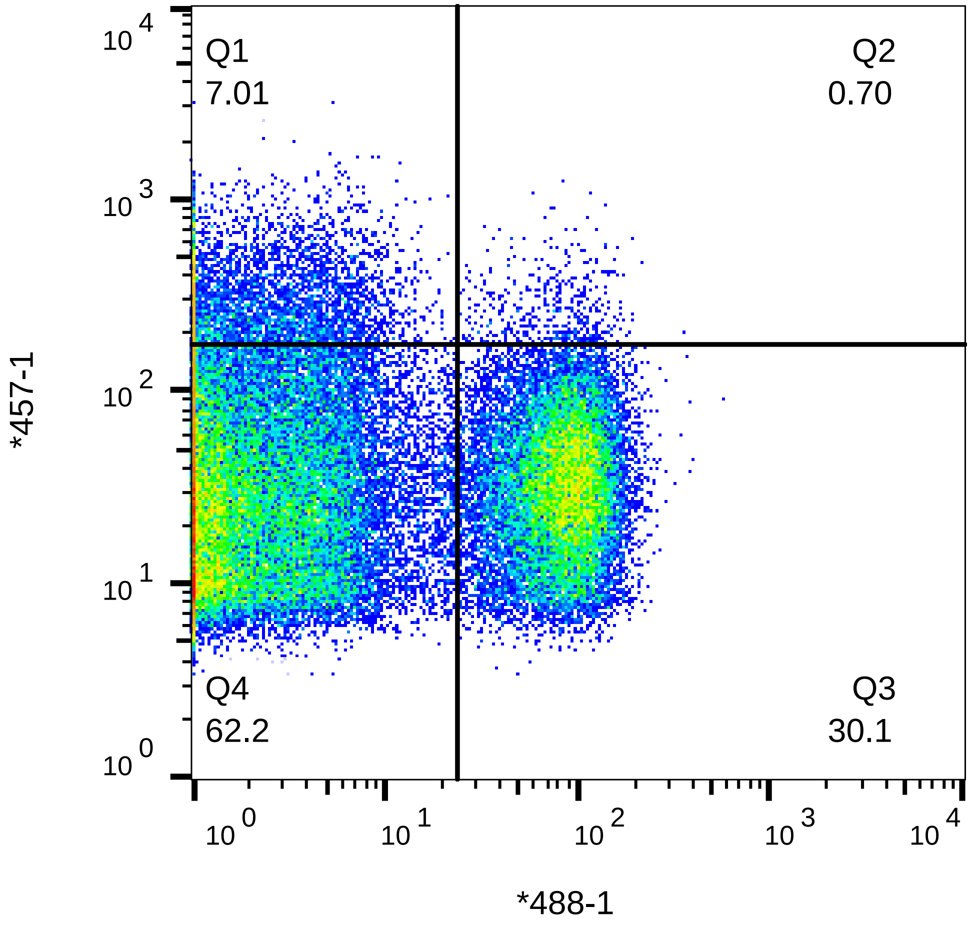

C:\Data\2020\Chrisler\20201007 Shank\SY329.fcs
pop
66193

ES2203; SY330; amyE::PsspB-Ypet (cam); lacA::PsdpA-mTurq (erm)

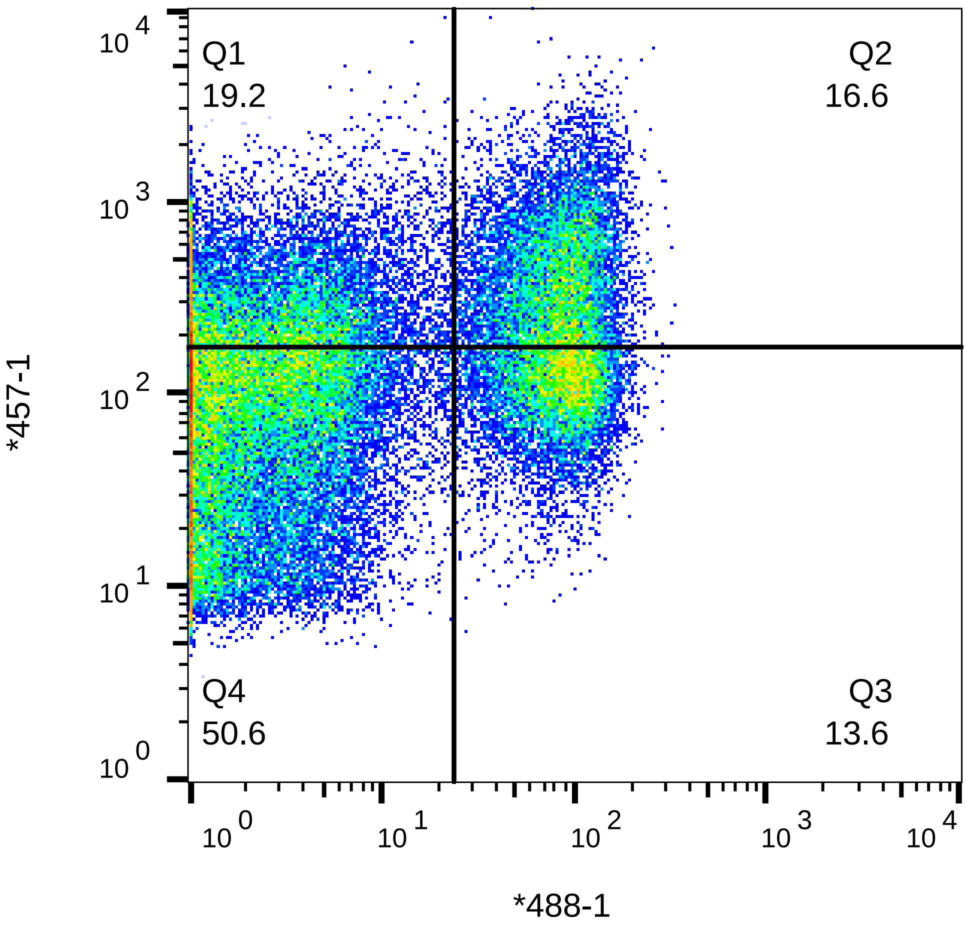

C:\Data\2020\Chrisler\20201007 Shank\SY330.fcs
pop
65196

ES2204; SY331; amyE::PsspB-Ypet (cam); lacA::PcomGA-mTurq (erm)

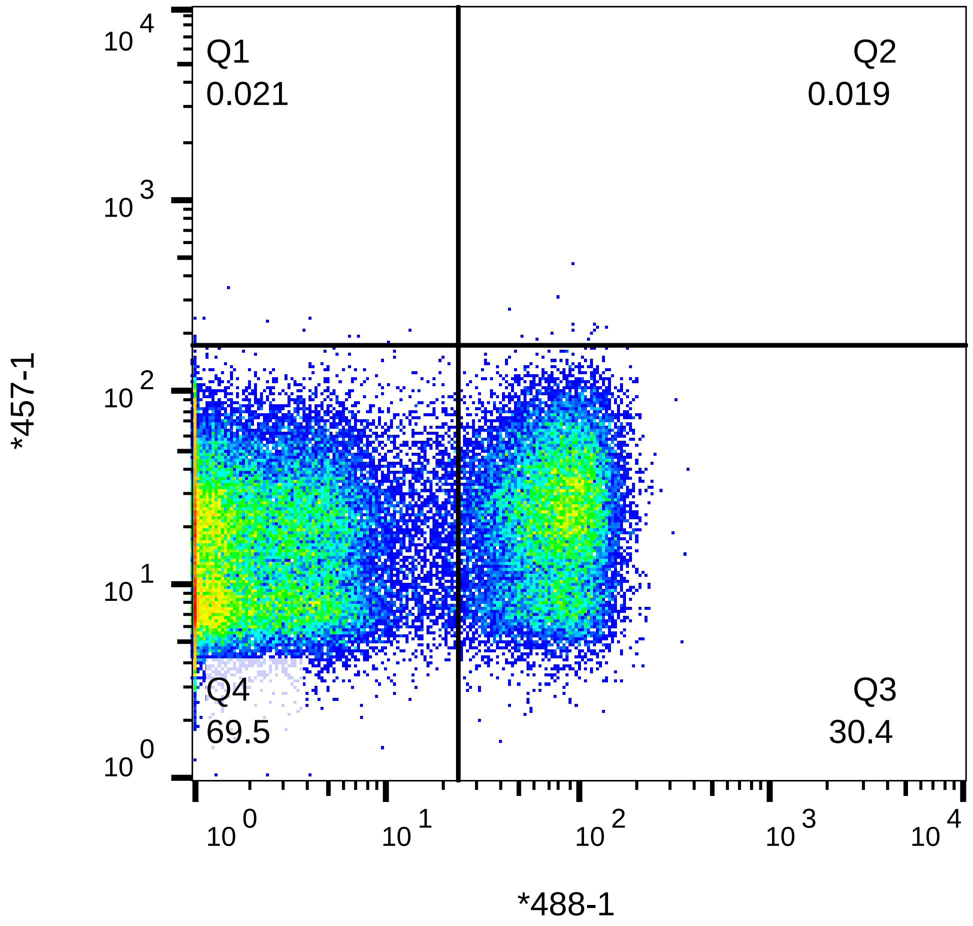

C:\Data\2020\Chrisler\20201007 Shank\SY331.fcs
pop
74773

ES2206; SY333; amyE::PsspB-Ypet (cam); lacA::PpksC-mTurq (erm)

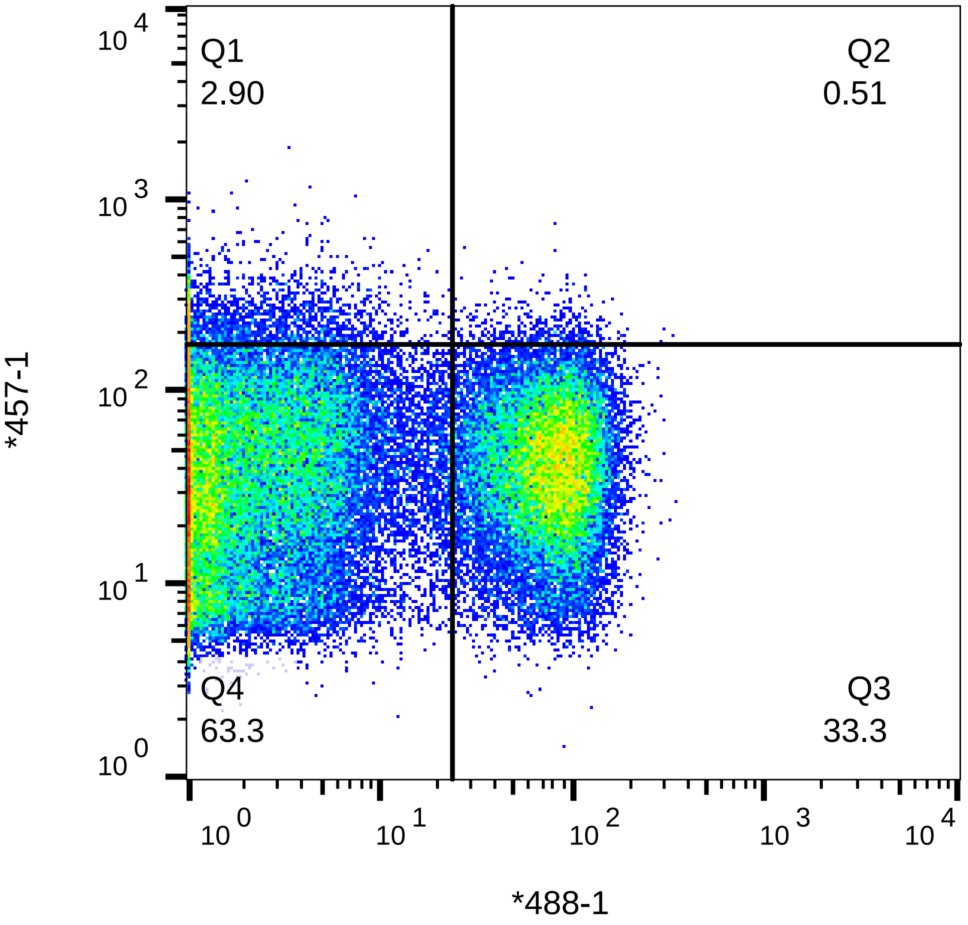

C:\Data\2020\Chrisler\20201007 Shank\SY333.fcs
pop
77869

ES2207; SY334; amyE::PsspB-Ypet (cam); lacA::PdhbA-mTurq (erm)

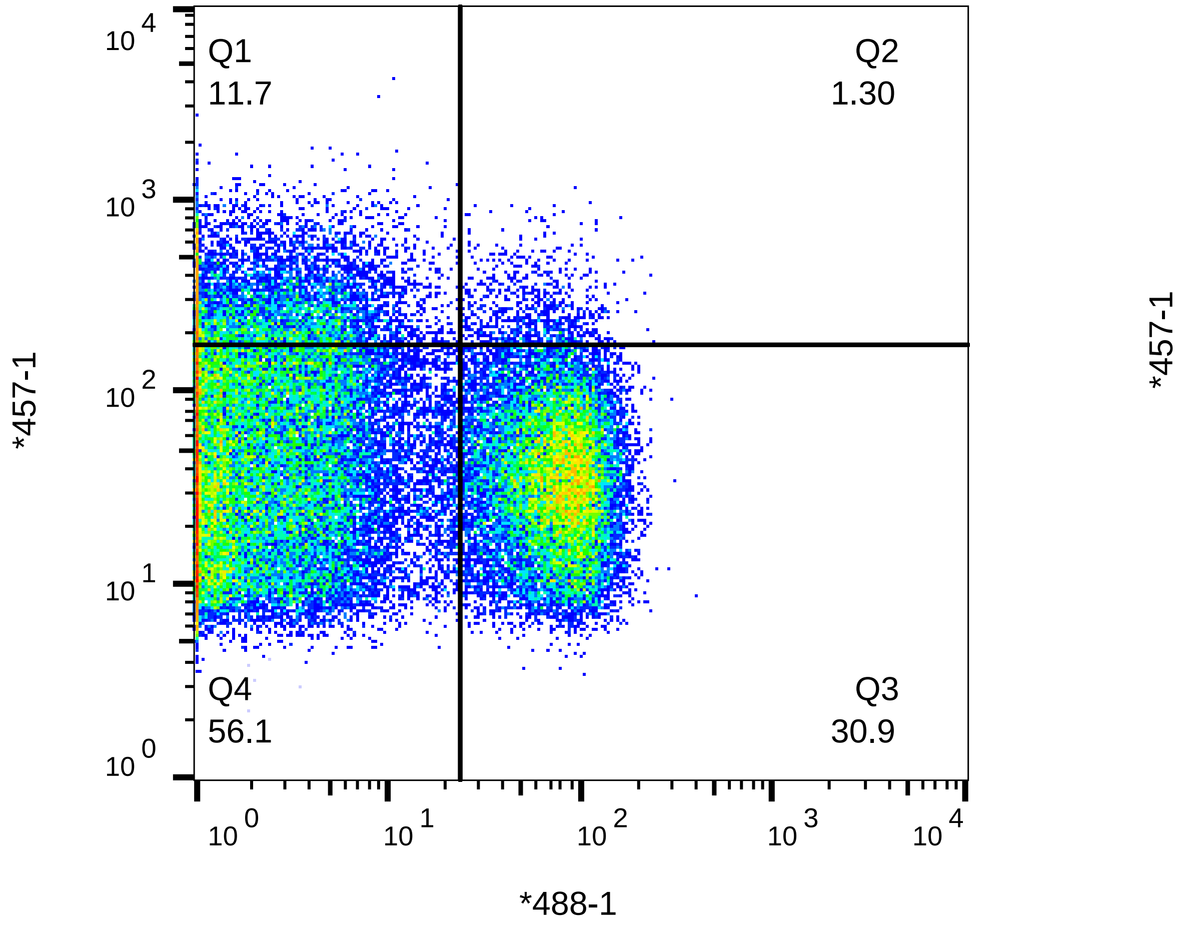

C:\Data\2020\Chrisler\20201007 Shank\SY334.fcs
pop
66504

ES2209; SY336; amyE::PsspB-Ypet (cam); lacA::PppsA-mTurq (erm)

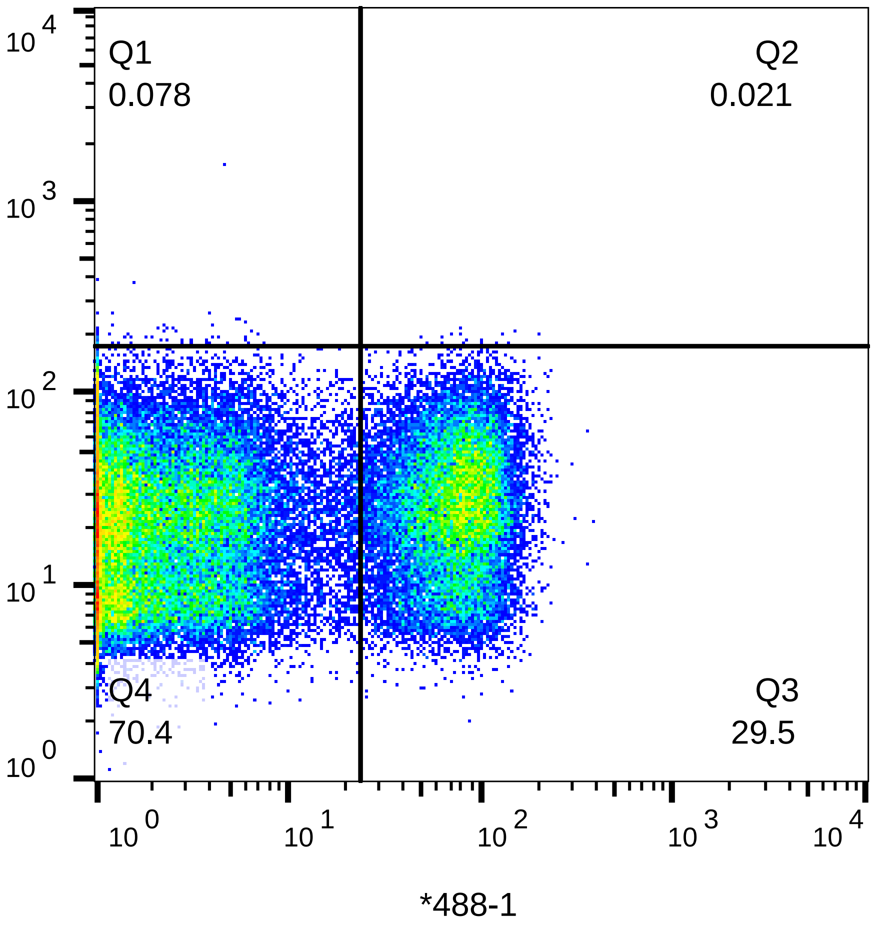

C:\Data\2020\Chrisler\20201007 Shank\SY336.fcs
pop
75378

ES2210; SY337; amyE::PsspB-Ypet (cam); lacA::PsrfAA-mTurq (erm)

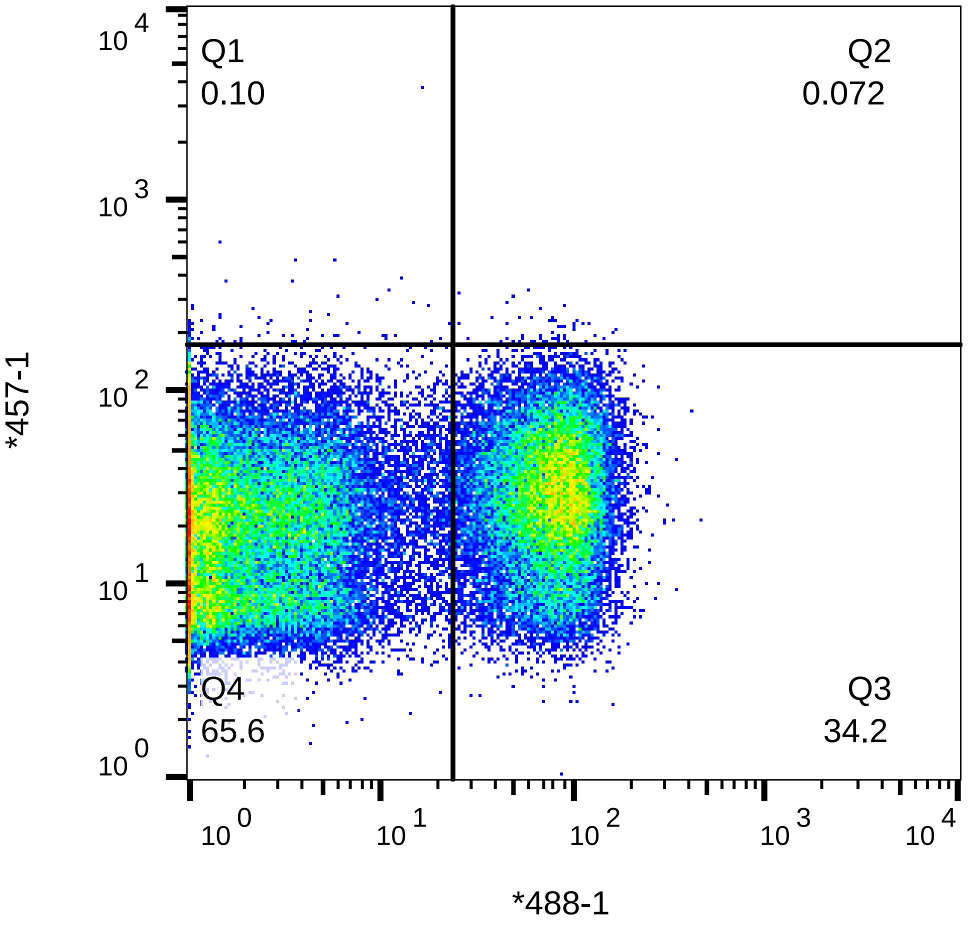

C:\Data\2020\Chrisler\20201007 Shank\SY337.fcs
pop
76793

ES2211; SY338; amyE::PsspB-Ypet (cam); lacA::PsboA-mTurq (erm)

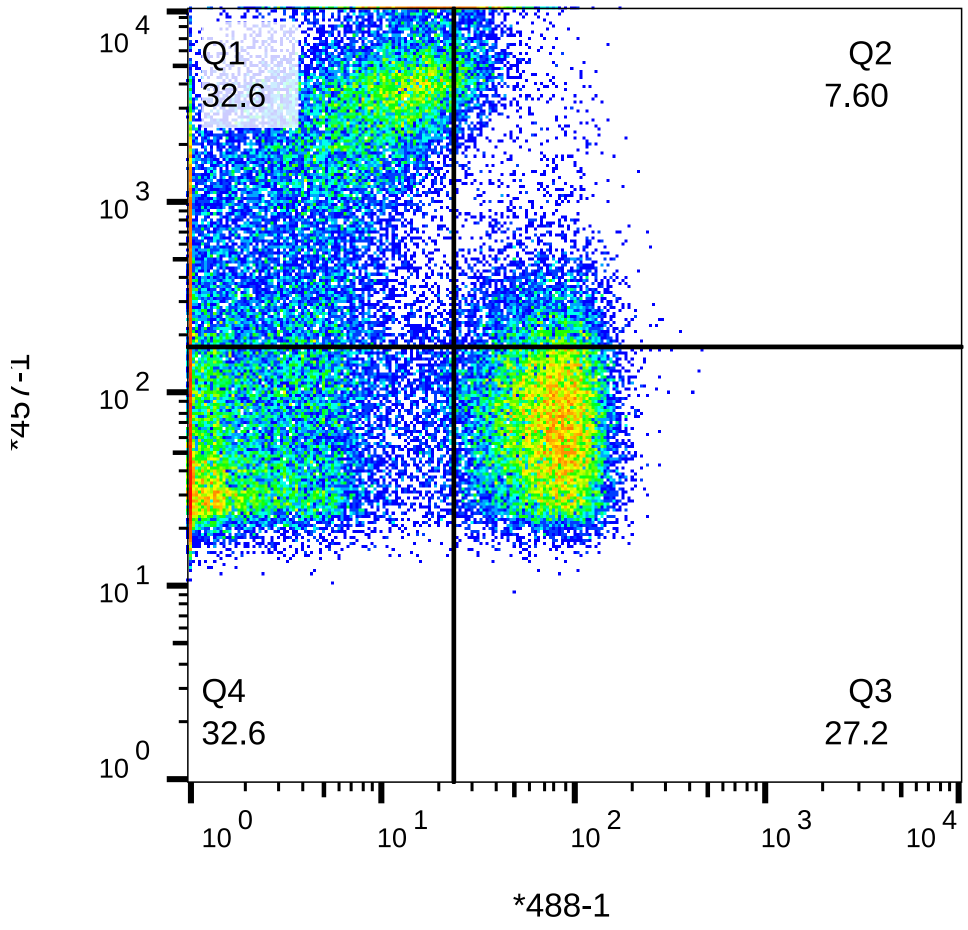

C:\Data\2020\Chrisler\20201007 Shank\SY338.fcs
pop
74903

ES2213; SY340; amyE::PsspB-Ypet (cam); lacA::PcomQX-mTurq (erm)

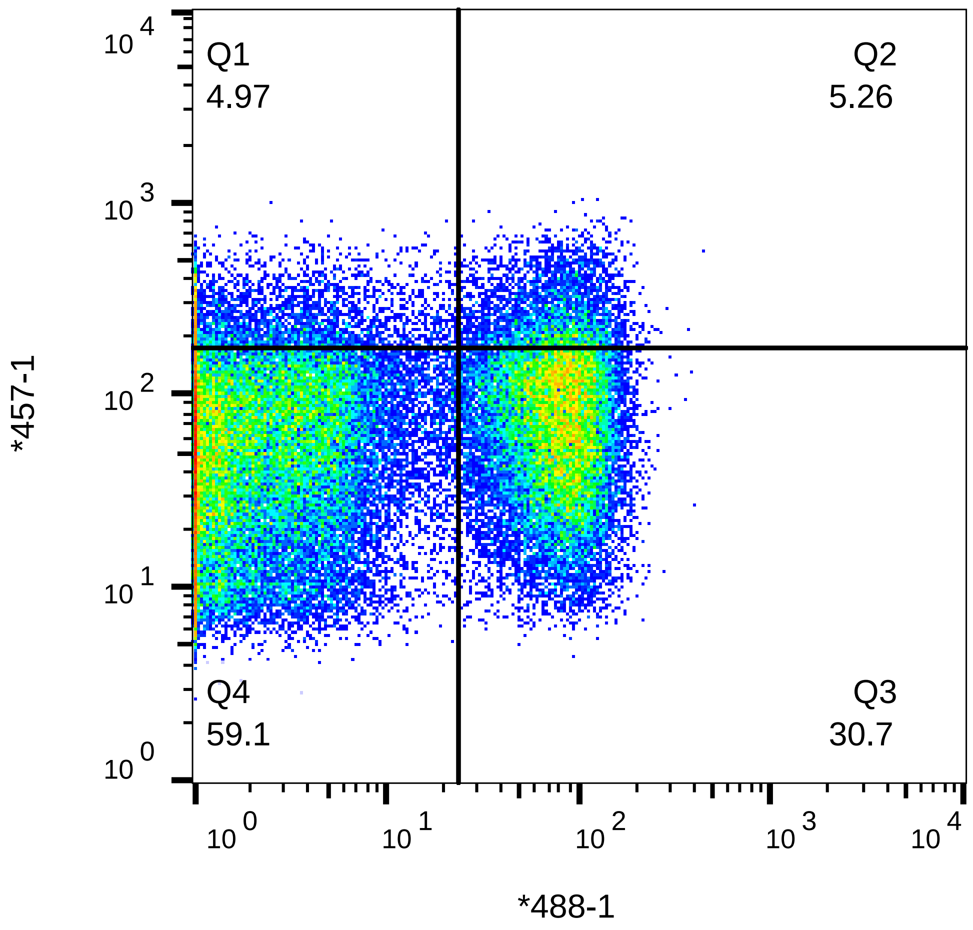

C:\Data\2020\Chrisler\20201007 Shank\SY340.fcs
pop
73284

ES2217; SY344; amyE::PsspB-Ypet (cam); lacA::PskfA-mTurq (erm)

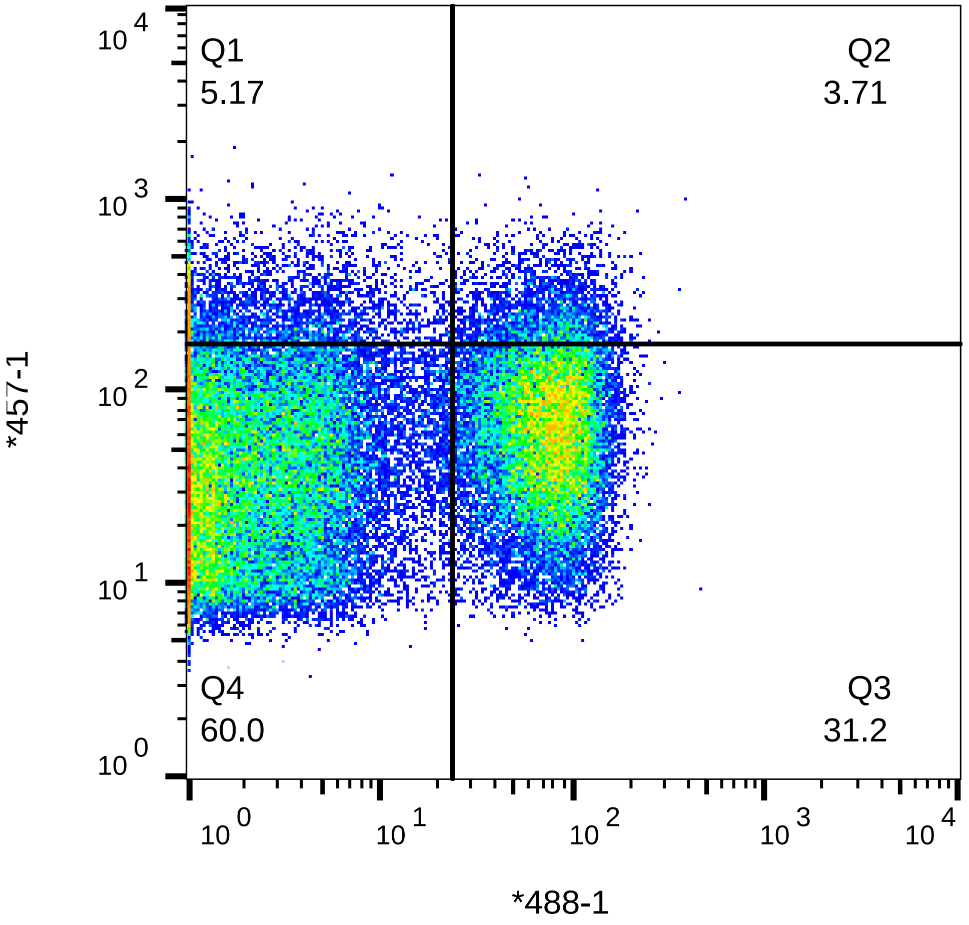

C:\Data\2020\Chrisler\20201007 Shank\SY344.fcs
pop
72012

ES2218; SY345; amyE::PsspB-Ypet (cam); lacA::PaprE-mTurq (erm)

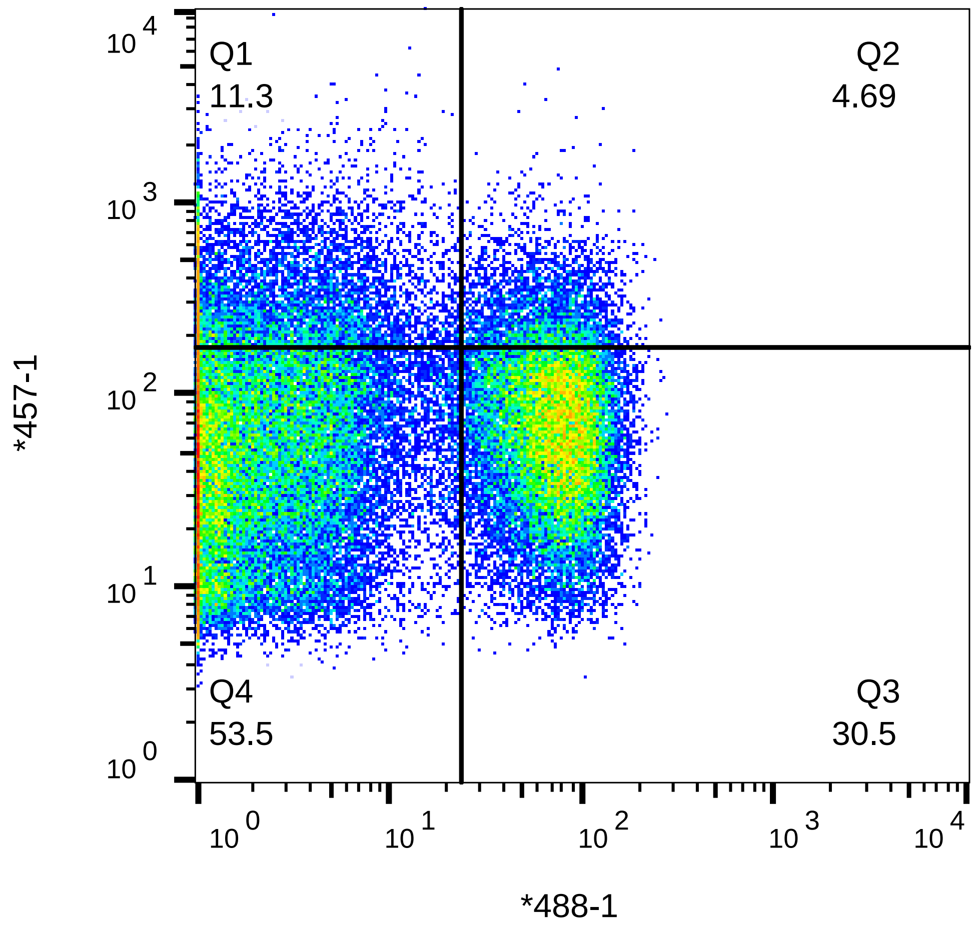

C:\Data\2020\Chrisler\20201007 Shank\SY345.fcs
pop
74323

ES2219; SY346; amyE::Phag-Ypet (cam); lacA::PsdpA-mTurq (erm)

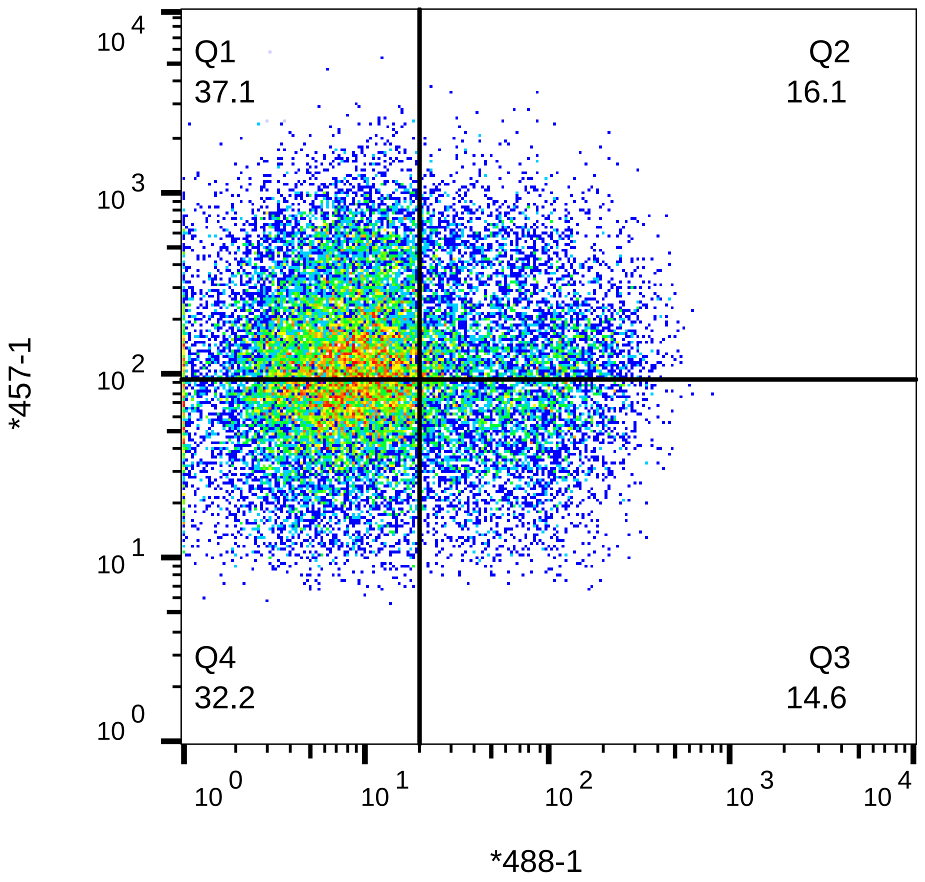

C:\Data\2019\Chrisler\20191219 Shank\76.fcs
All pop
26822

ES2220; SY347; amyE::Phag-Ypet (cam); lacA::PdhbA-mTurq (erm)

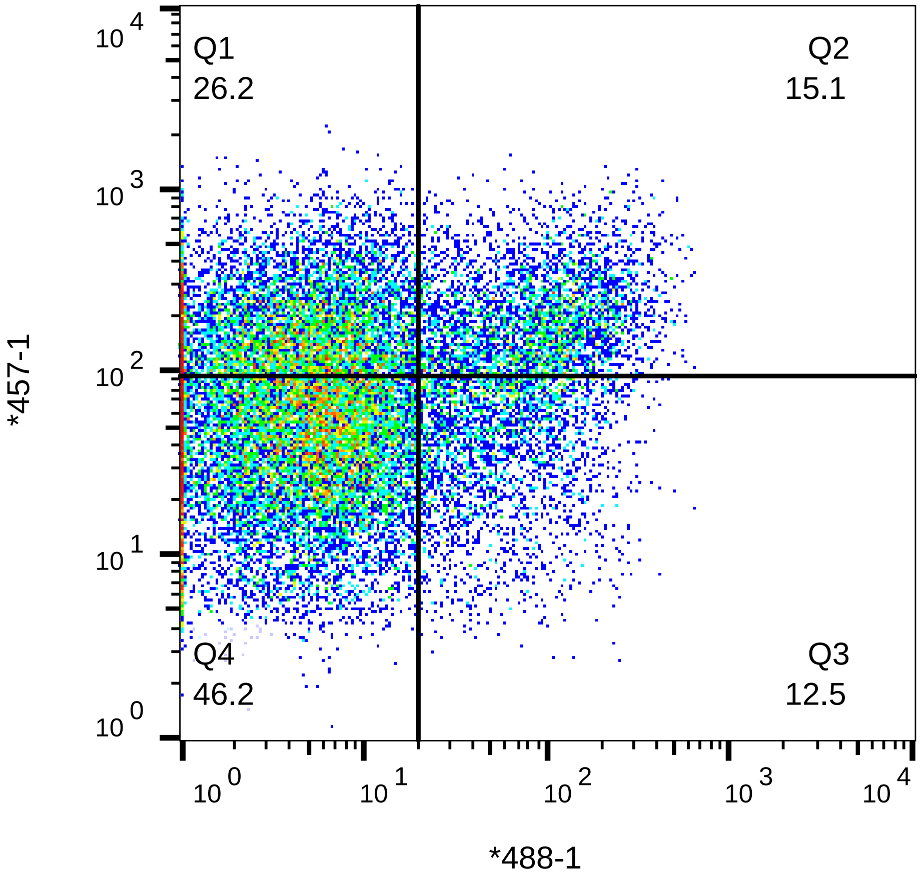

C:\Data\2019\Chrisler\20191219 Shank\77.fcs
All pop
25837

ES2221; SY348; amyE::Phag-Ypet (cam); lacA::PcomGA-mTurq (erm)

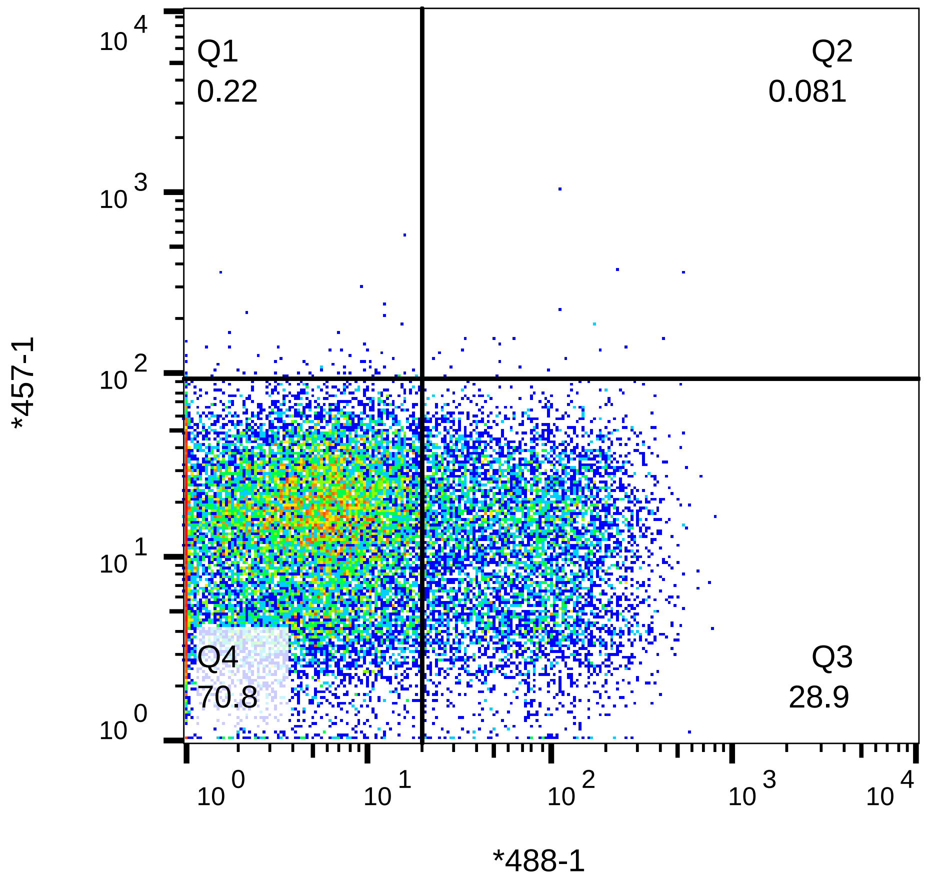

C:\Data\2019\Chrisler\20191219 Shank\78.fcs
All pop
25897

ES2223; SY350; amyE::Phag-Ypet (cam); lacA::PpksC-mTurq (erm)

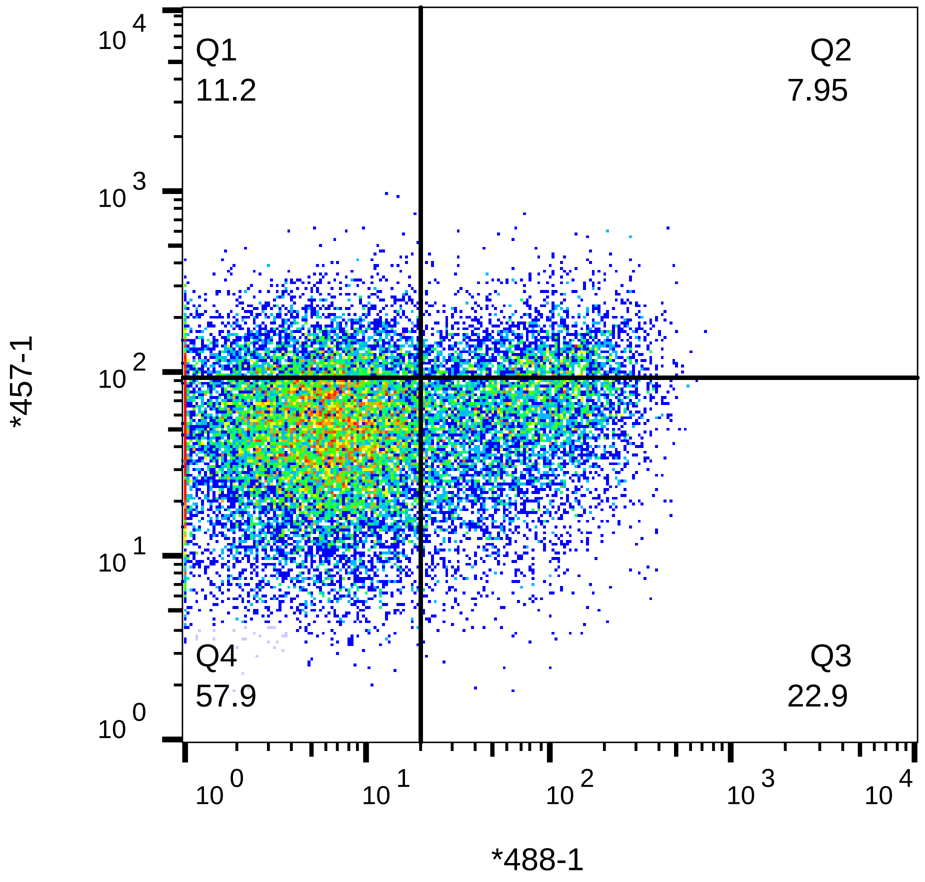

C:\Data\2019\Chrisler\20191219 Shank\80.fcs
All pop
25181

ES2224; SY351; amyE::Phag-Ypet (cam); lacA::PbacA-mTurq (erm)

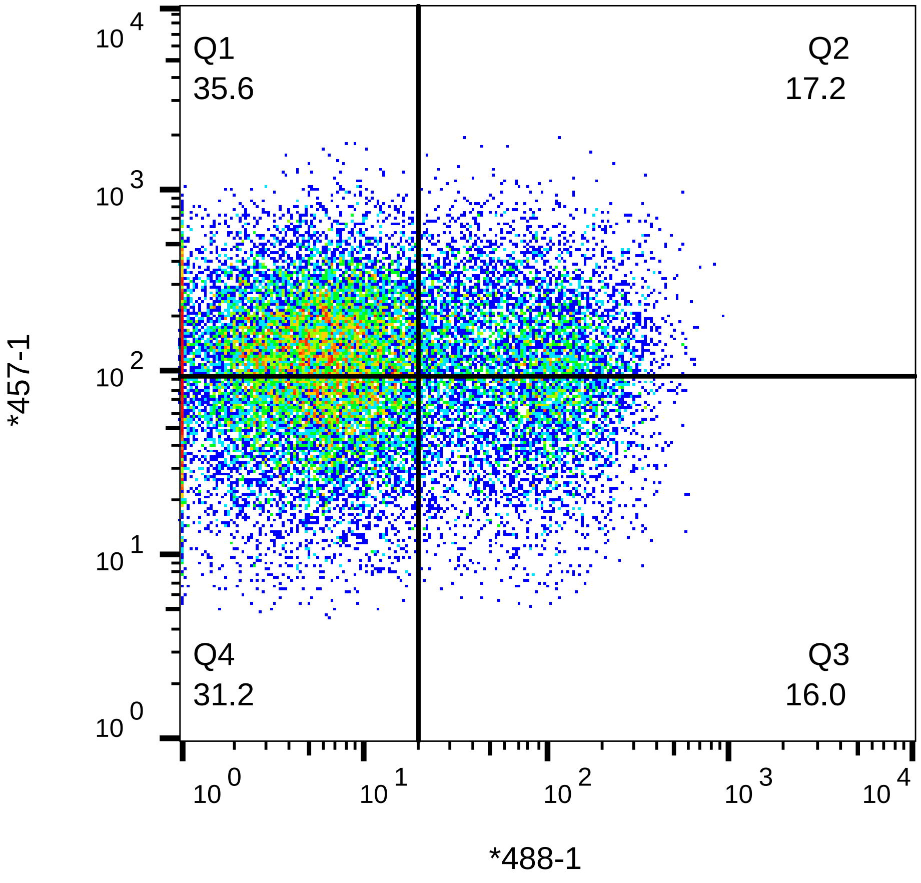

C:\Data\2019\Chrisler\20191219 Shank\81.fcs
All pop
24596

ES2225; SY352; amyE::Phag-Ypet (cam); lacA::PppsA-mTurq (erm)

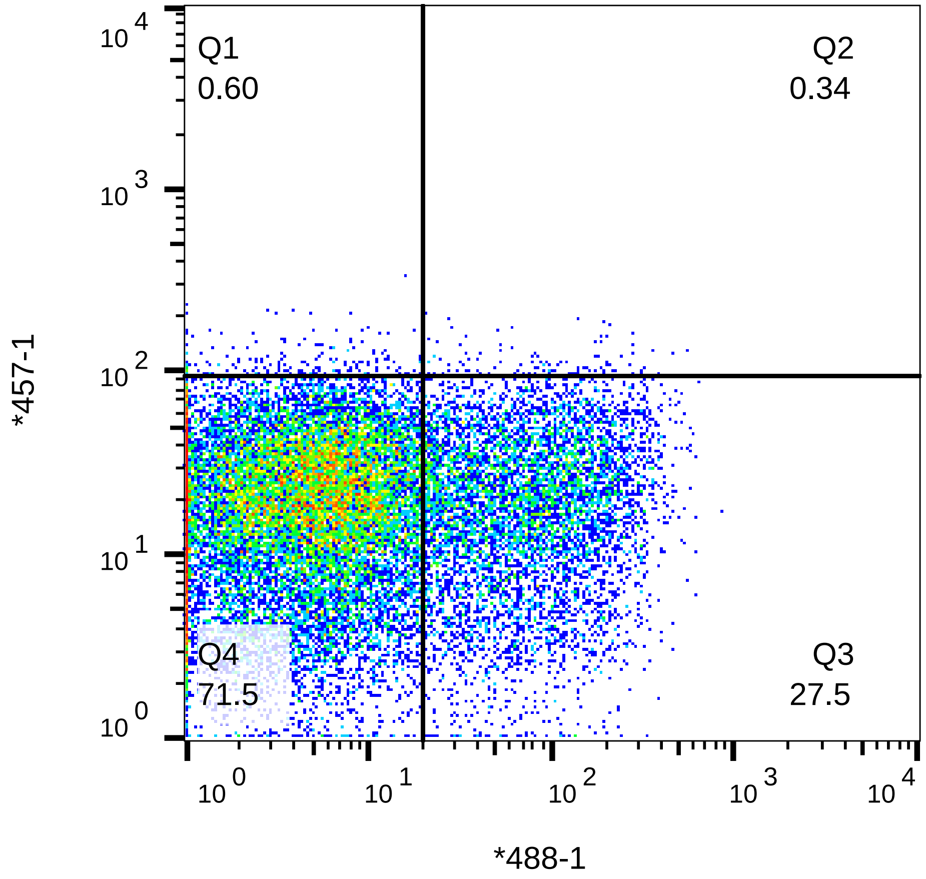

C:\Data\2019\Chrisler\20191219 Shank\82.fcs
All pop
25289

ES2226; SY353; amyE::Phag-Ypet (cam); lacA::PsrfAA-mTurq (erm)

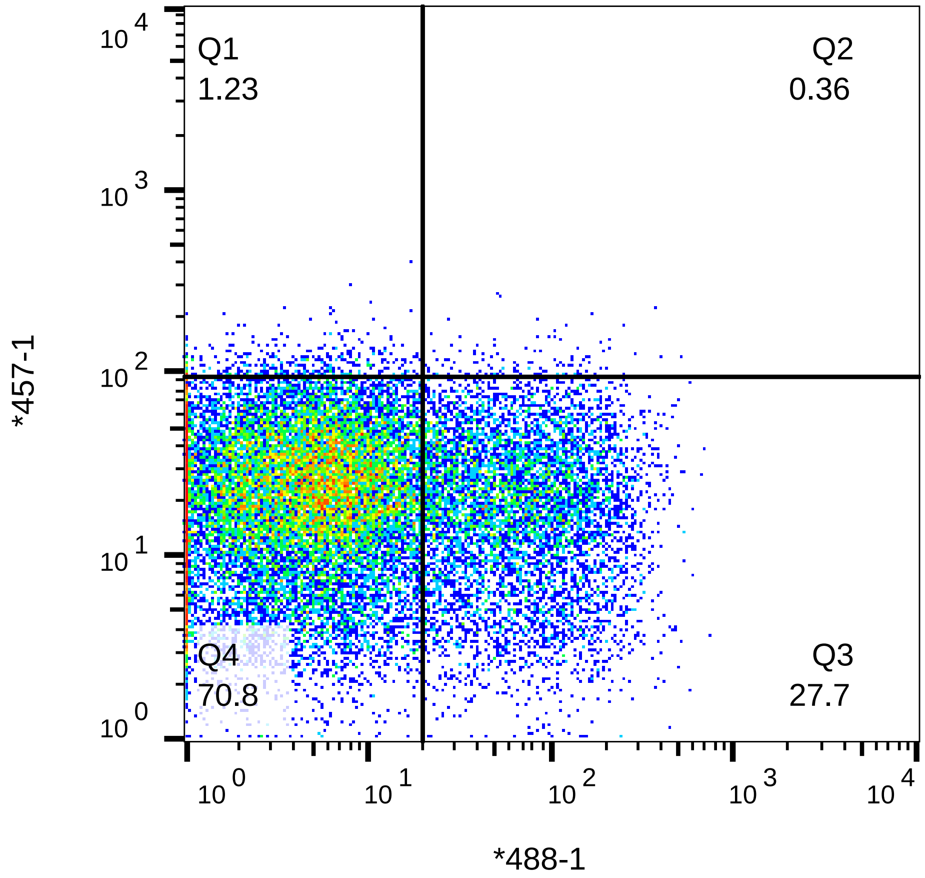

C:\Data\2019\Chrisler\20191219 Shank\83.fcs
All pop
25699

ES2227; SY354; amyE::Phag-Ypet (cam); lacA::PsboA-mTurq (erm)

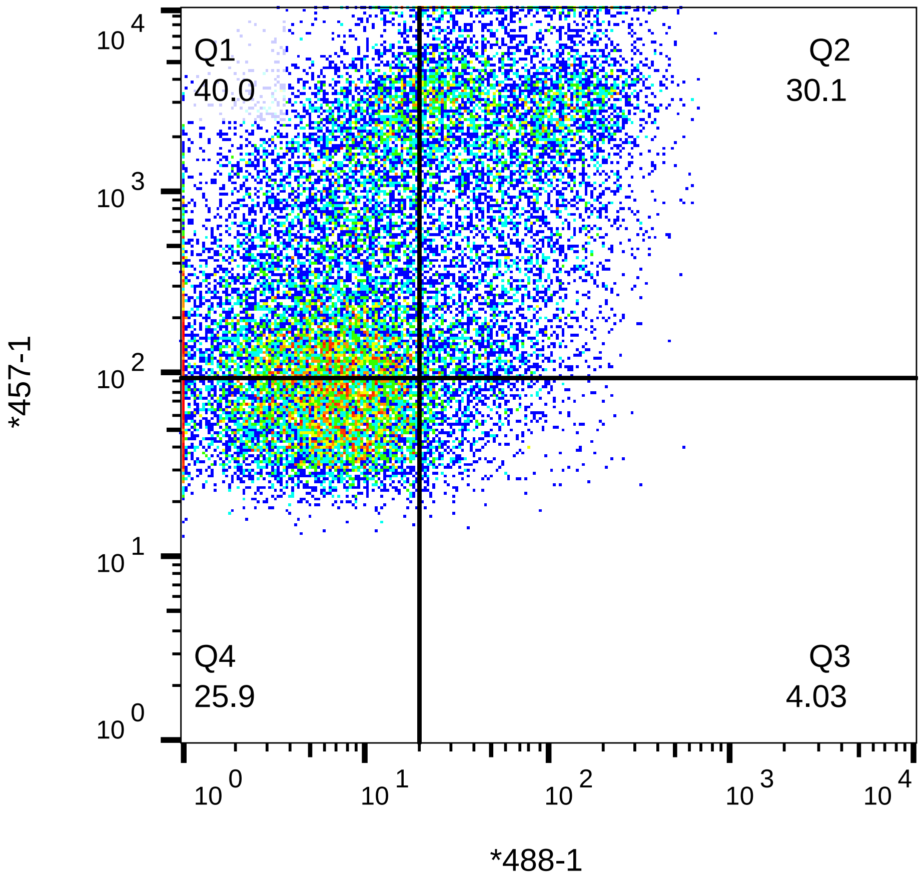

C:\Data\2019\Chrisler\20191219 Shank\84.fcs
All pop
26474

ES2229; SY356; amyE::Phag-Ypet (cam); lacA::PcomQX-mTurq (erm)

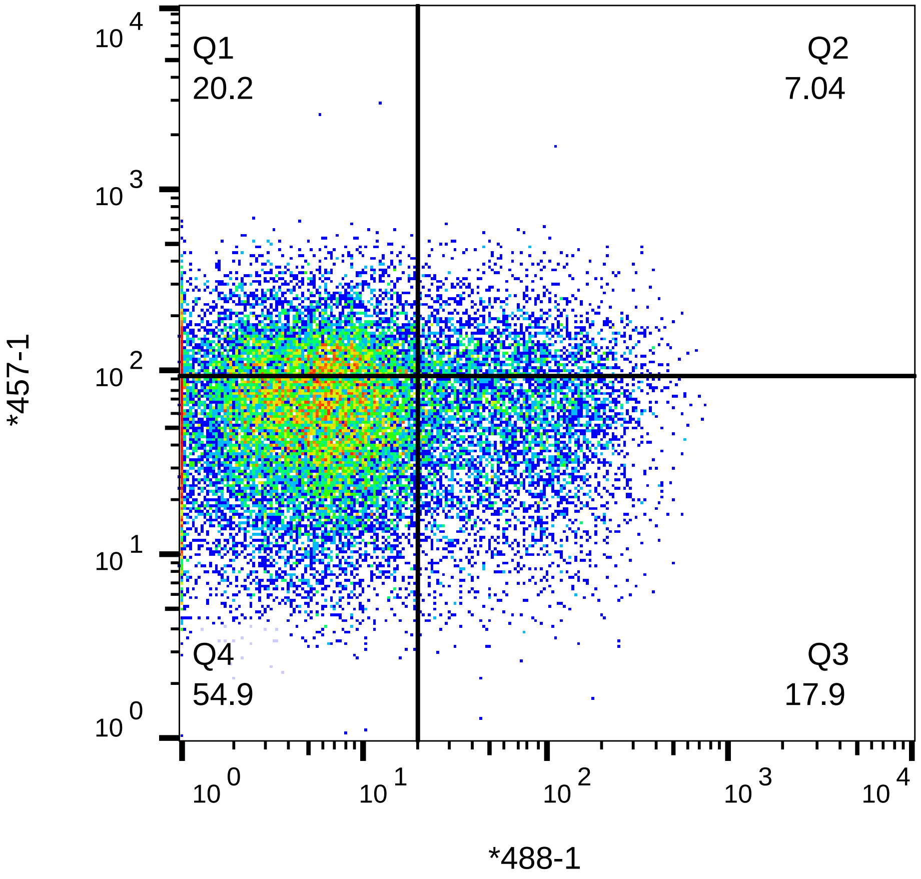

C:\Data\2019\Chrisler\20191219 Shank\86.fcs
All pop
26359

ES2233; SY360; amyE::Phag-Ypet (cam); lacA::PskfA-mTurq (erm)

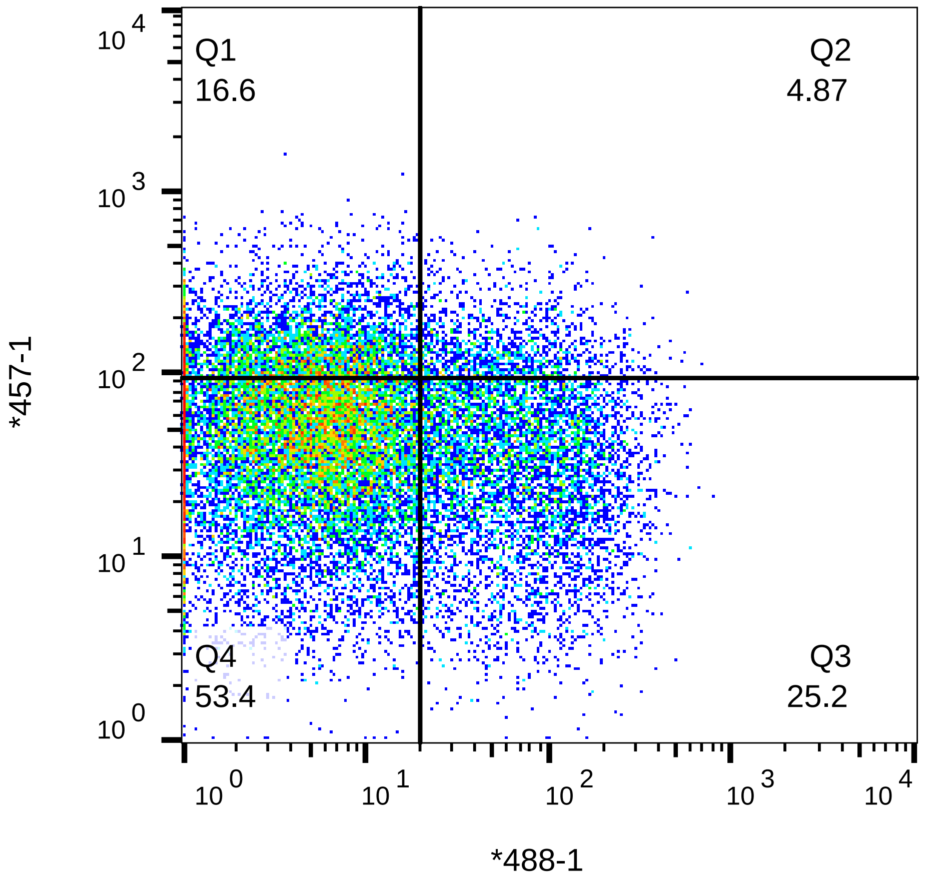

C:\Data\2019\Chrisler\20191219 Shank\90.fcs
All pop
25443

ES2234; SY361; amyE::Phag-Ypet (cam); lacA::PaprE-mTurq (erm)

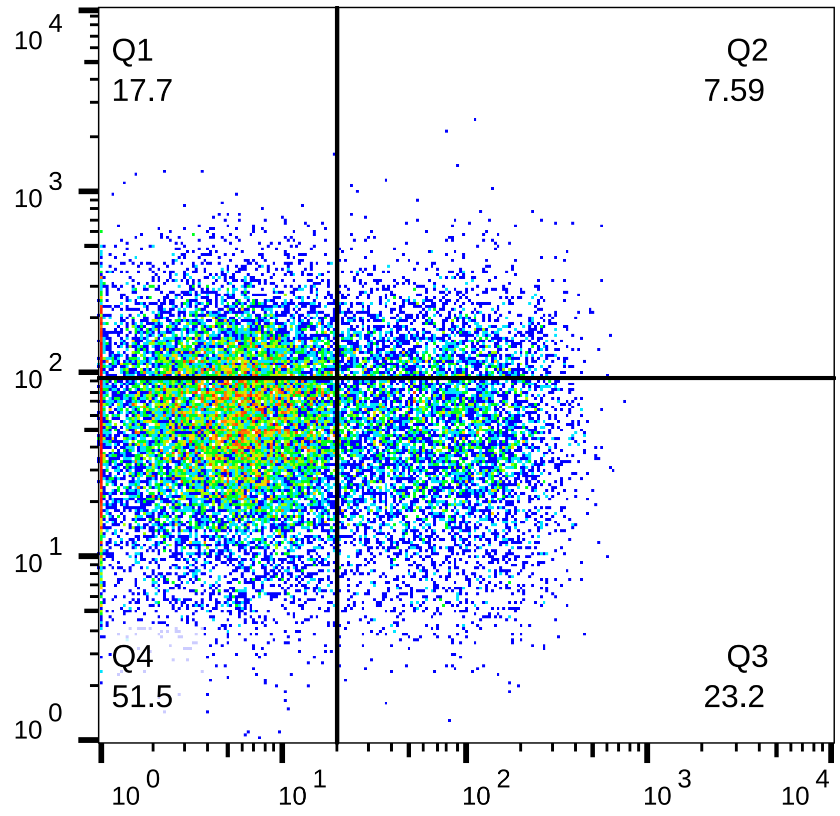

C:\Data\2019\Chrisler\20191219 Shank\91.fcs
All pop
25032

ES2235; SY362; amyE::PsdpA-Ypet (cam); lacA::PdhbA-mTurq (erm)

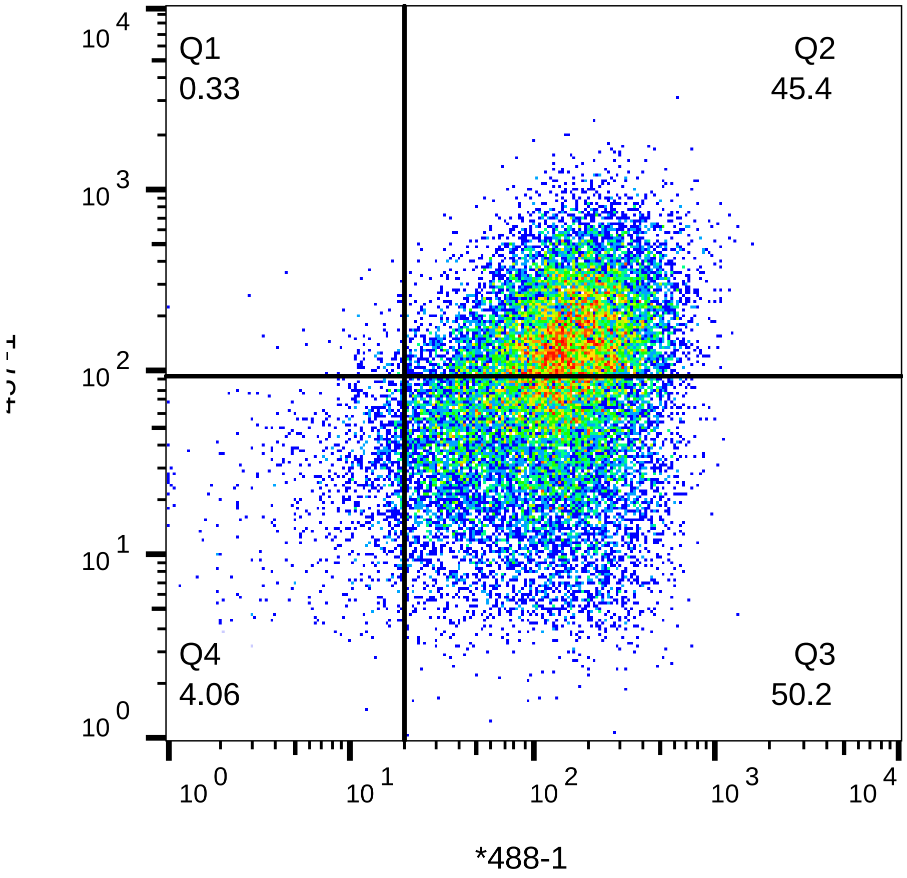

C:\Data\2019\Chrisler\20191219 Shank\92.fcs
All pop
24836

ES2236; SY363; amyE::PsdpA-Ypet (cam); lacA::PcomGA-mTurq (erm)

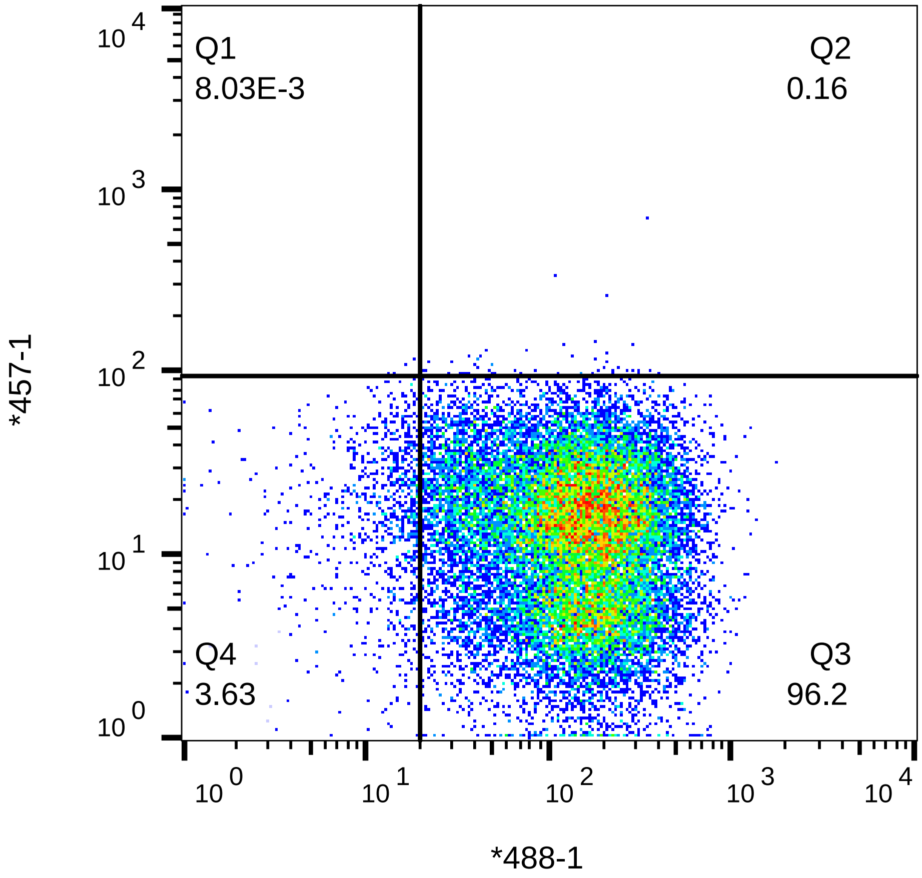

C:\Data\2019\Chrisler\20191219 Shank\93.fcs
All pop
24898

ES2238; SY365; amyE::PsdpA-Ypet (cam); lacA::PpksC-mTurq (erm)

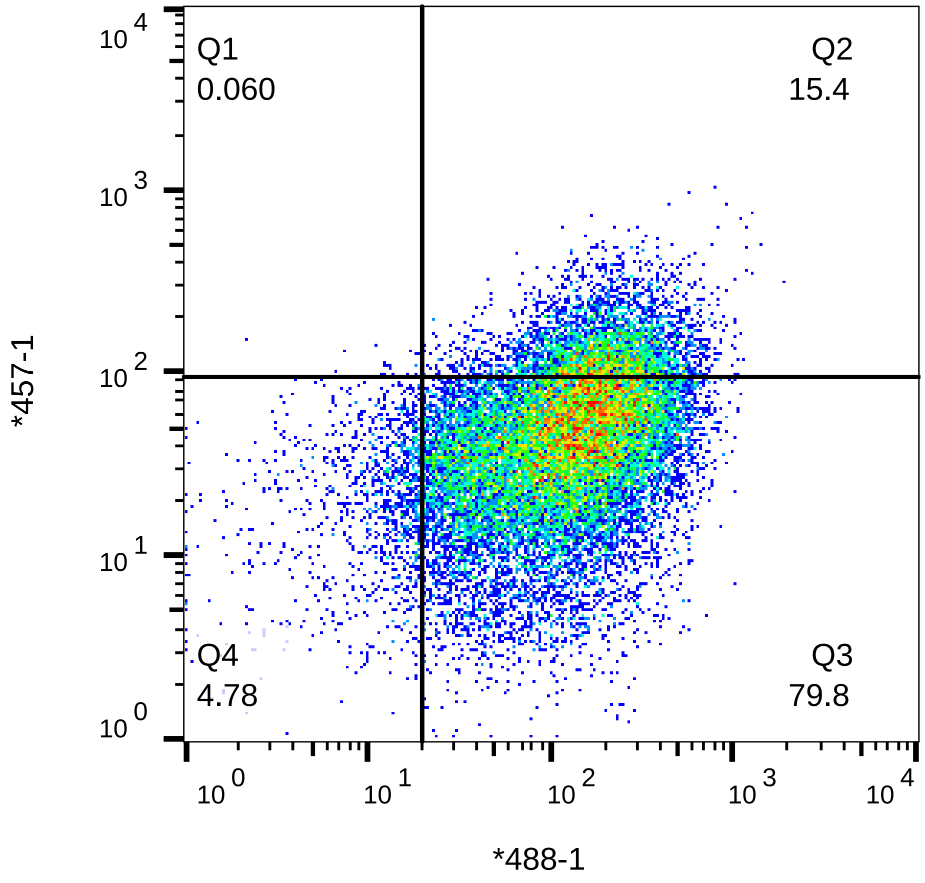

C:\Data\2019\Chrisler\20191219 Shank\95.fcs
All pop
25032

ES2239; SY366; amyE::PsdpA-Ypet (cam); lacA::PbacA-mTurq (erm)

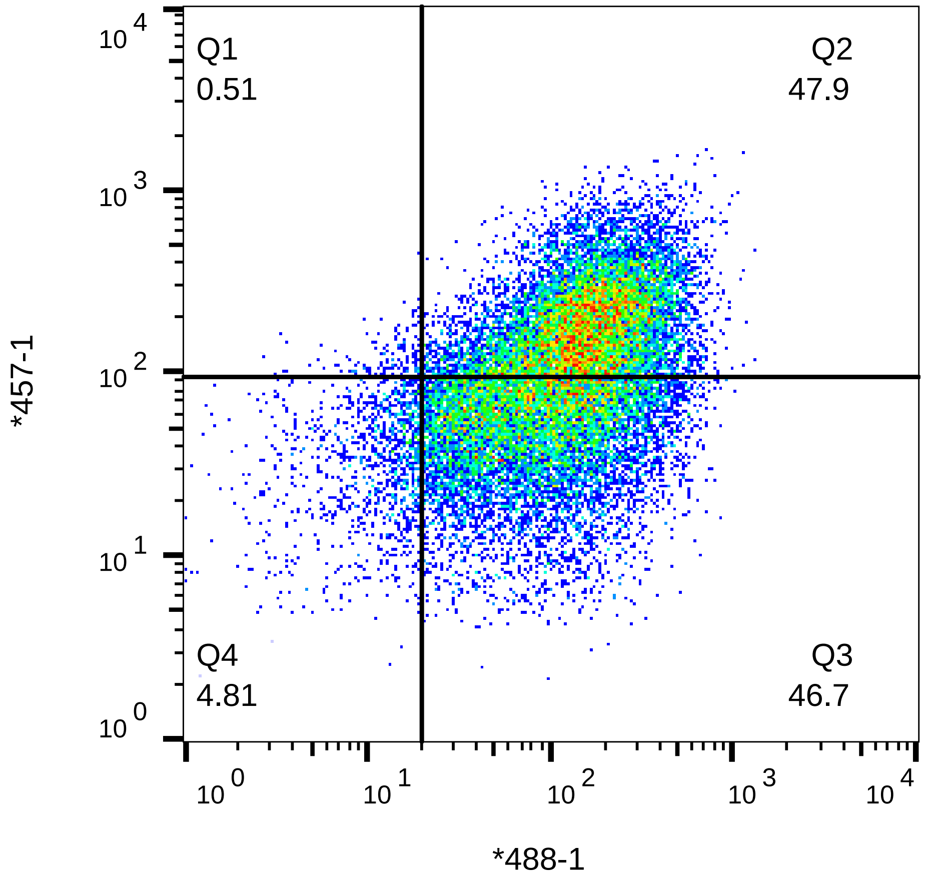

C:\Data\2019\Chrisler\20191219 Shank\96.fcs
All pop
25384

ES2240; SY367; amyE::PsdpA-Ypet (cam); lacA::PppsA-mTurq (erm)

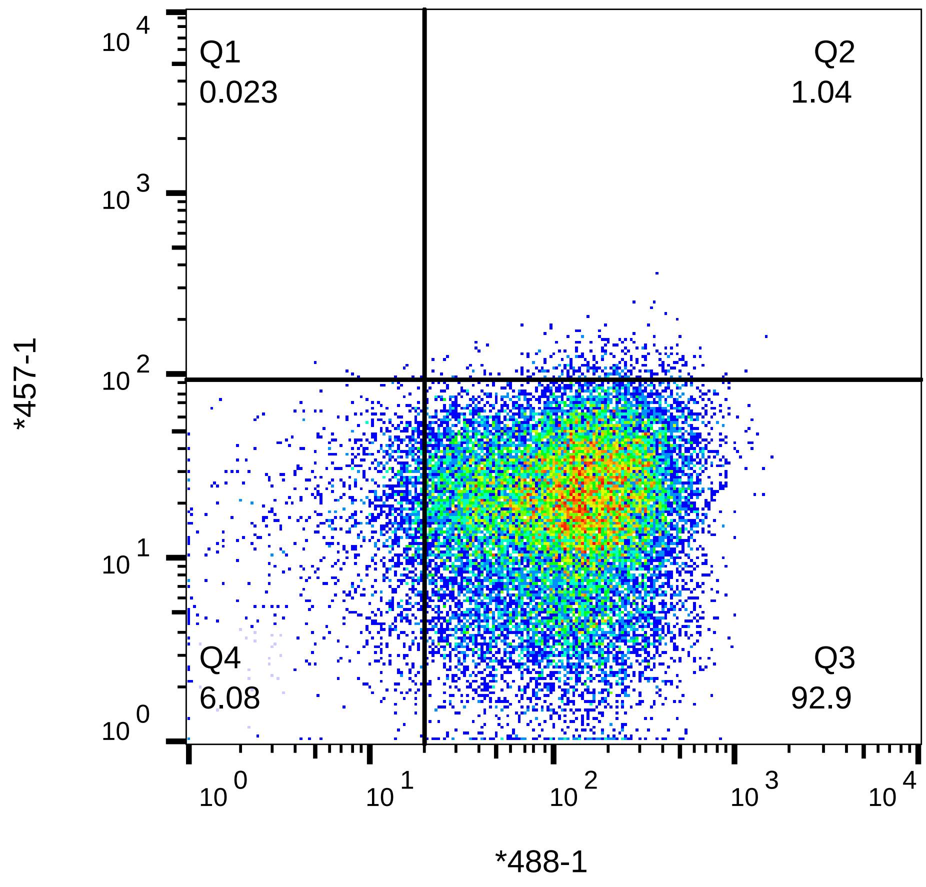

C:\Data\2019\Chrisler\20191219 Shank\97.fcs
All pop
25874

ES2241; SY368; amyE::PsdpA-Ypet (cam); lacA::PsrfAA-mTurq (erm)

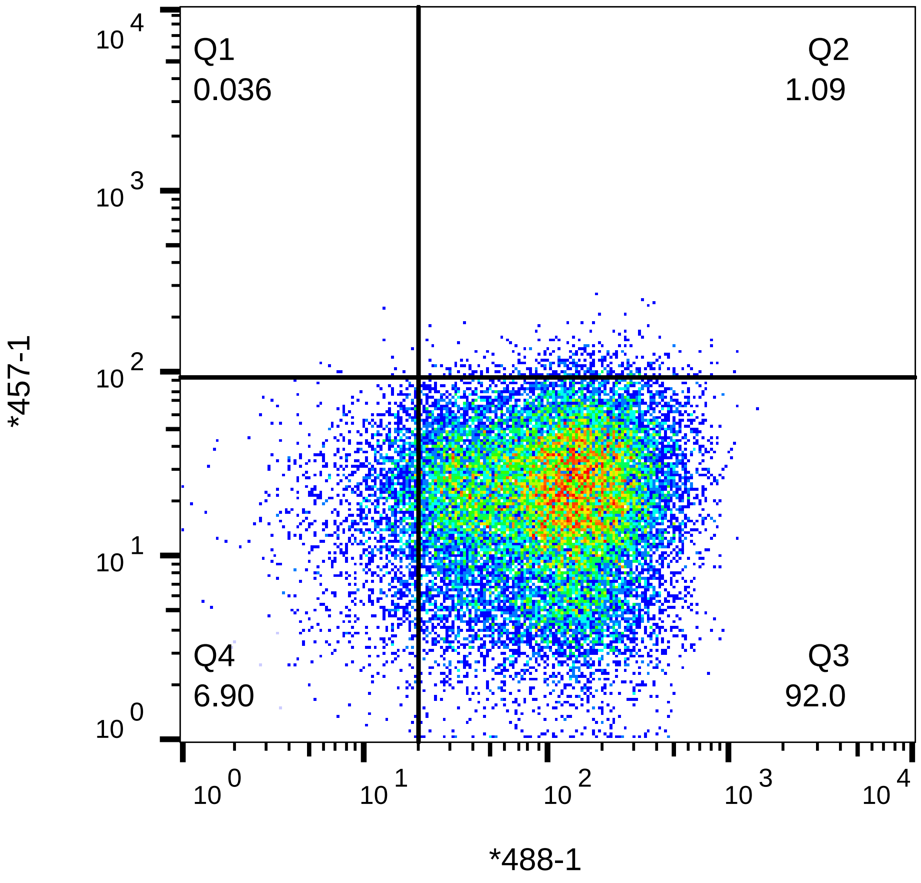

C:\Data\2019\Chrisler\20191219 Shank\98.fcs
All pop
27459

ES2242; SY369; amyE::PsdpA-Ypet (cam); lacA::PsboA-mTurq (erm)

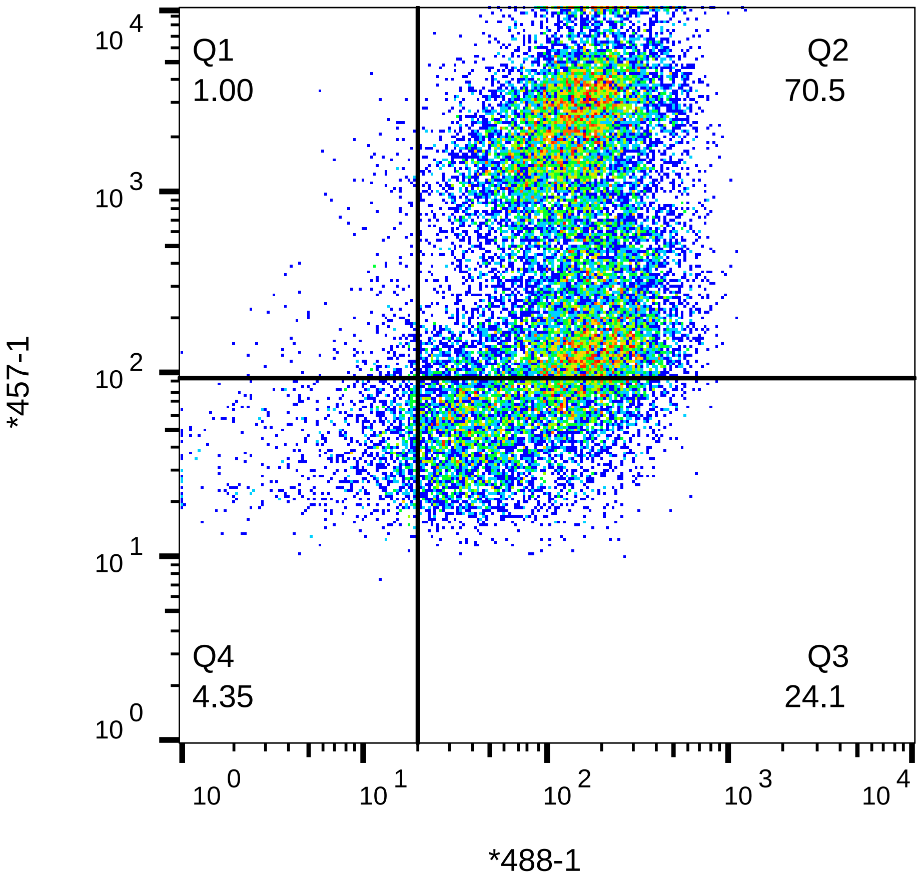

C:\Data\2019\Chrisler\20191219 Shank\99.fcs
All pop
24895

ES2244; SY371; amyE::PsdpA-Ypet (cam) ; lacA::PcomQX-mTurq (erm)

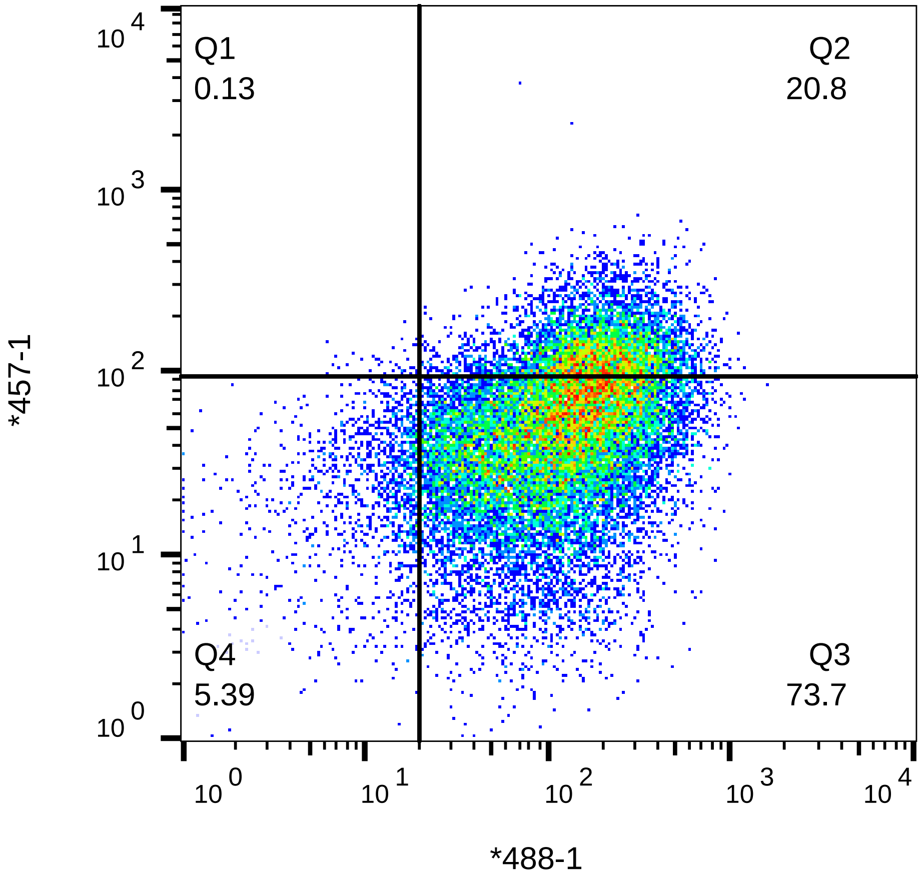

C:\Data\2019\Chrisler\20191219 Shank\101.fcs

All pop

24594

ES2248; SY375; amyE::PsdpA-Ypet (cam); lacA::PskfA-mTurq (erm)

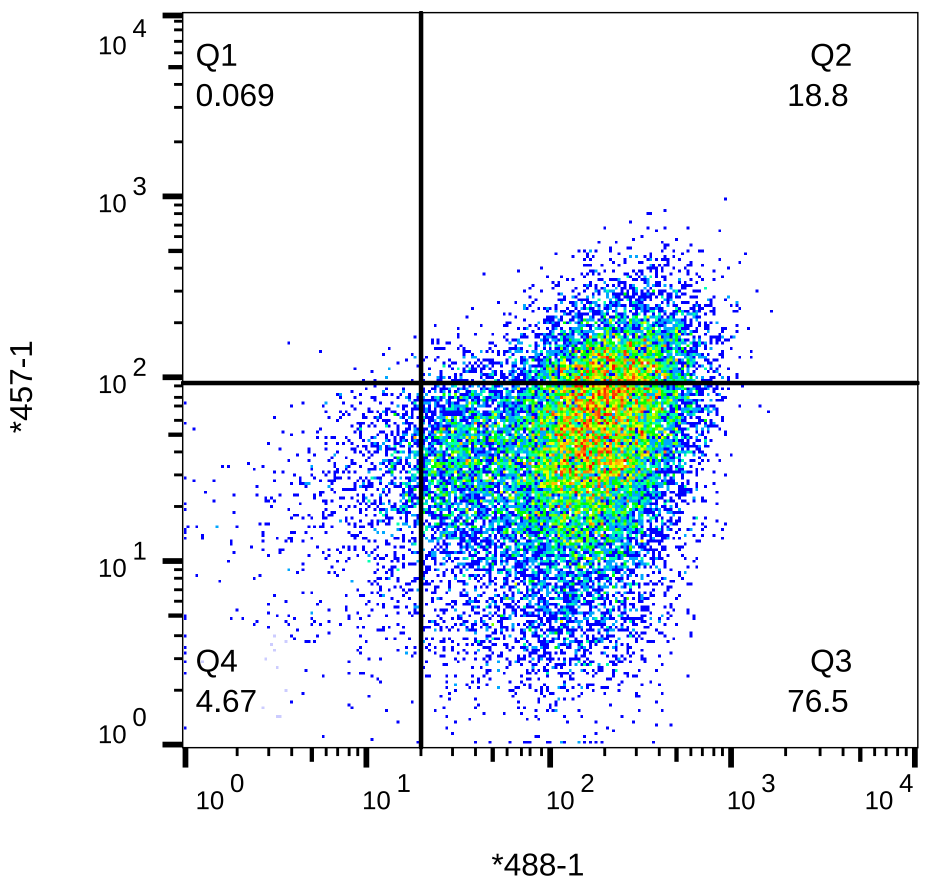

C:\Data\2019\Chrisler\20191219 Shank\105.fcs
All pop
24493

ES2249; SY376; amyE::PsdpA-Ypet (cam); lacA::PaprE-mTurq (erm)

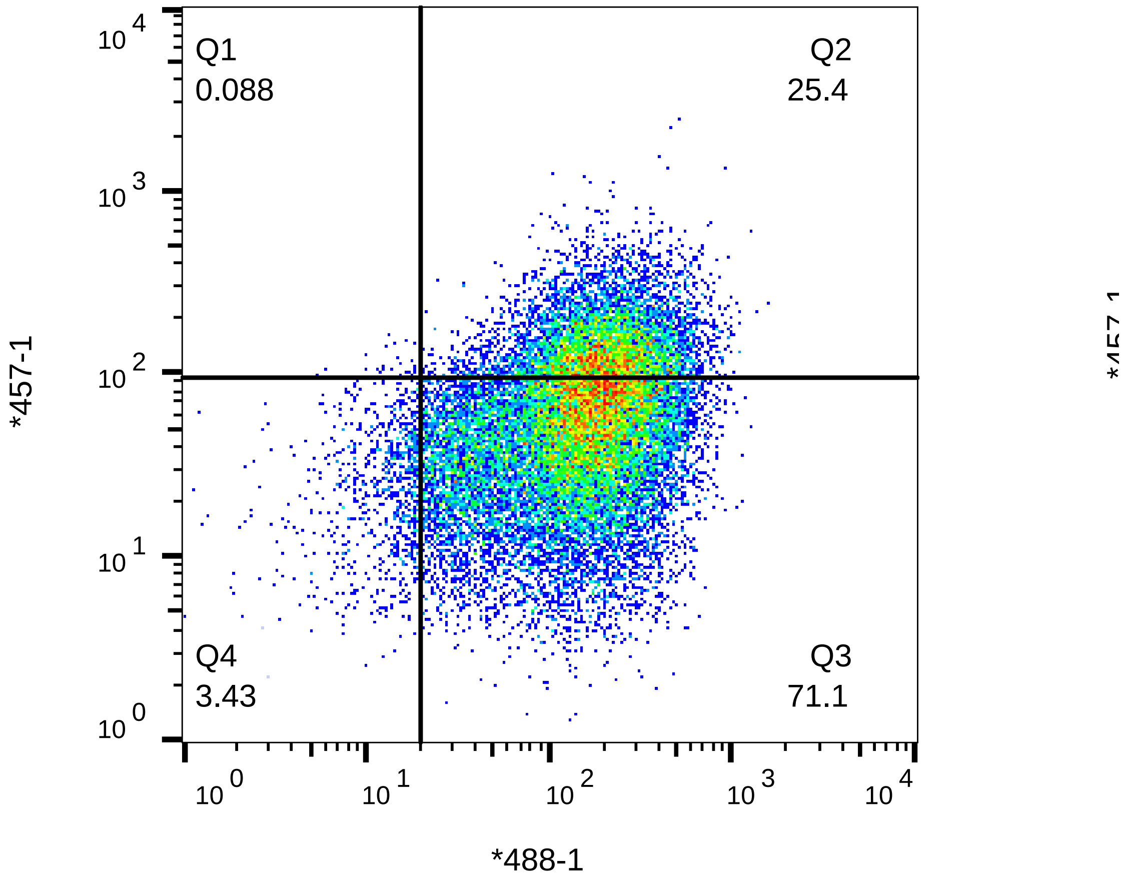

C:\Data\2019\Chrisler\20191219 Shank\106.fcs
All pop
25074

ES2251; SY378; amyE::PcomGA-Ypet (cam); lacA::PpksC-mTurq (erm)

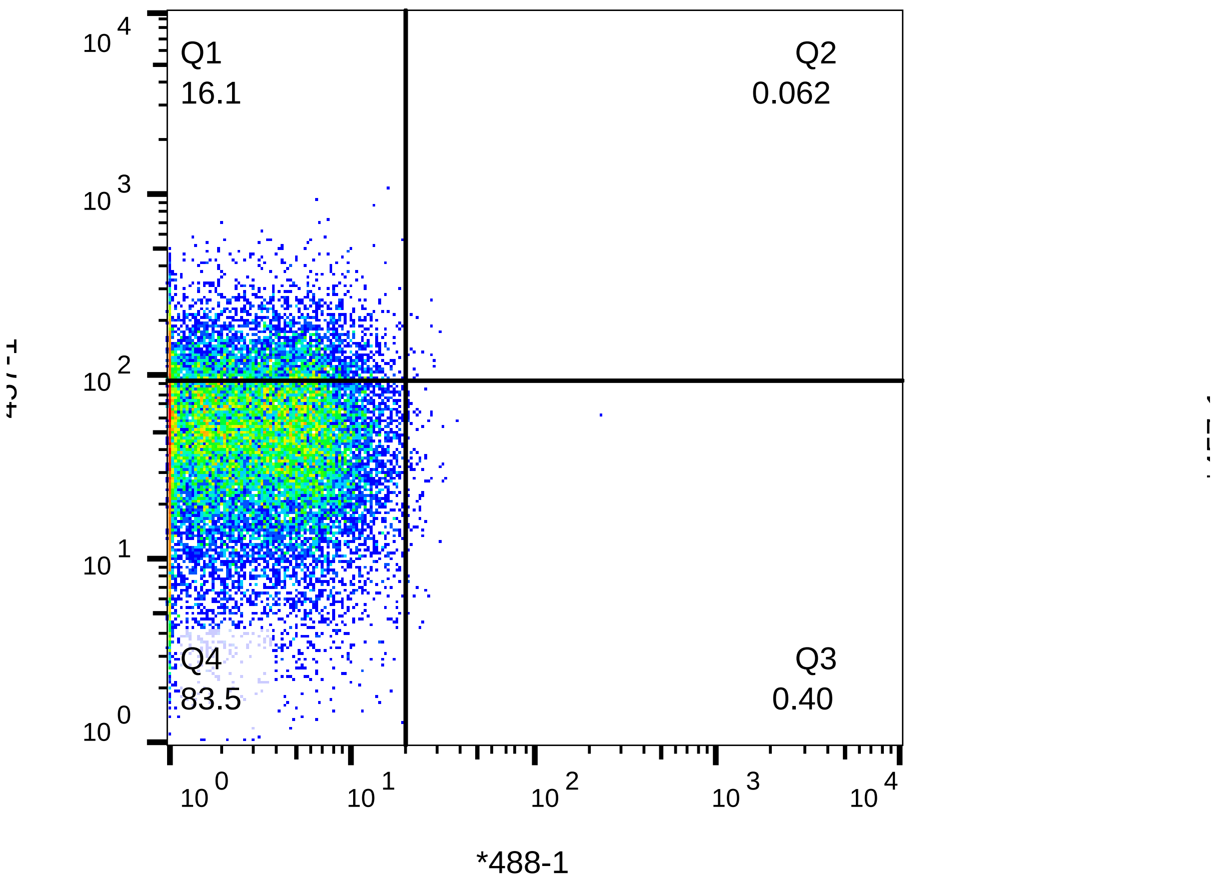

C:\Data\2019\Chrisler\20191219 Shank\108.fcs
All pop
25854

ES2252; SY379; amyE::PcomGA-Ypet (cam); lacA::PdhbA-mTurq (erm)

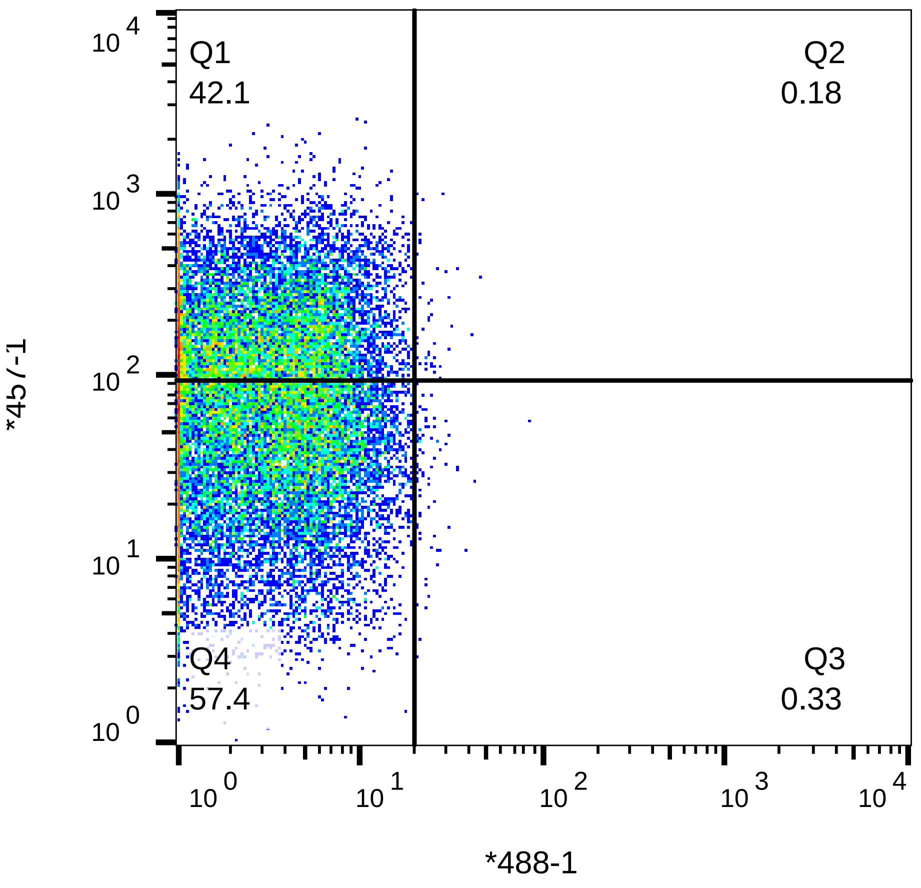

C:\Data\2019\Chrisler\20191219 Shank\109.fcs
All pop
26091

ES2253; SY380; amyE::PcomGA-Ypet (cam); lacA::PbacA-mTurq (erm)

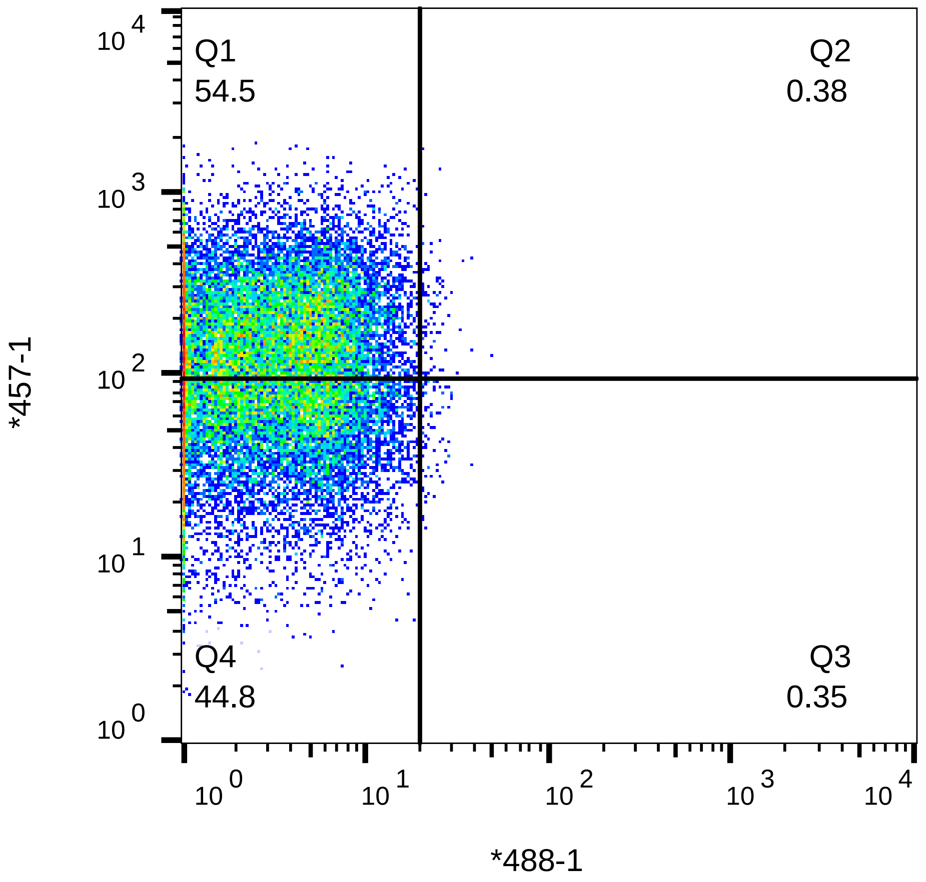

C:\Data\2019\Chrisler\20191219 Shank\110.fcs
All pop
26234

ES2254; SY381; amyE::PcomGA-Ypet (cam); lacA::PppsA-mTurq (erm)

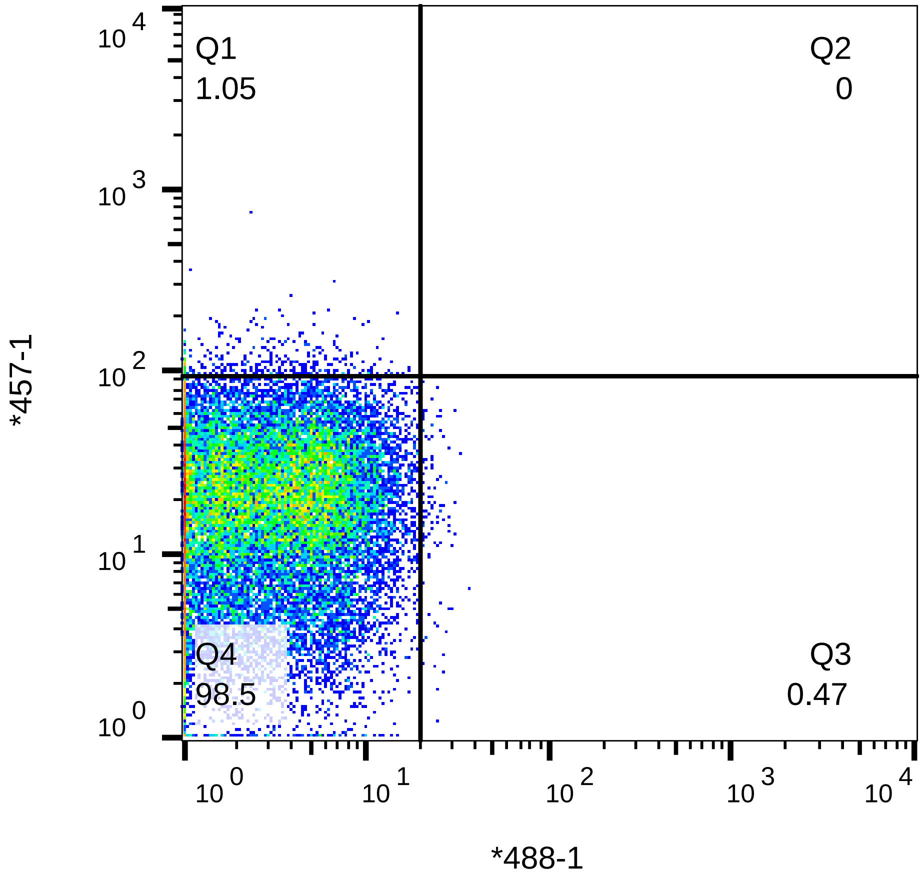

C:\Data\2019\Chrisler\20191219 Shank\111.fcs
All pop
26028

ES2255; SY382; amyE::PcomGA-Ypet (cam); lacA::PsrfAA-mTurq (erm)

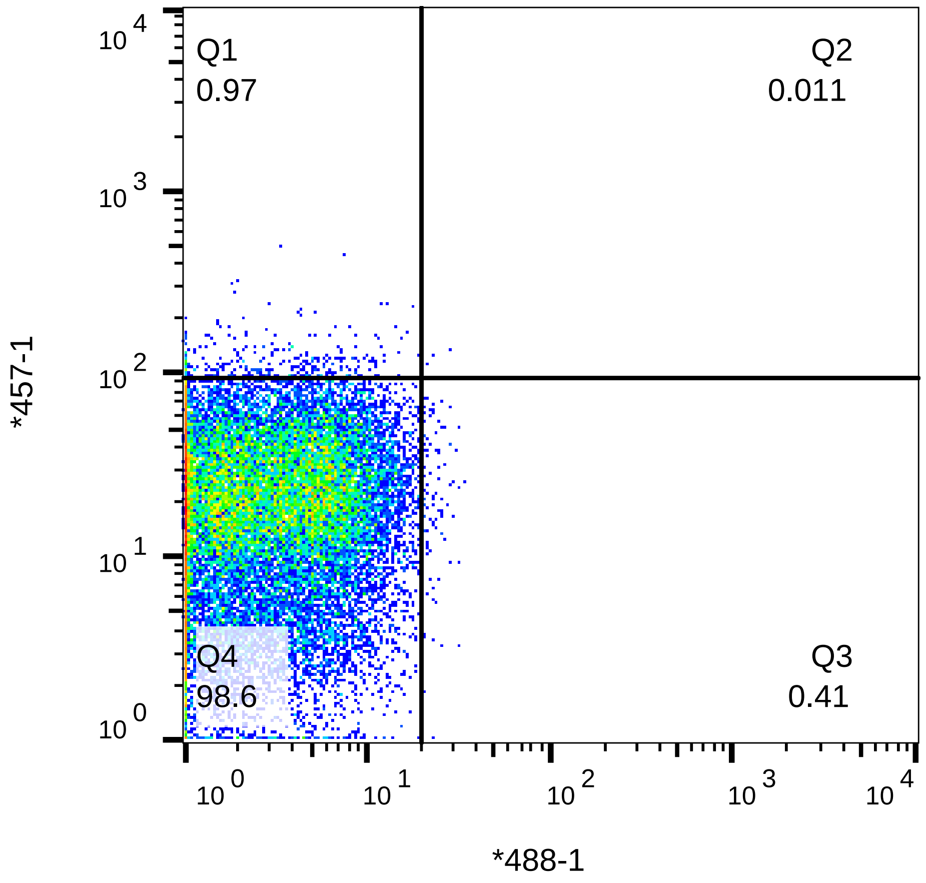

C:\Data\2019\Chrisler\20191219 Shank\112.fcs
All pop
26369

ES2256; SY383; amyE::PcomGA-Ypet (cam); lacA::PsboA-mTurq (erm)

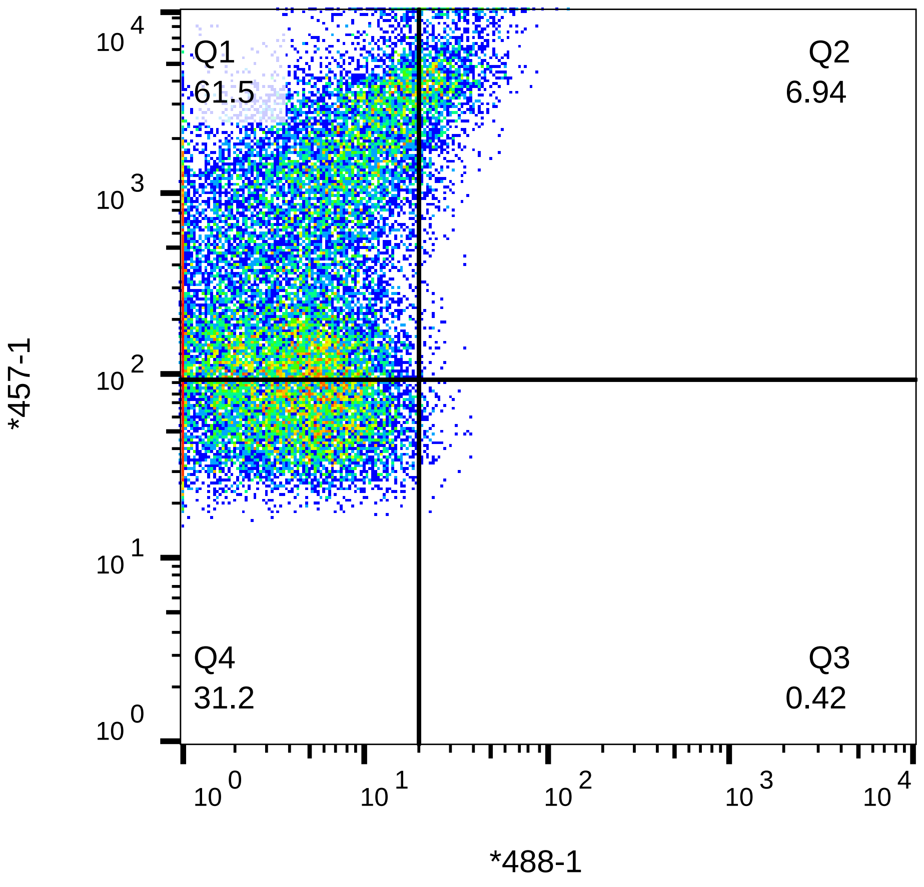

C:\Data\2019\Chrisler\20191219 Shank\113.fcs
All pop
26186

ES2258; SY385; amyE::PcomGA-Ypet (cam); lacA::PcomQX-mTurq (erm)

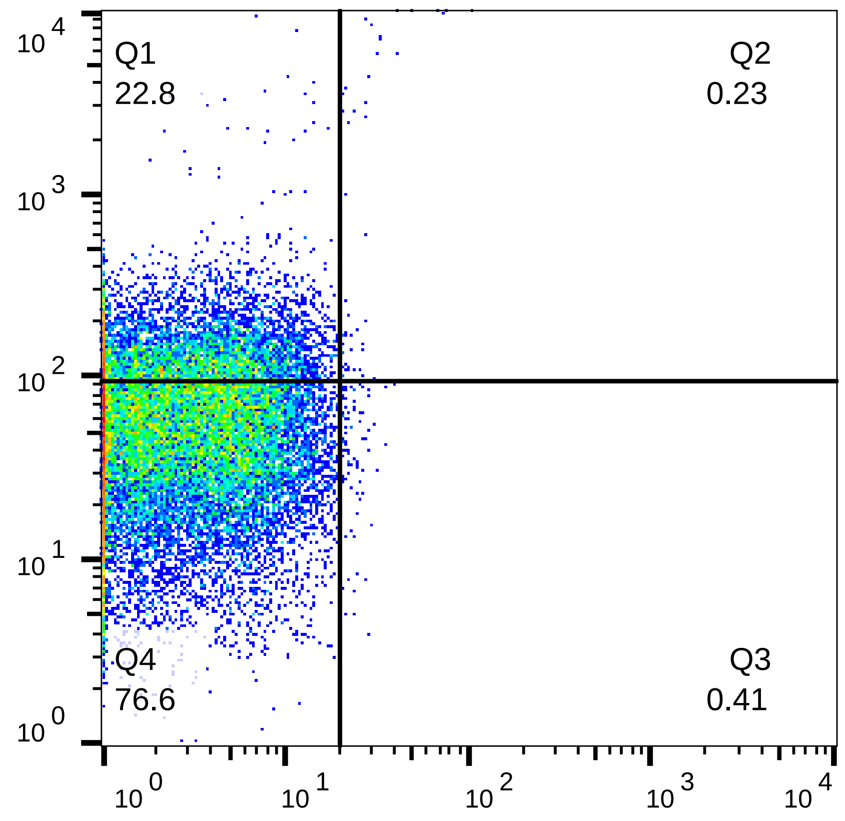

C:\Data\2019\Chrisler\20191219 Shank\115.fcs
All pop
25351

ES2262; SY389; amyE::PcomGA-Ypet (cam); lacA::PskfA-mTurq (erm)

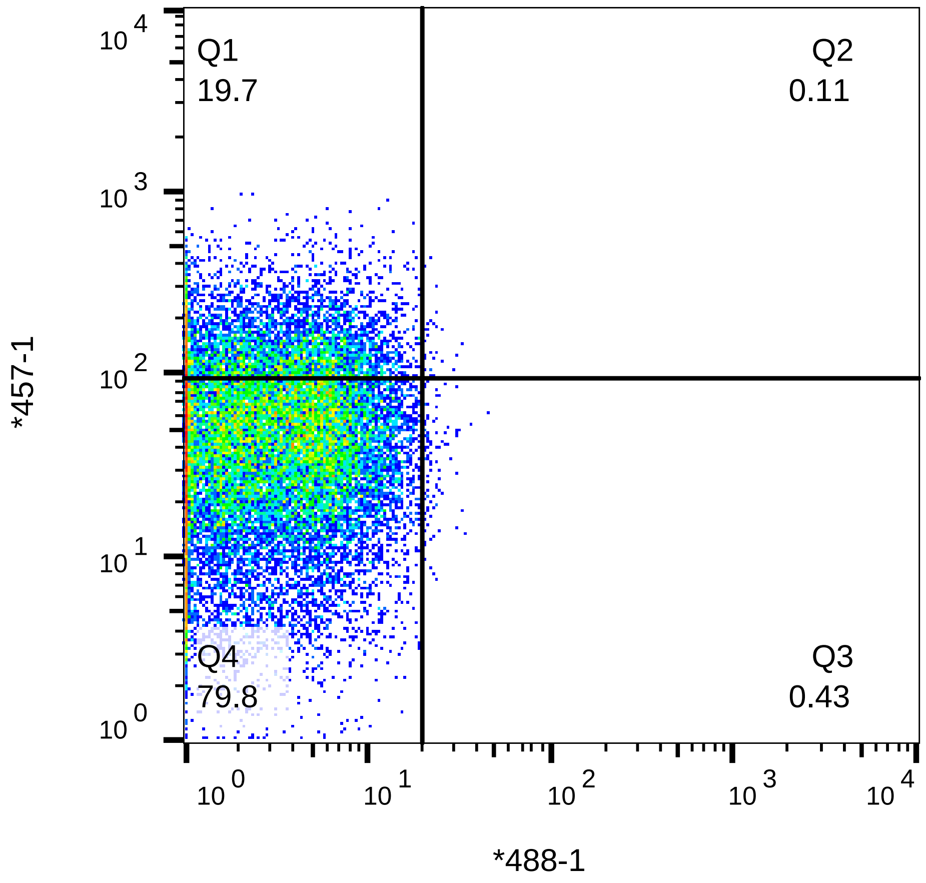

C:\Data\2019\Chrisler\20191219 Shank\119.fcs
All pop
26703

ES2263; SY390; amyE::PcomGA-Ypet (cam); lacA::PaprE-mTurq (erm)

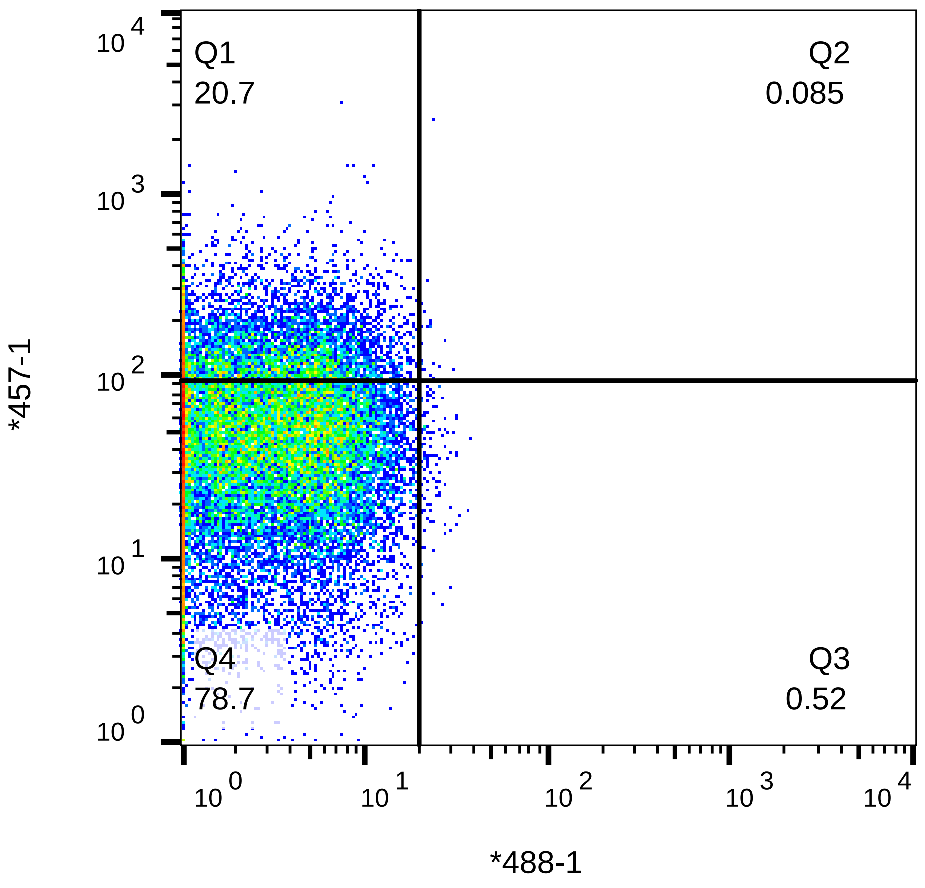

C:\Data\2019\Chrisler\20191219 Shank\120.fcs
All pop
27111

ES2265; SY392; amyE::PpksC-Ypet (cam); lacA::PdhbA-mTurq (erm)

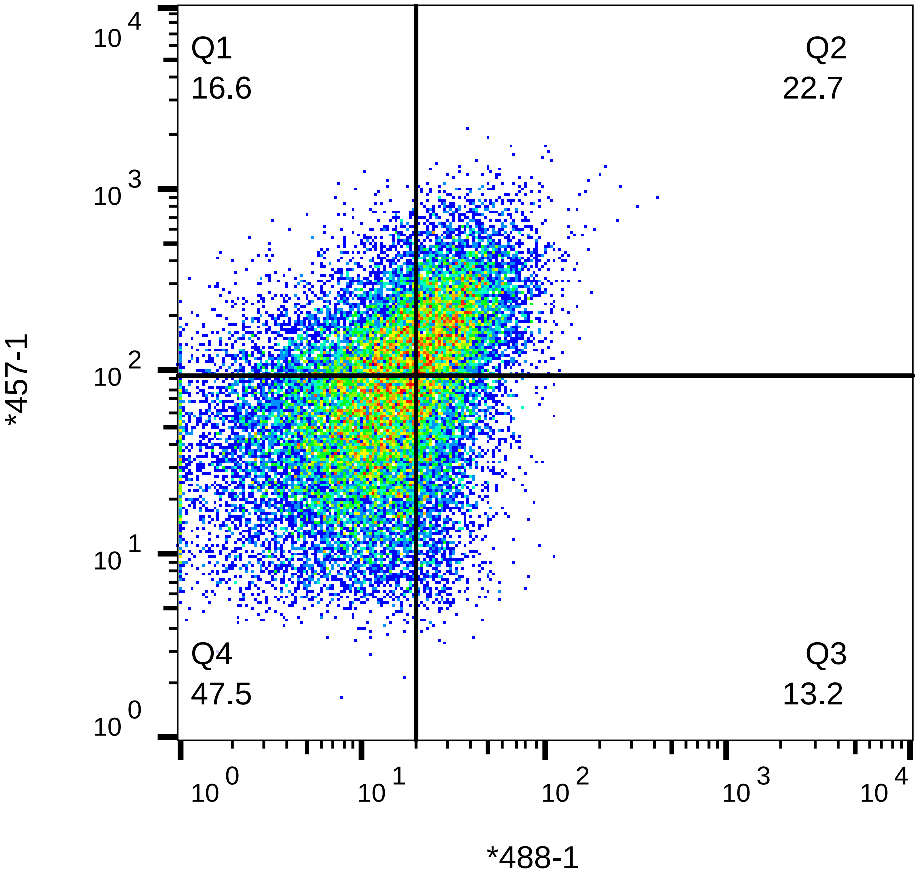

C:\Data\2019\Chrisler\20191219 Shank\122.fcs
All pop
25998

ES2266; SY393; amyE::PpksC-Ypet (cam); lacA::PbacA-mTurq (erm)

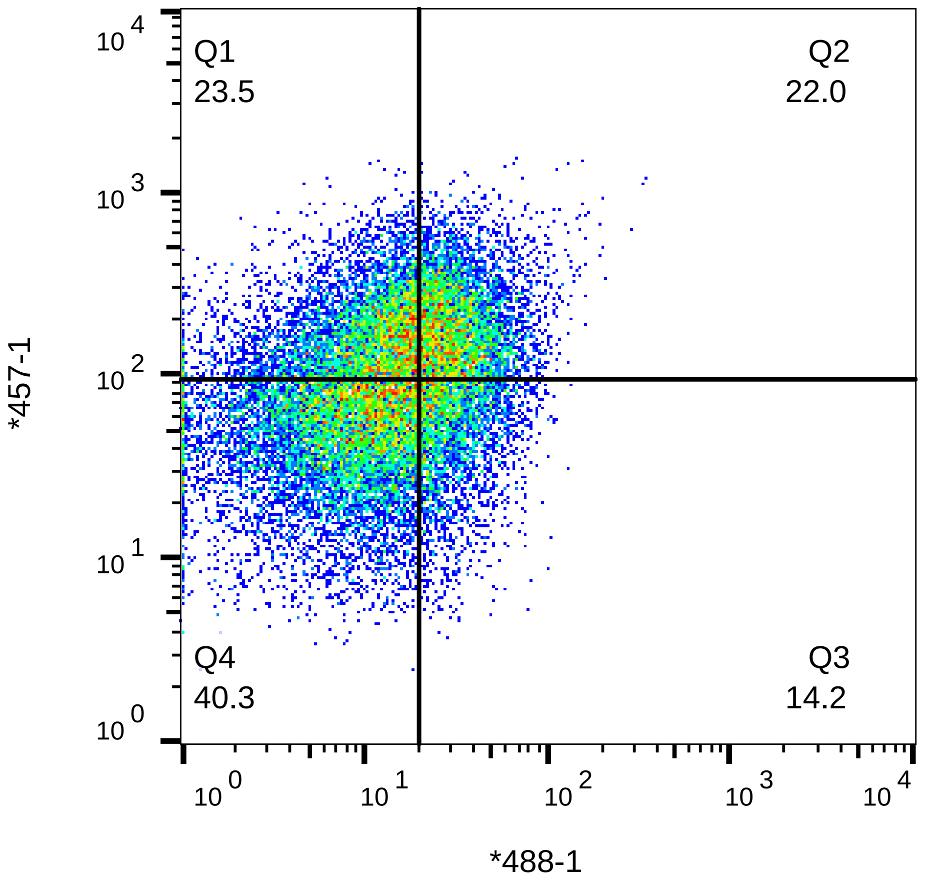

C:\Data\2019\Chrisler\20191219 Shank\123.fcs
All pop
26568

ES2267; SY394; amyE::PpksC-Ypet (cam); lacA::PppsA-mTurq (erm)

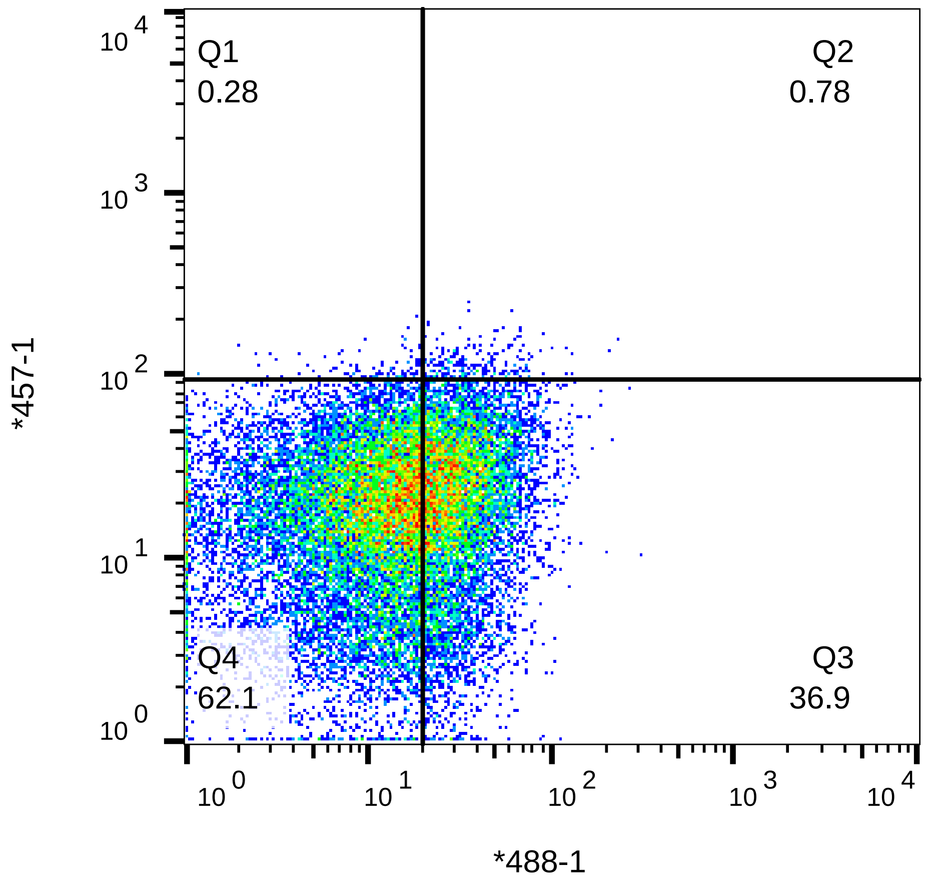

C:\Data\2019\Chrisler\20191219 Shank\124.fcs
All pop
25886

ES2268; SY395; amyE::PpksC-Ypet (cam); lacA::PsrfAA-mTurq (erm)

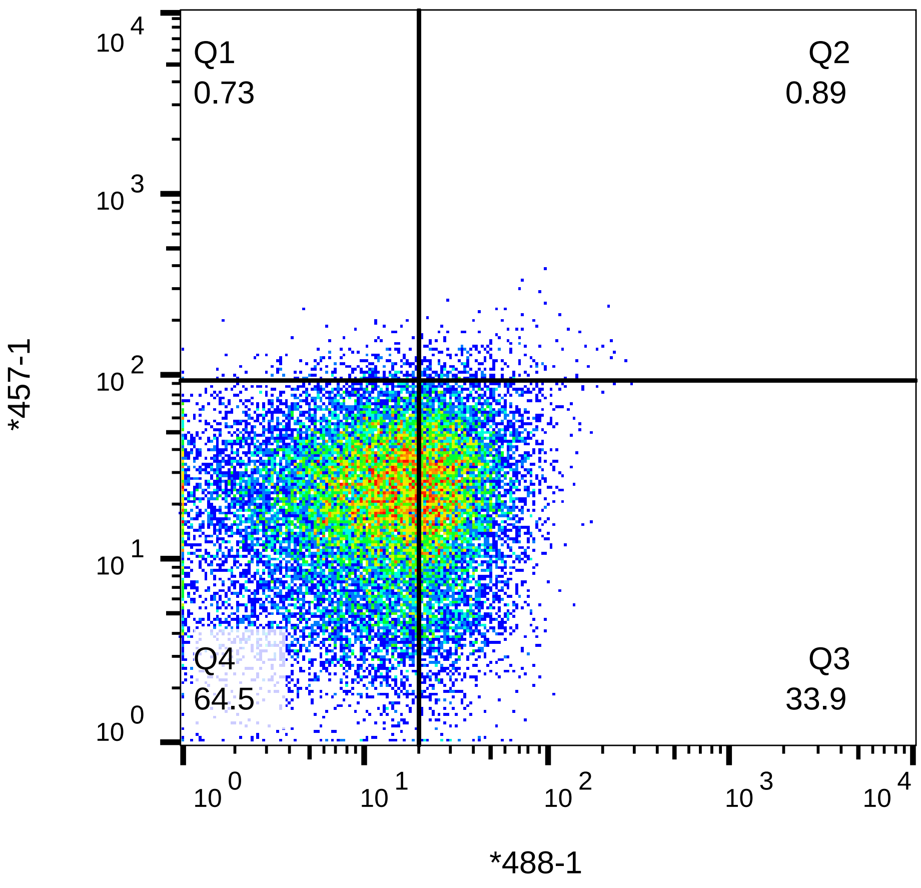

C:\Data\2019\Chrisler\20191219 Shank\125.fcs
All pop
26555

ES2269; SY396; amyE::PpksC-Ypet (cam); lacA::PsboA-mTurq (erm)

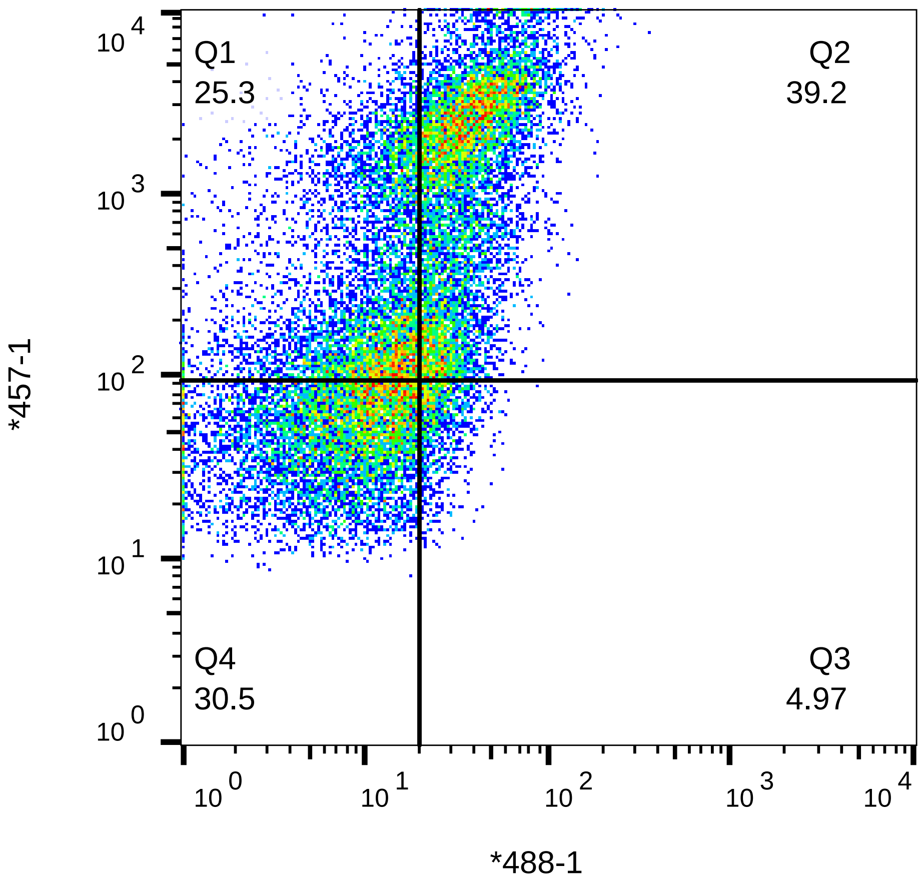

C:\Data\2019\Chrisler\20191219 Shank\126.fcs
All pop
25936

ES2271; SY398; amyE::PpksC-Ypet (cam); lacA::PcomQX-mTurq (erm)

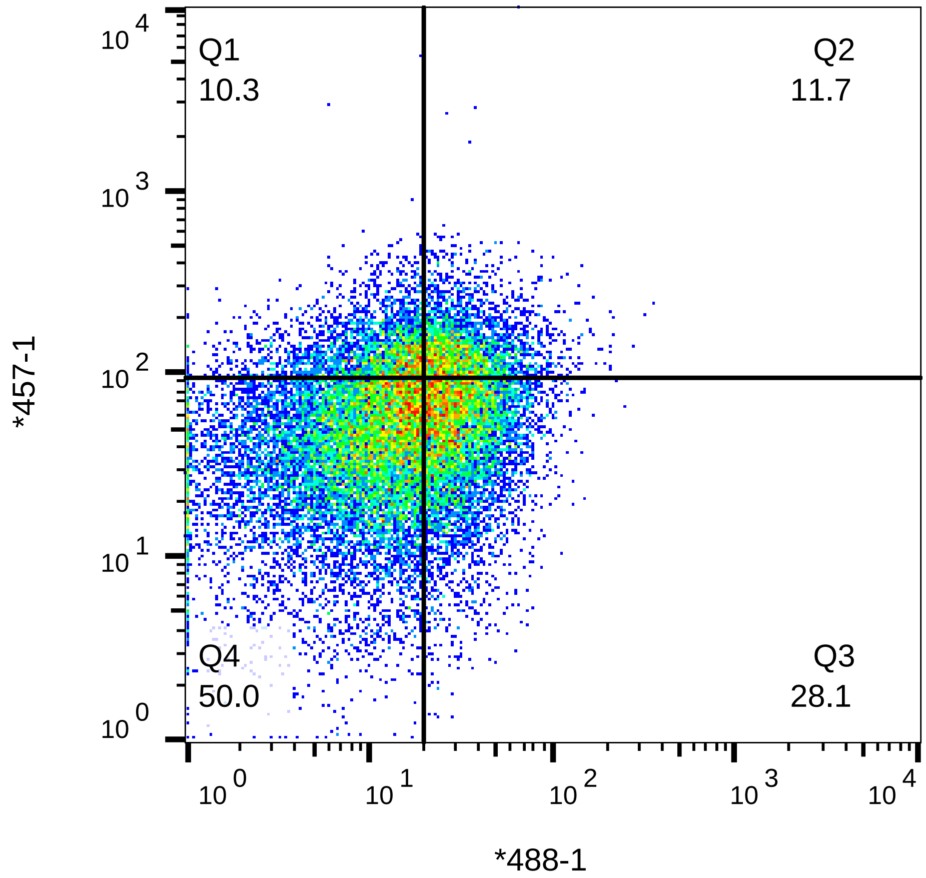

C:\Data\2019\Chrisler\20191219 Shank\128.fcs
All pop
25652

ES2275; SY402; amyE::PpksC-Ypet (cam); lacA::PskfA-mTurq (erm)

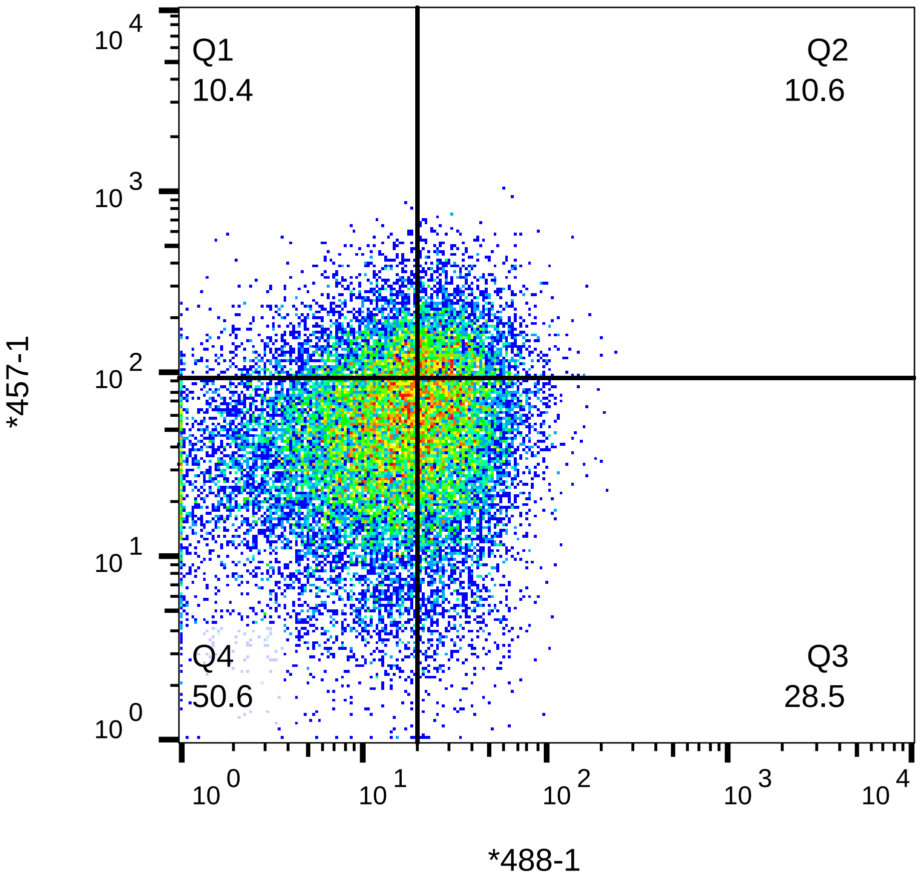

C:\Data\2019\Chrisler\20191219 Shank\132.fcs
All pop
25678

ES2276; SY403; amyE::PpksC-Ypet (cam); lacA::PaprE-mTurq (erm)

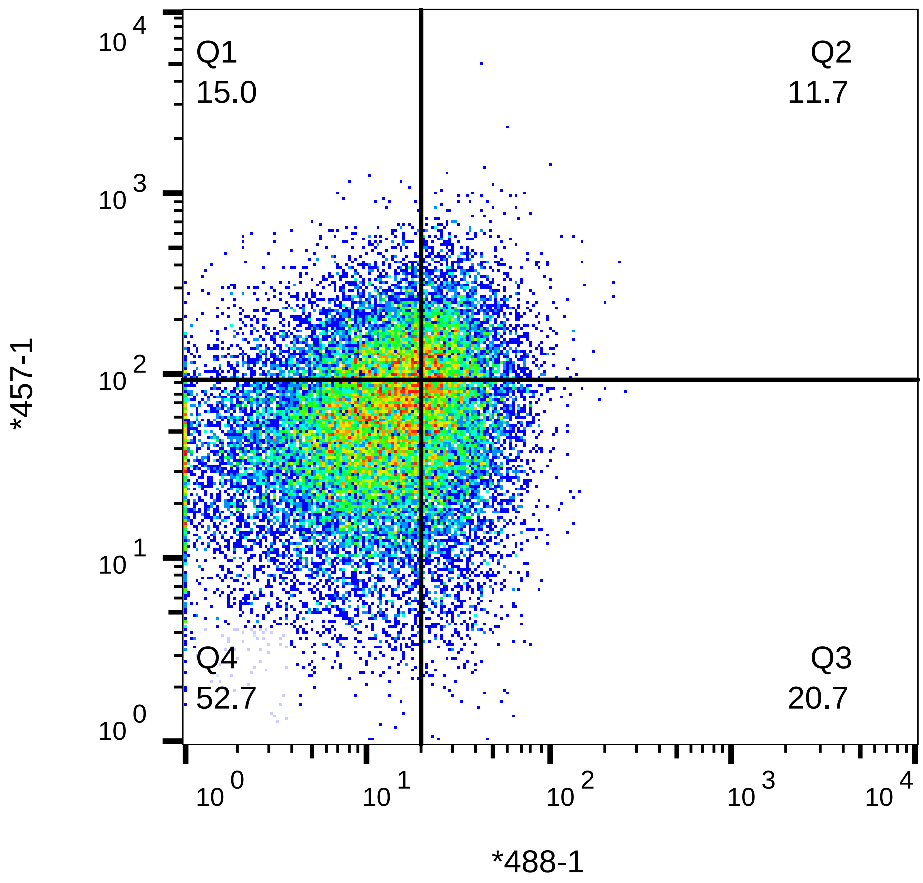

C:\Data\2019\Chrisler\20191219 Shank\133.fcs
All pop
27592

ES2279; SY405; amyE::PdhbA-Ypet (cam); lacA::PbacA-mTurq (erm)

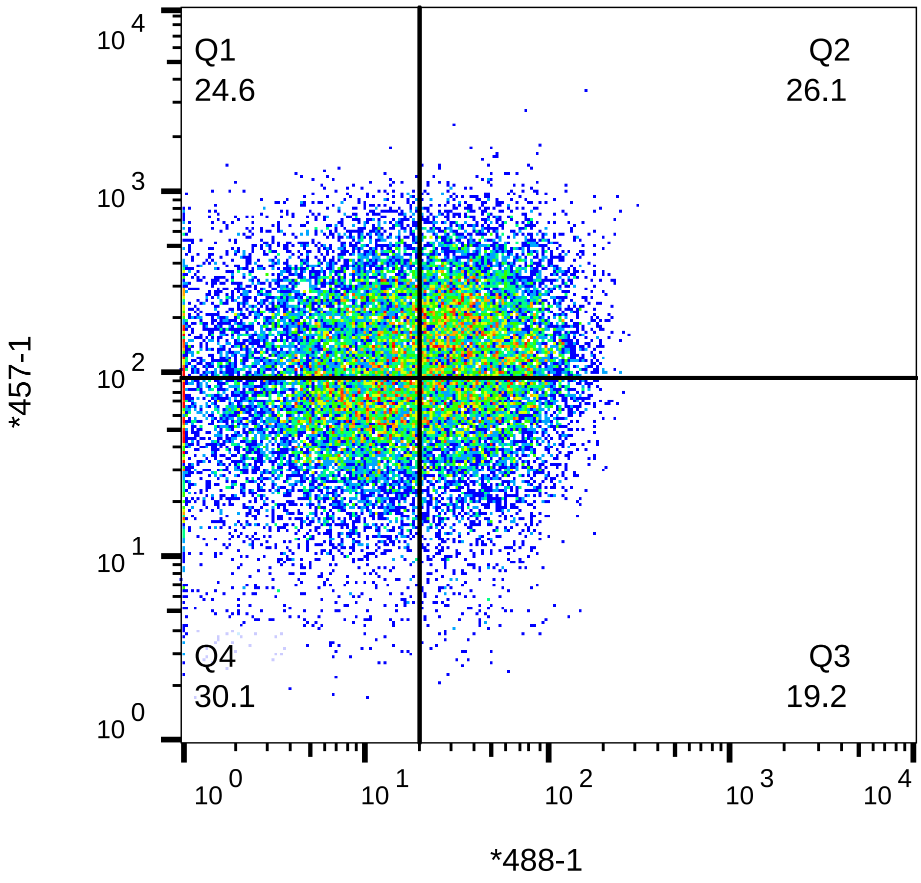

C:\Data\2019\Chrisler\20191219 Shank\135.fcs
All pop
27371

ES2280; SY406; amyE::PdhbA-Ypet (cam); lacA::PppsA-mTurq (erm)

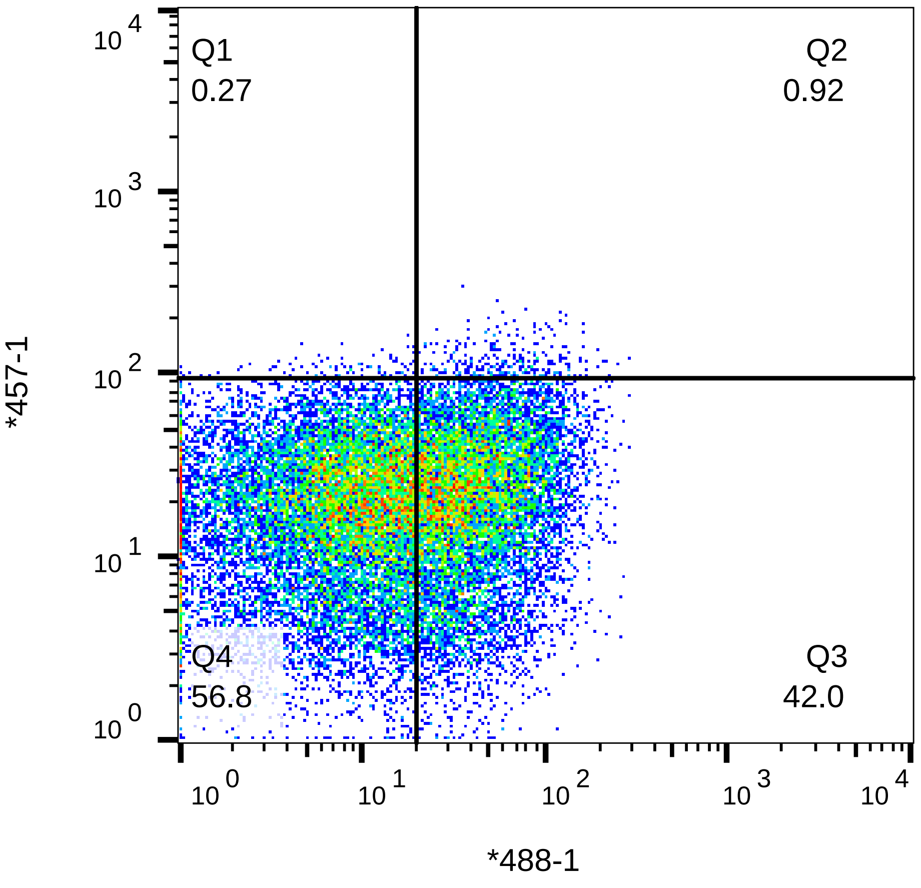

C:\Data\2019\Chrisler\20191219 Shank\136.fcs
All pop
27408

ES2281; SY407; amyE::PdhbA-Ypet (cam); lacA::PsrfAA-mTurq (erm)

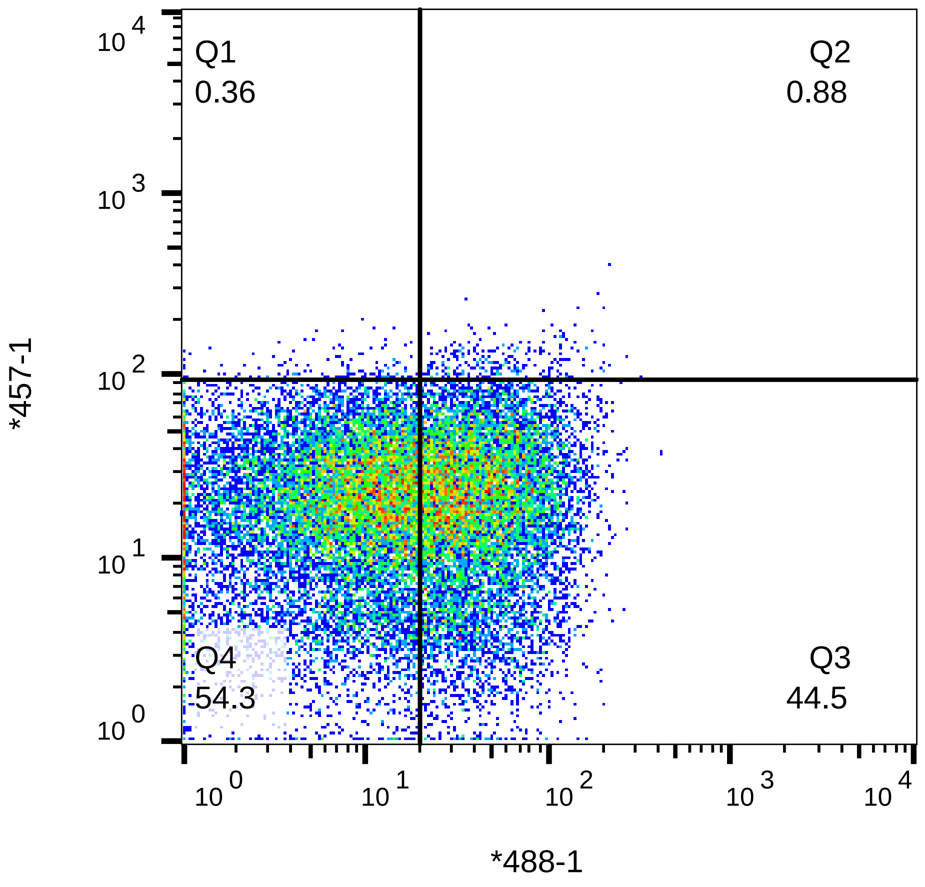

C:\Data\2019\Chrisler\20191219 Shank\137.fcs
All pop
26522

ES2282; SY408; amyE::PdhbA-Ypet (cam); lacA::PsboA-mTurq (erm)

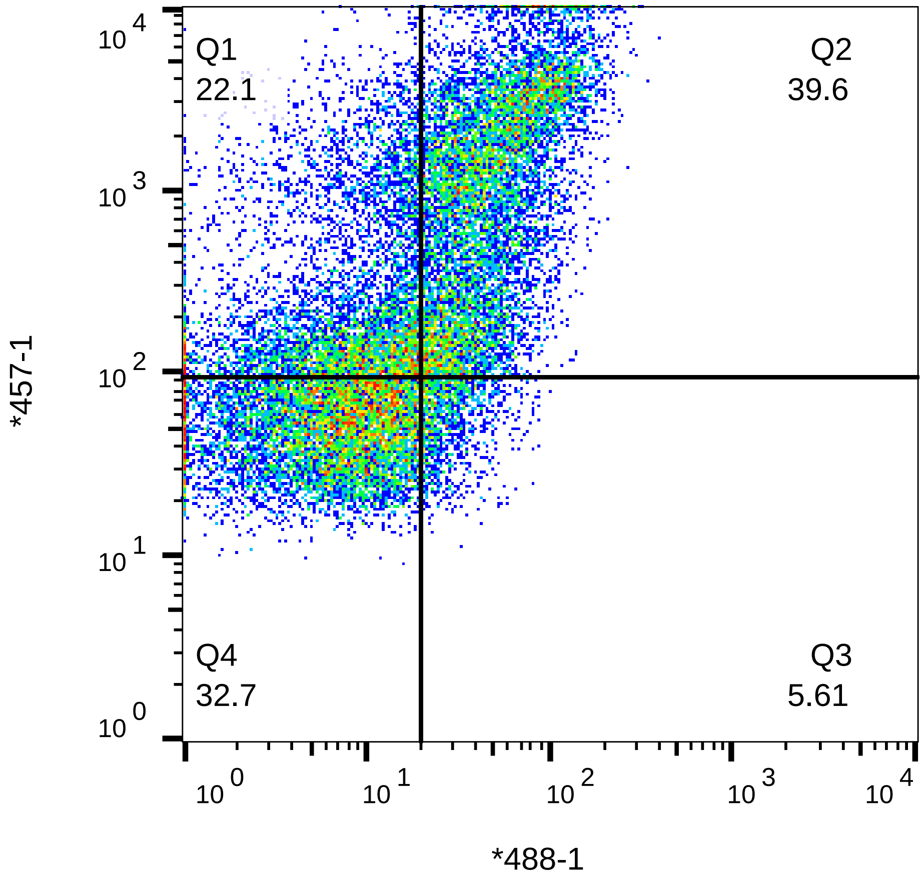

C:\Data\2019\Chrisler\20191219 Shank\138.fcs
All pop
26823

ES2288; SY414; amyE::PdhbA-Ypet (cam); lacA::PskfA-mTurq (erm)

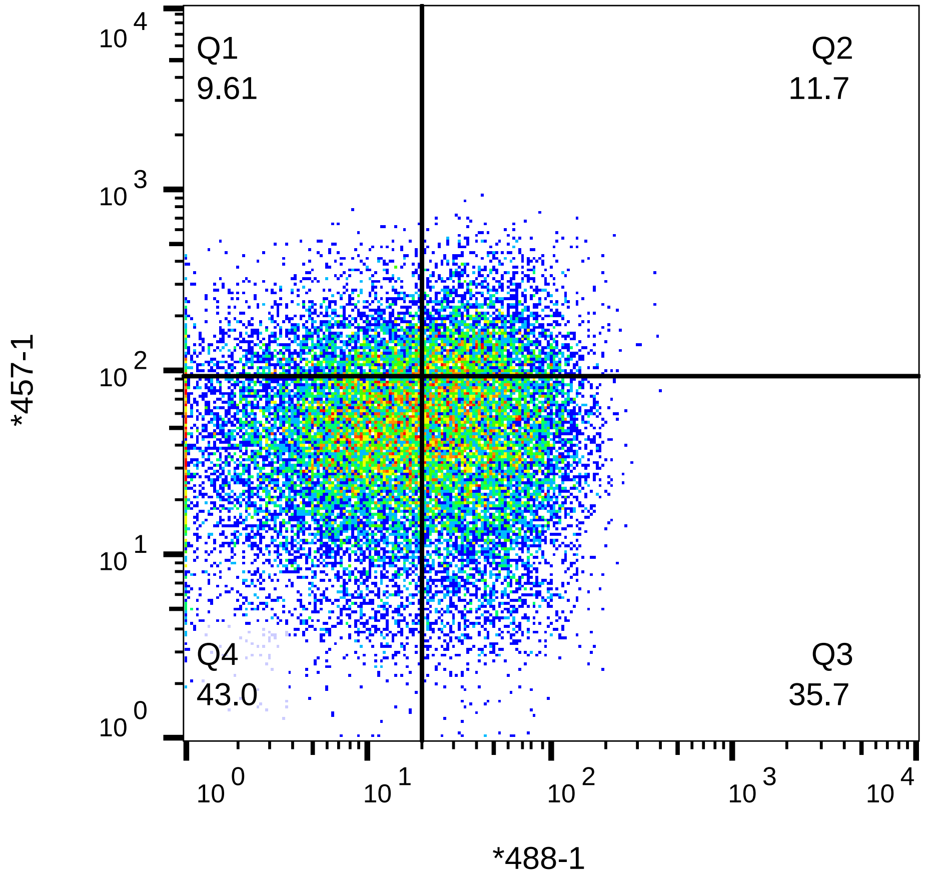

C:\Data\2019\Chrisler\20191219 Shank\144.fcs
All pop
26761

ES2289; SY415; amyE::PdhbA-Ypet (cam); lacA::PaprE-mTurq (erm)

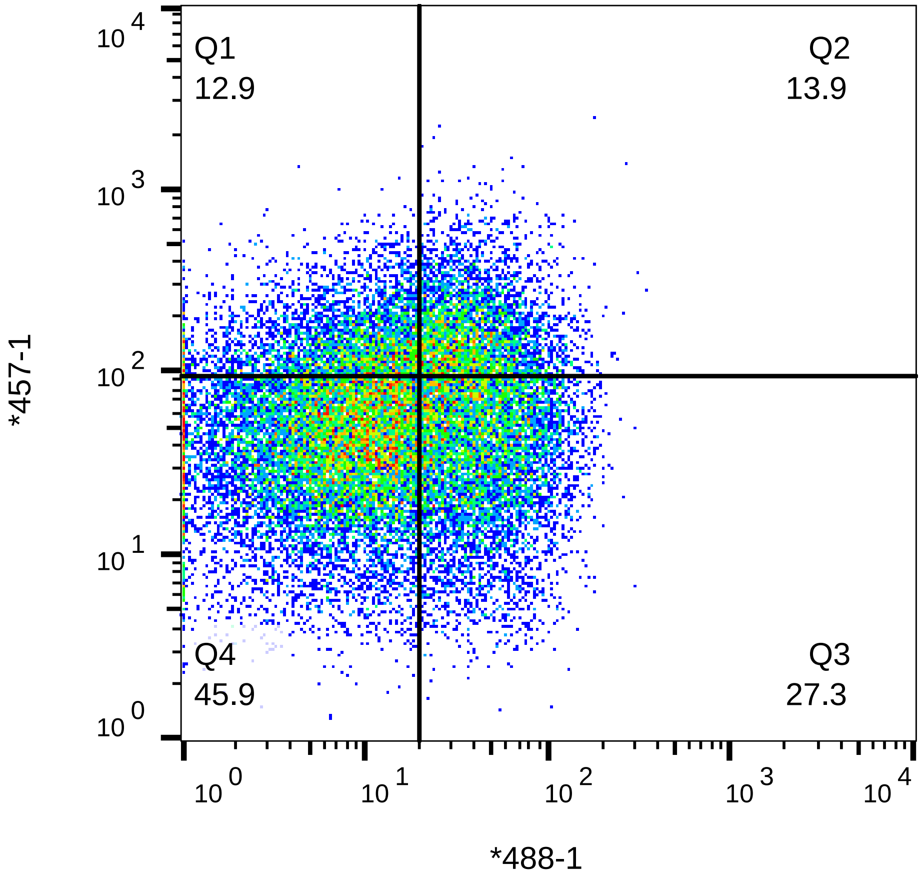

C:\Data\2019\Chrisler\20191219 Shank\145.fcs
All pop
28500

ES2291; SY417; amyE::PbacA-Ypet (cam); lacA::PppsA-mTurq (erm)

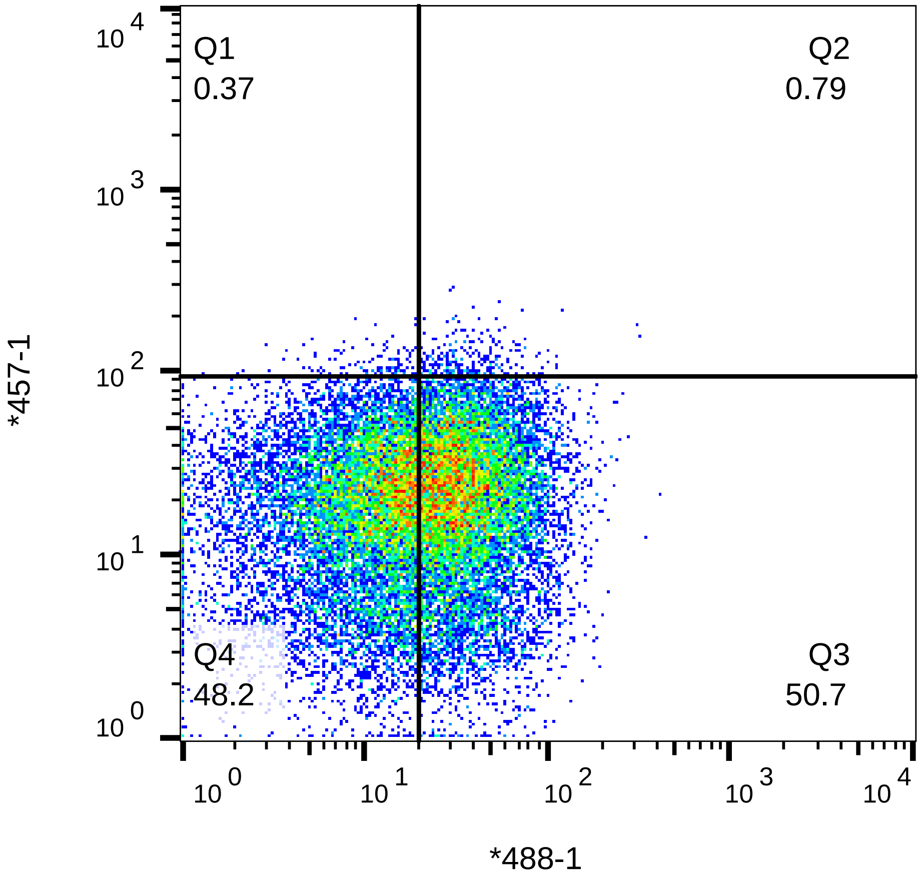

C:\Data\2019\Chrisler\20191219 Shank\147.fcs
All pop
25793

ES2292; SY418; amyE::PbacA-Ypet (cam); lacA::PsrfAA-mTurq (erm)

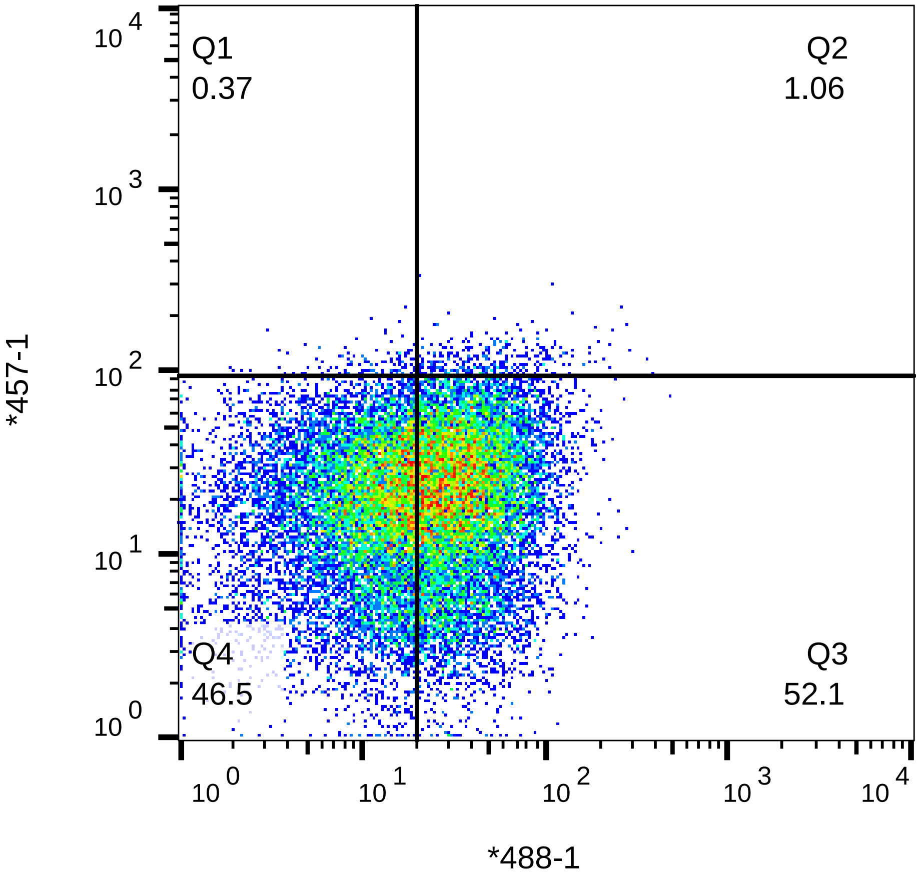

C:\Data\2019\Chrisler\20191219 Shank\148.fcs
All pop
26537

ES2293; SY419; amyE::PbacA-Ypet (cam); lacA::PsboA-mTurq (erm)

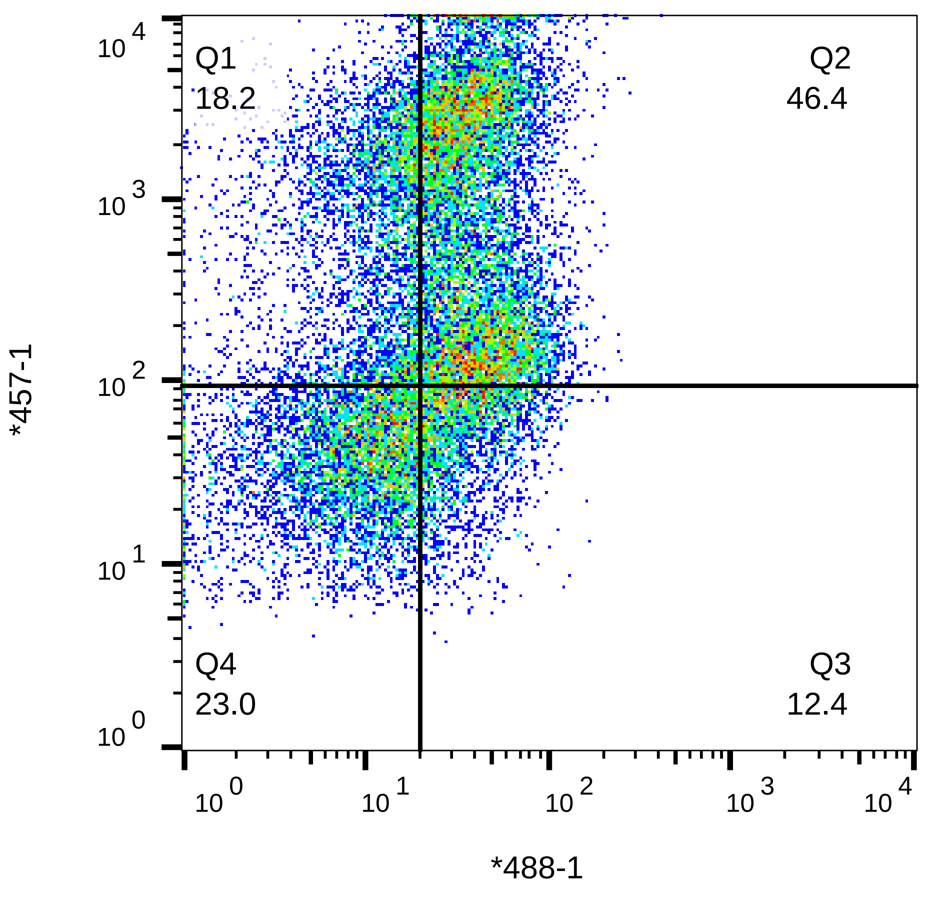

C:\Data\2019\Chrisler\20191219 Shank\149.fcs
All pop
25962

ES2295; SY421; amyE::PbacA-Ypet (cam); lacA::PcomQX-mTurq (erm)

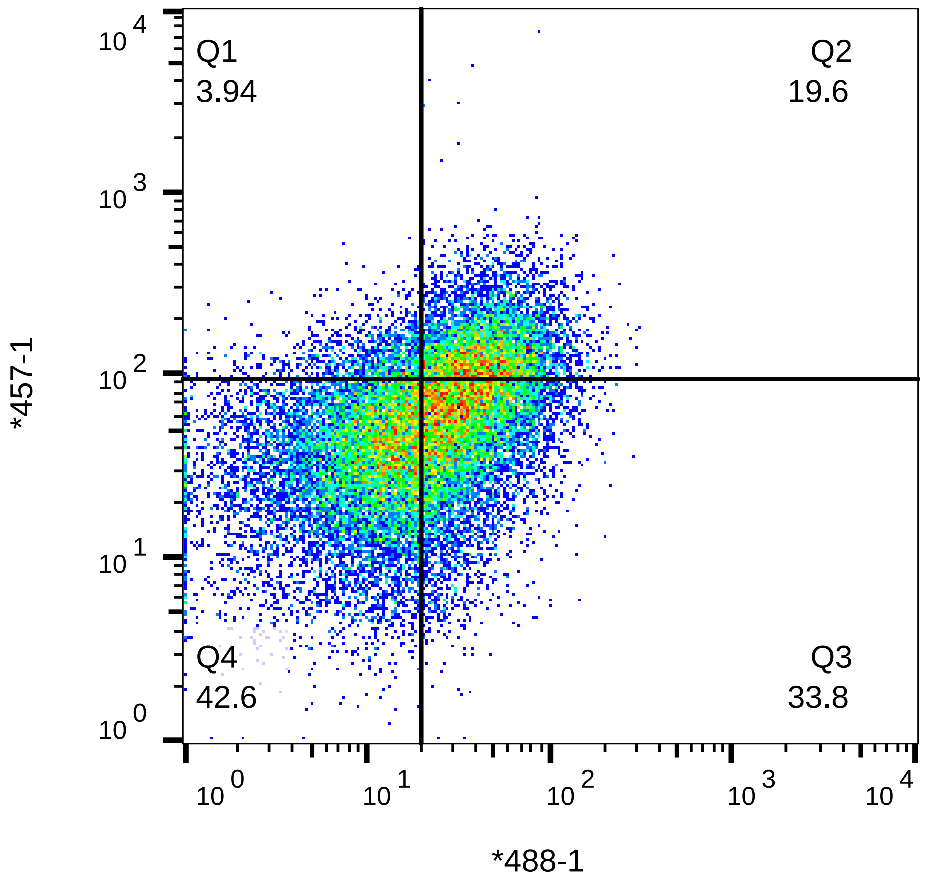

C:\Data\2019\Chrisler\20191219 Shank\151.fcs
All pop
27232

ES2299; SY425; amyE::PbacA-Ypet (cam); lacA::PskfA-mTurq (erm)

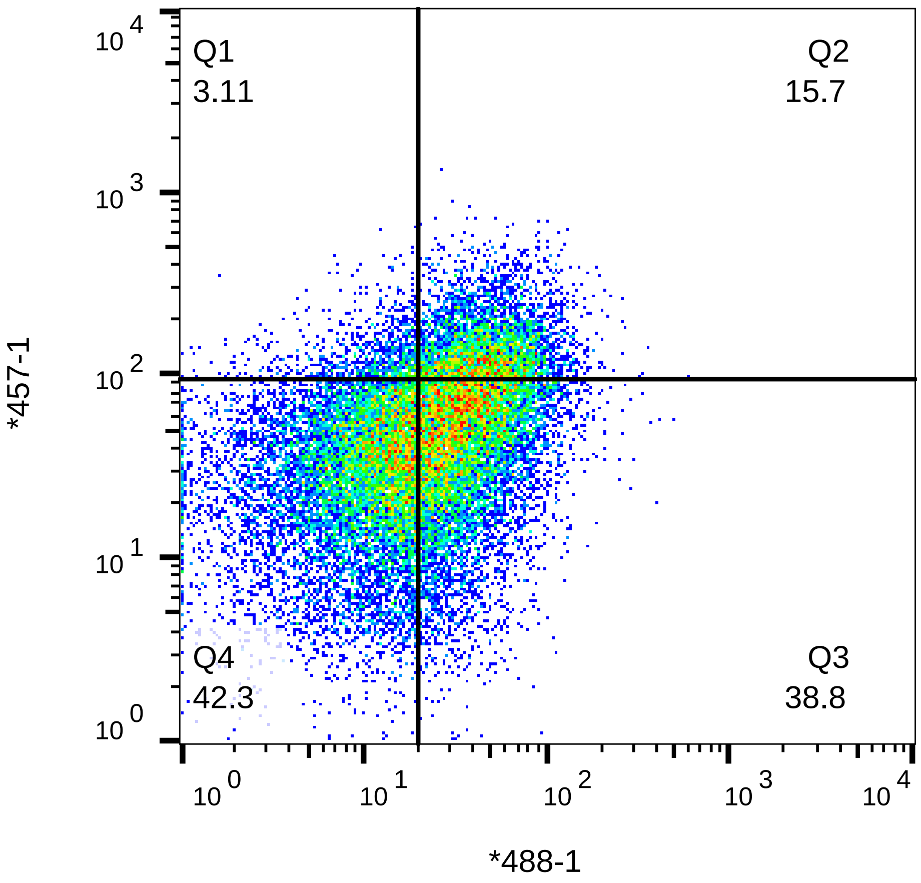

C:\Data\2019\Chrisler\20191219 Shank\155.fcs
All pop
26903

ES2300; SY426; amyE::PbacA-Ypet (cam); lacA::PaprE-mTurq (erm)

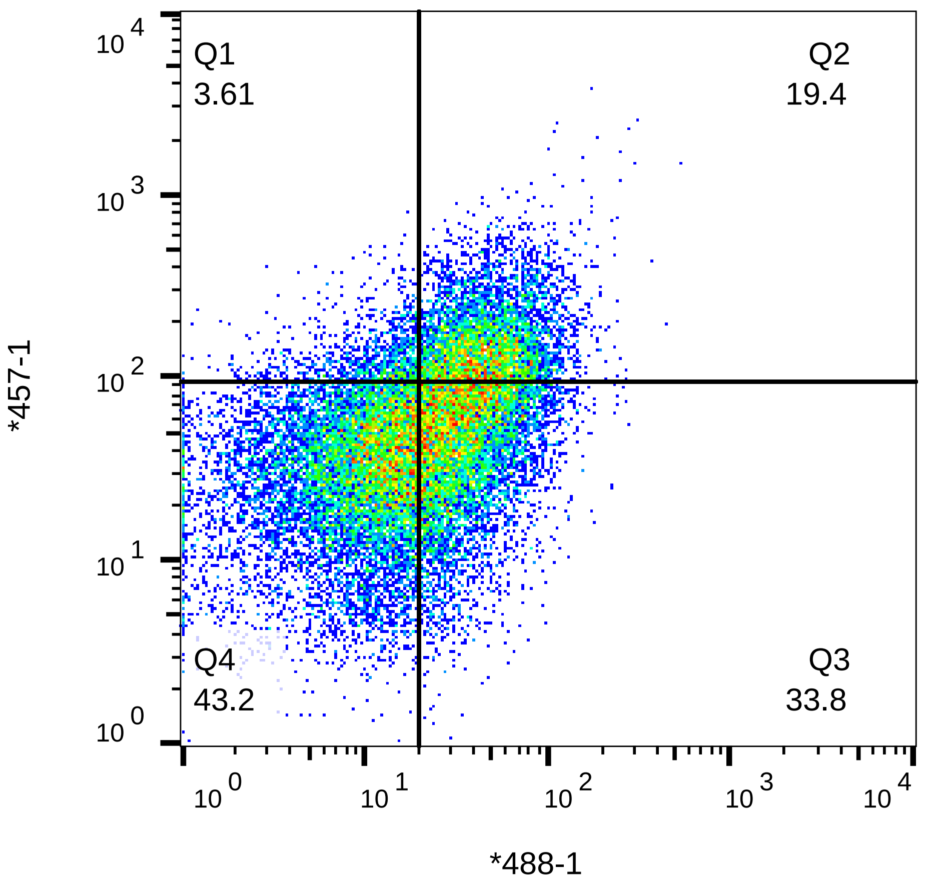

C:\Data\2019\Chrisler\20191219 Shank\156.fcs
All pop
27475

ES2302; SY428; amyE::PppsA-Ypet (cam); lacA::PsrfA-mTurq (erm)

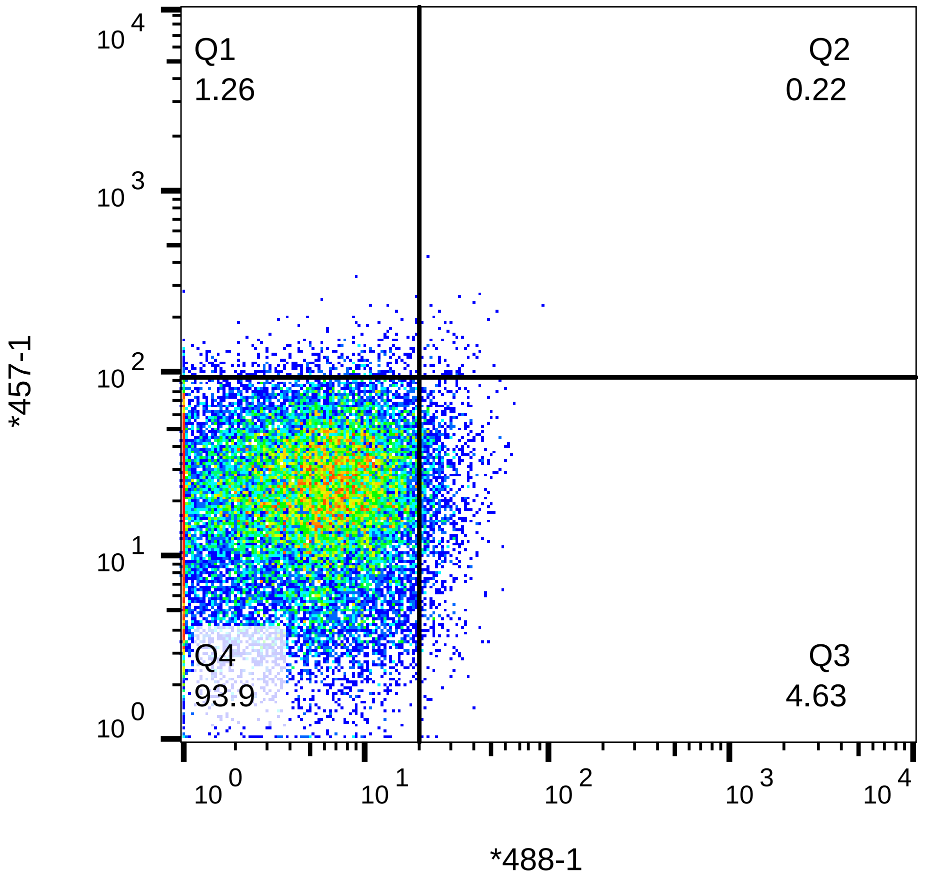

C:\Data\2019\Chrisler\20191219 Shank\158.fcs
All pop
26950

ES2305; SY431; amyE::PppsA-Ypet (cam); lacA::PcomQX-mTurq (erm)

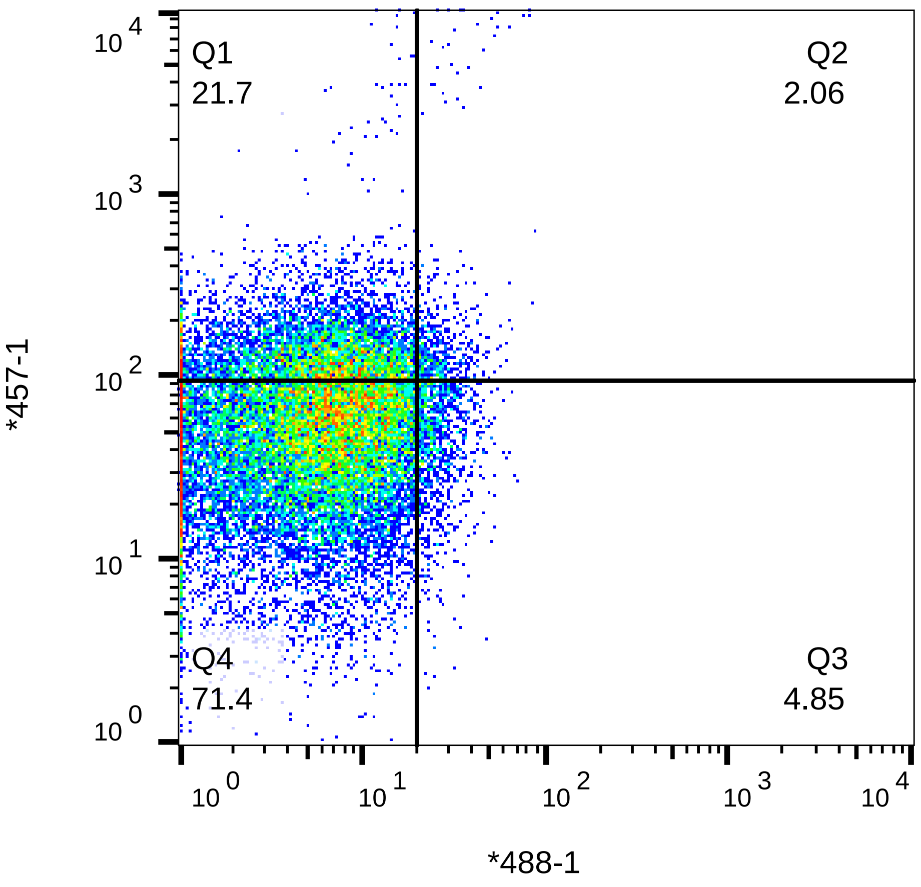

C:\Data\2019\Chrisler\20191219 Shank\161.fcs
All pop
24982

ES2309; SY435; amyE::PppsA-Ypet (cam); lacA::PskfA-mTurq (erm)

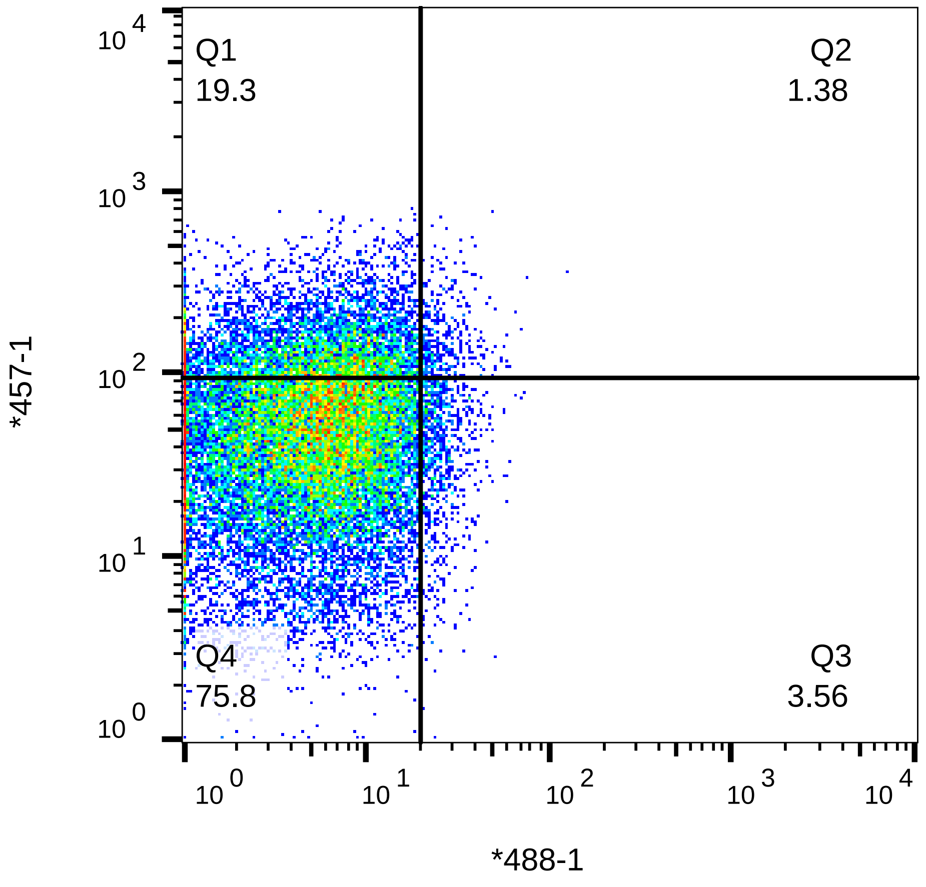

C:\Data\2019\Chrisler\20191219 Shank\165.fcs
All pop
27020

ES2310; SY436; amyE::PppsA-Ypet (cam); lacA::PaprE-mTurq (erm)

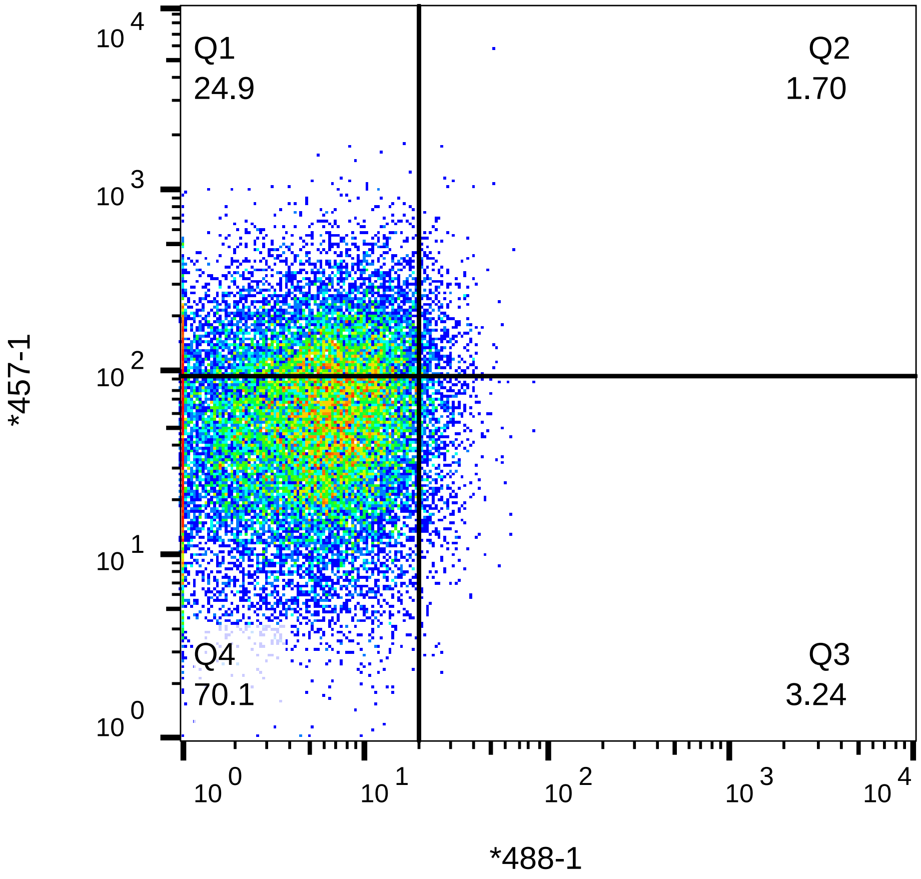

C:\Data\2019\Chrisler\20191219 Shank\166.fcs
All pop
26993

ES2312; SY438; amyE::PsrfAA-Ypet (cam); lacA::PsboA-mTurq (erm)

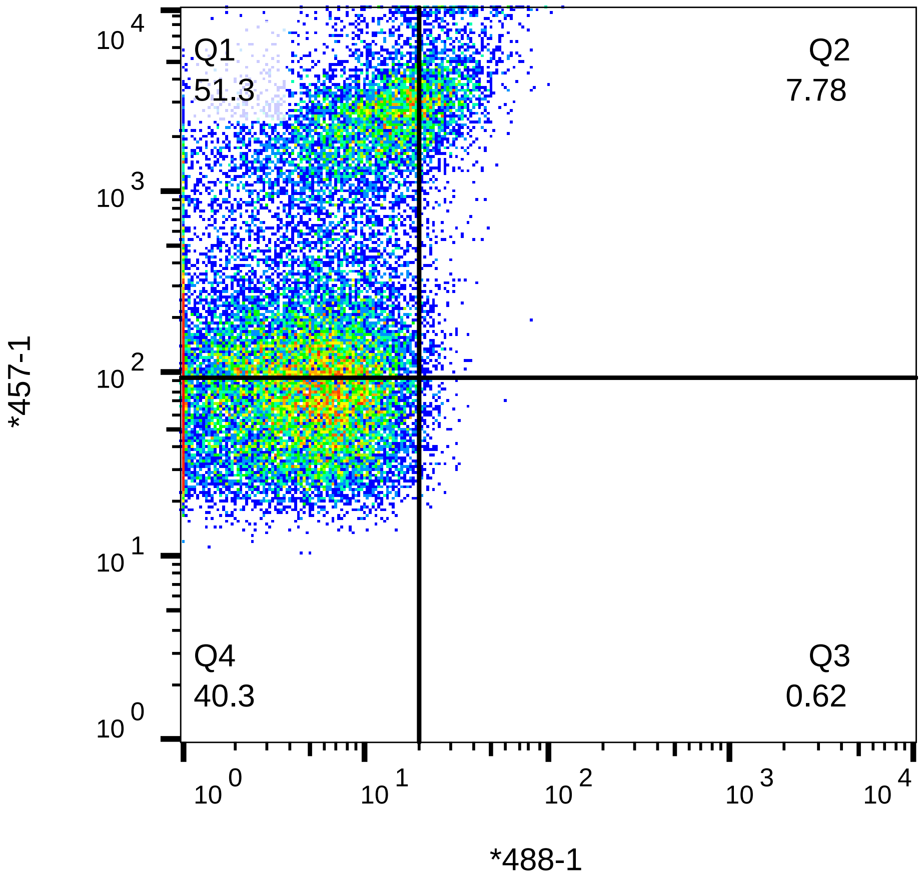

C:\Data\2019\Chrisler\20191219 Shank\168.fcs
All pop
27647

ES2314; SY440; amyE::PsrfAA-Ypet (cam); lacA::PcomQX-mTurq (erm)

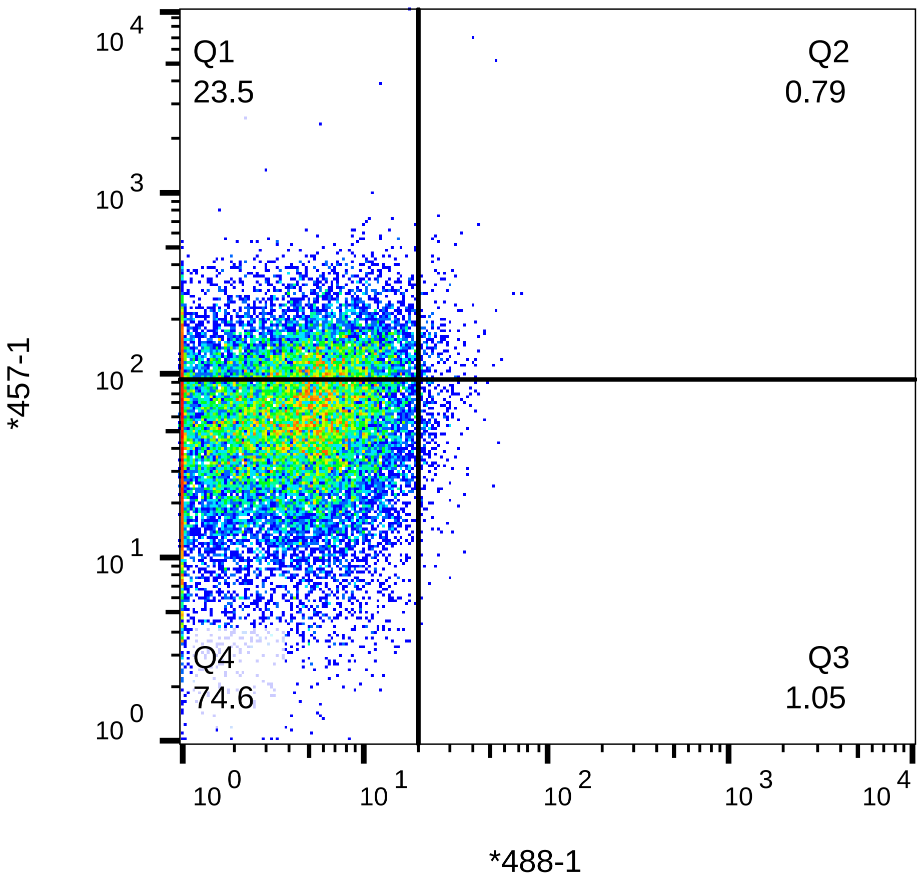

C:\Data\2019\Chrisler\20191219 Shank\170.fcs
All pop
26641

ES2318; SY444; amyE::PsrfAA-Ypet (cam); lacA::PskfA-mTurq (erm)

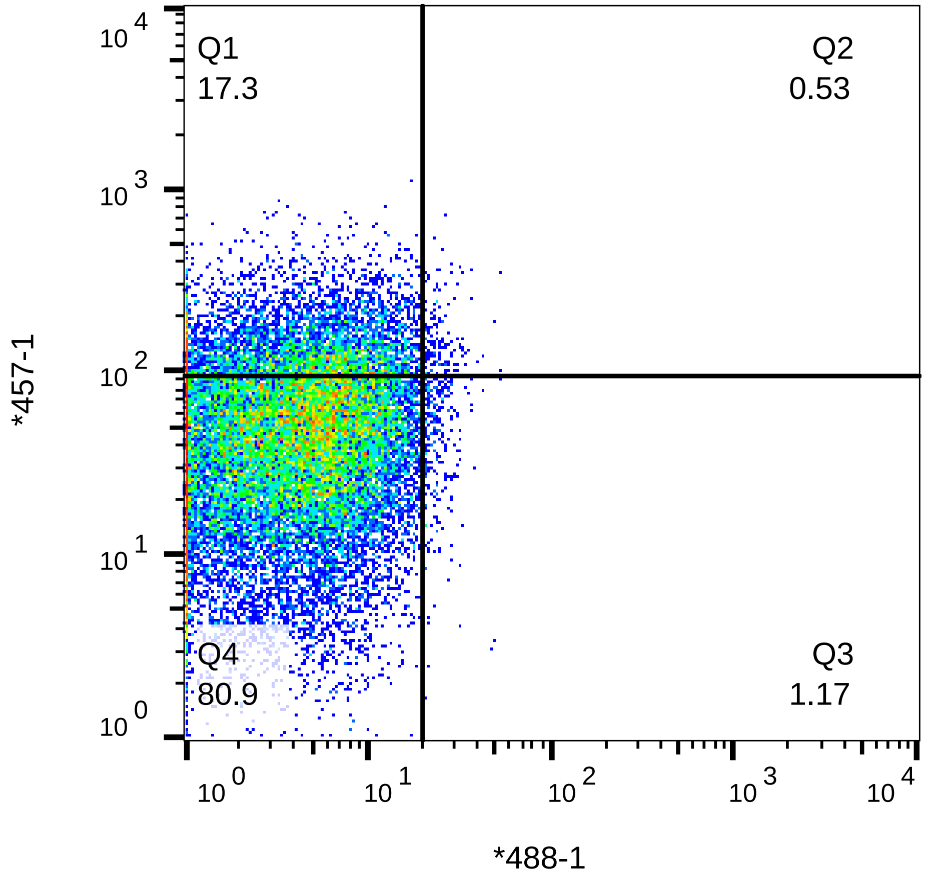

C:\Data\2019\Chrisler\20191219 Shank\174.fcs
All pop
27311

ES2319; SY445; amyE::PsrfAA-Ypet (cam); lacA::PaprE-mTurq (erm)

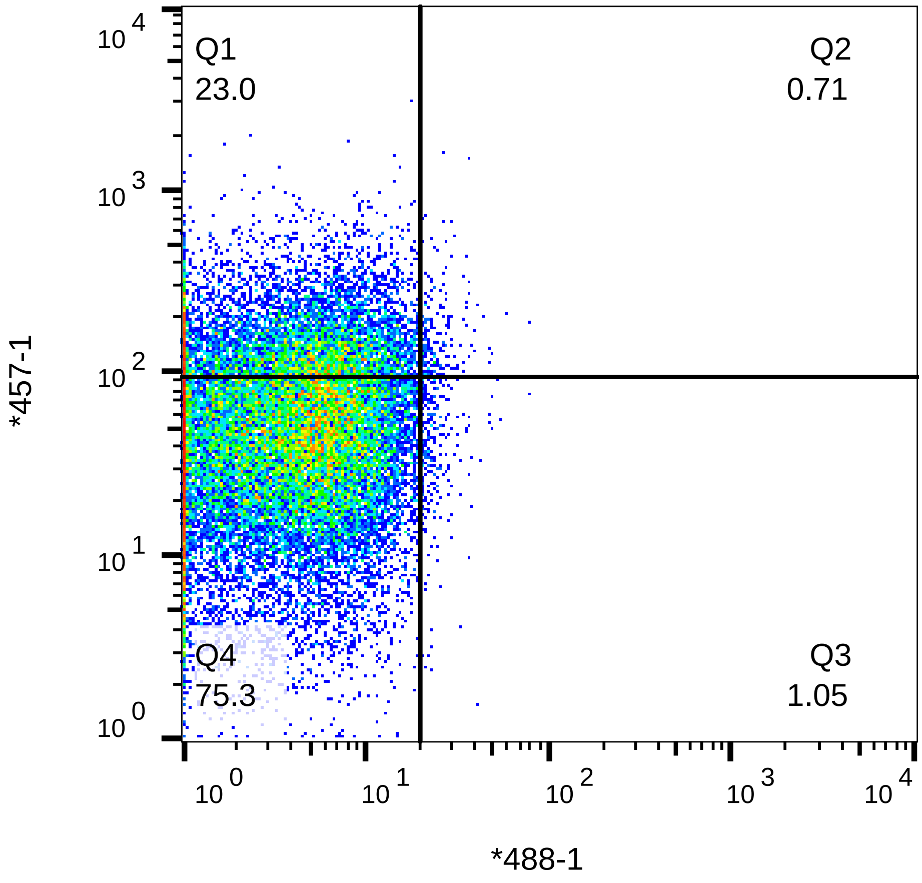

C:\Data\2019\Chrisler\20191219 Shank\175.fcs
All pop
28285

ES2322; SY448; amyE::PsboA-Ypet (cam); lacA::PcomQX-mTurq (erm)

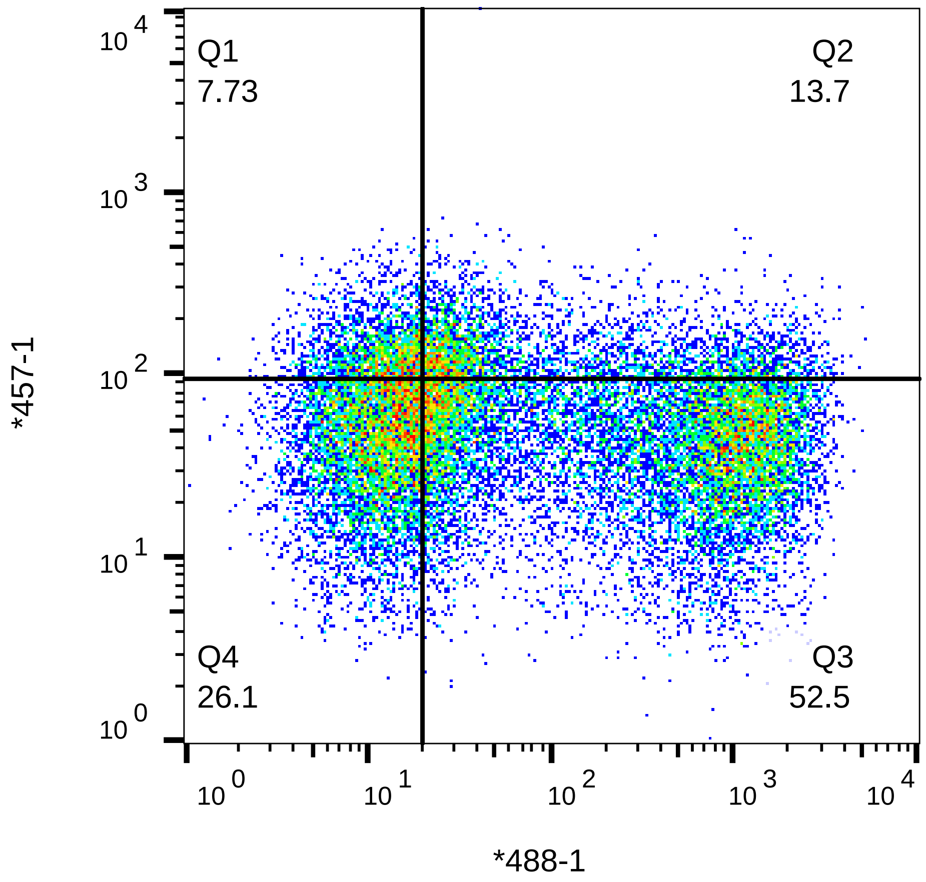

C:\Data\2019\Chrisler\20191219 Shank\178.fcs
All pop
25642

ES2326; SY452; amyE:: PsboA-Ypet (cam); lacA::PskfA-mTurq (erm)

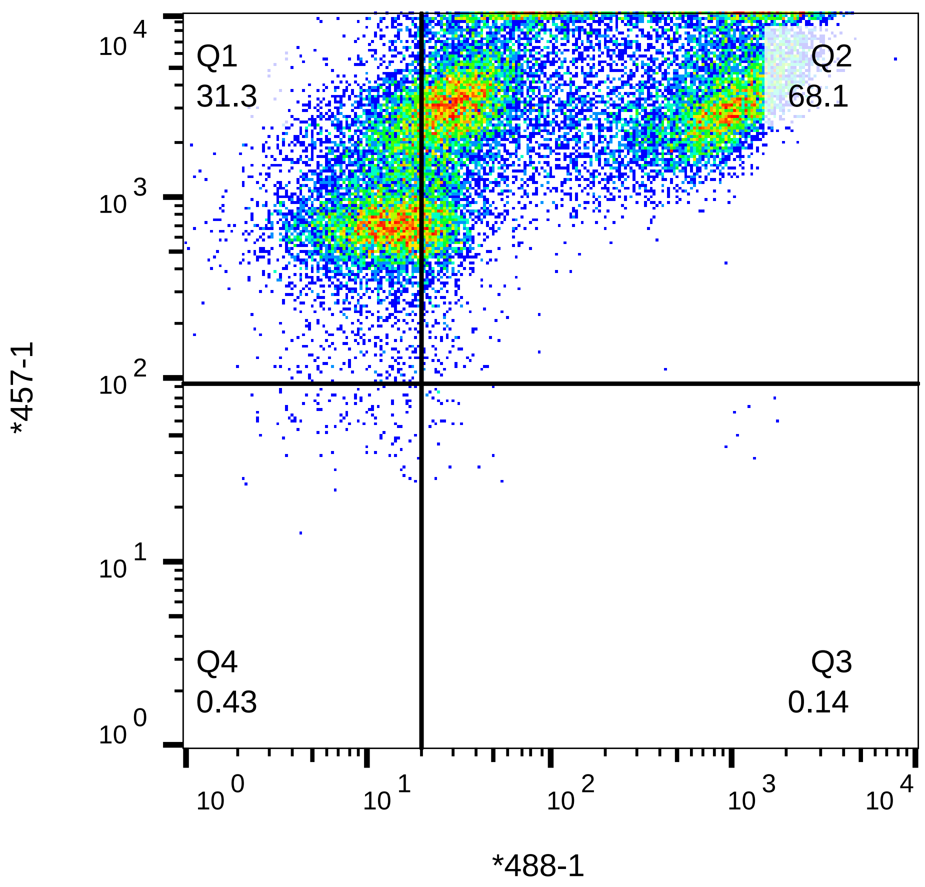

C:\Data\2019\Chrisler\20191219 Shank\182.fcs
All pop
27705

ES2327; SY453; amyE::PsboA-Ypet (cam); lacA::PaprE-mTurq (erm)

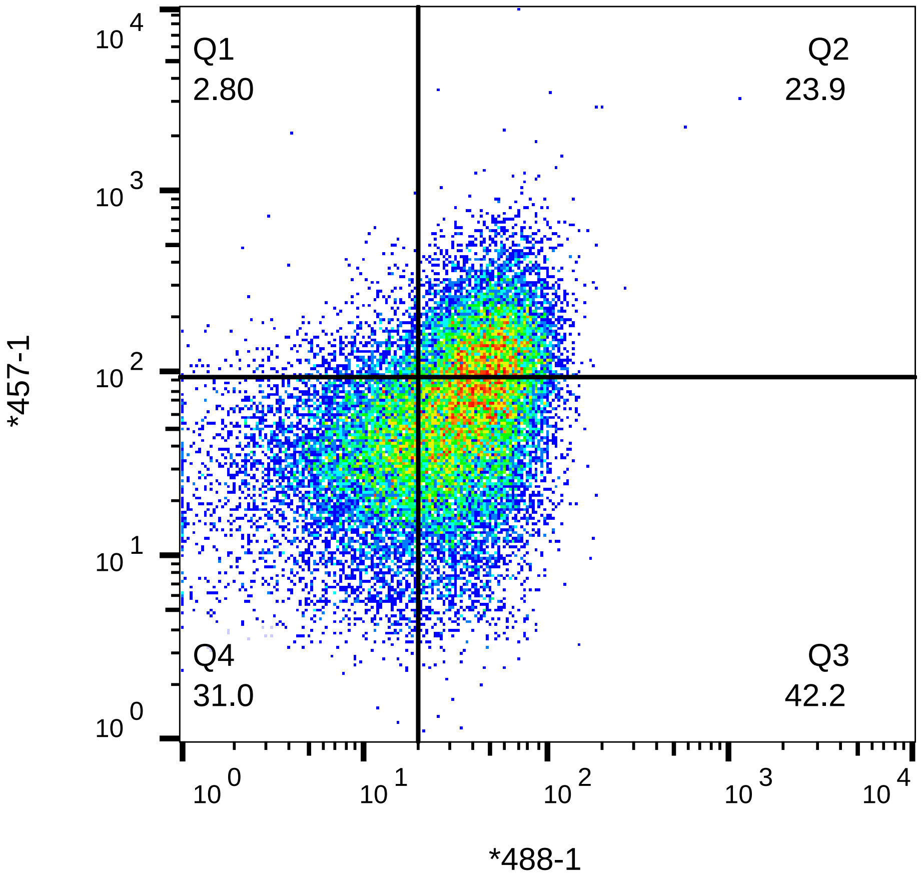

C:\Data\2019\Chrisler\20191219 Shank\183.fcs
All pop
27685

ES3 = NCIB3610 = WT

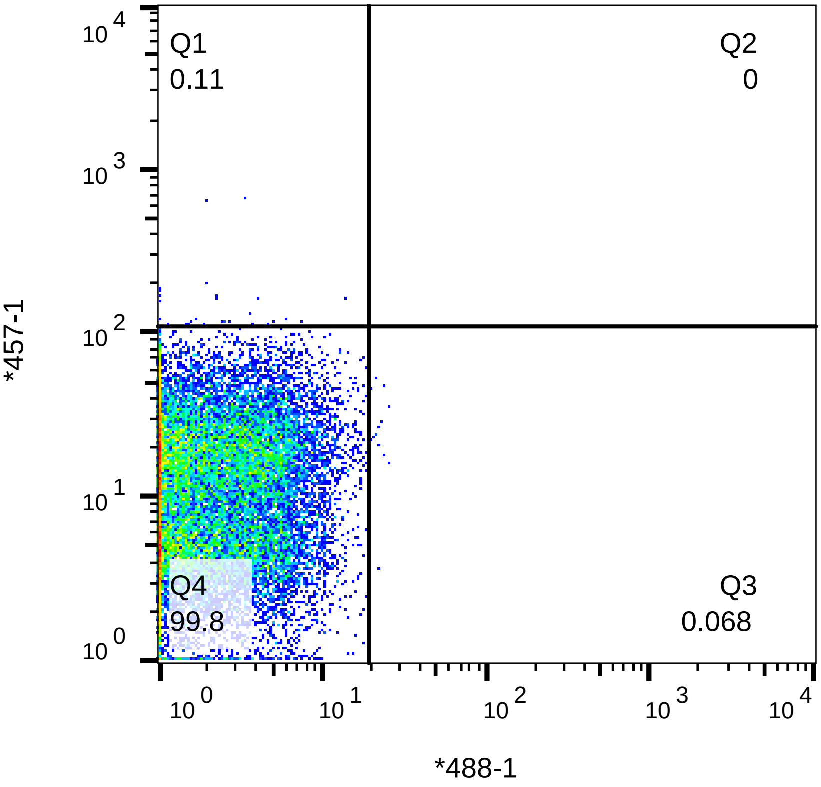

C:\Data\2019\Chrisler\20191220 Shank\No Fl Control.fcs
All pop
25119

ES2339; SY465; amyE::PcomQX-Ypet (cam); lacA::PskfA-mTurq (erm)

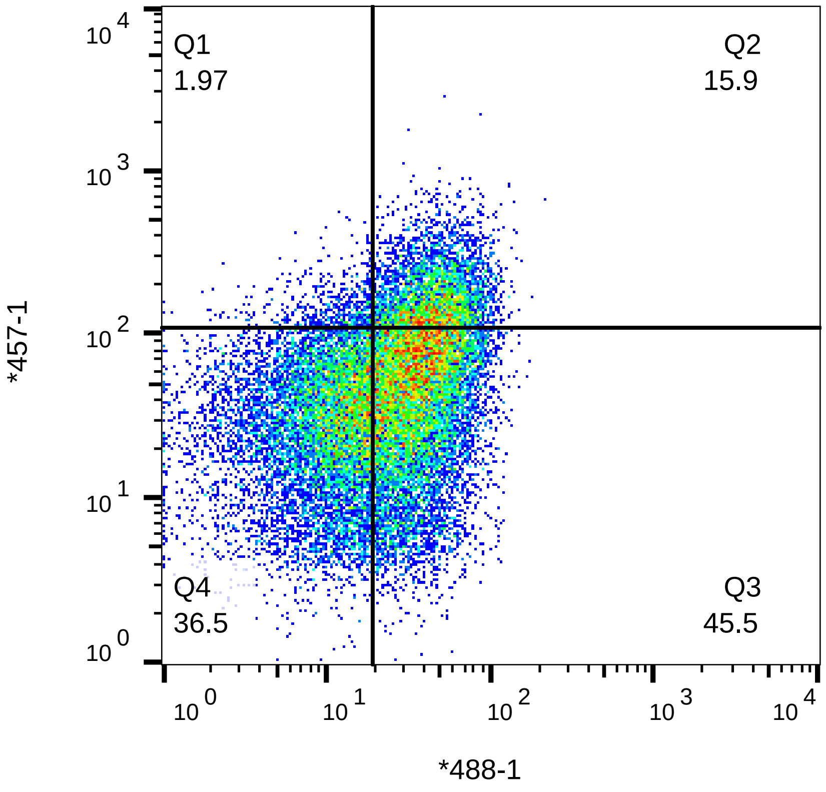

C:\Data\2019\Chrisler\20191220 Shank\195.fcs
All pop
27474

ES2340; SY466; amyE::PcomQX-Ypet (cam); lacA::PaprE-mTurq (erm)

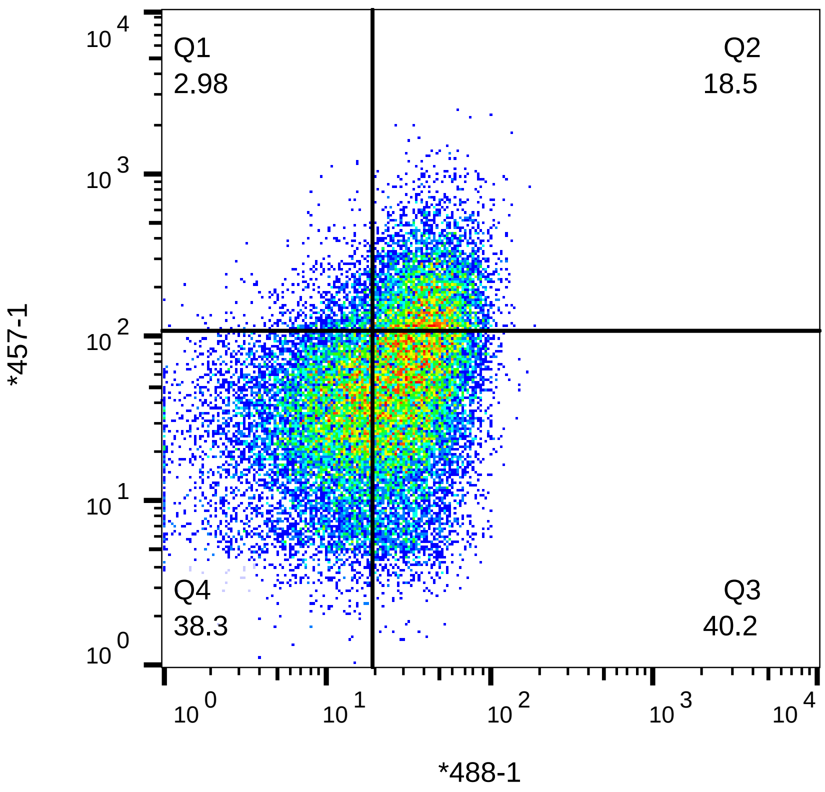

C:\Data\2019\Chrisler\20191220 Shank\196.fcs
All pop
28652

ES2354; SY480; amyE::PskfA-Ypet (cam); lacA::PaprE-mTurq (erm)

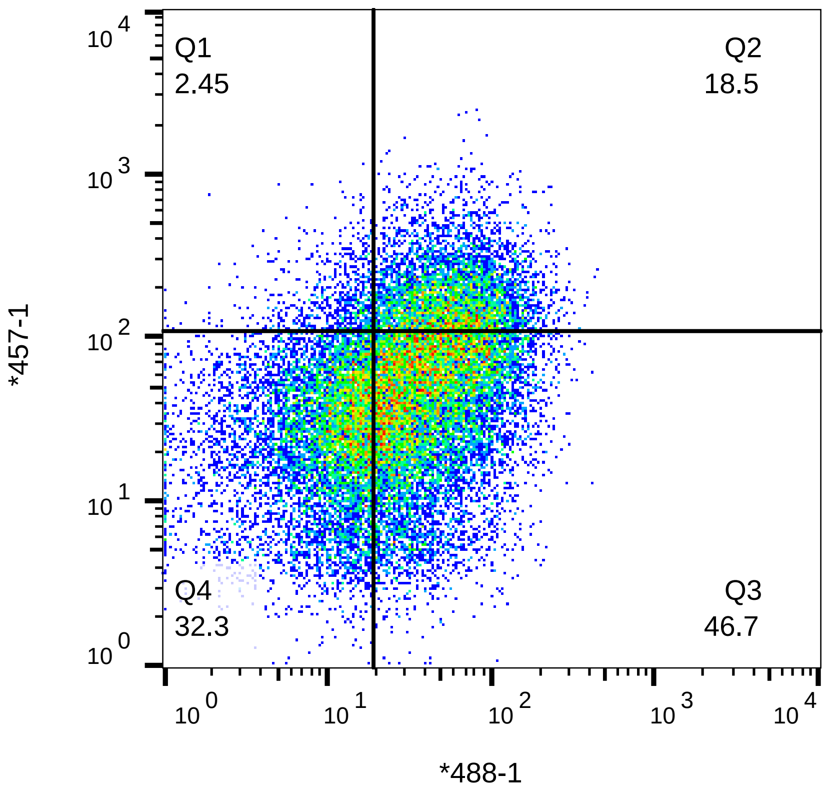

C:\Data\2019\Chrisler\20191220 Shank\210.fcs
All pop
27495

ES2404; SY505; amyE::PcomGA-Ypet (cam); lacA::PtapA-mTurq (erm)

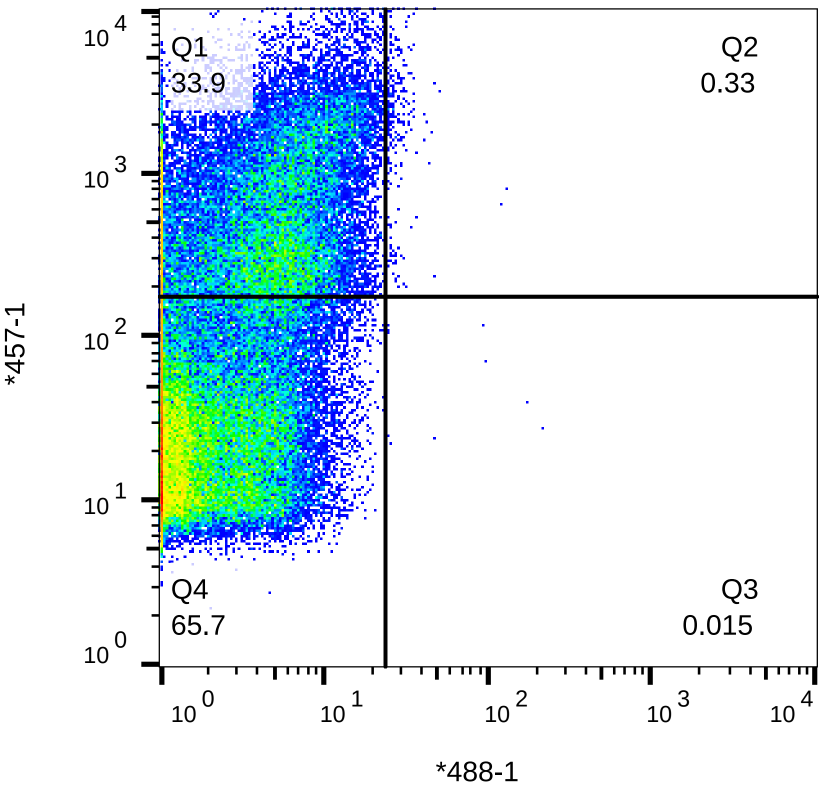

C:\Data\2020\Chrisler\20201007 Shank\SY505.fcs
pop
85564

ES2405; SY506; amyE::PcomGA-Ypet (cam); lacA::PsspB-mTurq (erm)

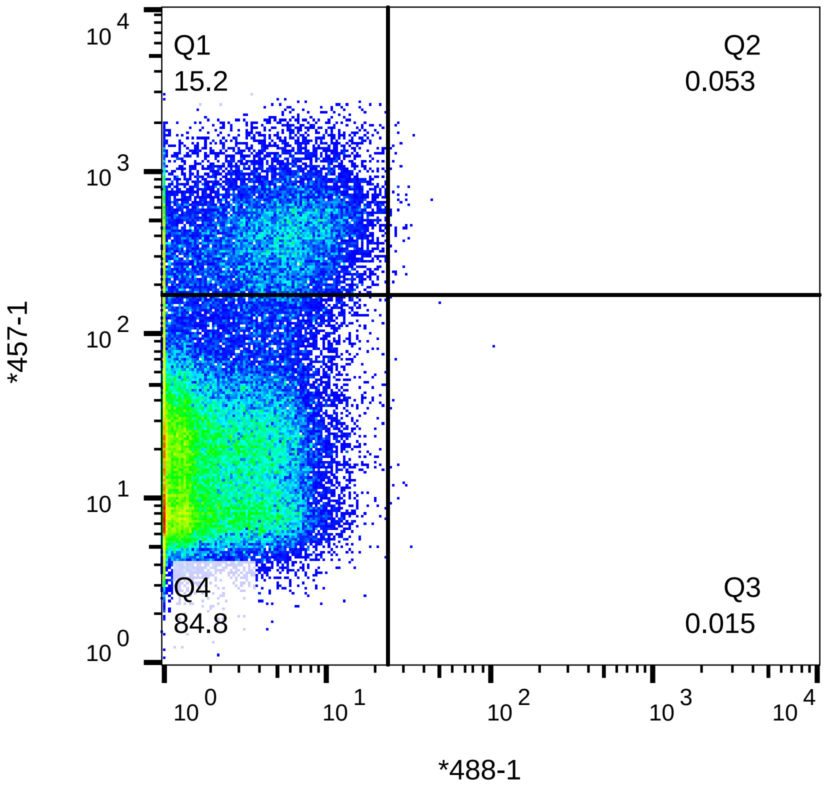

C:\Data\2020\Chrisler\20201007 Shank\SY506.fcs
pop
111020

ES2406; SY507; amyE::PcomGA-Ypet (cam); lacA::Phag-mTurq (erm)

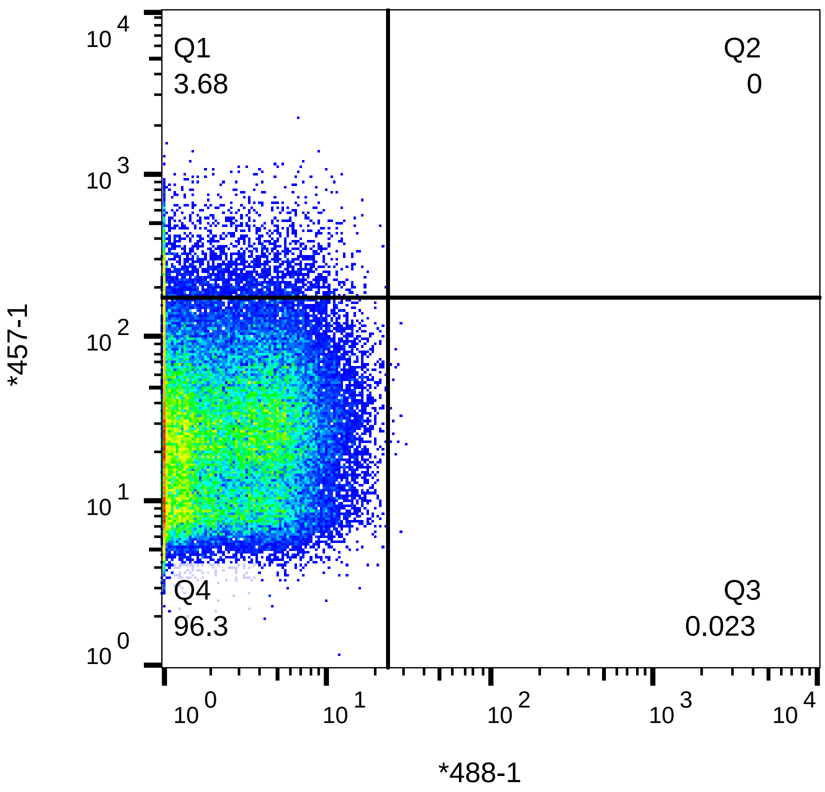

C:\Data\2020\Chrisler\20201007 Shank\SY507.fcs
pop
82775

ES2407; SY508; amyE::PcomGA-Ypet (cam); lacA::PsdpA-mTurq (erm)

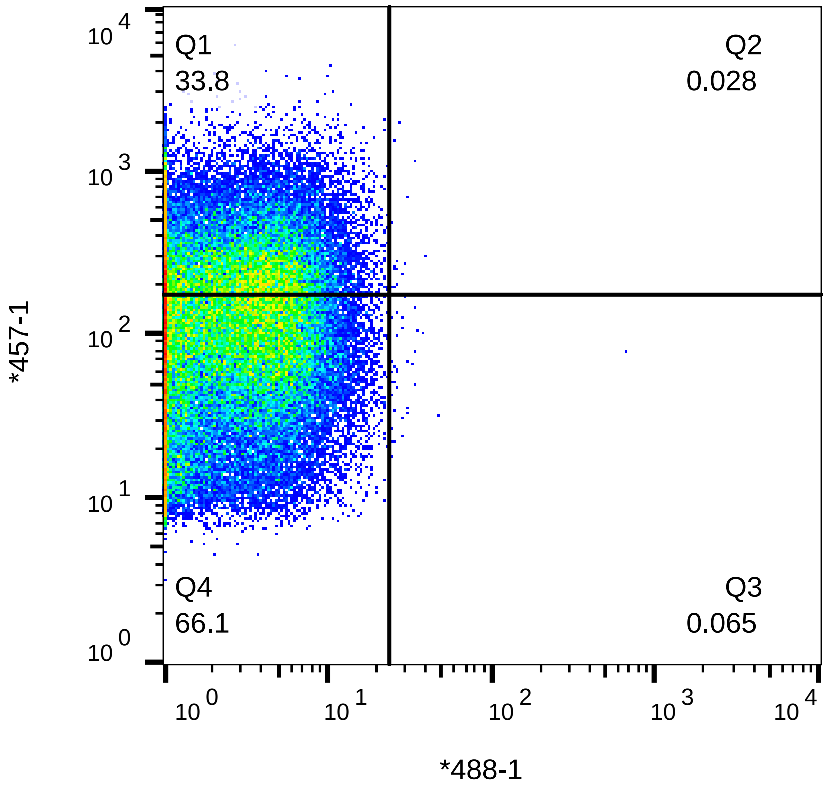

C:\Data\2020\Chrisler\20201007 Shank\SY508.fcs
pop
72721

ES2408; SY509; amyE::PppsA-Ypet (cam); lacA::PtapA-mTurq (erm)

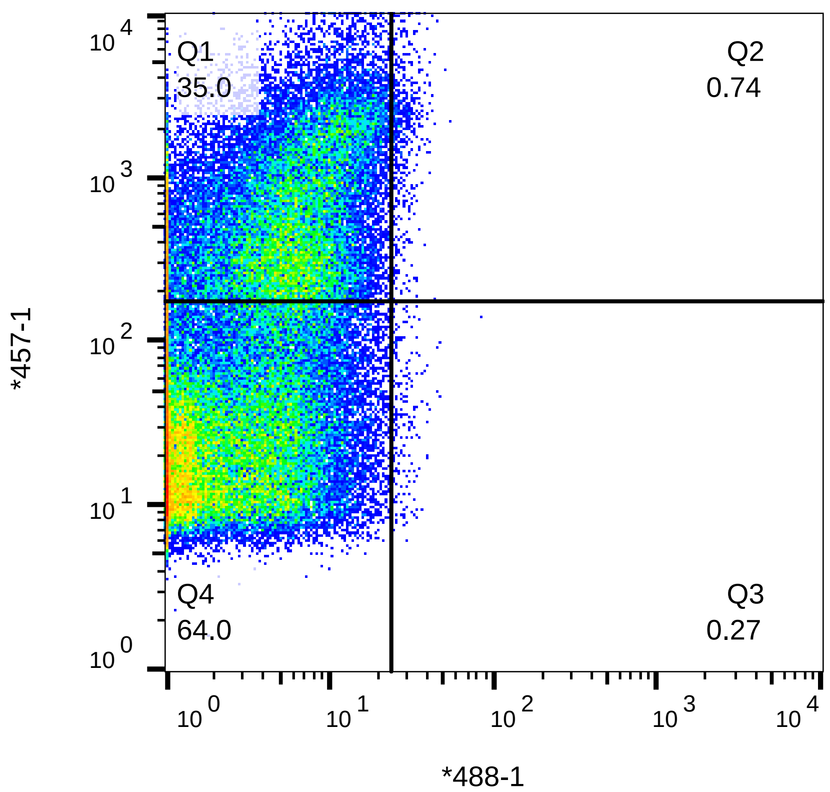

C:\Data\2020\Chrisler\20201007 Shank\SY509.fcs
pop
88389

ES2409; SY510; amyE::PppsA-Ypet (cam); lacA::PsspB-mTurq (erm)

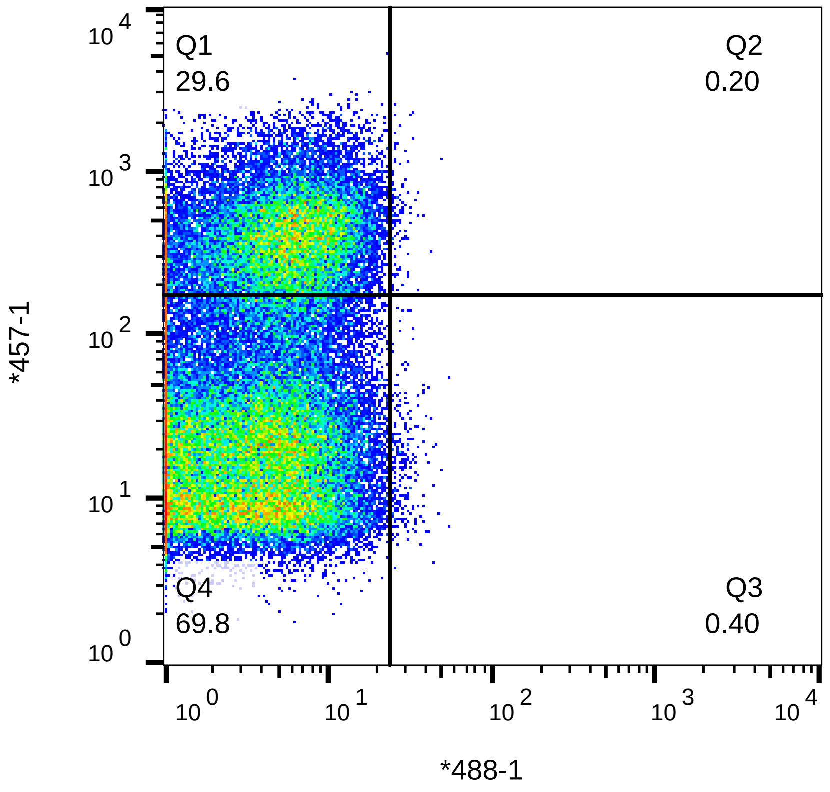

C:\Data\2020\Chrisler\20201007 Shank\SY510.fcs
pop
75085

ES2410; SY511; amyE::PppsA-Ypet (cam); lacA::Phag-mTurq (erm)

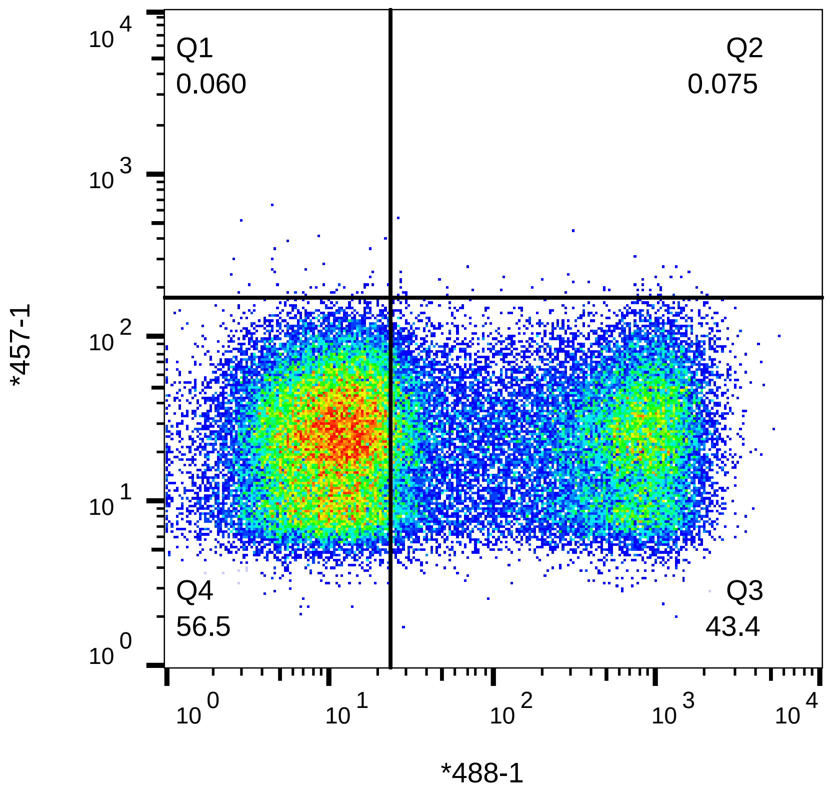

C:\Data\2020\Chrisler\20201007 Shank\SY511.fcs
pop
68326

ES2411; SY512; amyE::PppsA-Ypet (cam); lacA::PsdpA-mTurq (erm)

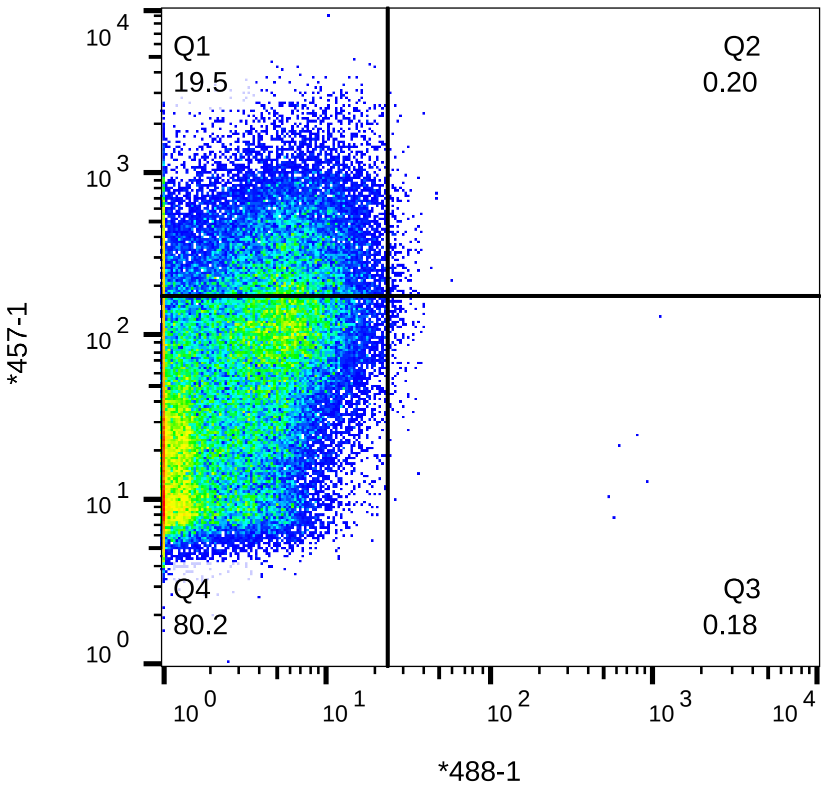

C:\Data\2020\Chrisler\20201007 Shank\SY512.fcs
pop
93998

ES2412; SY513; amyE::PppsA-Ypet (cam); lacA::PcomGA-mTurq (erm)

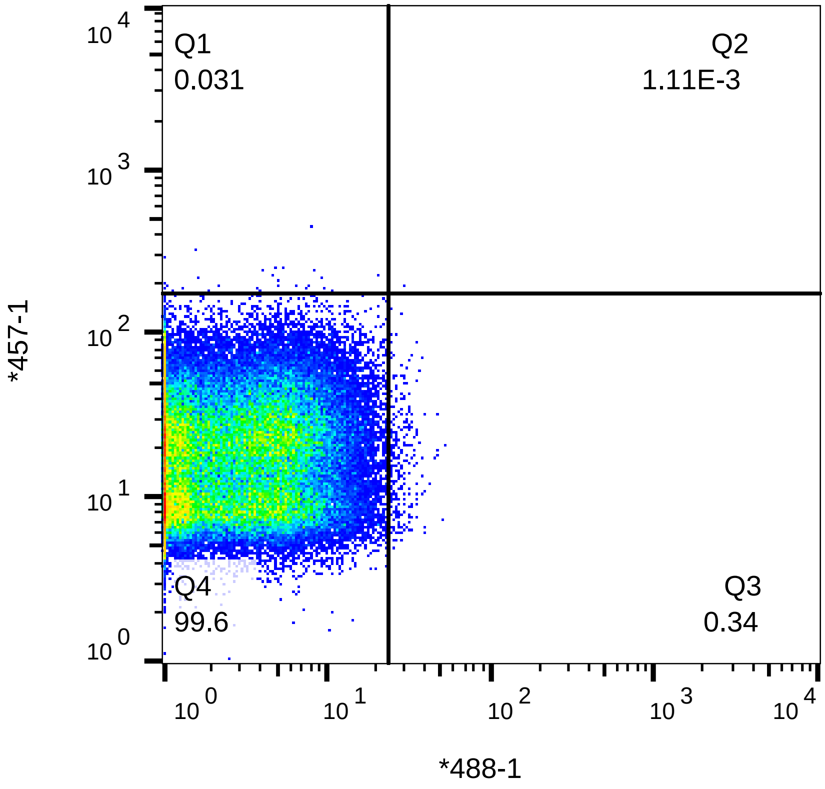

C:\Data\2020\Chrisler\20201007 Shank\SY513.fcs
pop
89884

ES2413; SY514; amyE::PppsA-Ypet (cam); lacA::PpksC-mTurq (erm)

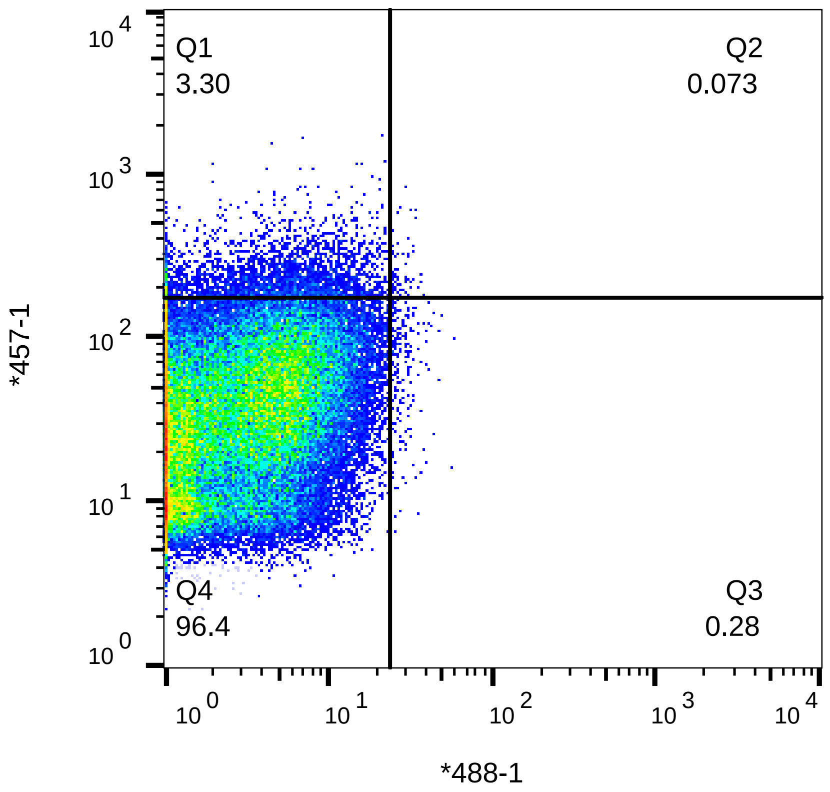

C:\Data\2020\Chrisler\20201007 Shank\SY514.fcs
pop
89457

ES2414; SY515; amyE::PppsA-Ypet (cam); lacA::PdhbA-mTurq (erm)

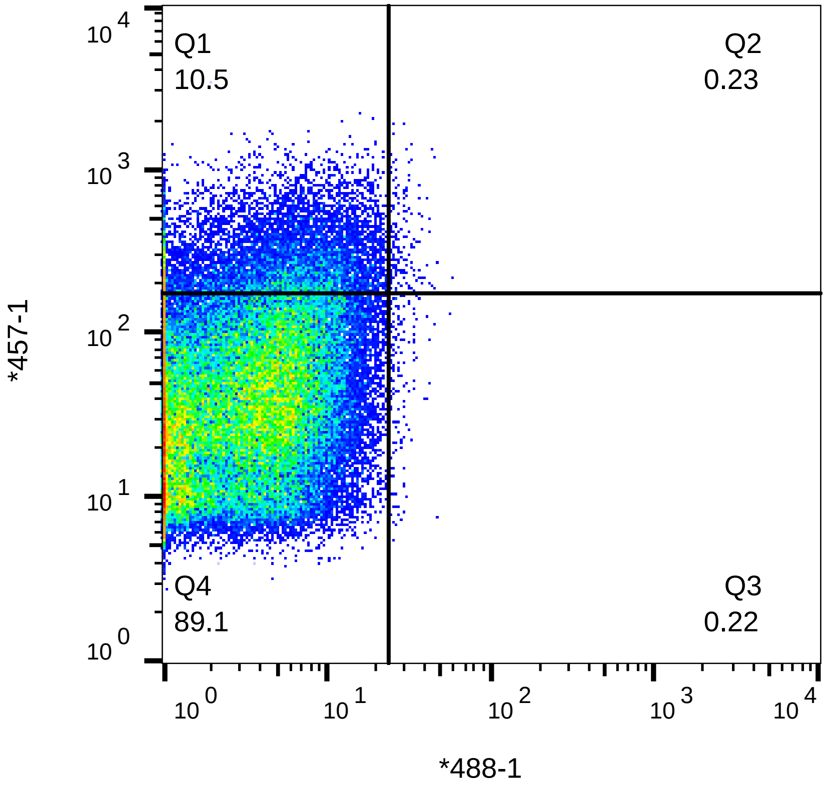

C:\Data\2020\Chrisler\20201007 Shank\SY515.fcs
pop
81477

ES2415; SY516; amyE::PppsA-Ypet (cam); lacA::PbacA-mTurq (erm)

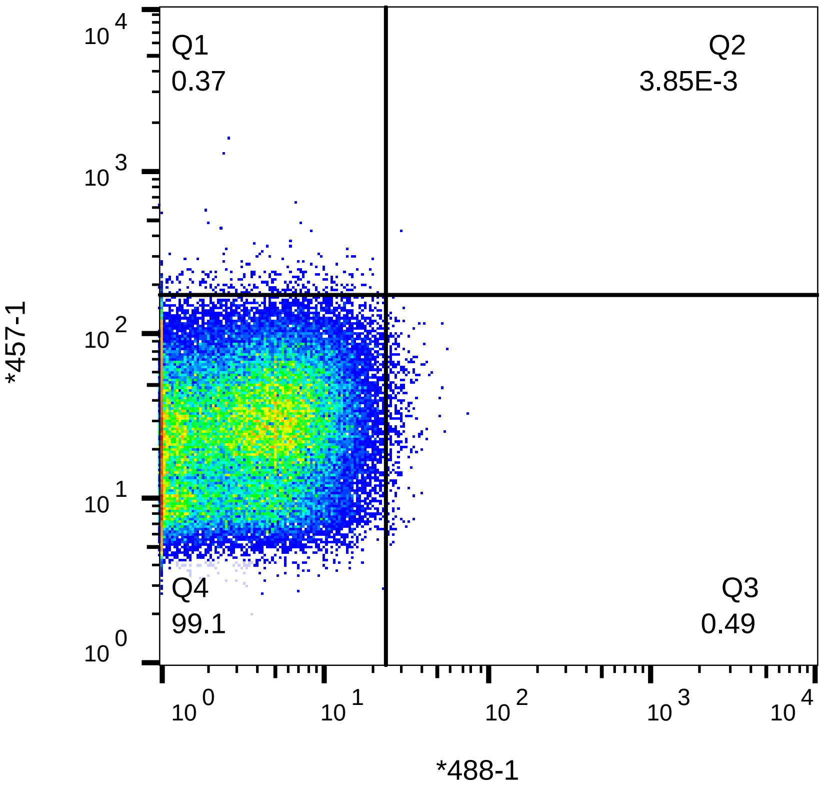

C:\Data\2020\Chrisler\20201007 Shank\SY516.fcs
pop
77909

ES2416; SY517; amyE::PsrfAA-Ypet (cam); lacA::PtapA-mTurq (erm)

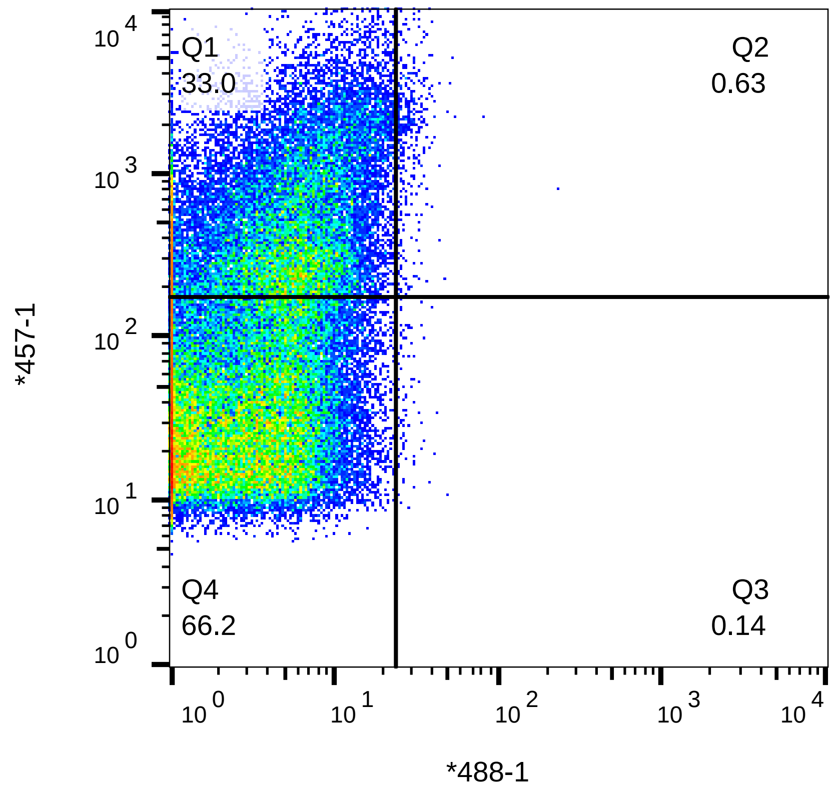

C:\Data\2020\Chrisler\20201007 Shank\SY517.fcs
pop
72424

ES2417; SY518; amyE::PsrfAA-Ypet (cam); lacA::PsspB-mTurq (erm)

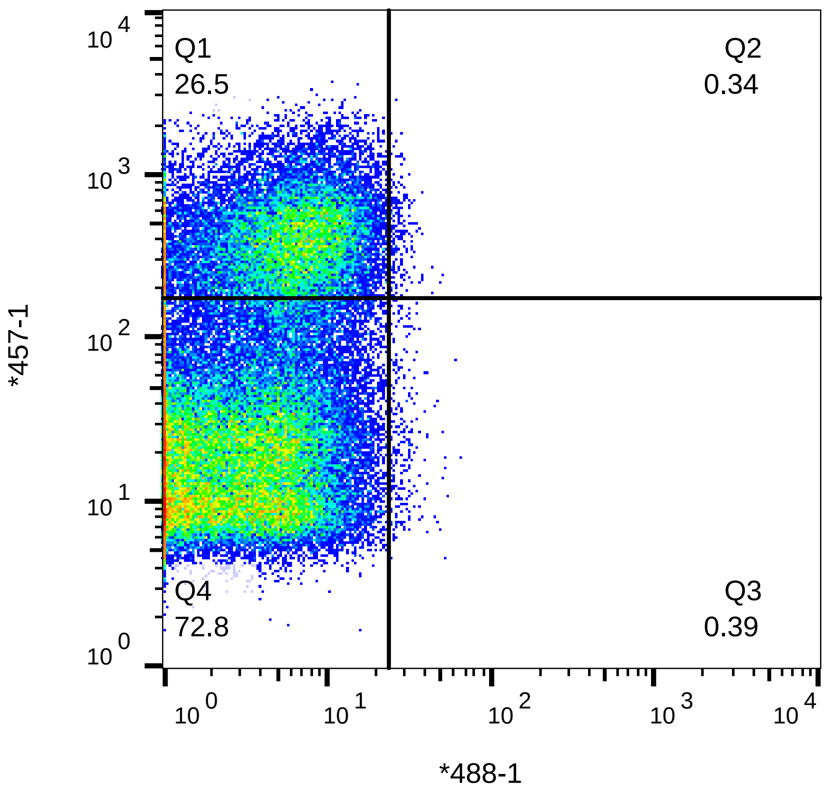

C:\Data\2020\Chrisler\20201007 Shank\SY518.fcs
pop
75325

ES2418; SY519; amyE::PsrfAA-Ypet (cam); lacA::Phag-mTurq (erm)

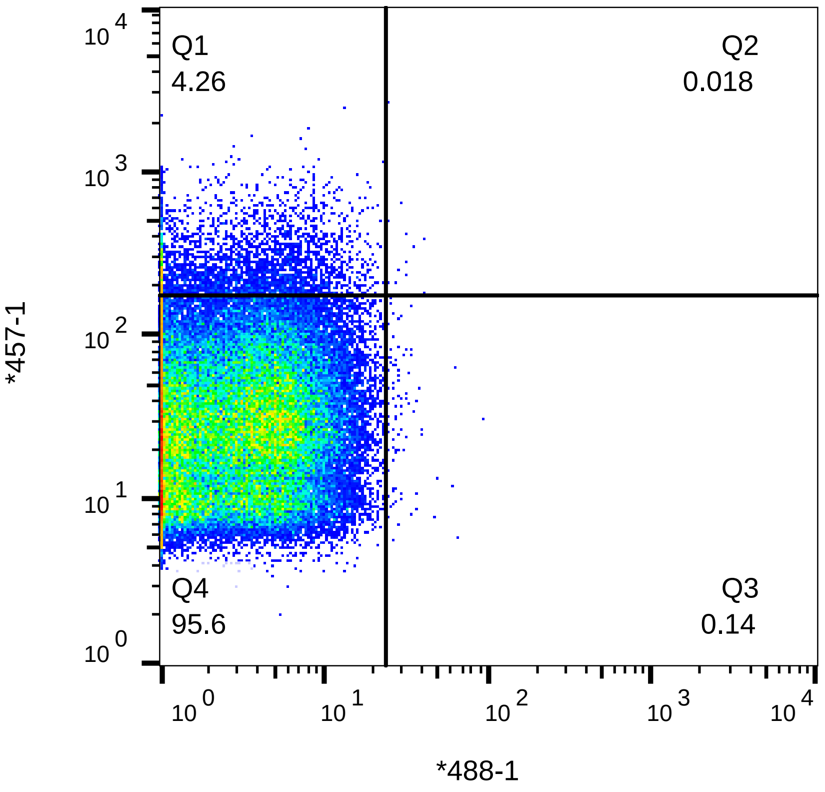

C:\Data\2020\Chrisler\20201007 Shank\SY519.fcs
pop
78866

ES2419; SY520; amyE::PsrfAA-Ypet (cam); lacA::PsdpA-mTurq (erm)

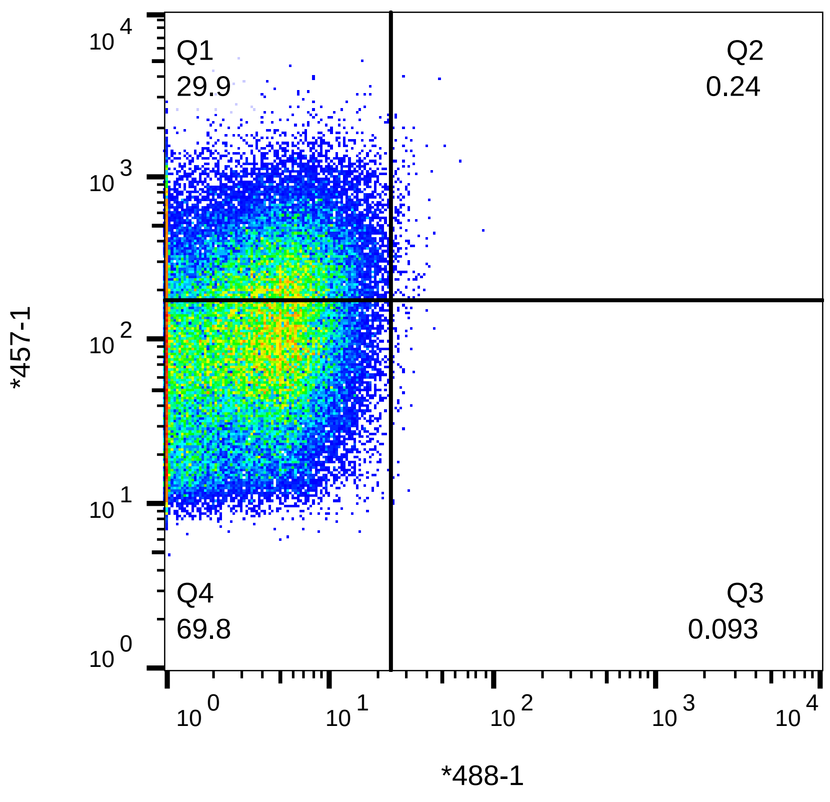

C:\Data\2020\Chrisler\20201007 Shank\SY520.fcs
pop
76170

ES2420; SY521; amyE::PsrfAA-Ypet (cam); lacA::PcomGA-mTurq (erm)

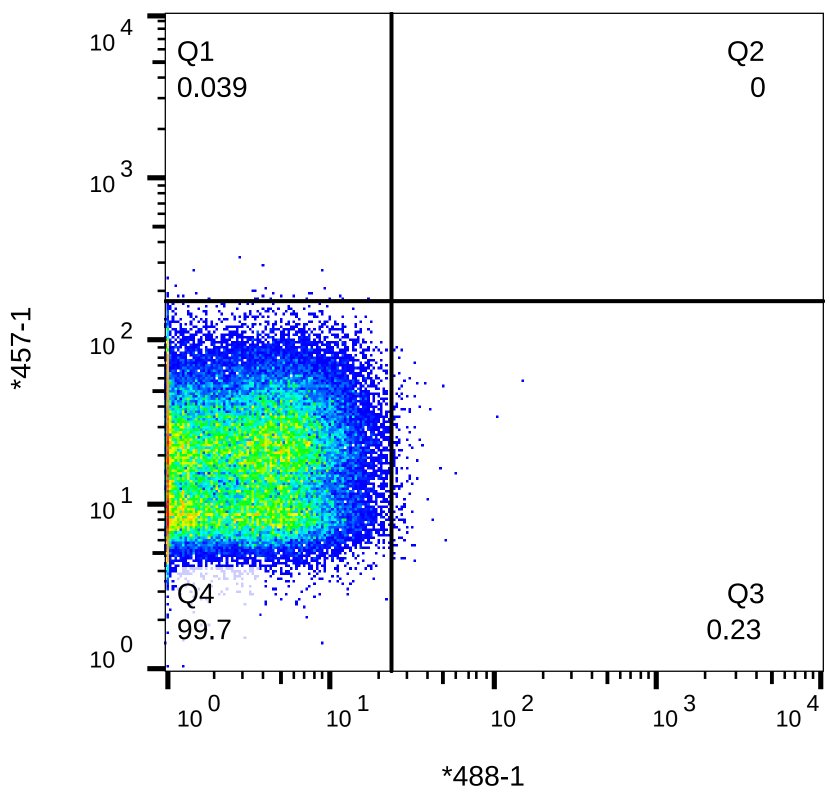

C:\Data\2020\Chrisler\20201007 Shank\SY521.fcs
pop
77373

ES2421; SY522; amyE::PsrfAA-Ypet (cam); lacA::PpksC-mTurq (erm)

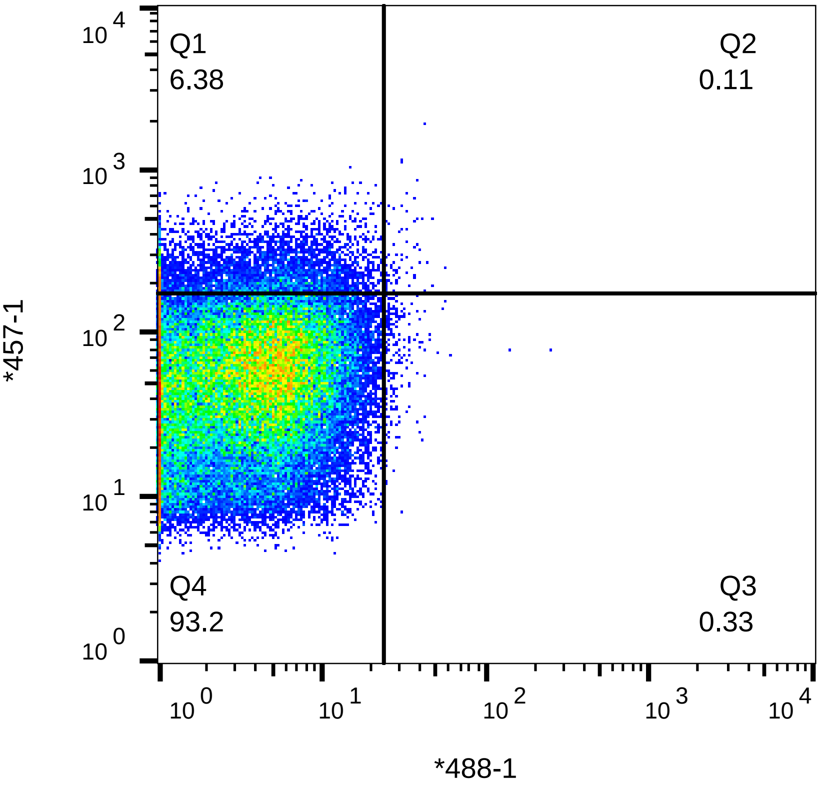

C:\Data\2020\Chrisler\20201007 Shank\SY522.fcs
pop
71911

ES2422; SY523; amyE::PsrfAA-Ypet (cam); lacA::PdhbA-mTurq (erm)

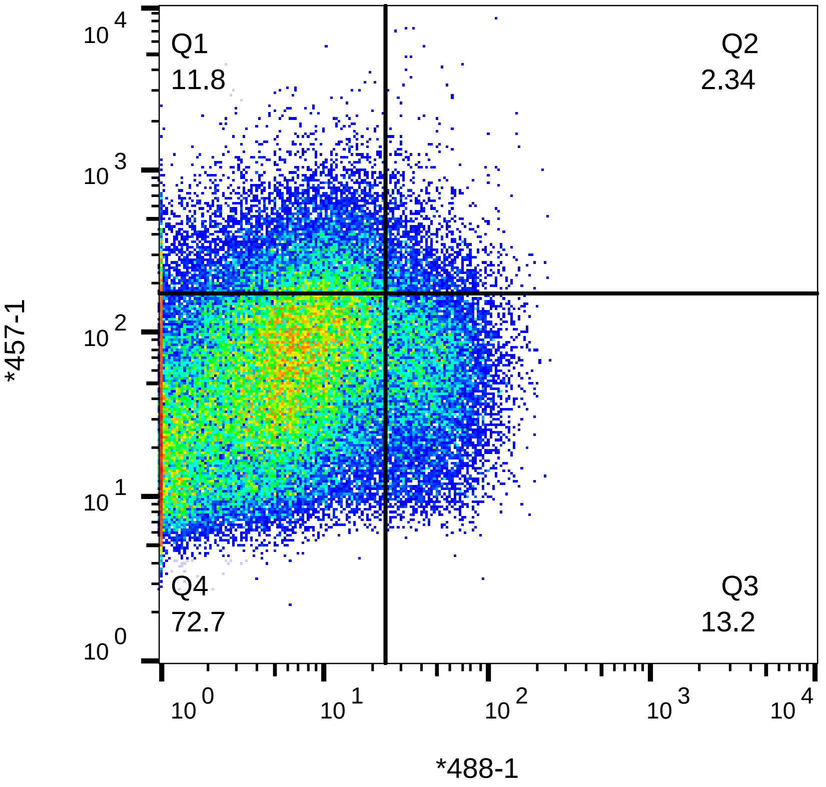

C:\Data\2020\Chrisler\20201007 Shank\SY523.fcs
pop
80978

ES2423; SY524; amyE::PsrfAA-Ypet (cam); lacA::PbacA-mTurq (erm)

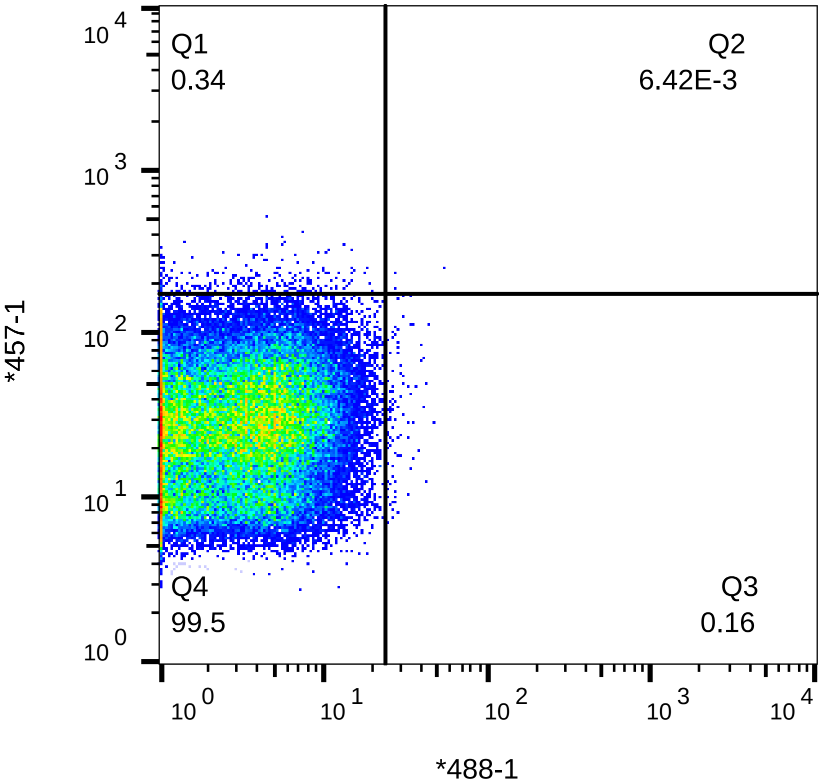

C:\Data\2020\Chrisler\20201007 Shank\SY524.fcs
pop
77861

ES2424; SY525; amyE::PsrfAA-Ypet (cam); lacA::PppsA-mTurq (erm)

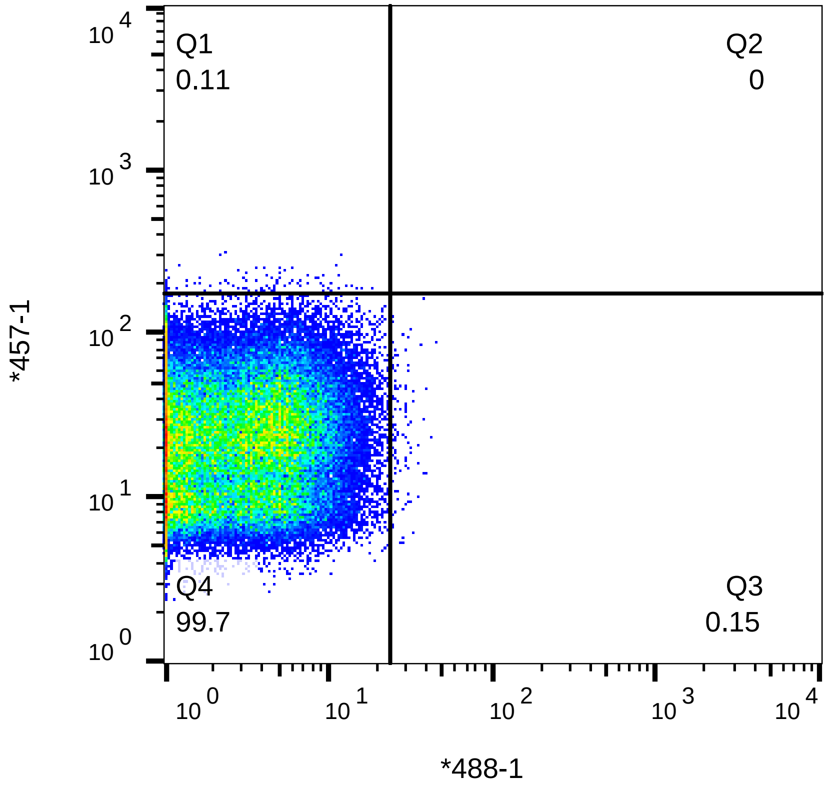

C:\Data\2020\Chrisler\20201007 Shank\SY525.fcs
pop
80407

ES3 = NCIB3610 = WT

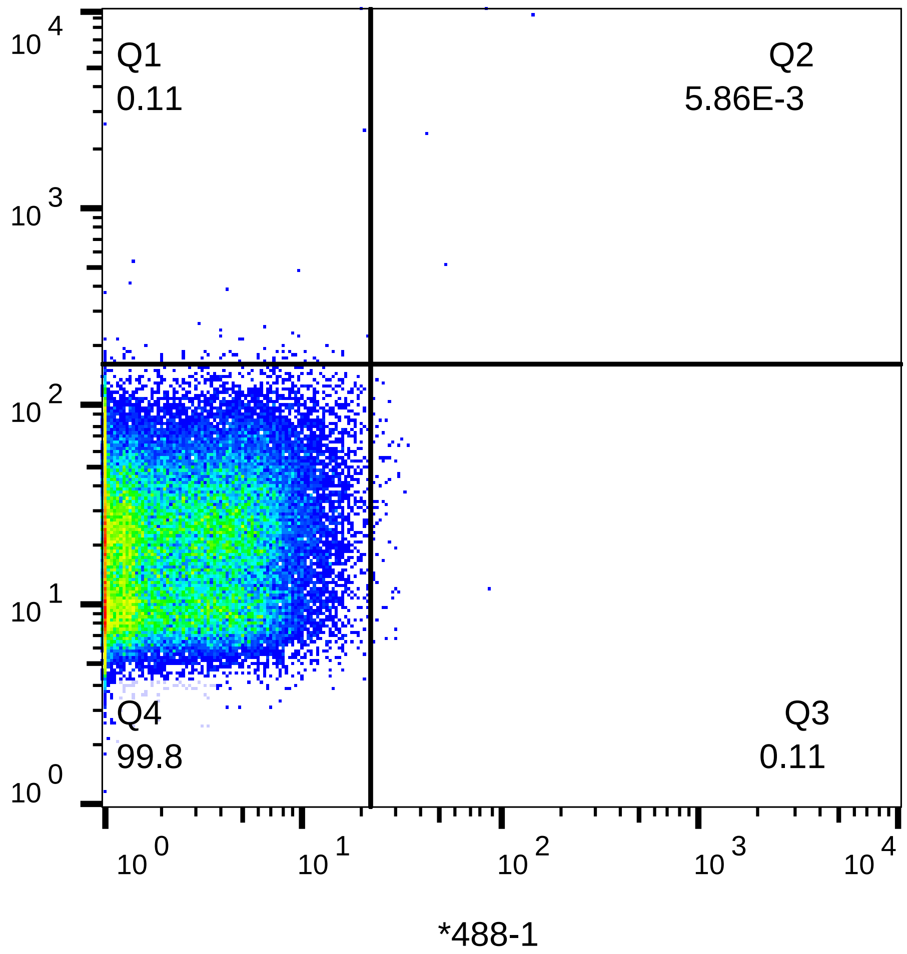

C:\Data\2020\Chrisler\20201007 Shank\SY1.fcs
pop
68282

ES2586; SY554; amyE::PspacC-Ypet (cam); lacA::PspacC-mTurq (erm)

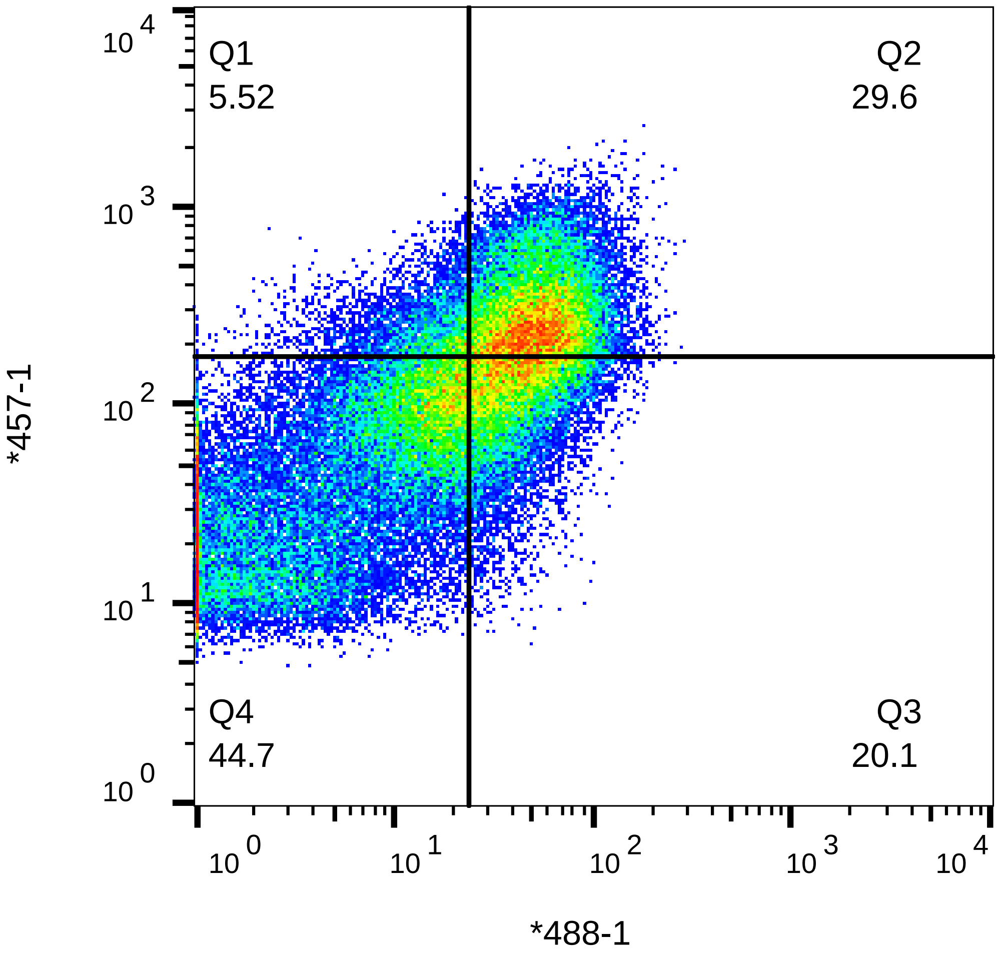

C:\Data\2020\Chrisler\20201007 Shank\SY554.fcs
pop
76217

ES2587; SY555; amyE::Phag-Ypet (cam); lacA::Phag-mTurq (erm)

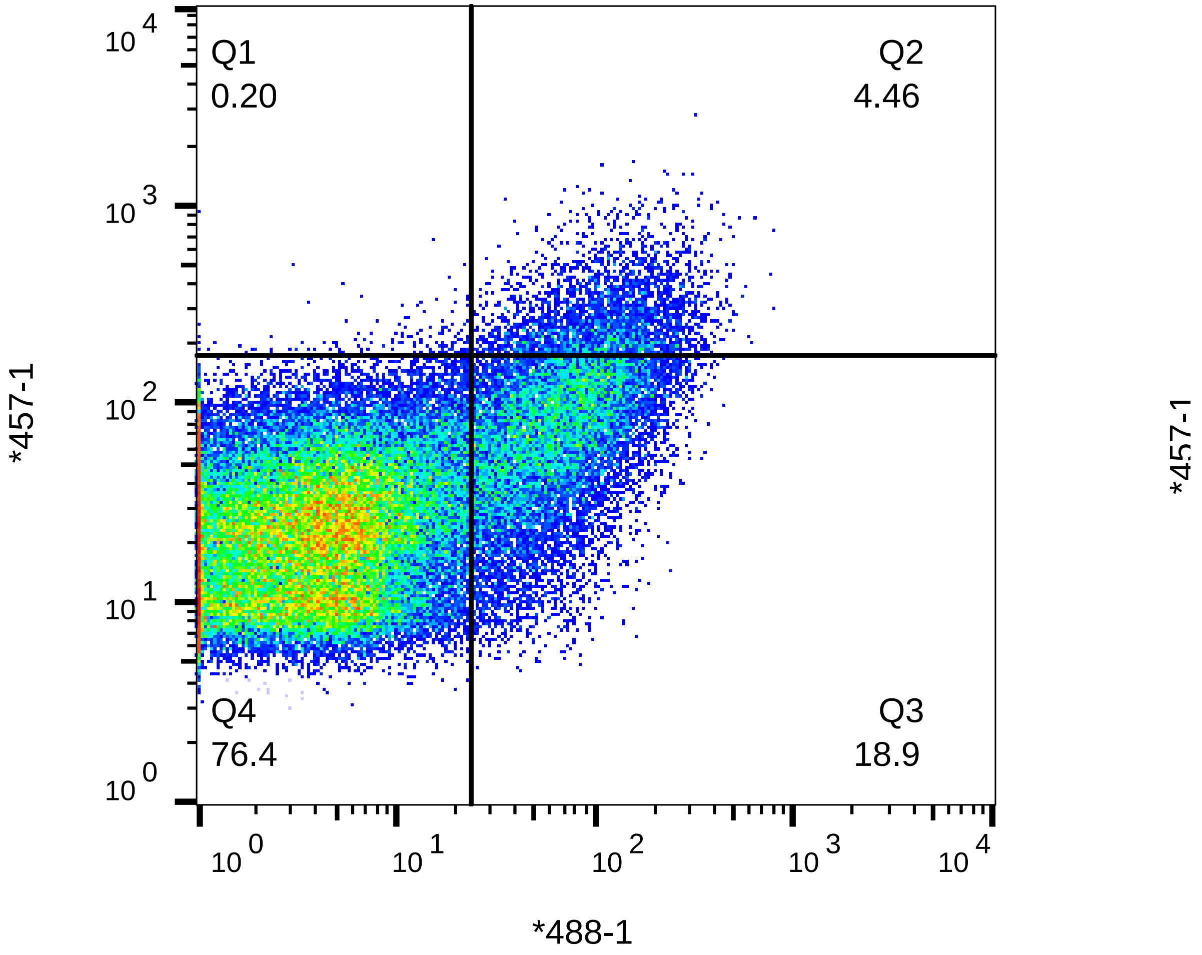

C:\Data\2020\Chrisler\20201007 Shank\SY555.fcs
pop
72919

ES2588; SY556; amyE::PtapA-Ypet (cam); lacA::PtapA-mTurq (erm)

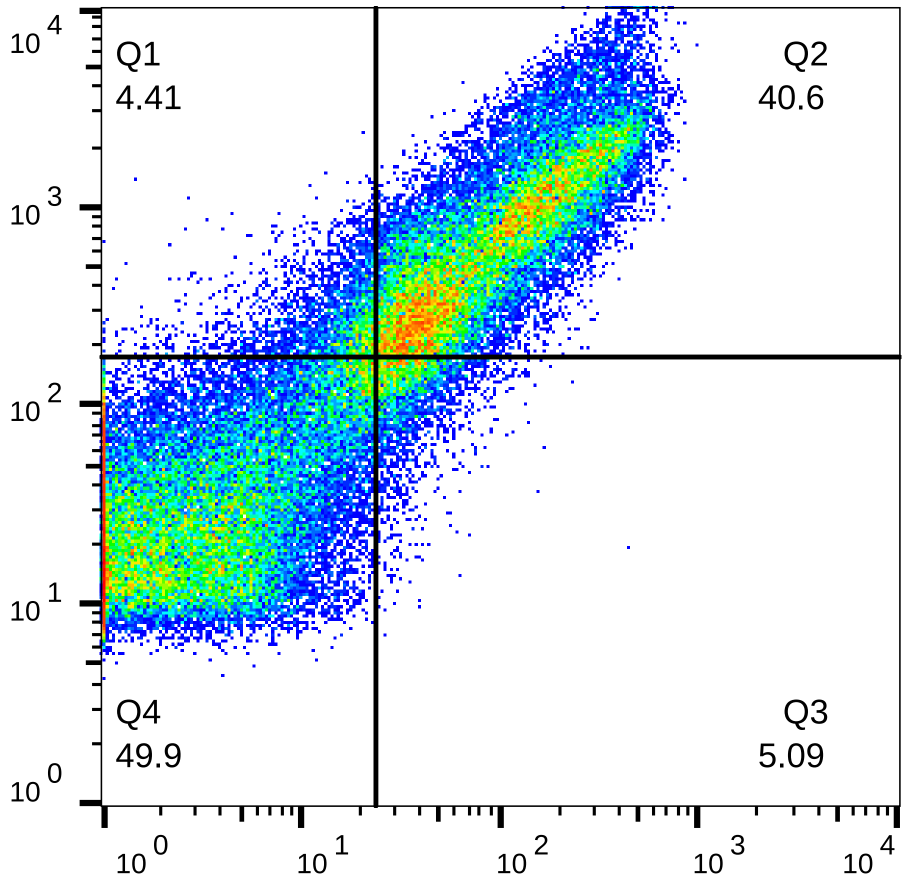

C:\Data\2020\Chrisler\20201007 Shank\SY556.fcs
pop
76057

ES2589; SY557; amyE::PsspB-Ypet (cam); lacA::PsspB-mTurq (erm)

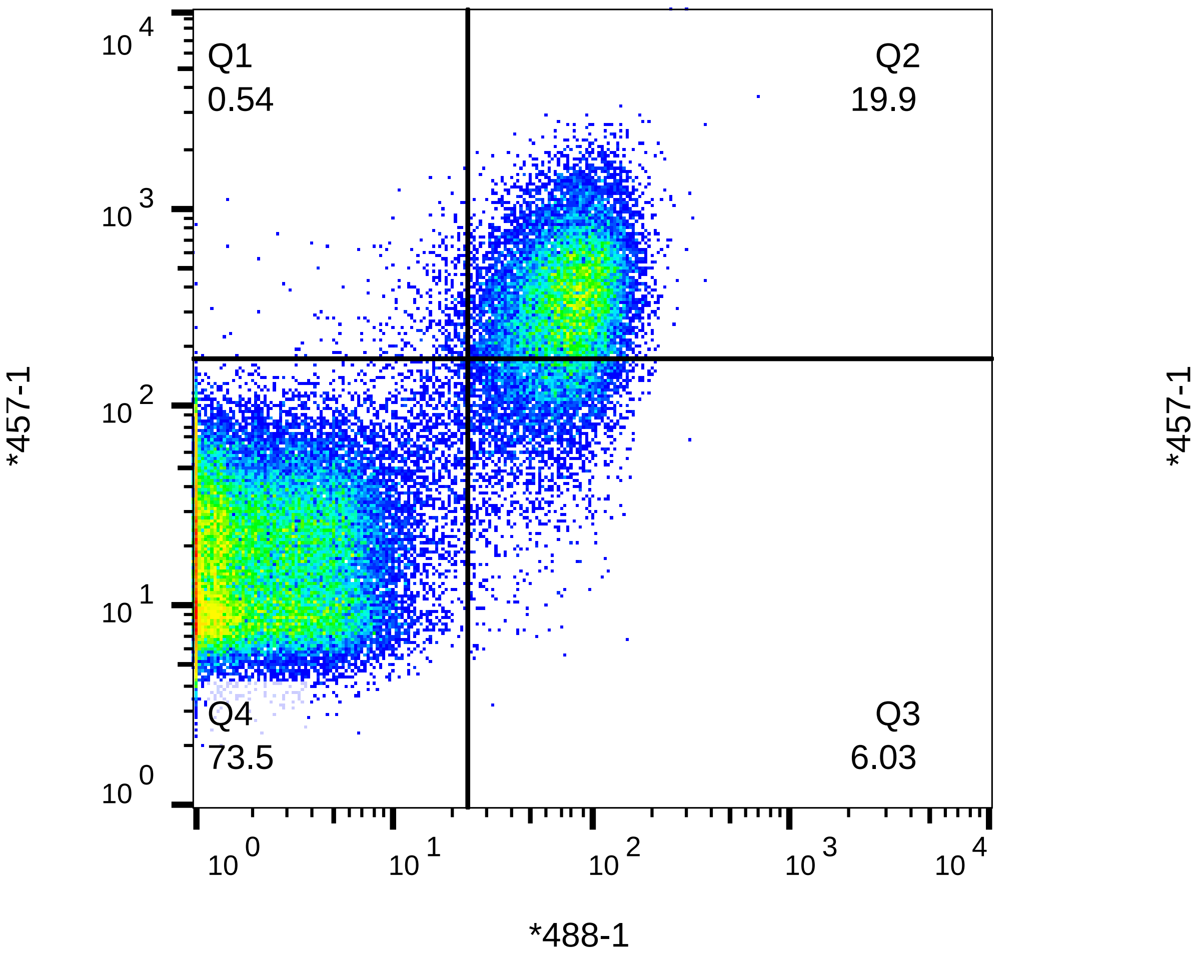

C:\Data\2020\Chrisler\20201007 Shank\SY557.fcs
pop
73906

ES2590; SY558; amyE::PsdpA-Ypet (cam); lacA::PsdpA-mTurq (erm)

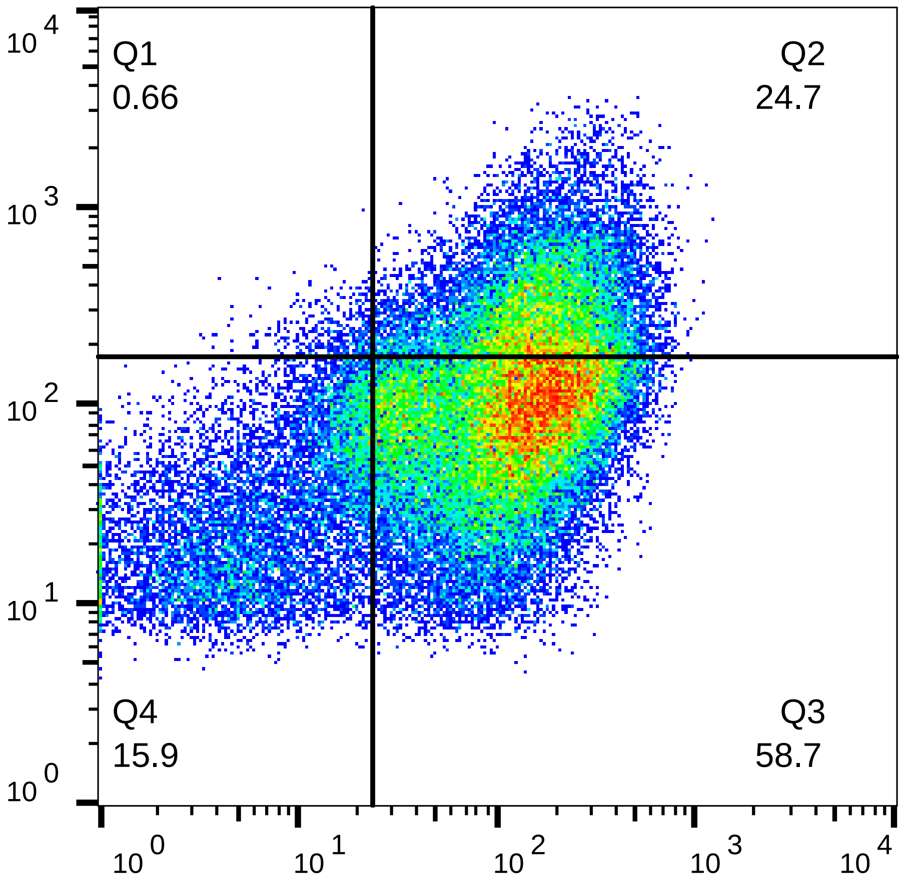

C:\Data\2020\Chrisler\20201007 Shank\SY558.fcs
pop
67127

ES2591; SY559; amyE::PcomGA-Ypet (cam); lacA::PcomGA-mTurq (erm)

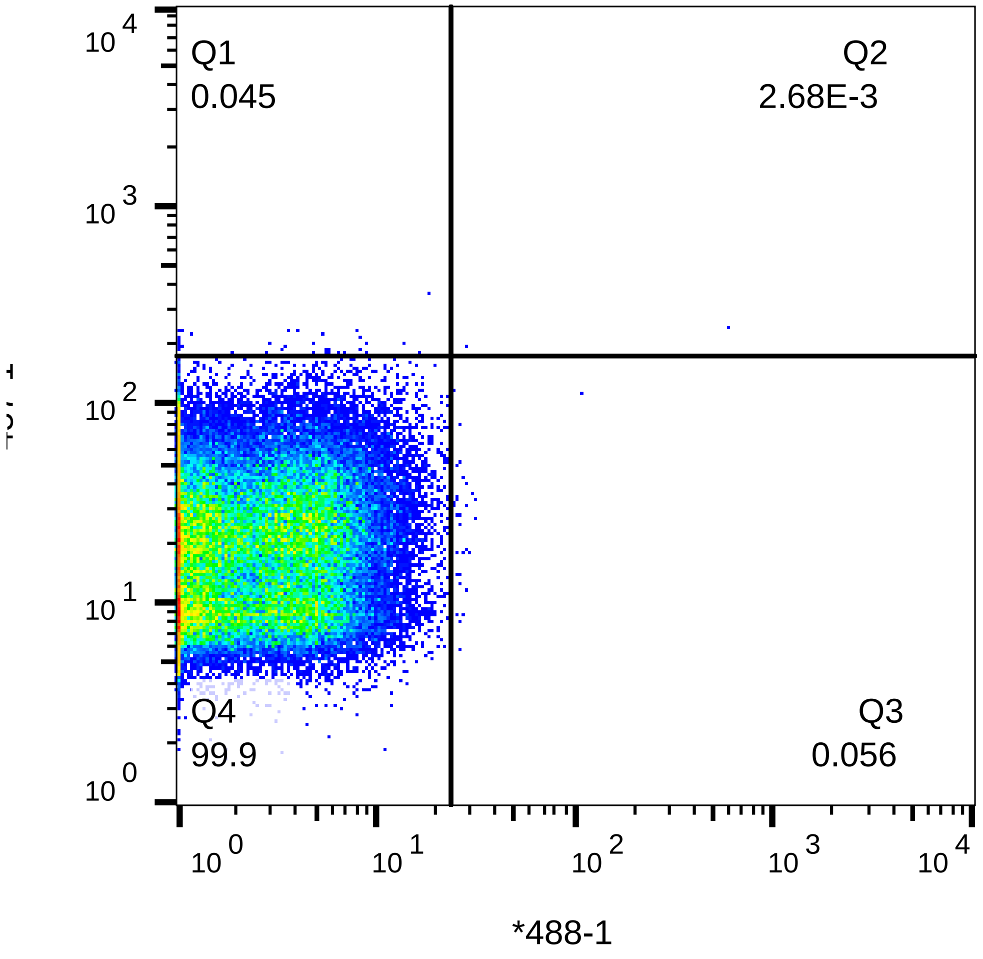

C:\Data\2020\Chrisler\20201007 Shank\SY559.fcs
pop
74734

ES2592; SY560; amyE::PpksC-Ypet (cam); lacA::PpksC-mTurq (erm)

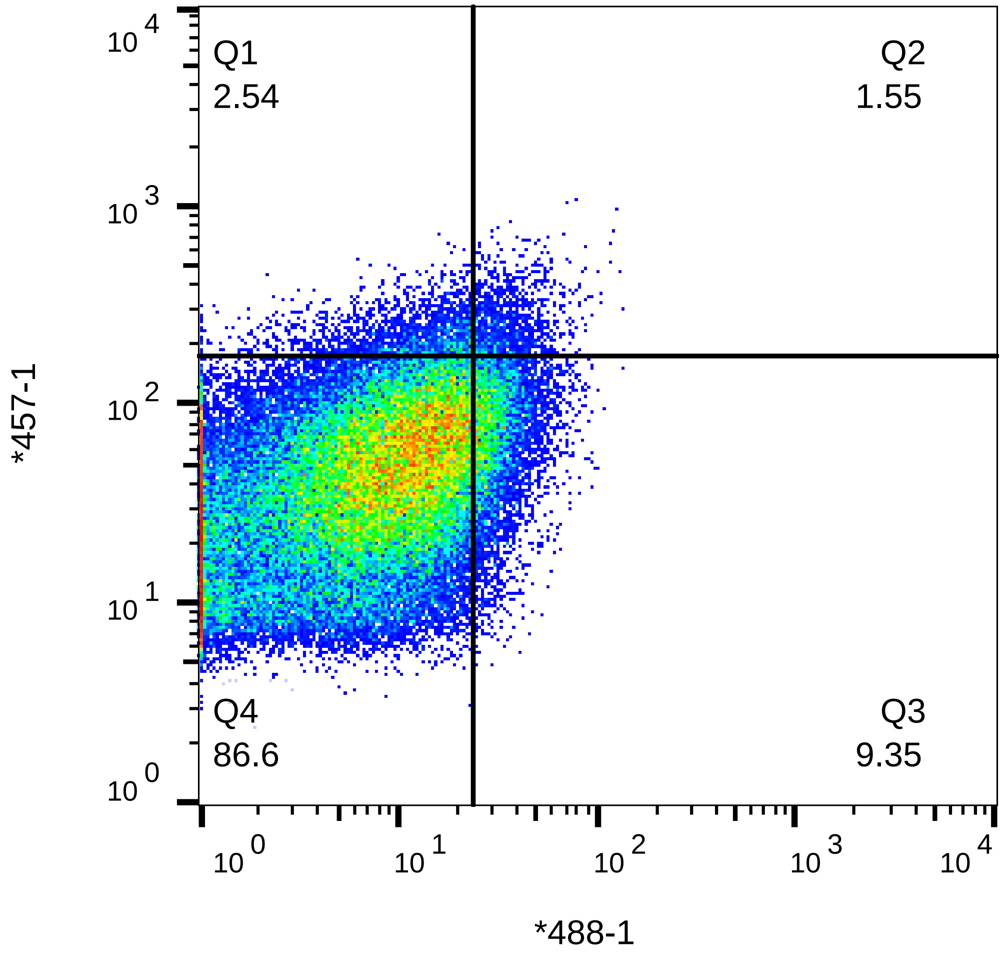

C:\Data\2020\Chrisler\20201007 Shank\SY560.fcs
pop
77193

ES2593; SY561; amyE::PbacA-Ypet (cam); lacA::PbacA-mTurq (erm)

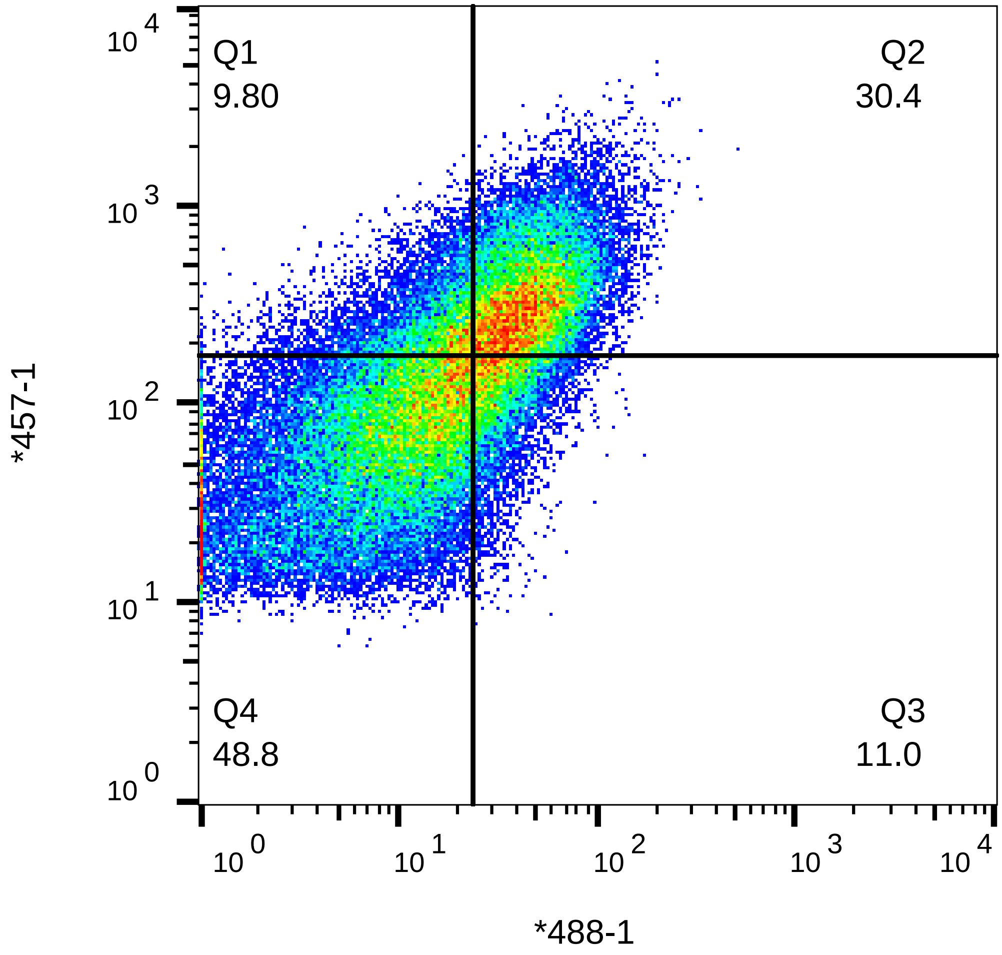

C:\Data\2020\Chrisler\20201007 Shank\SY561.fcs
pop
72473

ES2594; SY562; amyE::PppsA-Ypet (cam); lacA::PppsA-mTurq (erm)

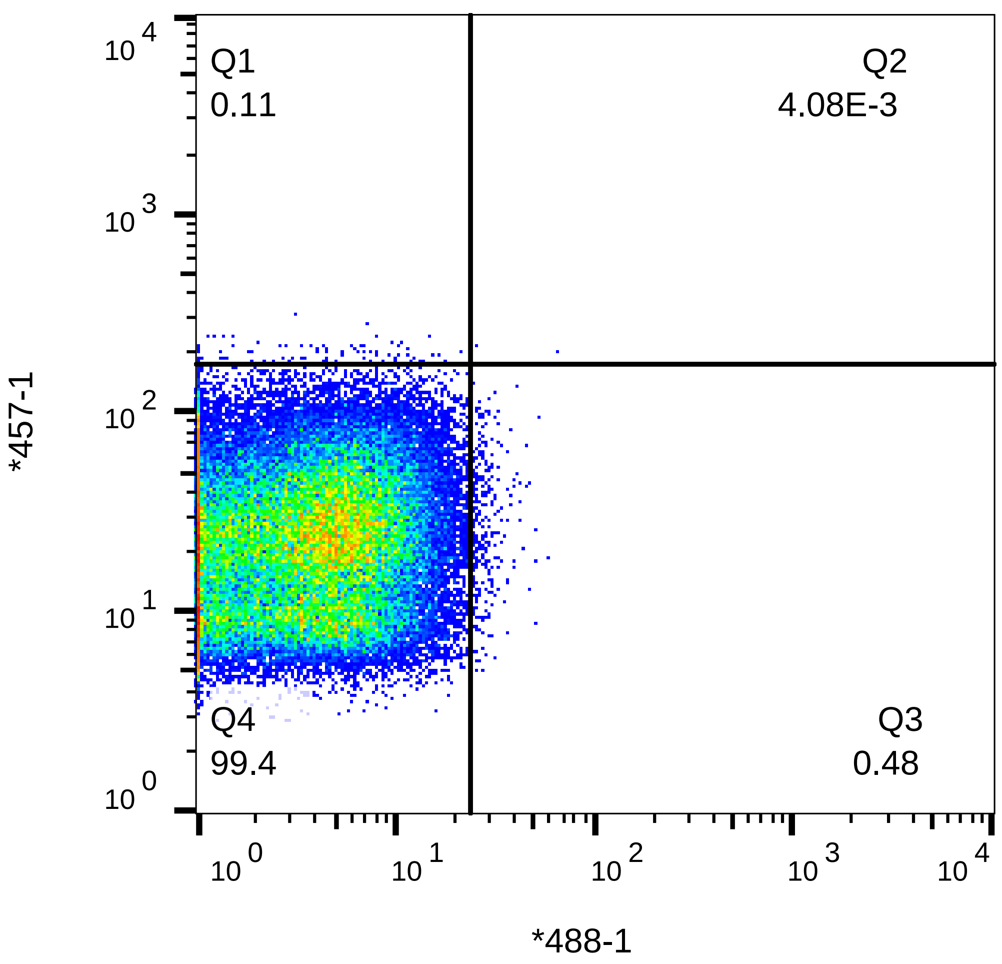

C:\Data\2020\Chrisler\20201007 Shank\SY562.fcs
pop
73573

ES2595; SY563; amyE::PsrfAA-Ypet (cam); lacA::PsrfAA-mTurq (erm)

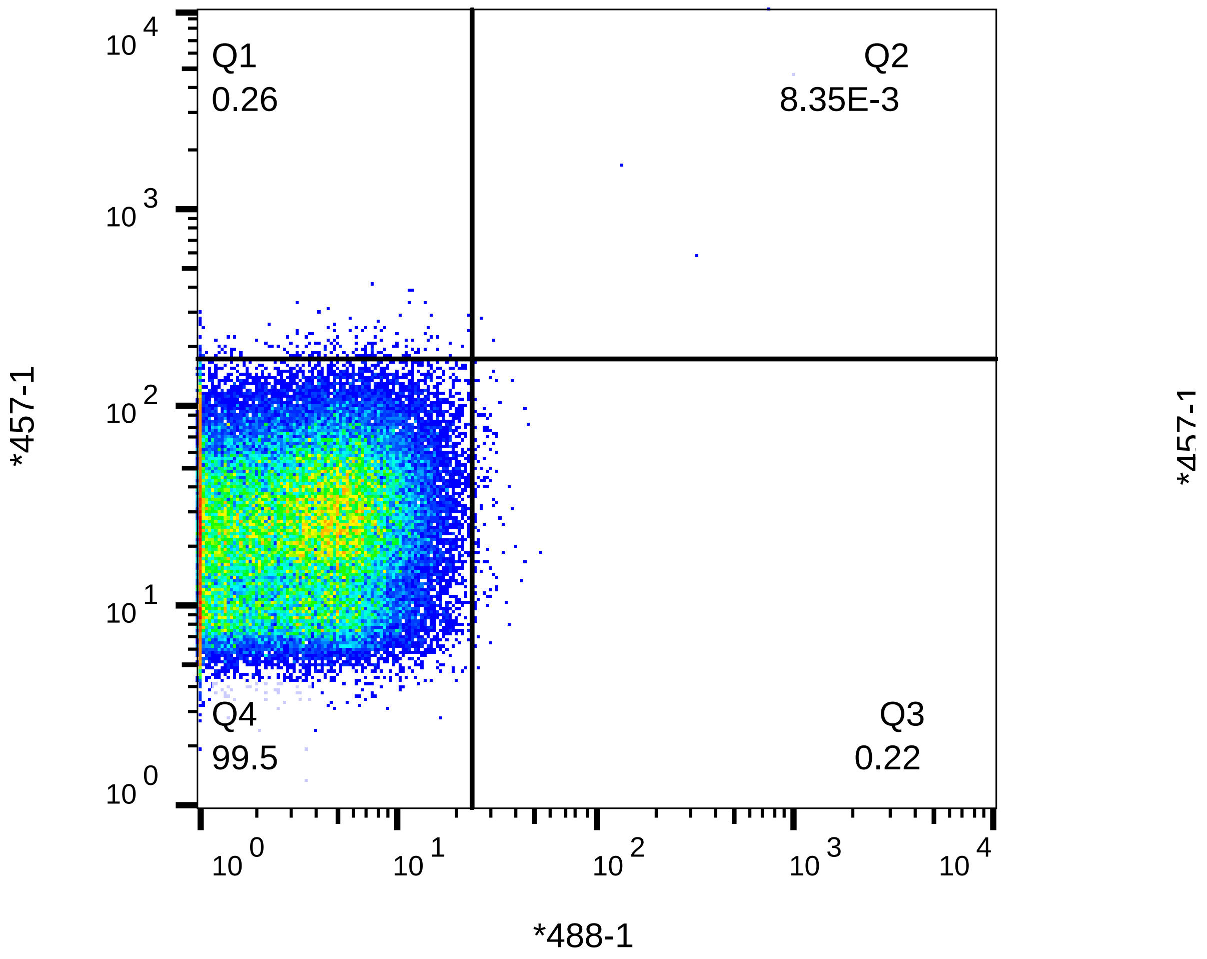

C:\Data\2020\Chrisler\20201007 Shank\SY563.fcs
pop
71848

ES2596; SY564; amyE::PsboA-Ypet (cam); lacA::PsboA-mTurq (erm)

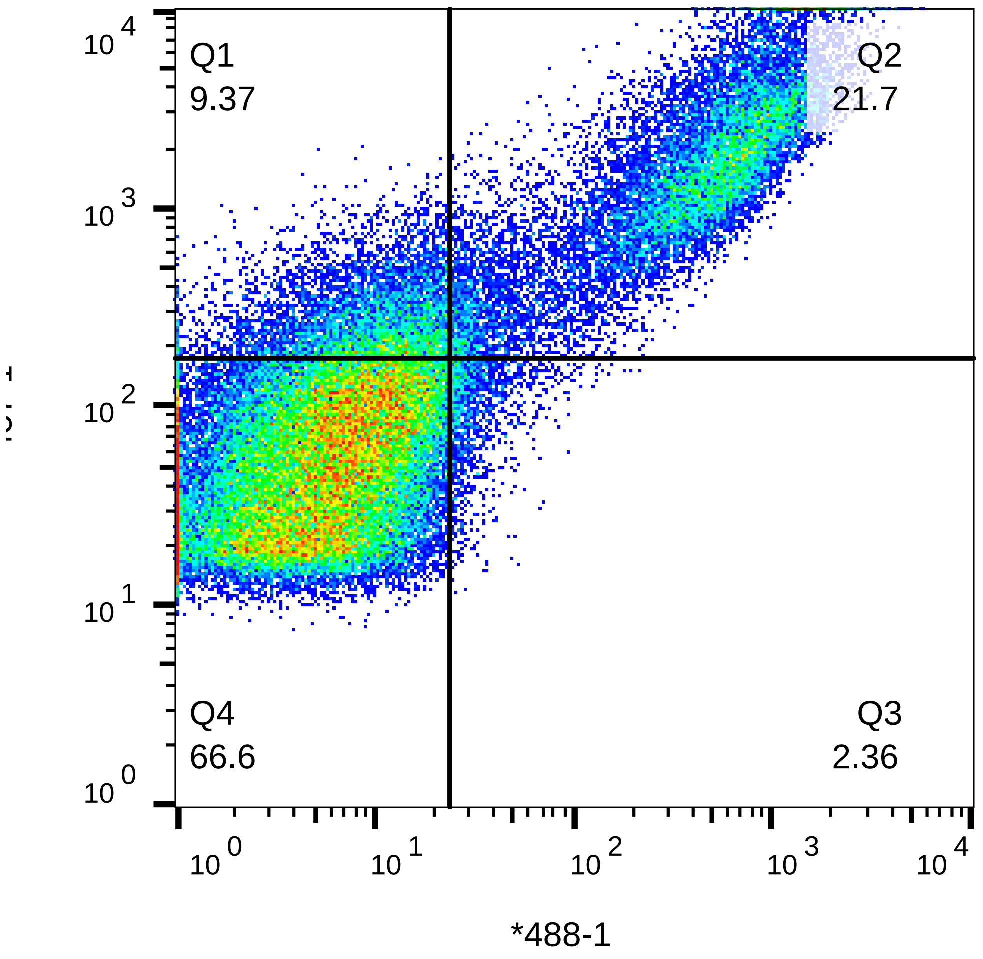

C:\Data\2020\Chrisler\20201007 Shank\SY564.fcs
pop
76343

ES2597; SY565; amyE::PcomQX-Ypet (cam); lacA::PcomQX-mTurq (erm)

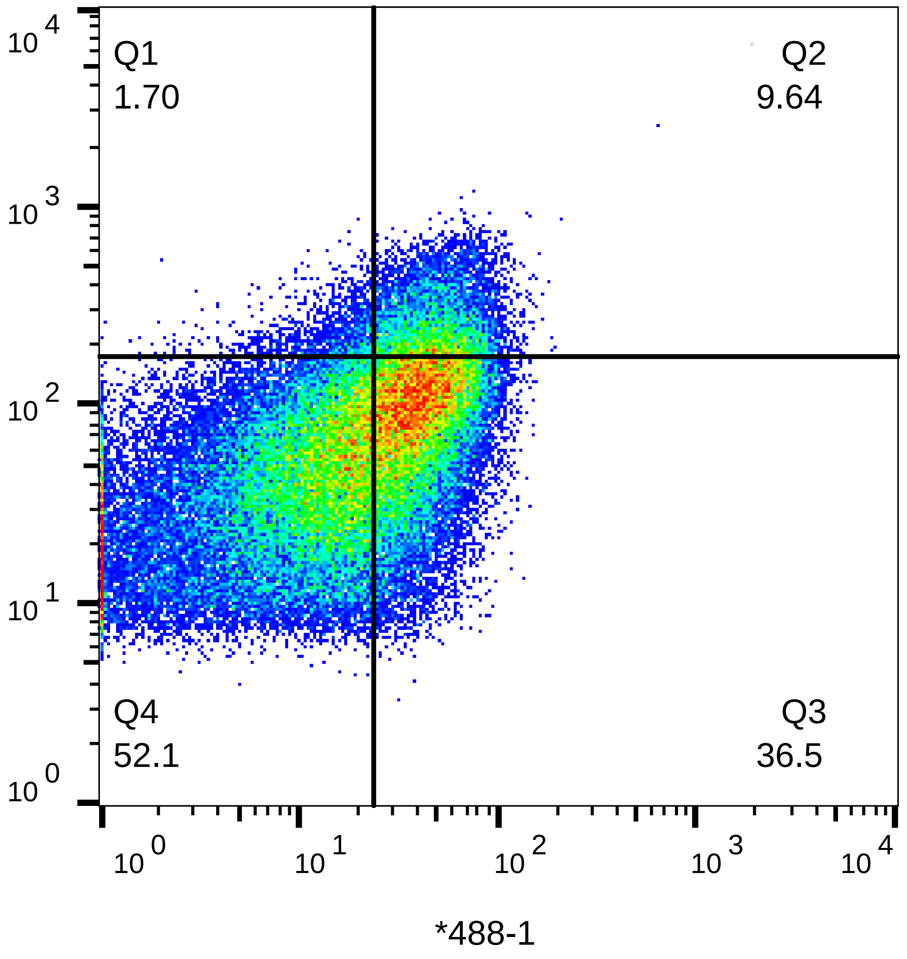

C:\Data\2020\Chrisler\20201007 Shank\SY565.fcs
pop
74036

ES2601; SY569; amyE::PskfA-Ypet (cam); lacA::PskfA-mTurq (erm)

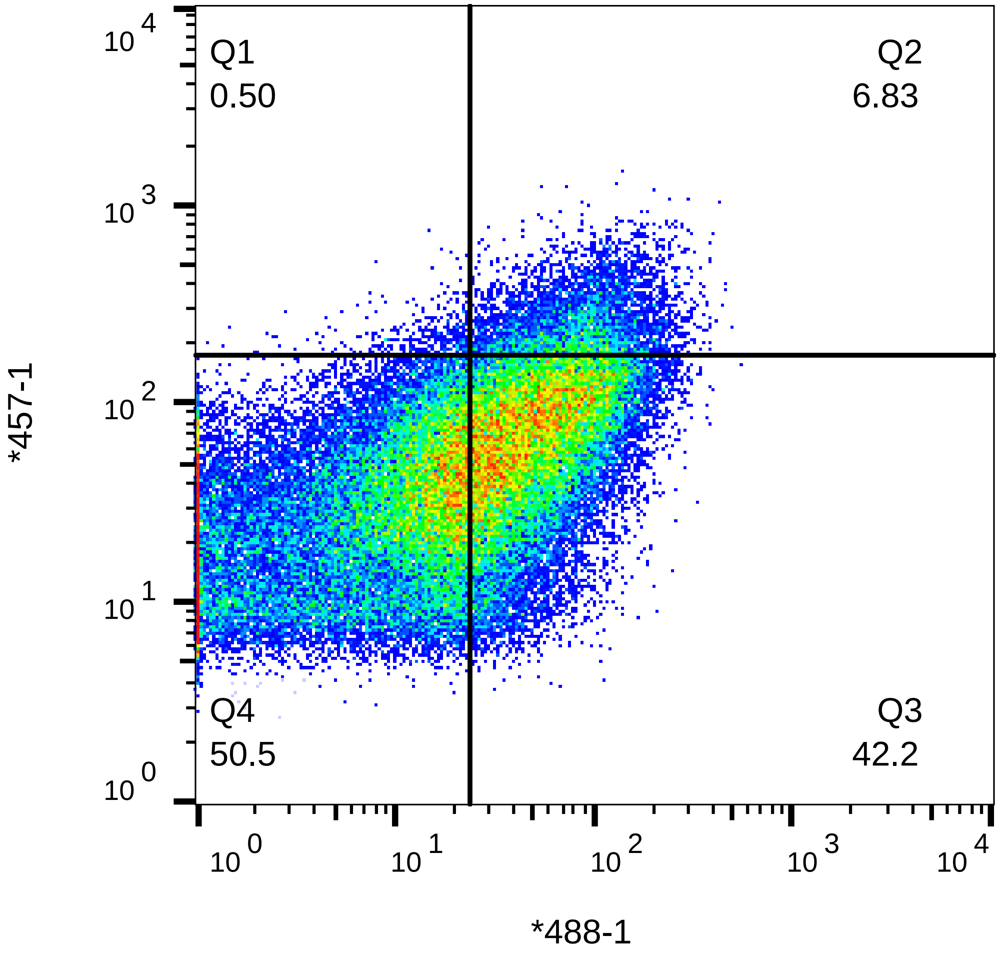

C:\Data\2020\Chrisler\20201007 Shank\SY569.fcs
pop
74446

ES2602; SY570; amyE::PaprE-Ypet (cam); lacA::PaprE-mTurq (erm)

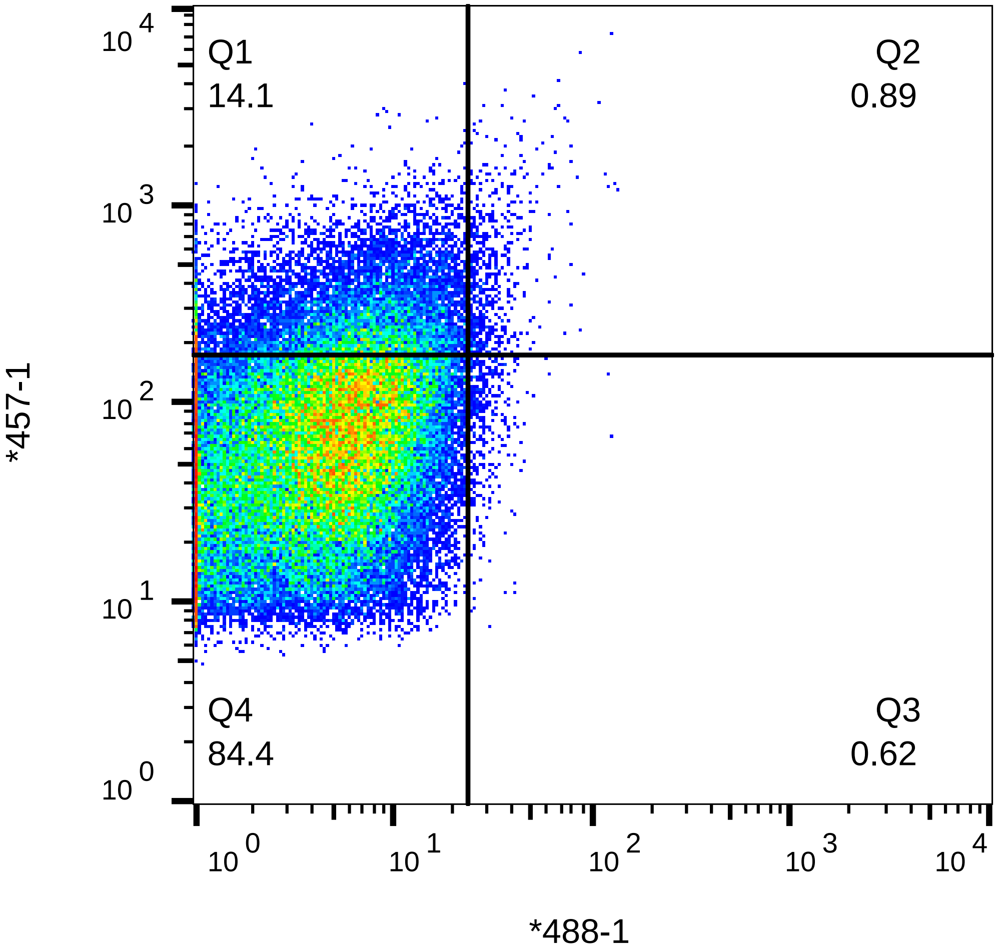

C:\Data\2020\Chrisler\20201007 Shank\SY570.fcs
pop
72961

ES2603; SY571; amyE::PdhbA-Ypet (cam); lacA::PdhbA-mTurq (erm)

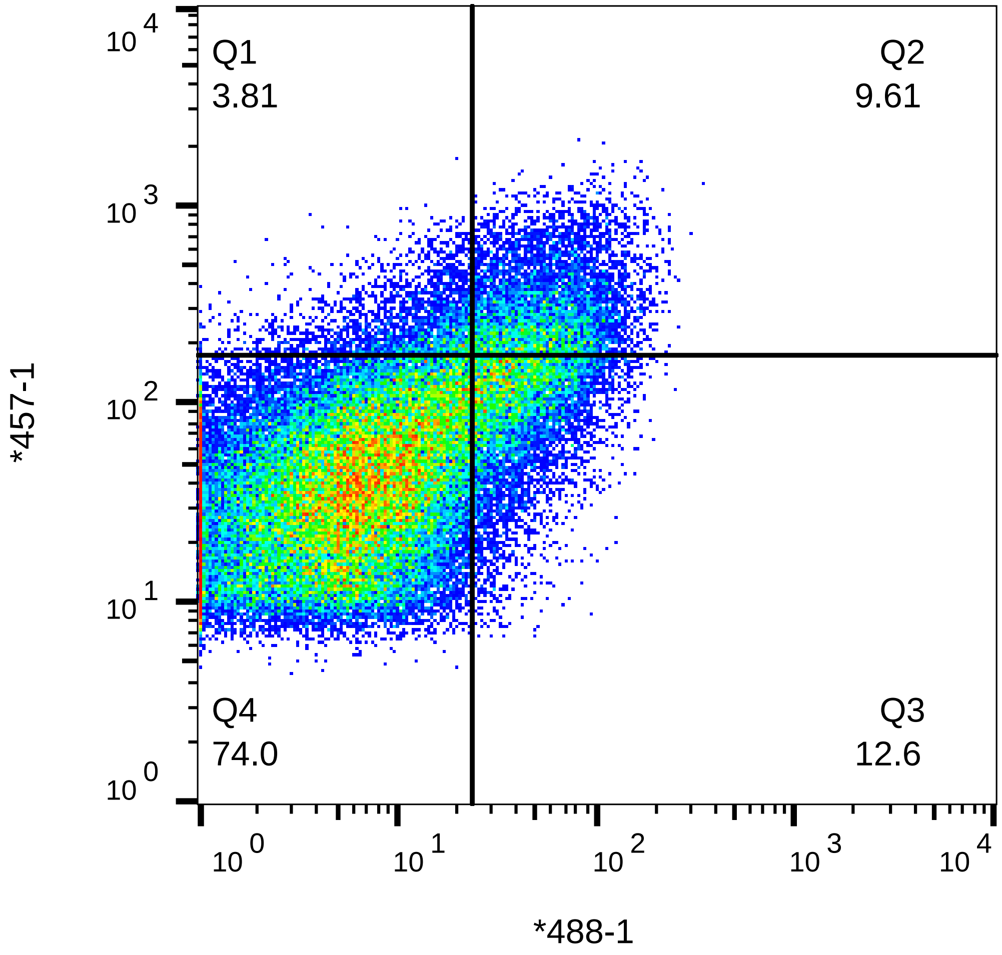

C:\Data\2020\Chrisler\20201007 Shank\SY571.fcs
pop
71758
