# Supplementary material for: Extensive cellular multi-tasking within Bacillus subtilis biofilms
Source: mSystems. 2023 Aug 1;8(4):e00891-22. doi: 10.1128/msystems.00891-22 (PMC10469600; doi:10.1128/msystems.00891-22)

2019-11-13

SY408 P_{dhbA}-Ypet (*cam*); P_{sboA}-mTurq (*erm*) interior

Brightfield

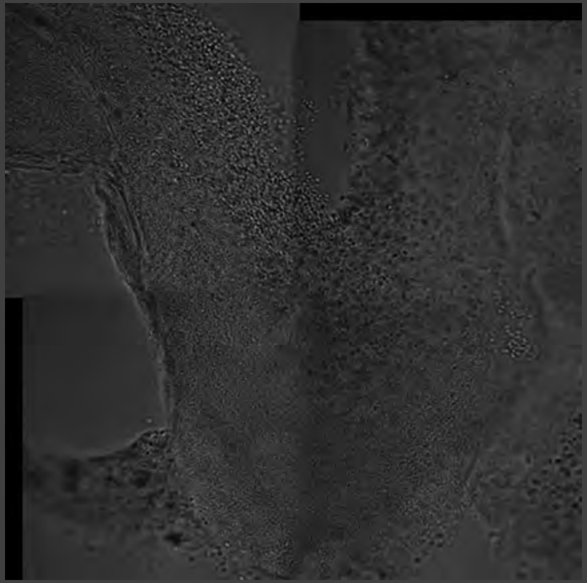

YPet

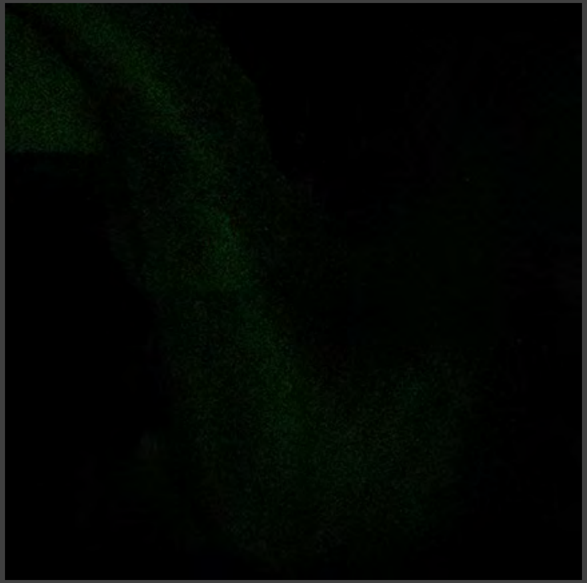

mTurq

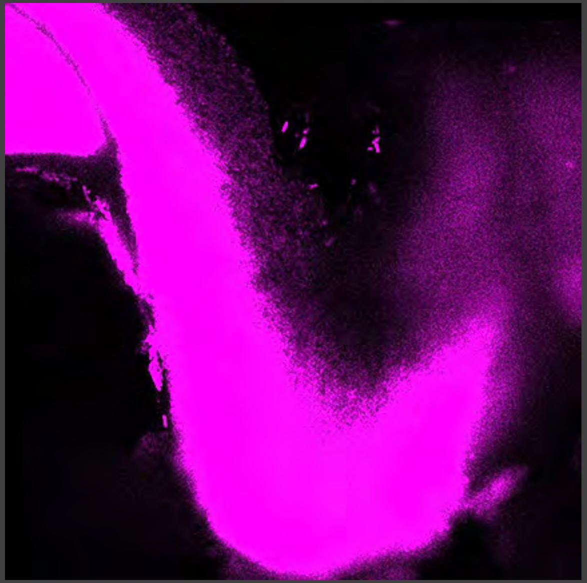

Merged

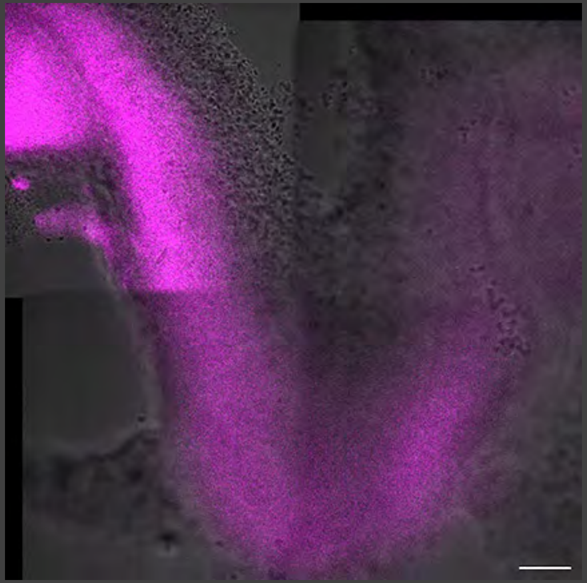

SY403 P_{pksC}-Ypet (*cam*); P_{aprE}-mTurq (*erm*) periphery

Brightfield YPet mTurq Merged

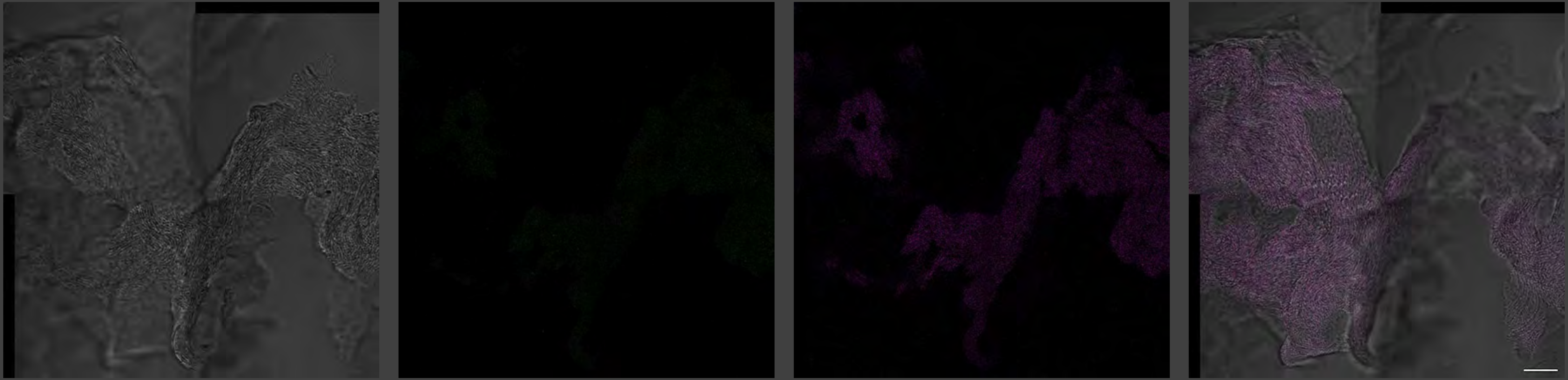

SY403 P_{pksC}-Ypet (*cam*); P_{aprE}-mTurq (*erm*) interior

2019-11-13

Brightfield | YPet | mTurq | Merged

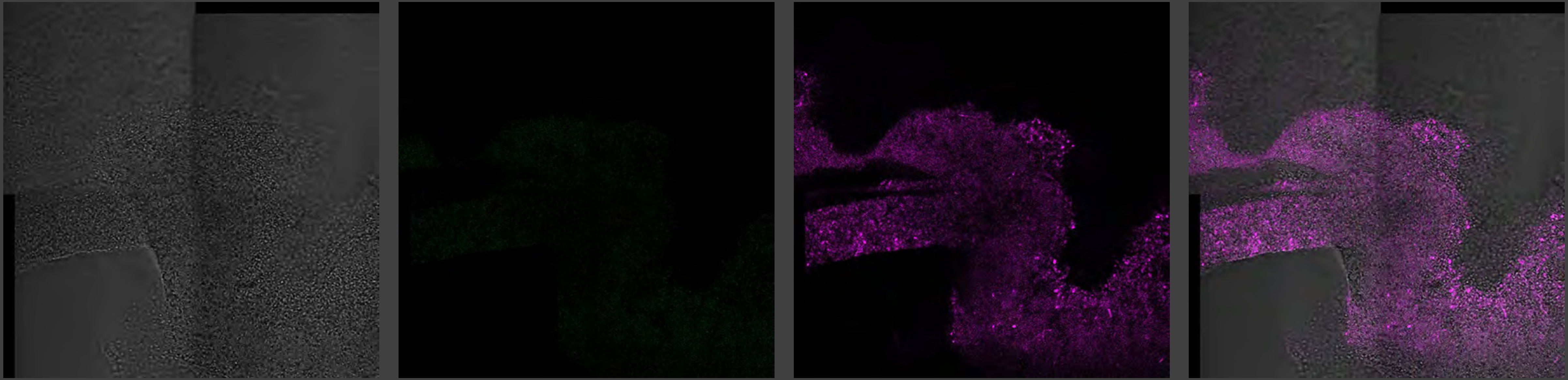

SY405 P_{dhbA}-Ypet (*cam*); P_{bacA}-mTurq (*erm*) periphery

2019-11-13

Brightfield YPet mTurq Merged

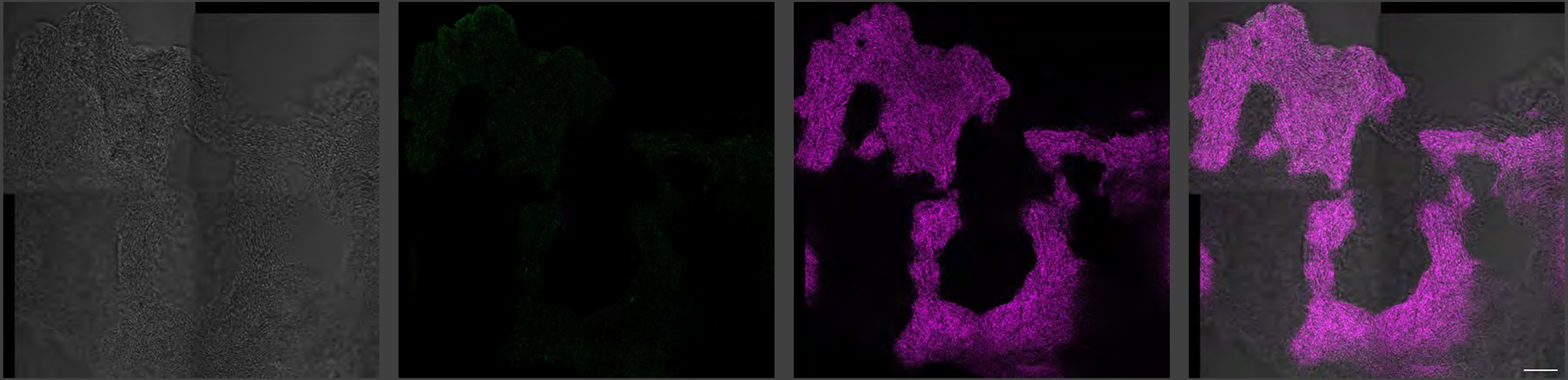

SY405 P_{dhbA}-Ypet (*cam*); P_{bacA}-mTurq (*erm*) interior

2019-11-13

Brightfield YPet mTurq Merged

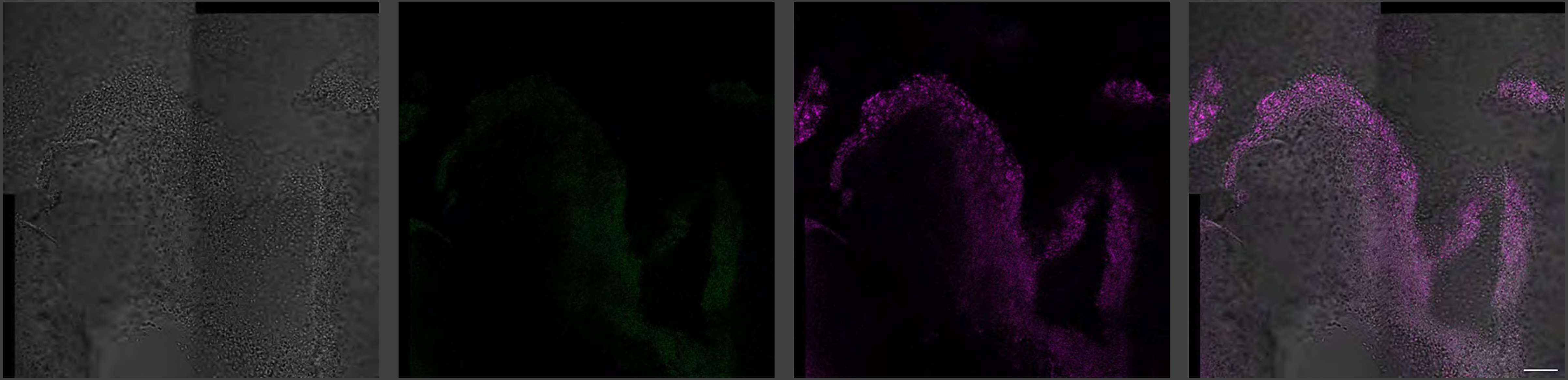

SY318 P_{tapA}-Ypet (*cam*); P_{bacA}-mTurq (*erm*) periphery

Brightfield YPet mTurq Merged

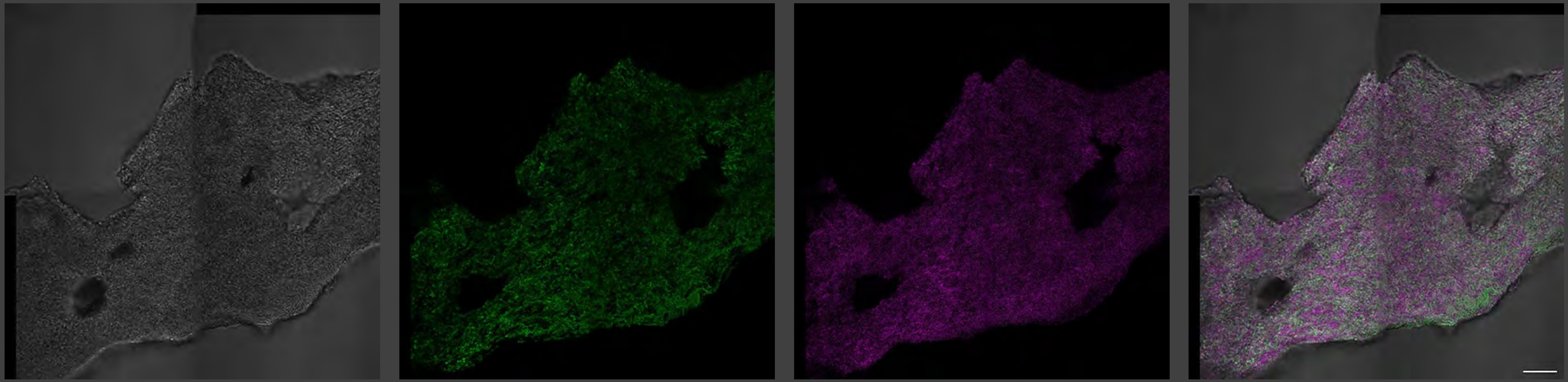

2019-11-15

SY318 P_{tapA}-Ypet (*cam*); P_{bacA}-mTurq (*erm*) interior

Brightfield | YPet | mTurq | Merged

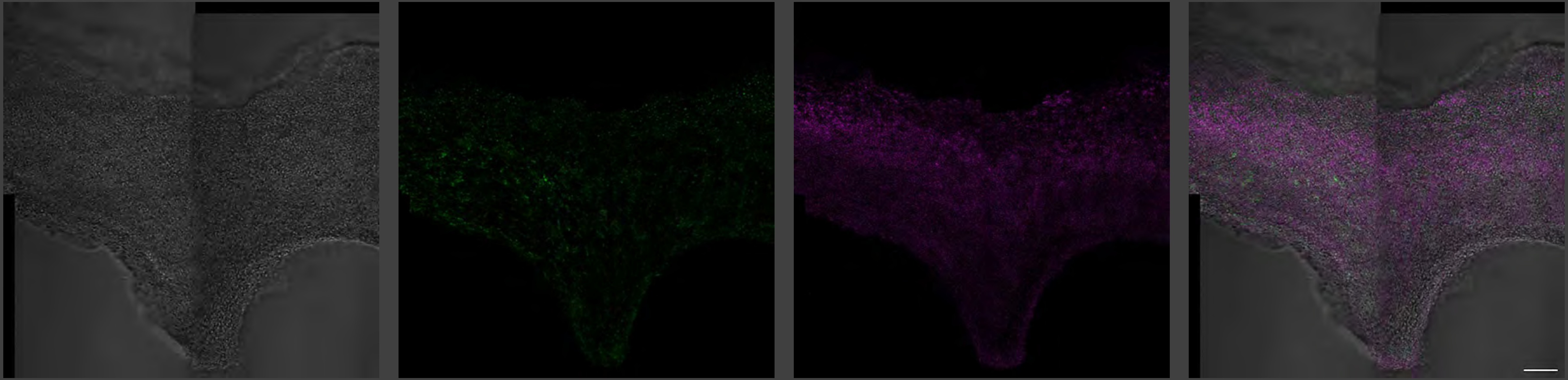

2019-11-15

SY329 P_{sspB}-Ypet (*cam*); P_{hag}-mTurq (*erm*) periphery

Brightfield | YPet | mTurq | Merged

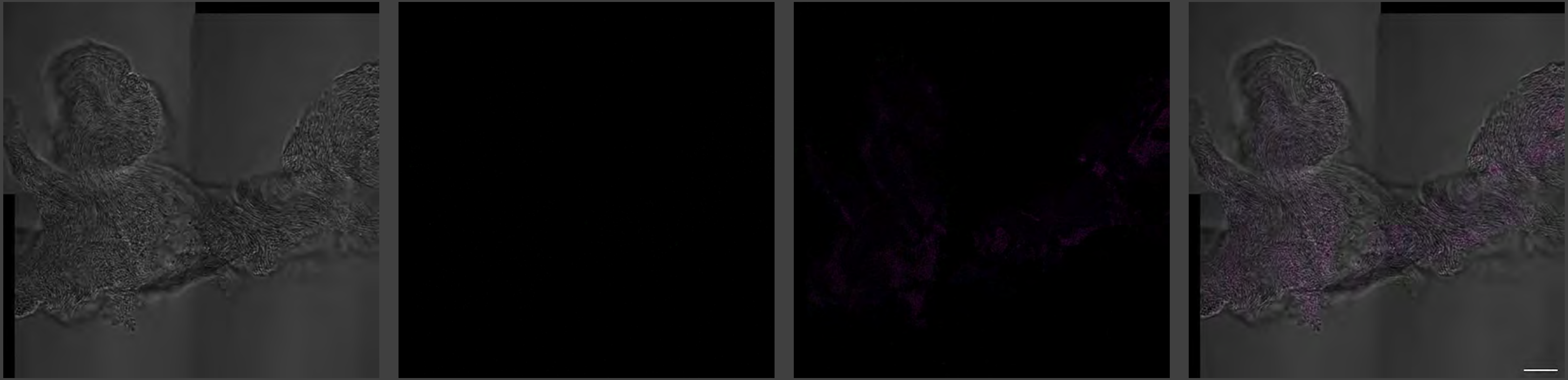

SY329 P_{sspB}-Ypet (*cam*); P_{hag}-mTurq (*erm*) interior

Brightfield

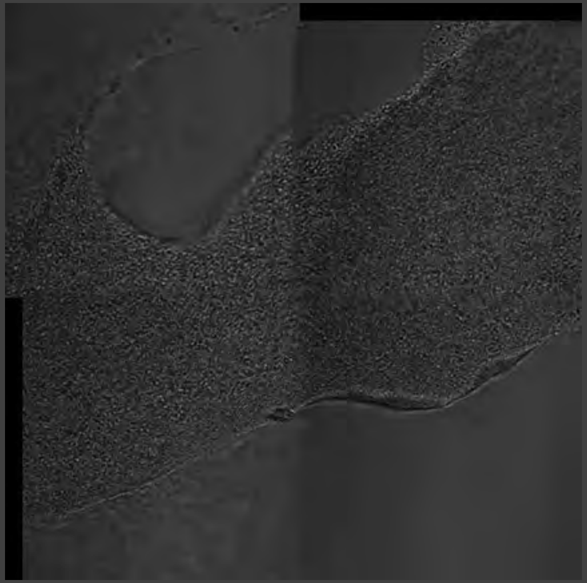

YPet

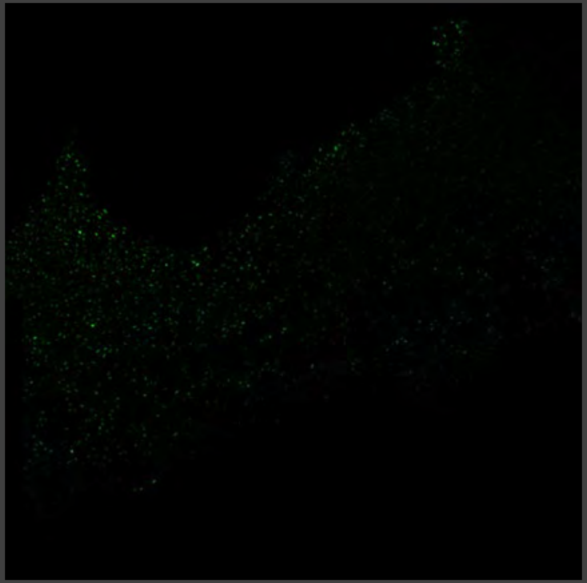

mTurq

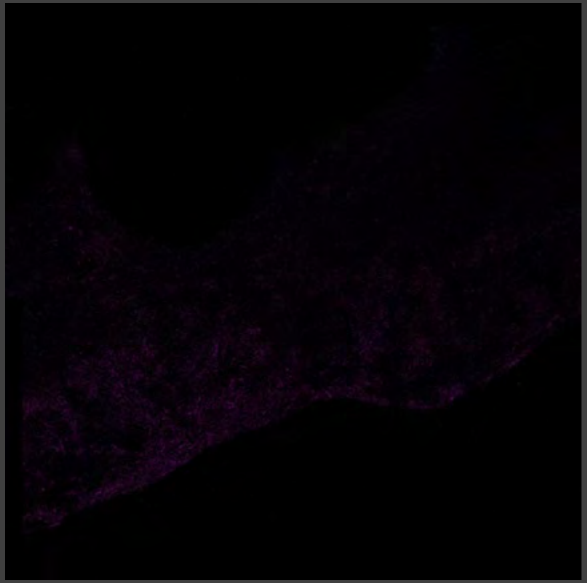

Merged

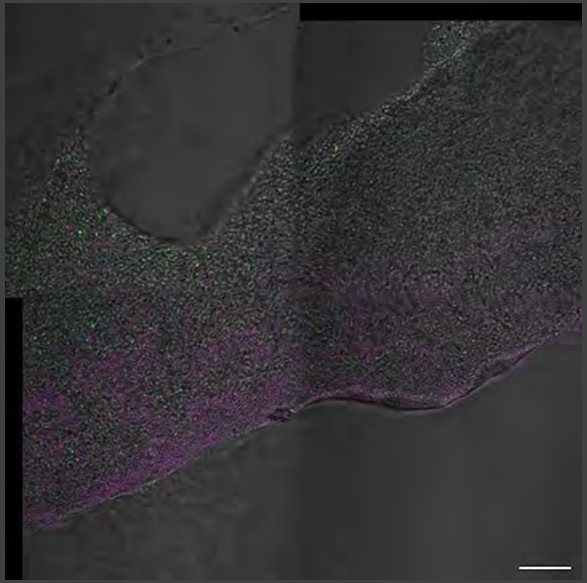

2019-11-18

SY376 $P_{\textit{sdpA}}$-Ypet (*cam*); $P_{\textit{aprE}}$-mTurq (*erm*) periphery

Brightfield

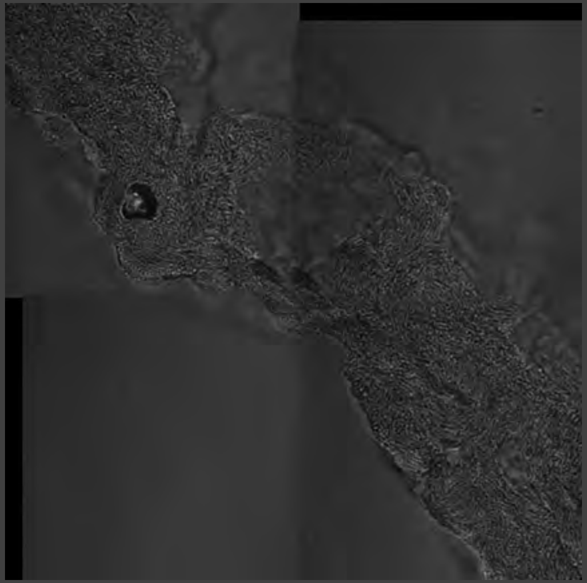

YPet

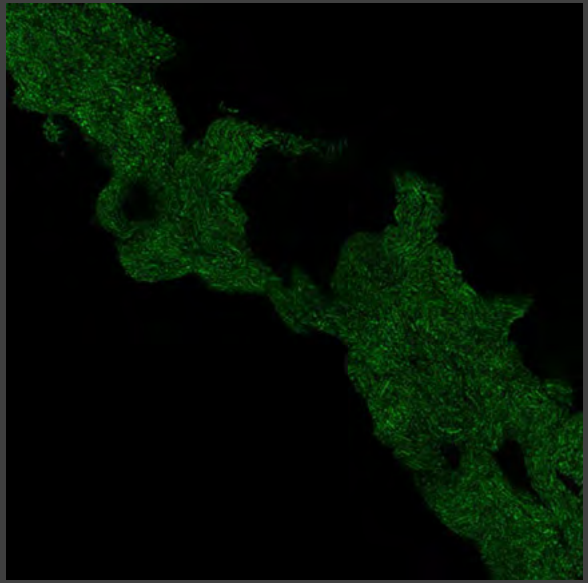

mTurq

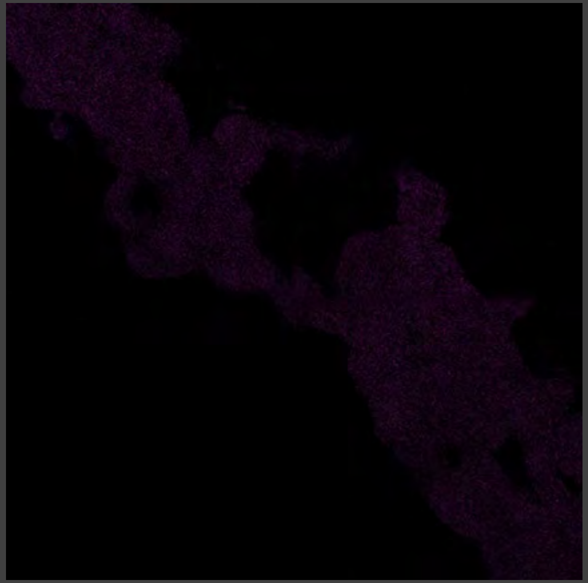

Merged

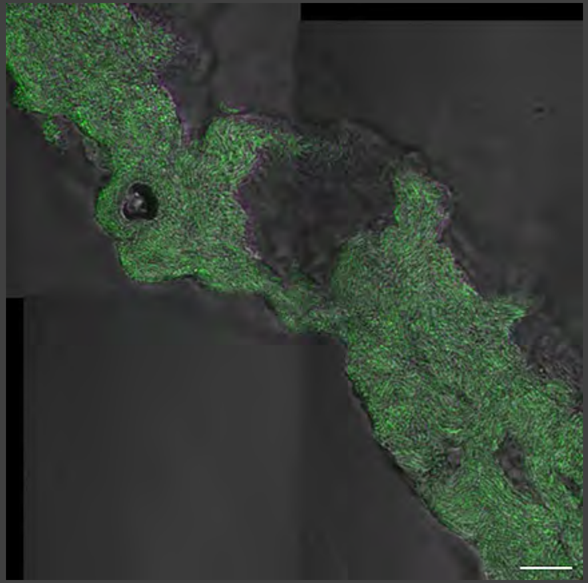

SY376 $P_{\textit{sdpA}}$-Ypet (*cam*); $P_{\textit{aprE}}$-mTurq (*erm*) interior

Brightfield | YPet | mTurq | Merged

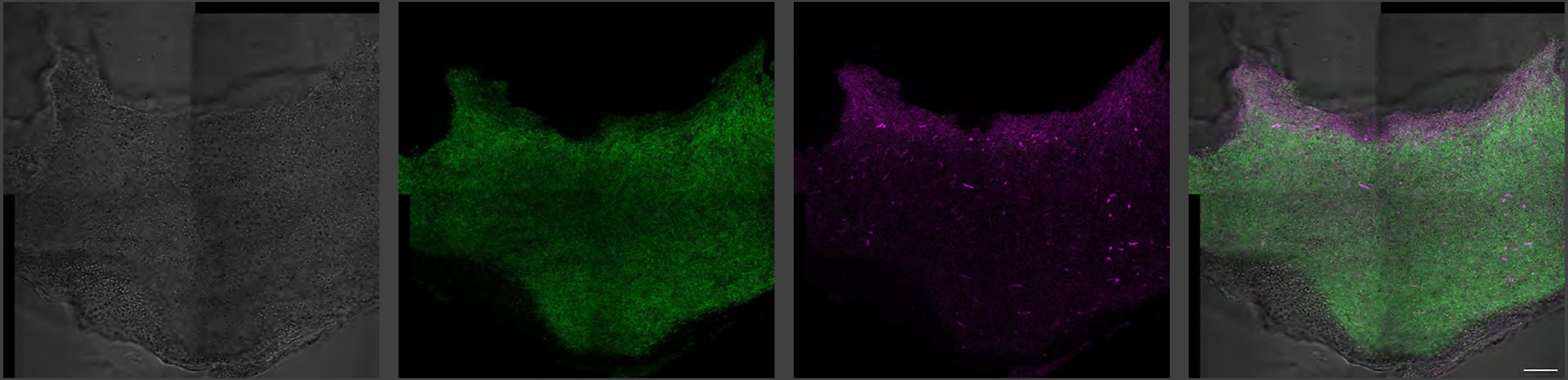

2019-11-18

SY426 P_{bacA}-Ypet (cam); P_{aprE}-mTurq (erm) periphery

Brightfield YPet mTurq Merged

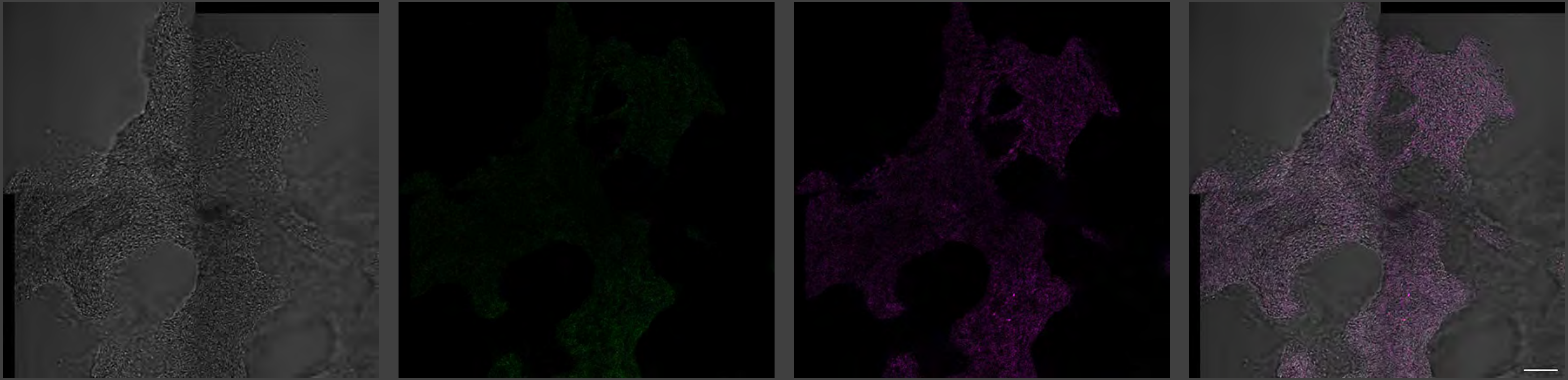

2019-11-19

SY414 P_{dhbA}-Ypet (*cam*); P_{skfA}-mTurq (*erm*) periphery

Brightfield

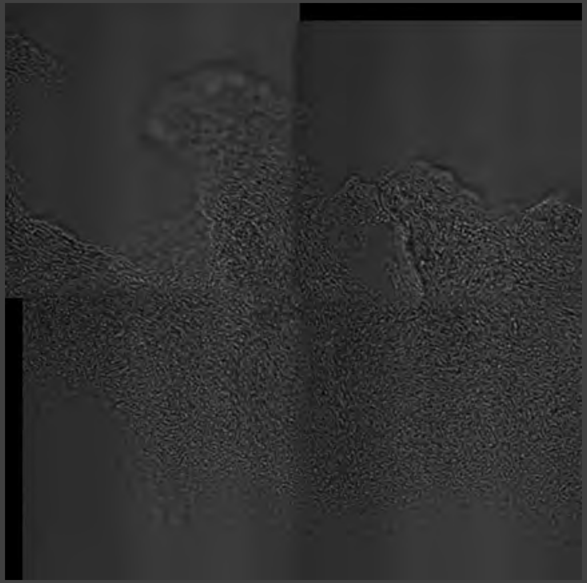

YPet

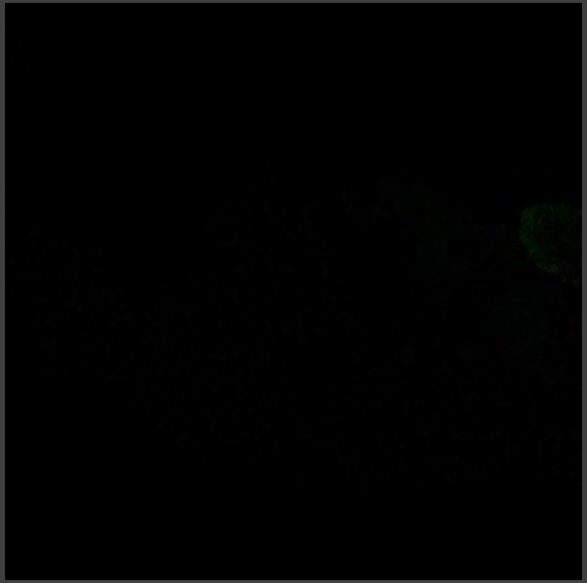

mTurq

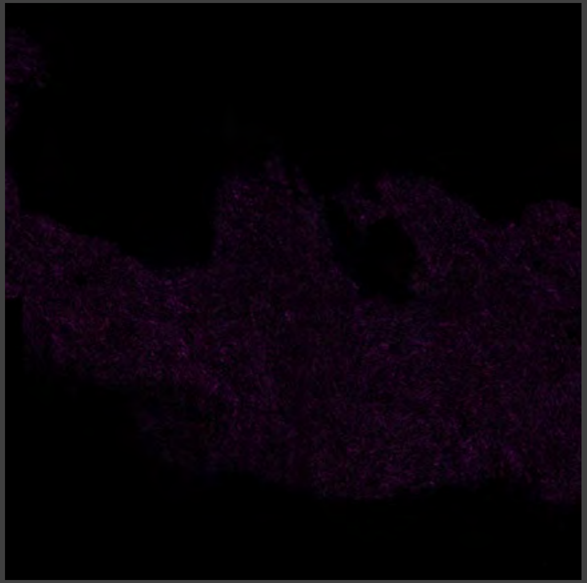

Merged

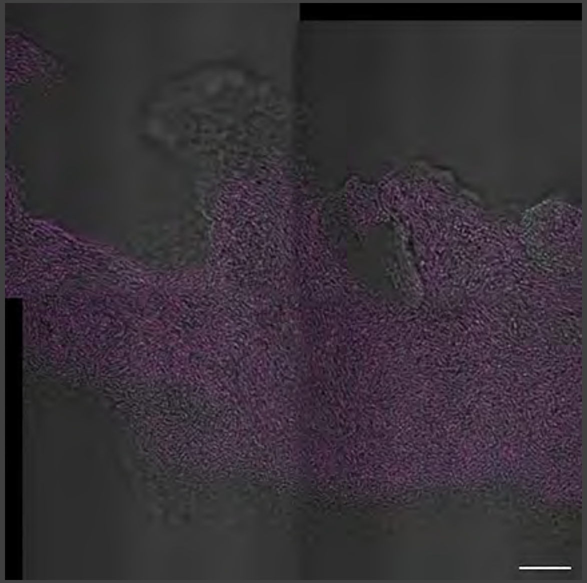

2019-11-19

SY414 P_{dhbA}-Ypet (*cam*); P_{skfA}-mTurq (*erm*) interior

Brightfield

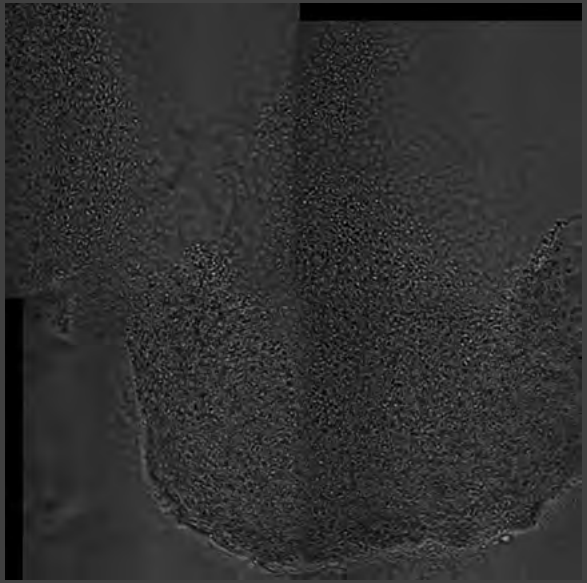

YPet

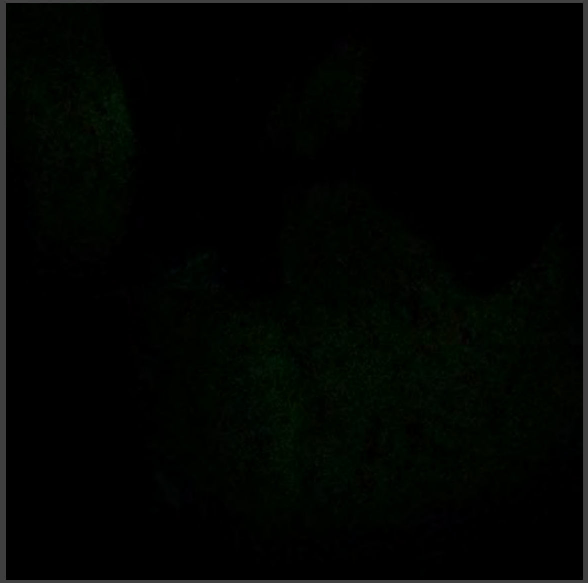

mTurq

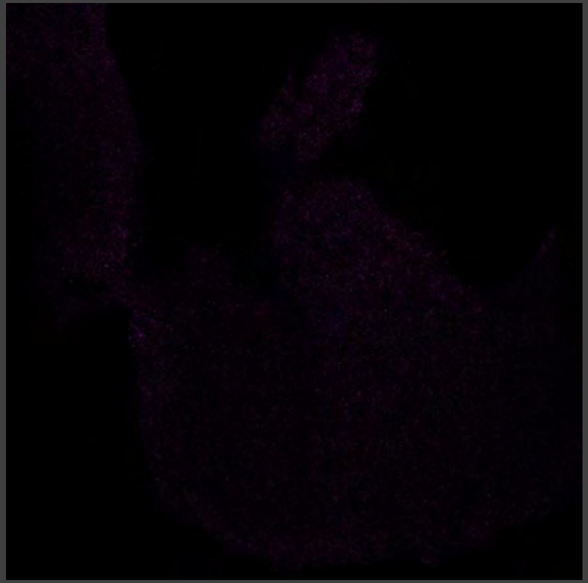

Merged

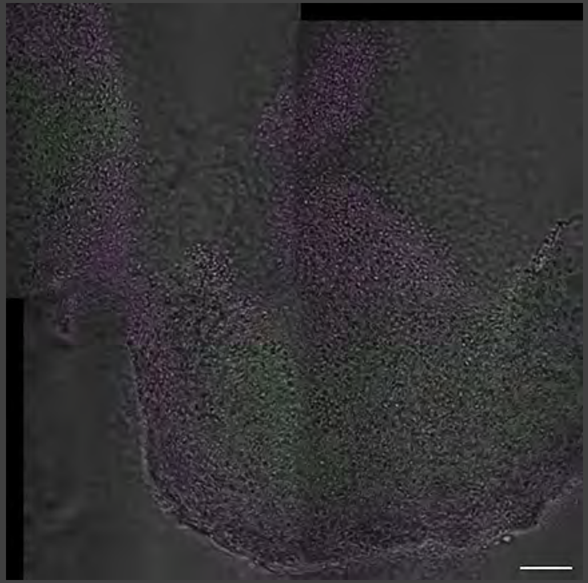

SY425 P_{bacA}-Ypet (*cam*); P_{skfA}-mTurq (*erm*) periphery

Brightfield

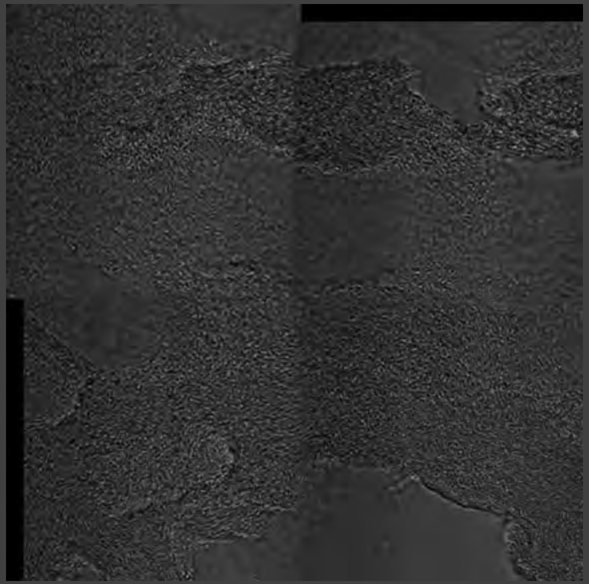

YPet

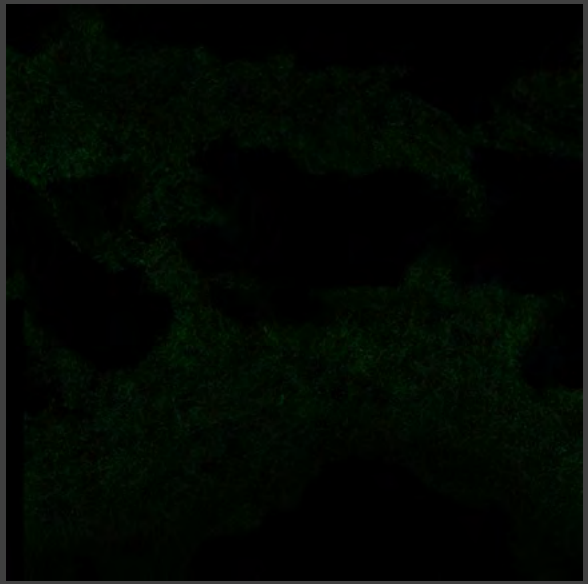

mTurq

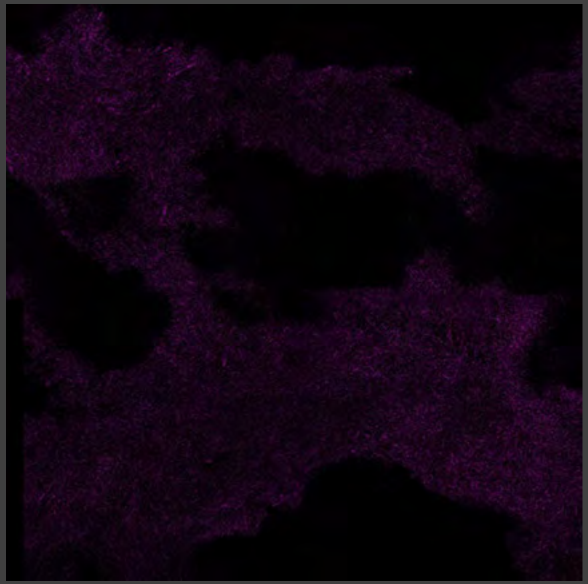

Merged

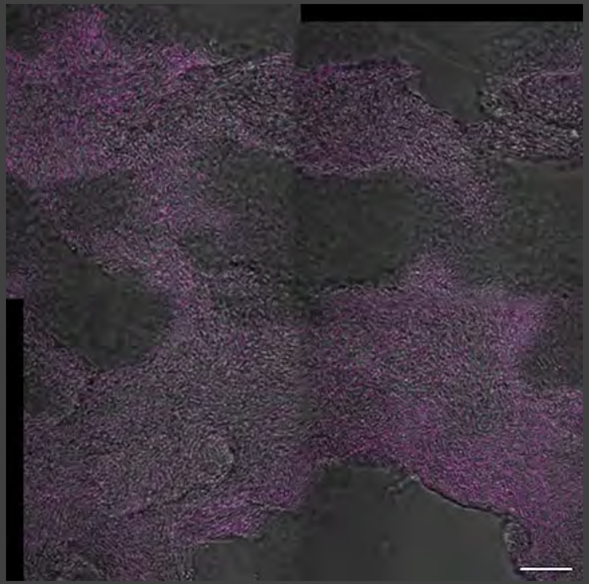

2019-11-19

SY425 P_{bacA}-Ypet (*cam*); P_{skfA}-mTurq (*erm*) interior

Brightfield

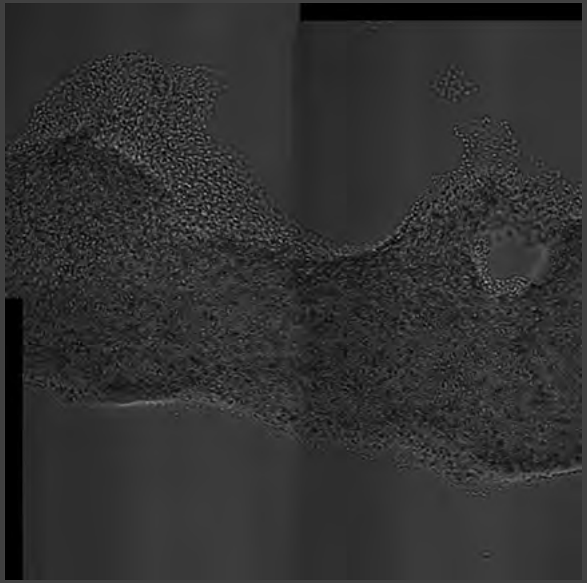

YPet

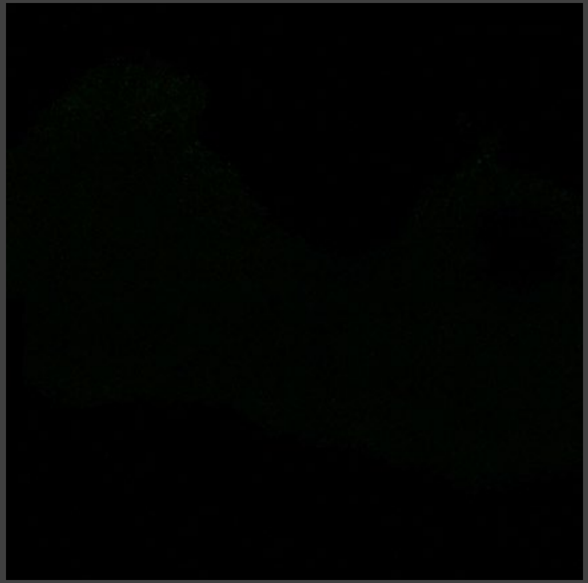

mTurq

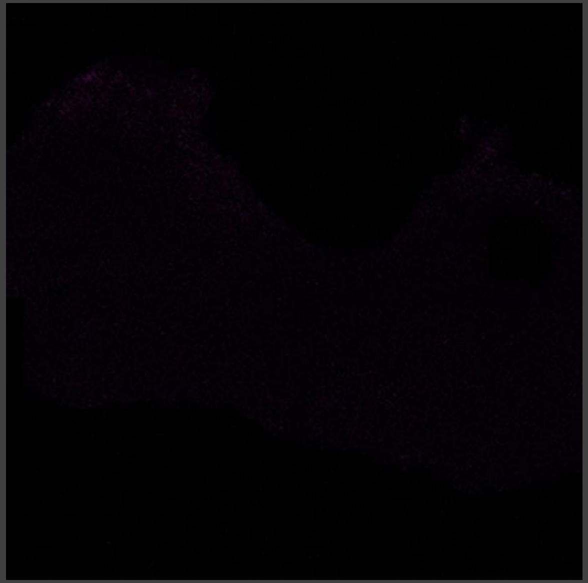

Merged

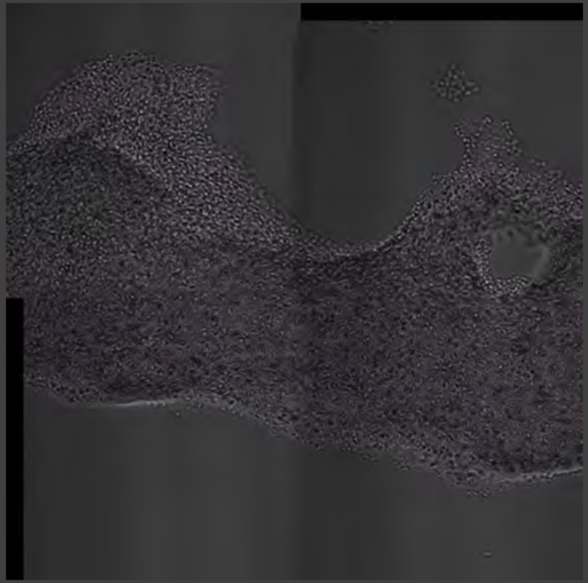

SY375 PsdpA-Ypet (cam); PskfA-mTurq (erm) periphery

Brightfield

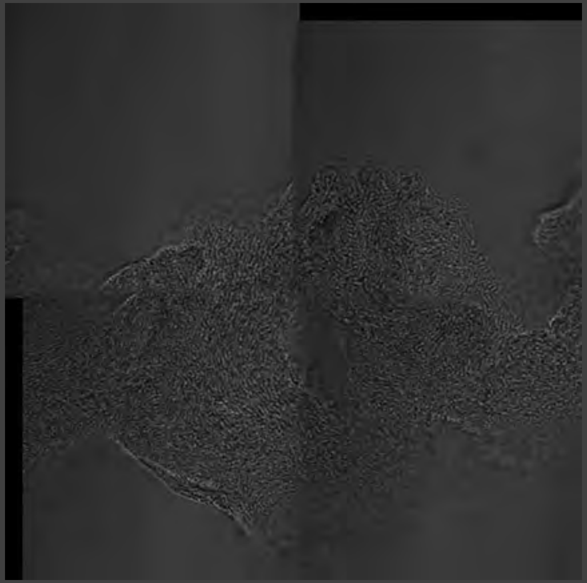

YPet

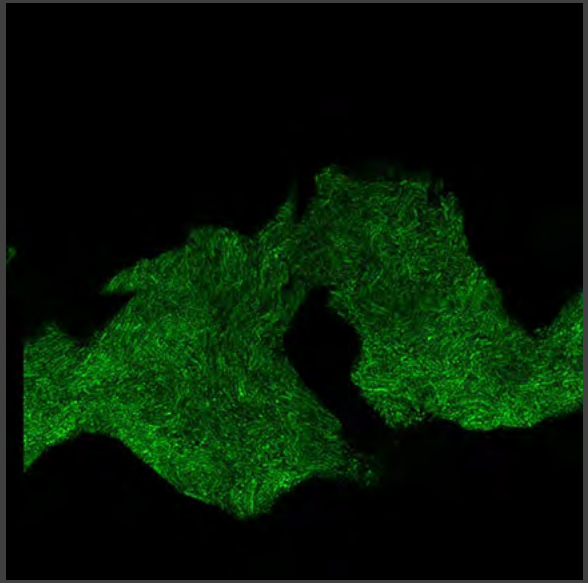

mTurq

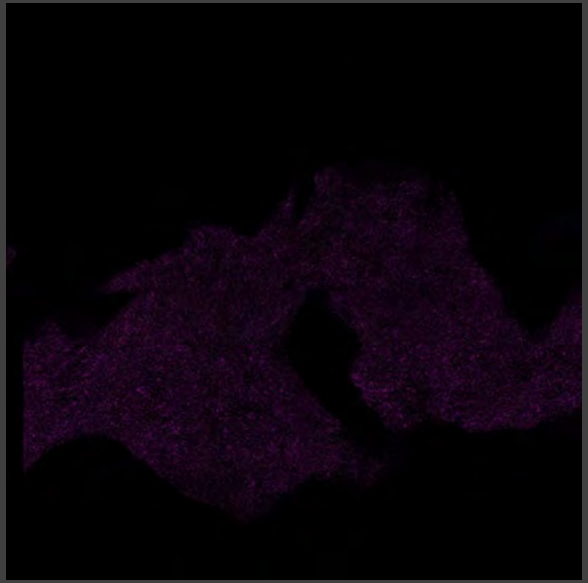

Merged

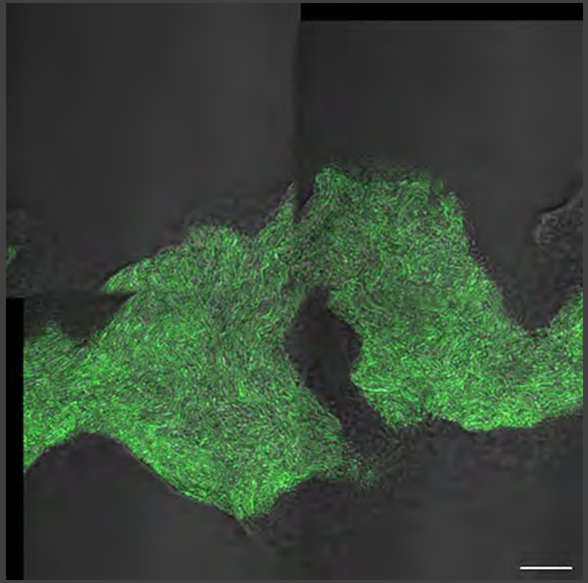

SY375 P_{sdpA}-Ypet (*cam*); P_{skfA}-mTurq (*erm*) interior

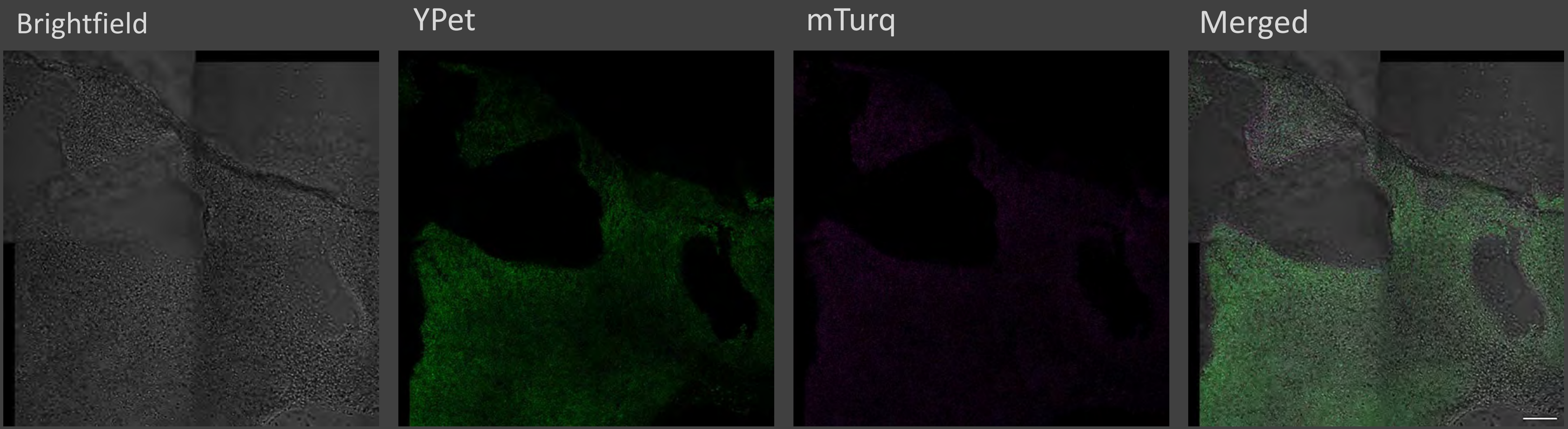

SY415 P$_{dhbA}$-Ypet (*cam*); P$_{aprE}$-mTurq (*erm*) periphery

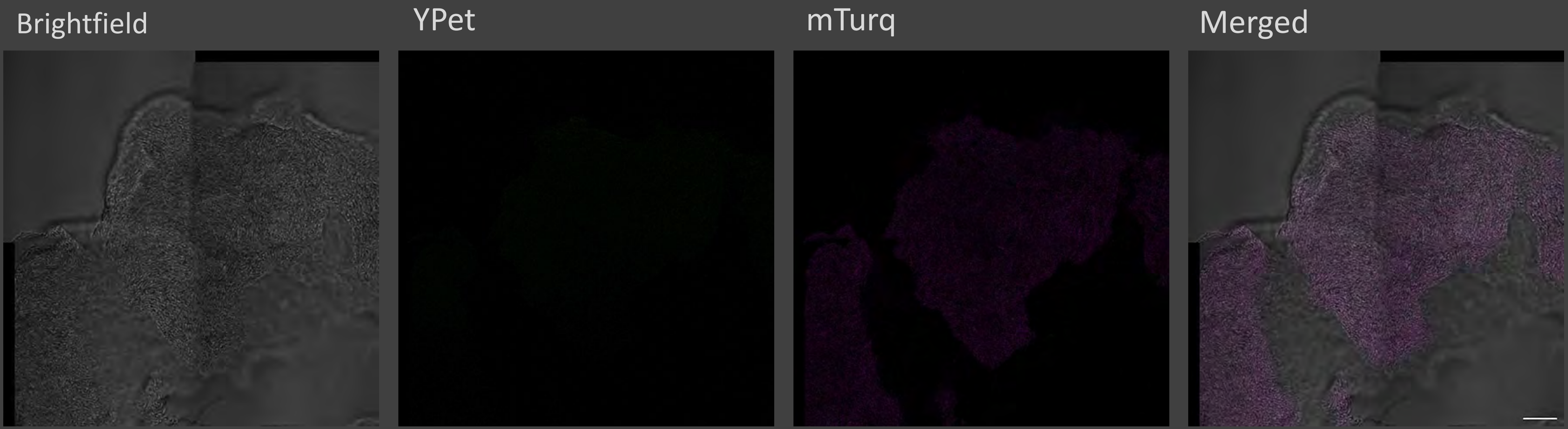

SY415 P_{dhbA}-Ypet (*cam*); P_{aprE}-mTurq (*erm*) interior

Brightfield | YPet | mTurq | Merged

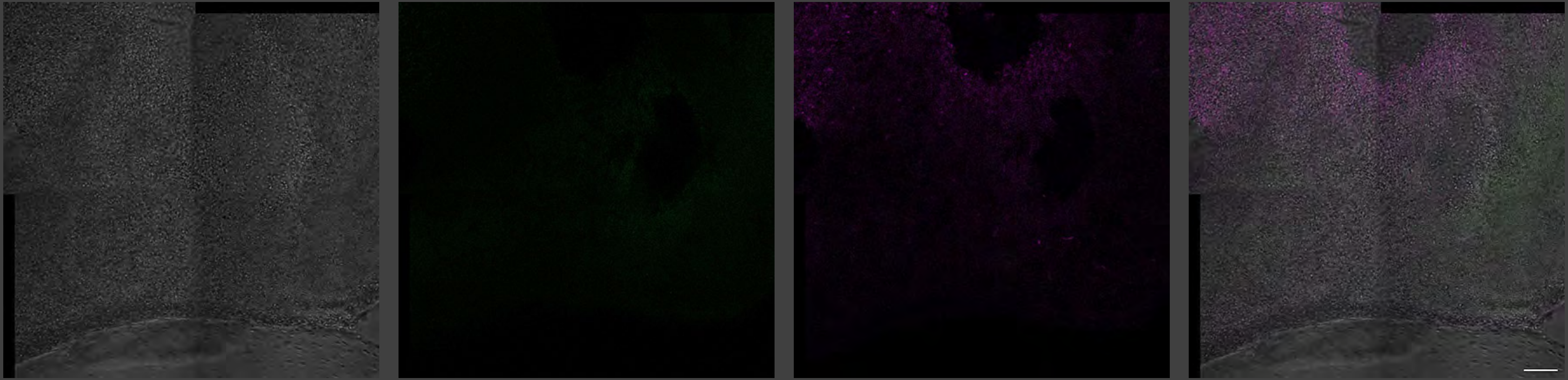

SY393 P_{pksC}-Ypet (cam); P_{bacA}-mTurq (erm) periphery

Brightfield | YPet | mTurq | Merged

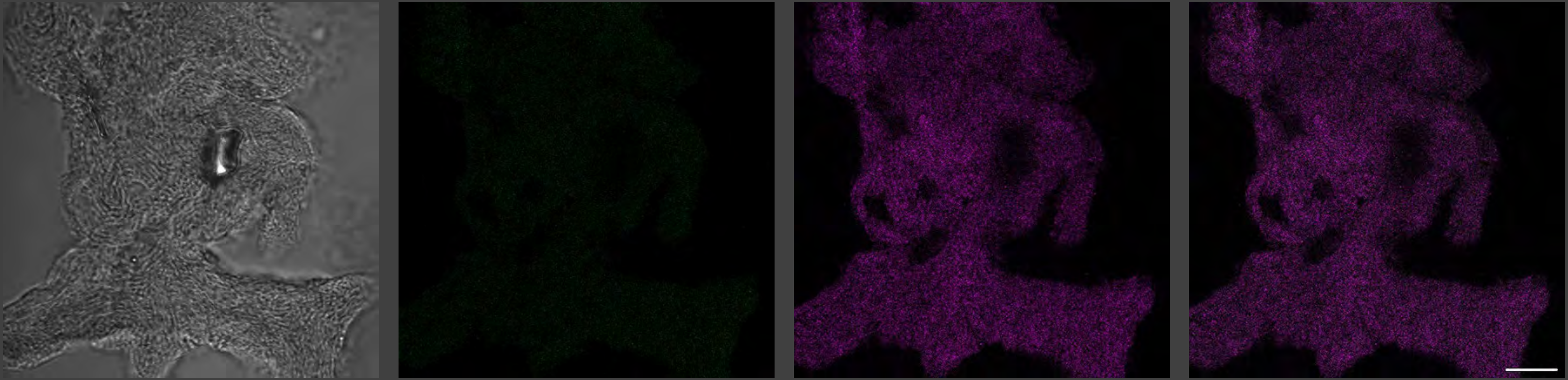

SY393 P_{pksC}-Ypet (cam); P_{bacA}-mTurq (erm) middle

Brightfield | YPet | mTurq | Merged

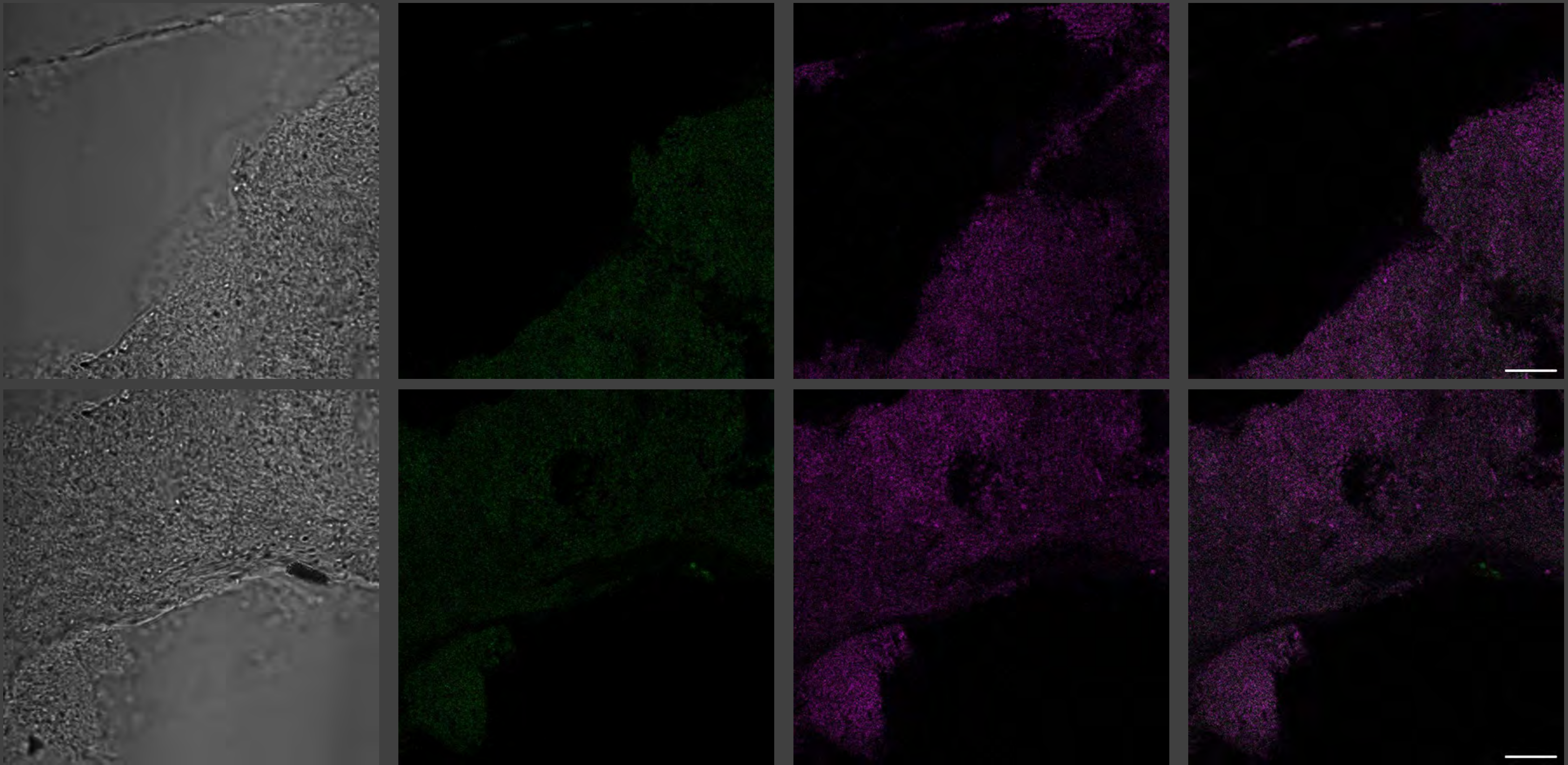

2019-12-12

SY311 P_{tapA}-Ypet (*cam*); P_{hag}-mTurq (*erm*) periphery

Brightfield | YPet | mTurq | Merged

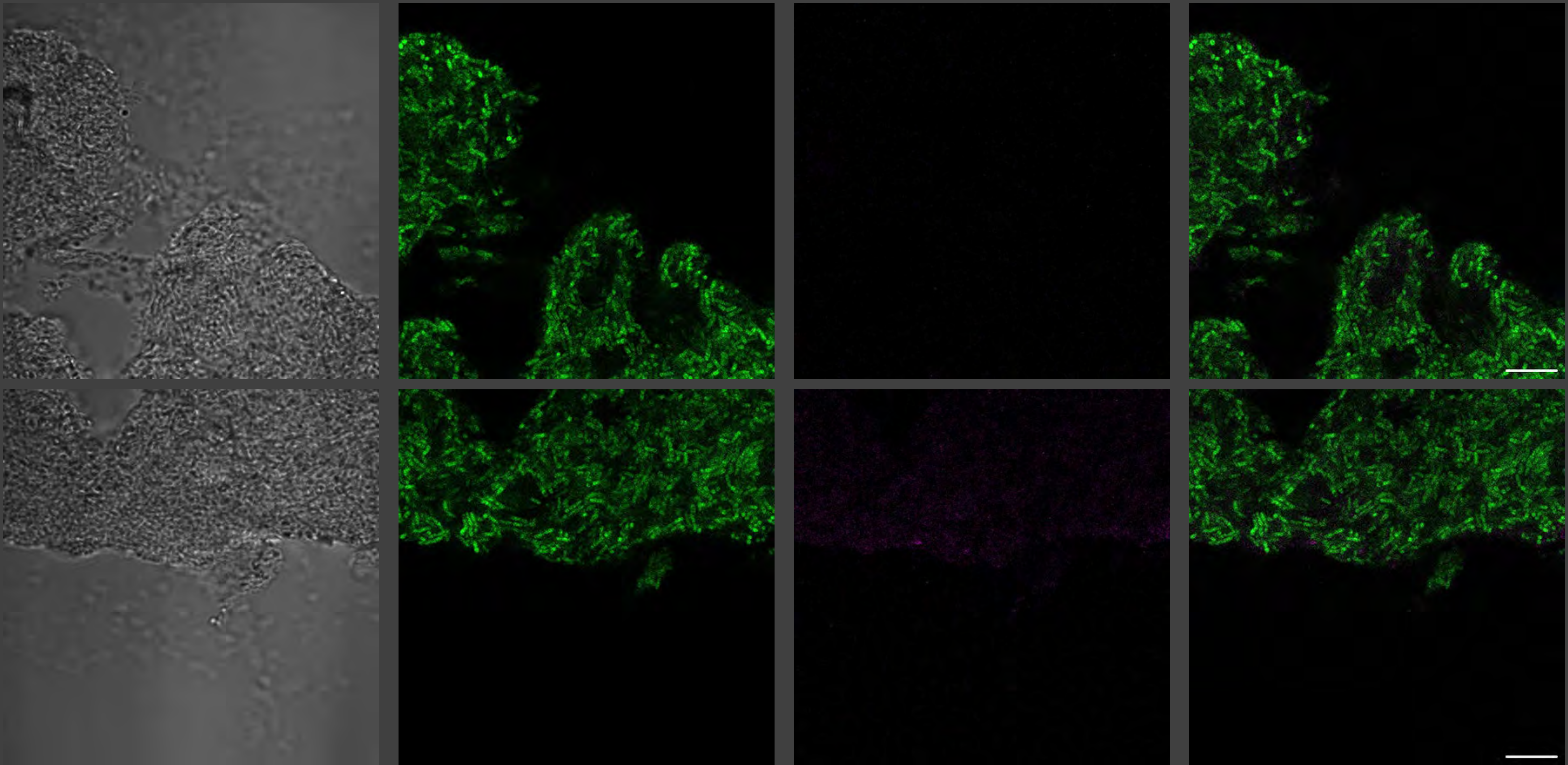

SY311 P_{tapA}-Ypet (*cam*); P_{hag}-mTurq (*erm*) middle

2019-12-12

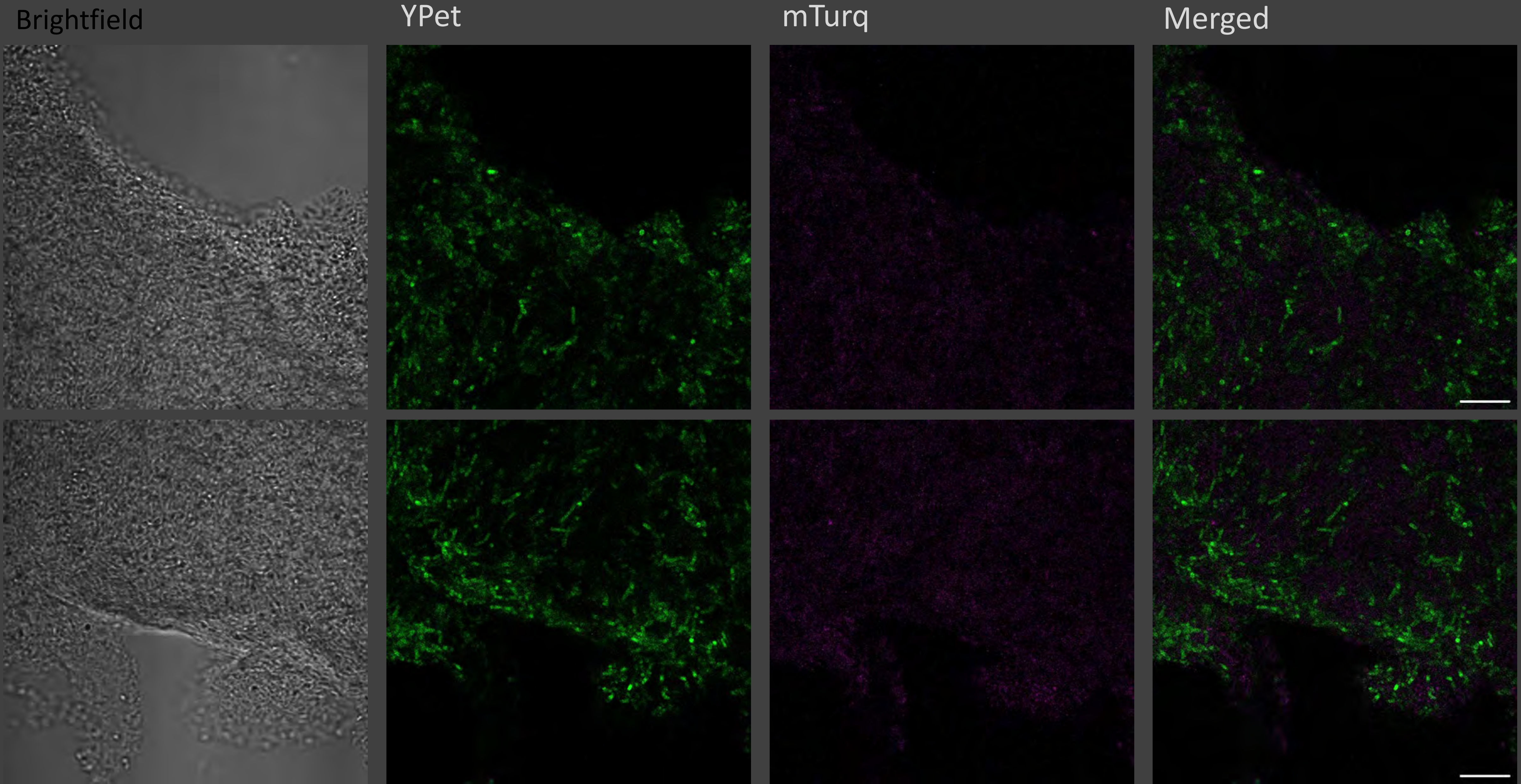

2019-12-12

SY311 P_{tapA}-Ypet (*cam*); P_{hag}-mTurq (*erm*) interior

Brightfield | YPet | mTurq | Merged

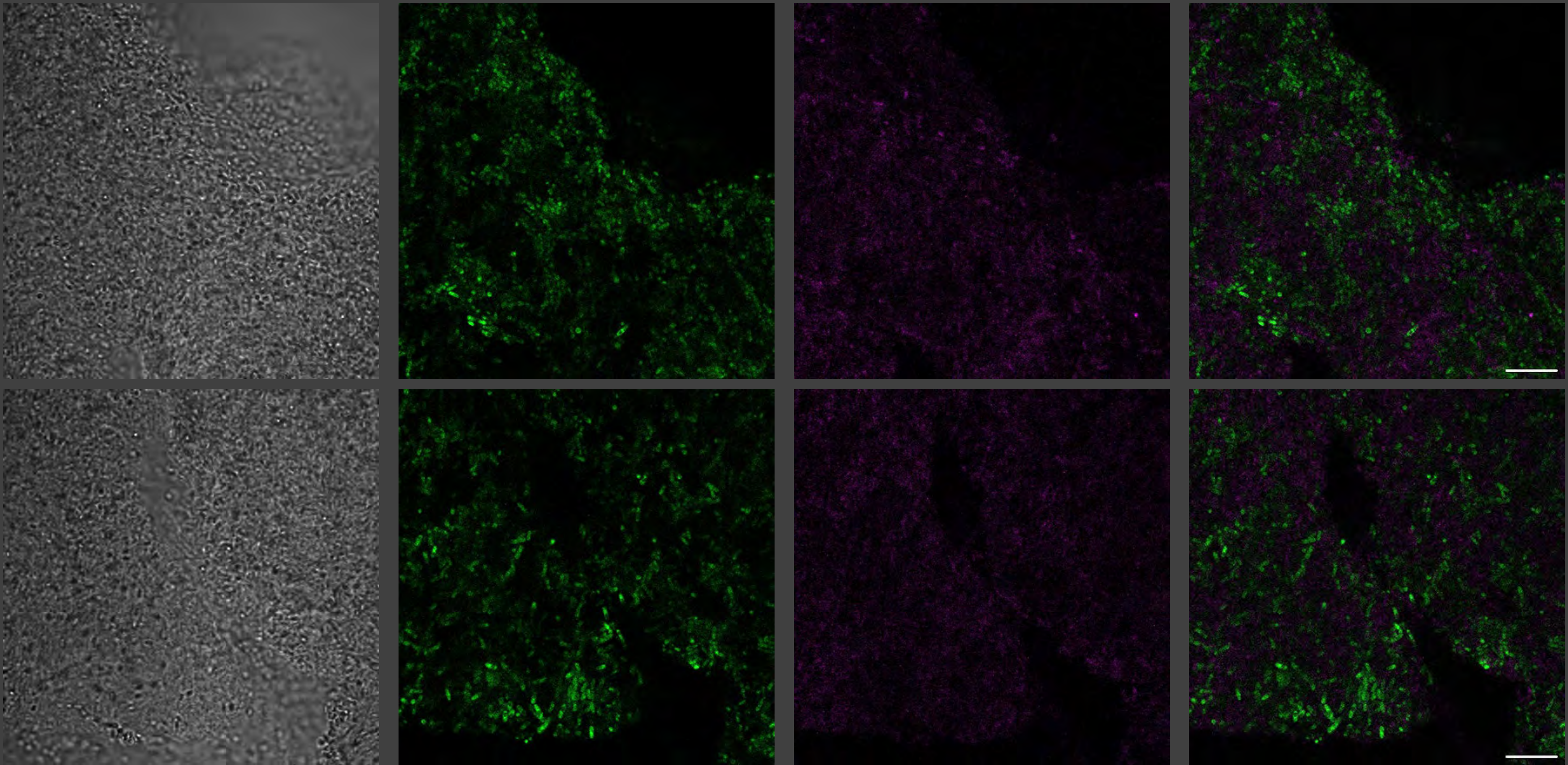

SY346 P_{hag}-Ypet (*cam*); P_{sdpA}-mTurq (*erm*) periphery

Brightfield YPet mTurq Merged

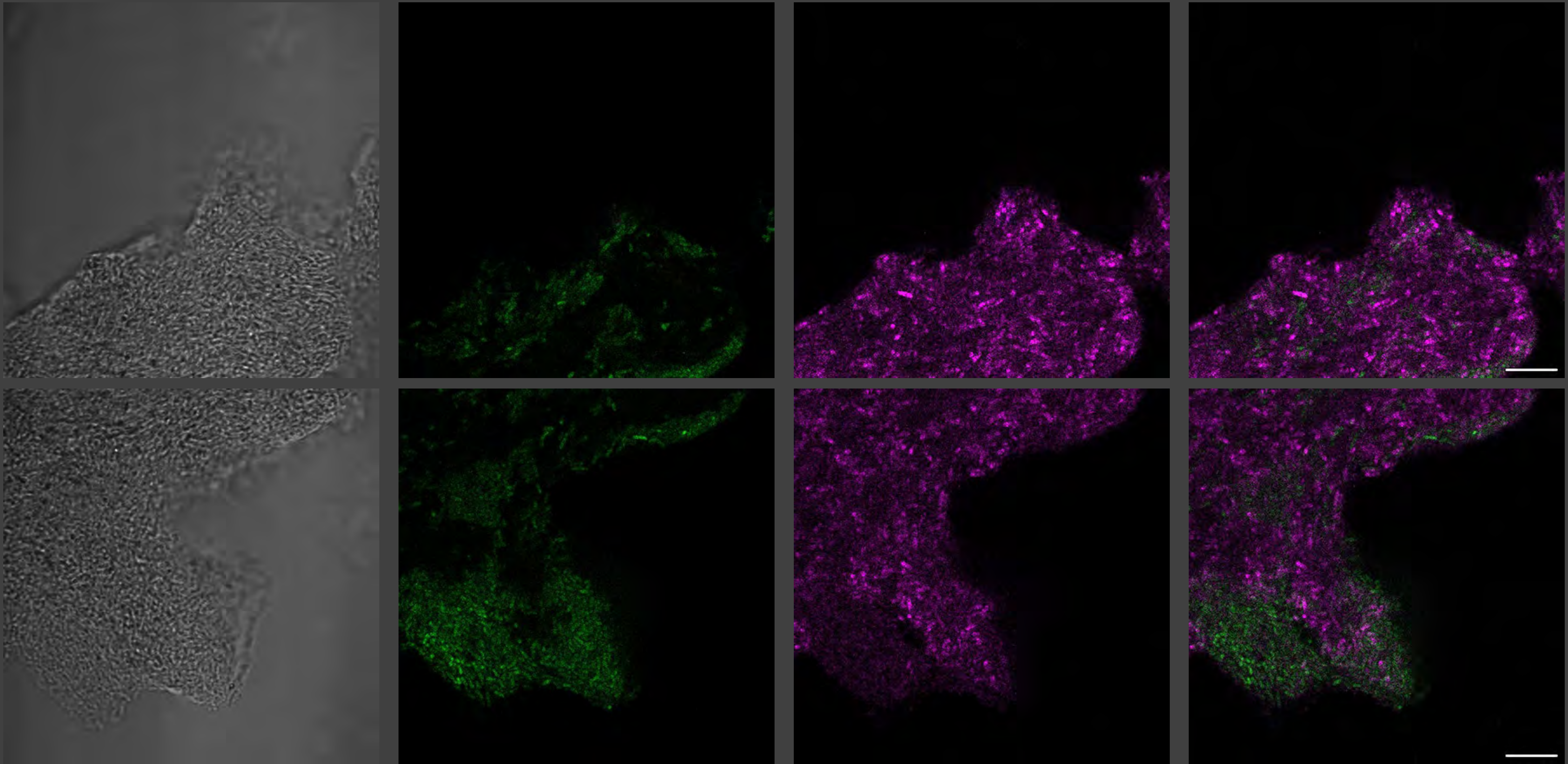

2019-12-12

SY346 P_{hag}-Ypet (*cam*); P_{sdpA}-mTurq (*erm*) middle

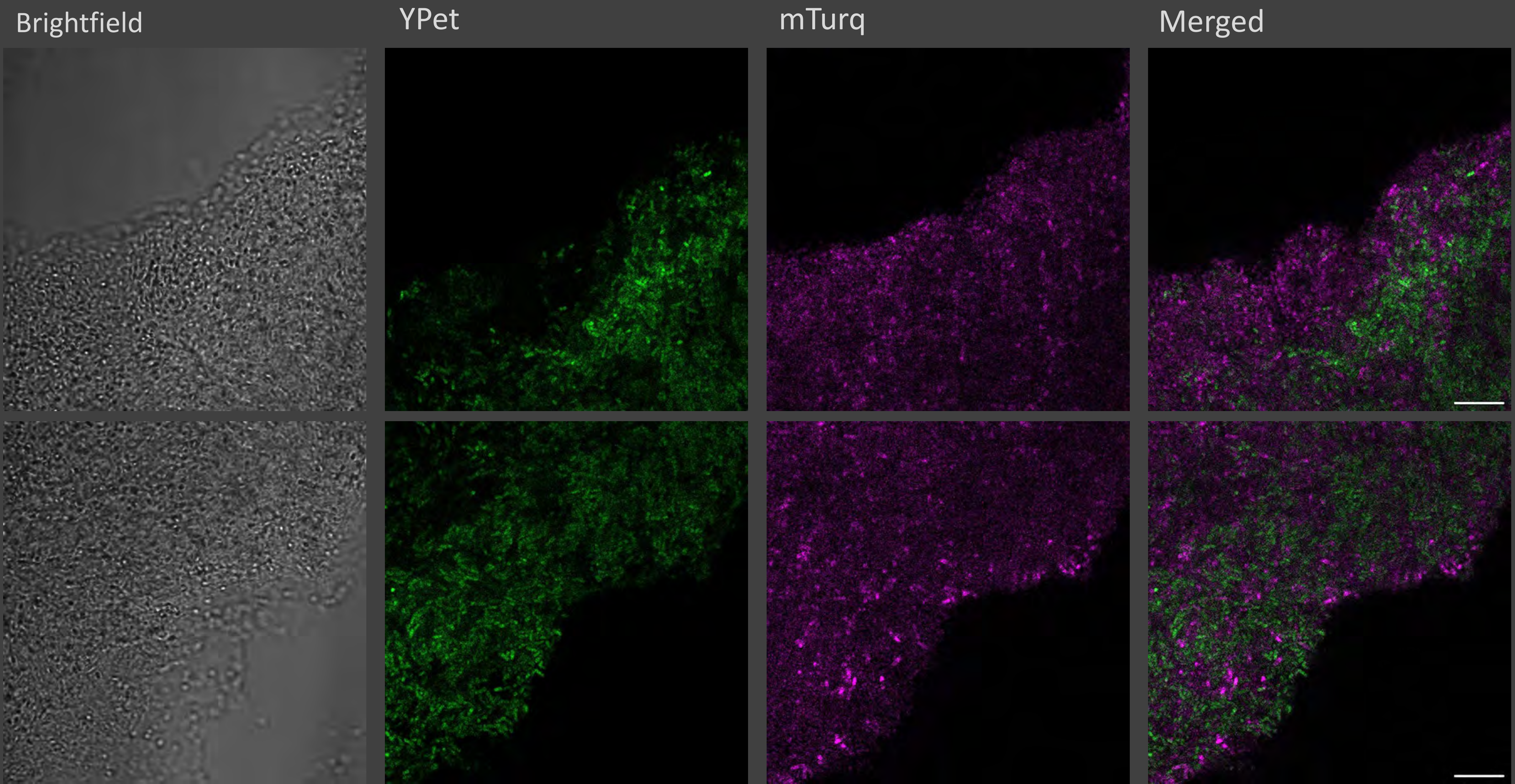

SY346 P_{hag}-Ypet (*cam*); P_{sdpA}-mTurq (*erm*) interior

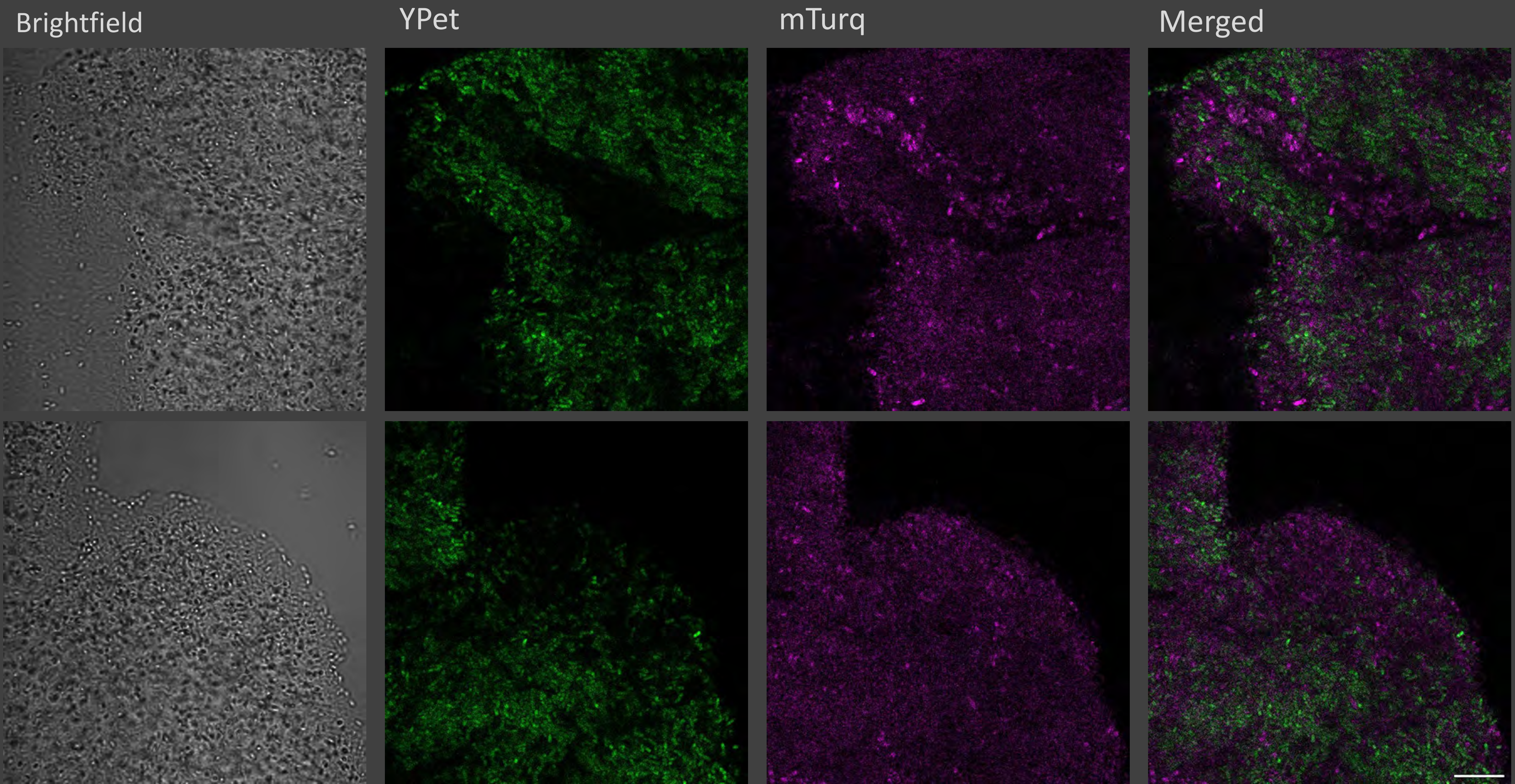

SY421 P_{bacA}-Ypet (*cam*); P_{comQX}-mTurq (*erm*) periphery

Brightfield | YPet | mTurq | Merged

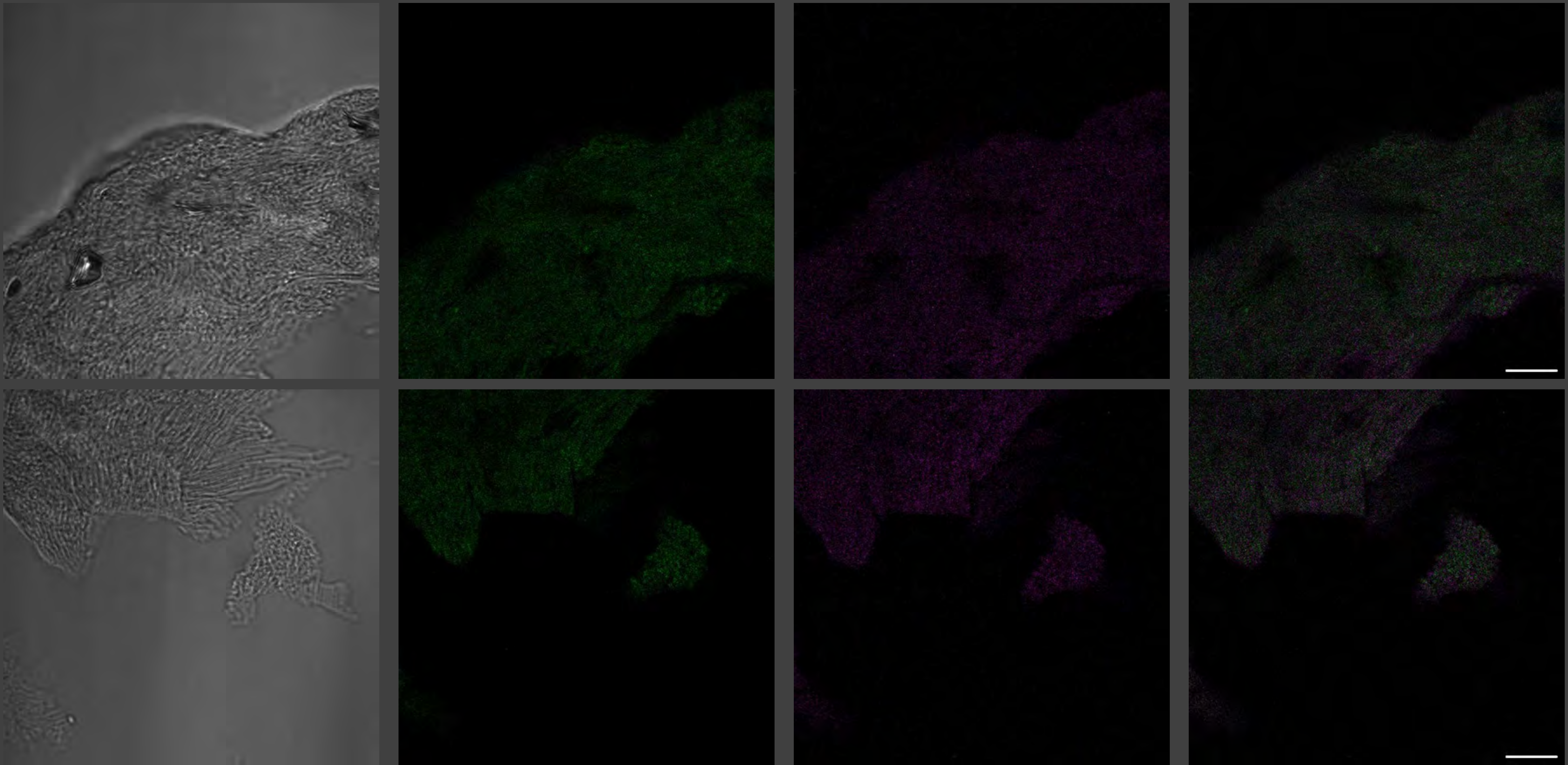

2019-12-12

SY421 P_{bacA}-Ypet (*cam*); P_{comQX}-mTurq (*erm*) middle

Brightfield | YPet | mTurq | Merged

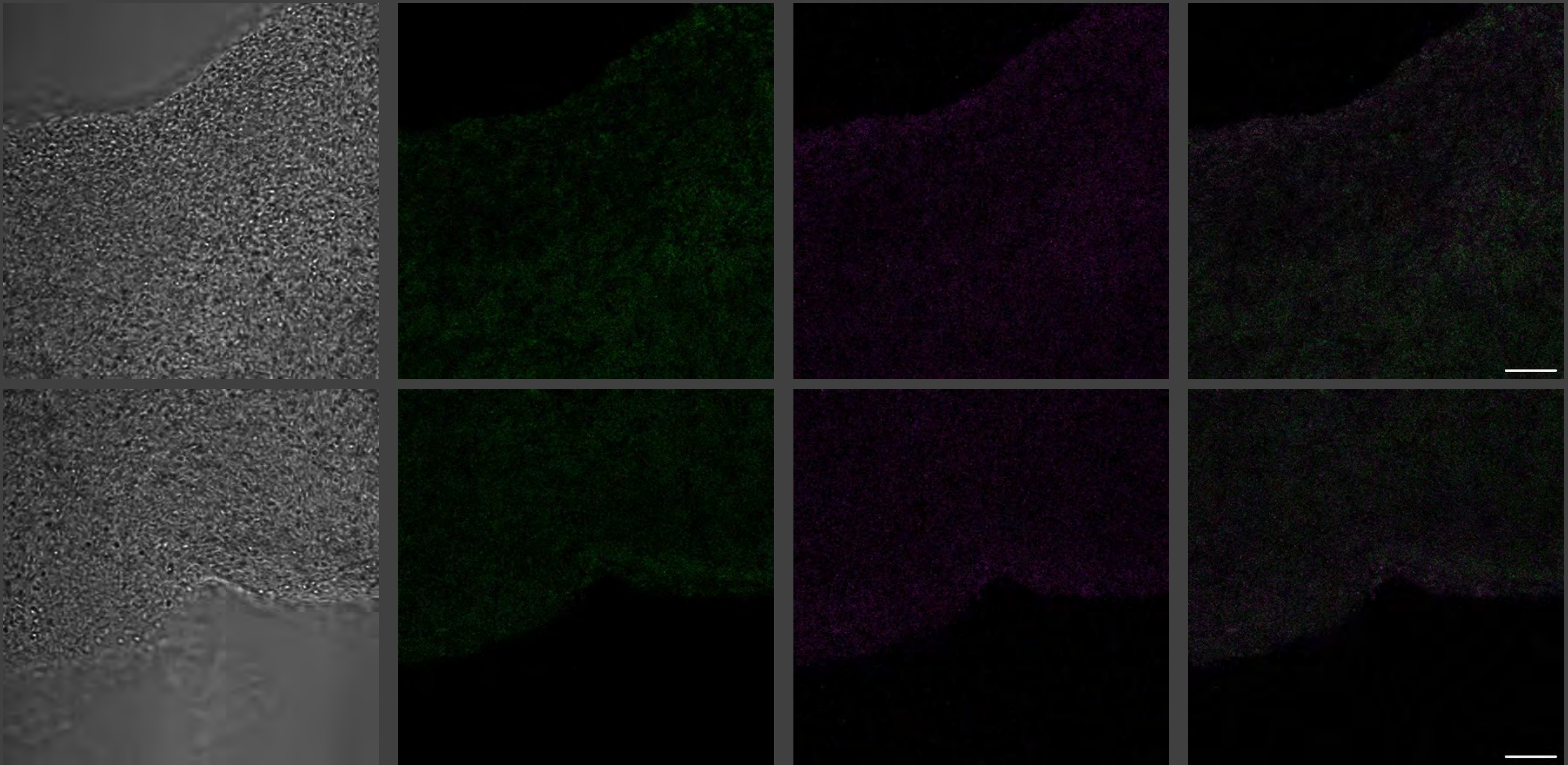

2019-12-12

SY421 P_{bacA}-Ypet (cam); P_{comQX}-mTurq (erm) interior

Brightfield | YPet | mTurq | Merged

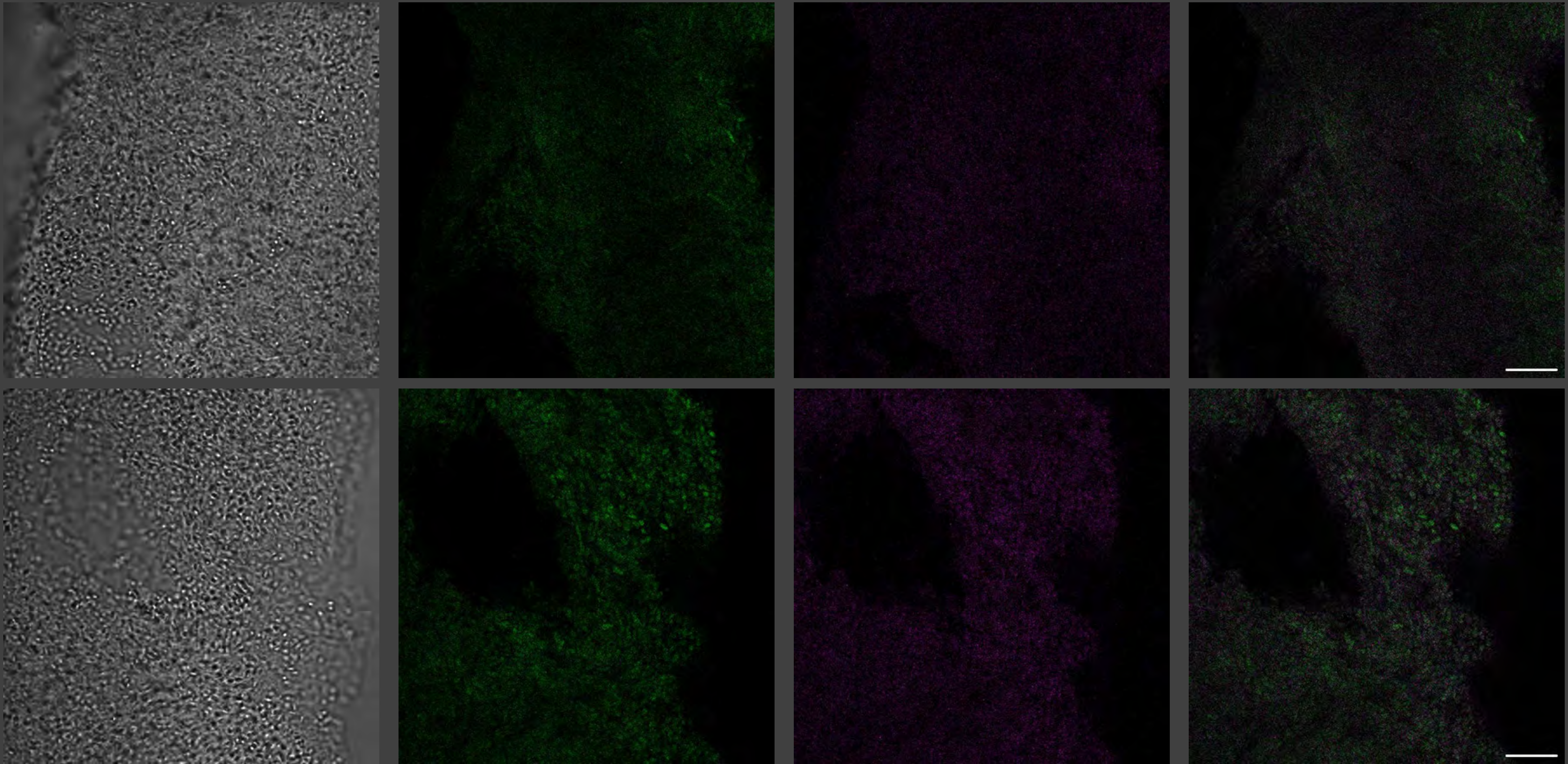

SY398 P_{bacA}-Ypet (*cam*); P_{comQX}-mTurq (*erm*) periphery

2019-12-17

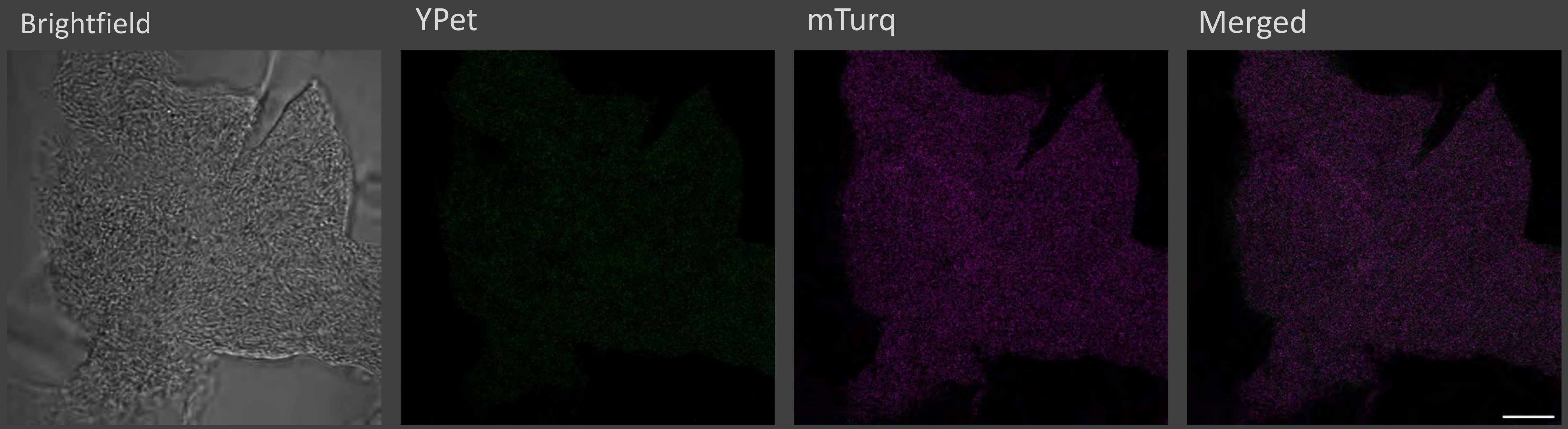

SY398 P_{bacA}-Ypet (*cam*); P_{comQX}-mTurq (*erm*) middle

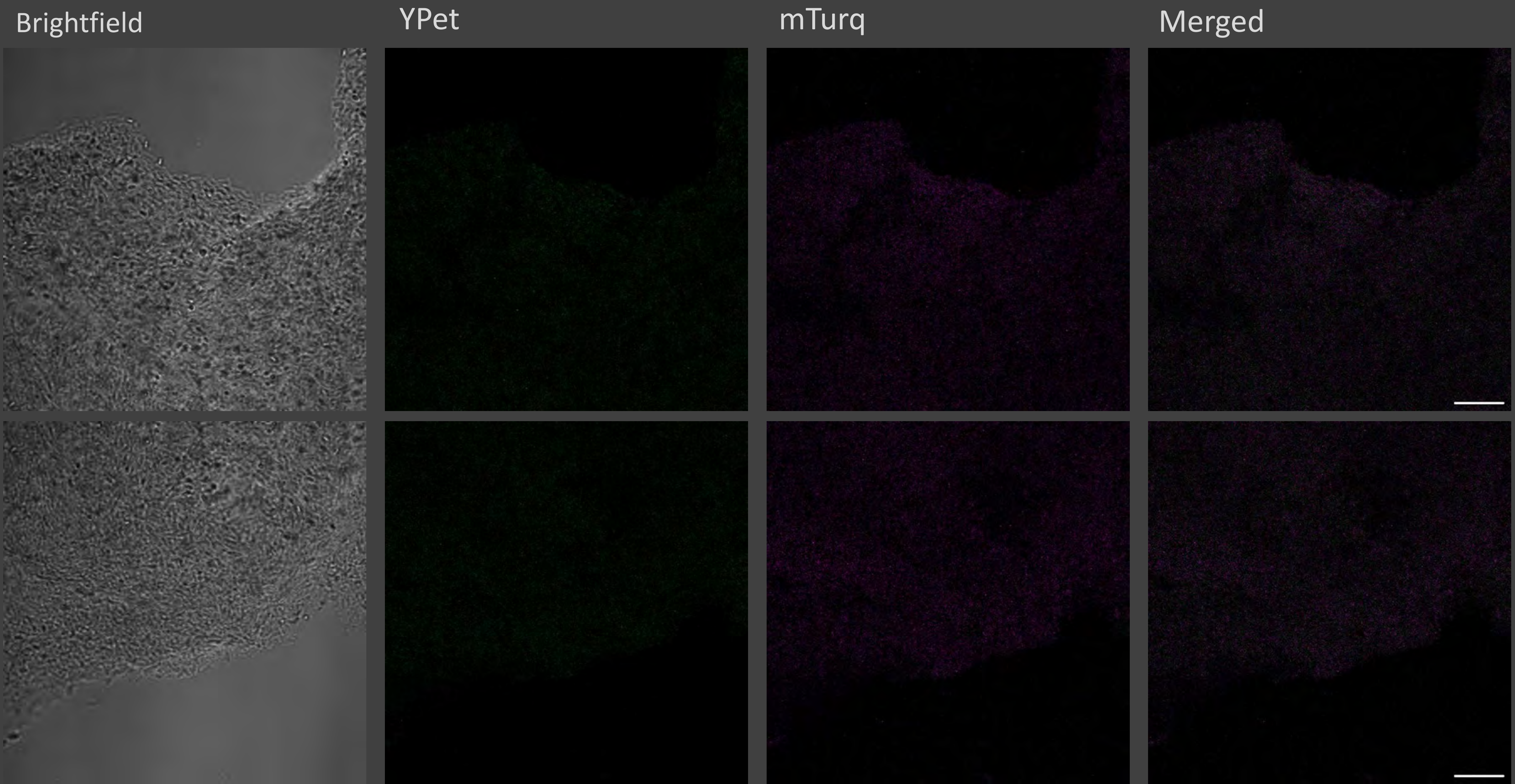

2019-12-17

SY398 P_{bacA}-Ypet (*cam*); P_{comQX}-mTurq (*erm*) interior

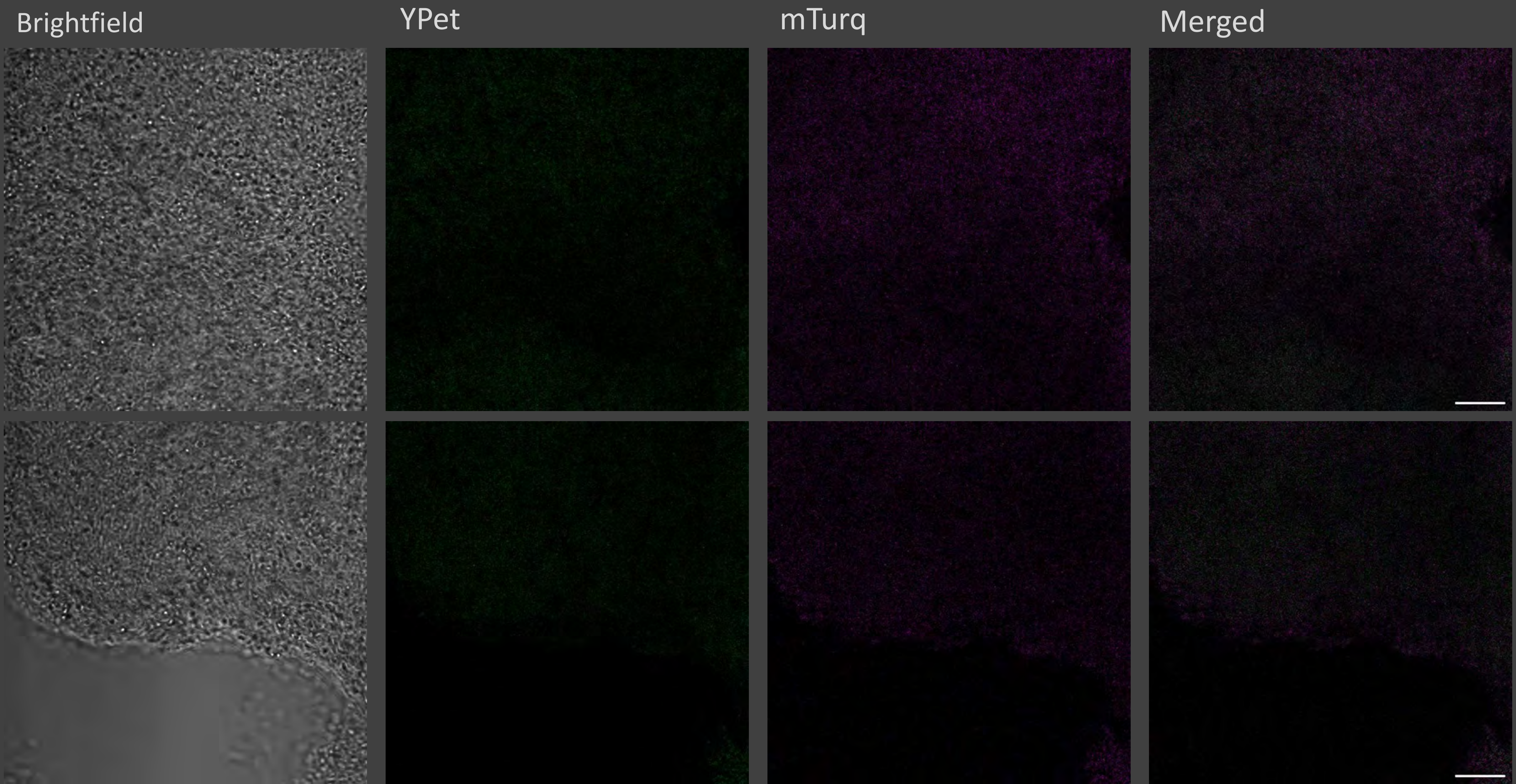

SY465 P_{comQX}-Ypet (*cam*); P_{skfA}-mTurq (*erm*) periphery

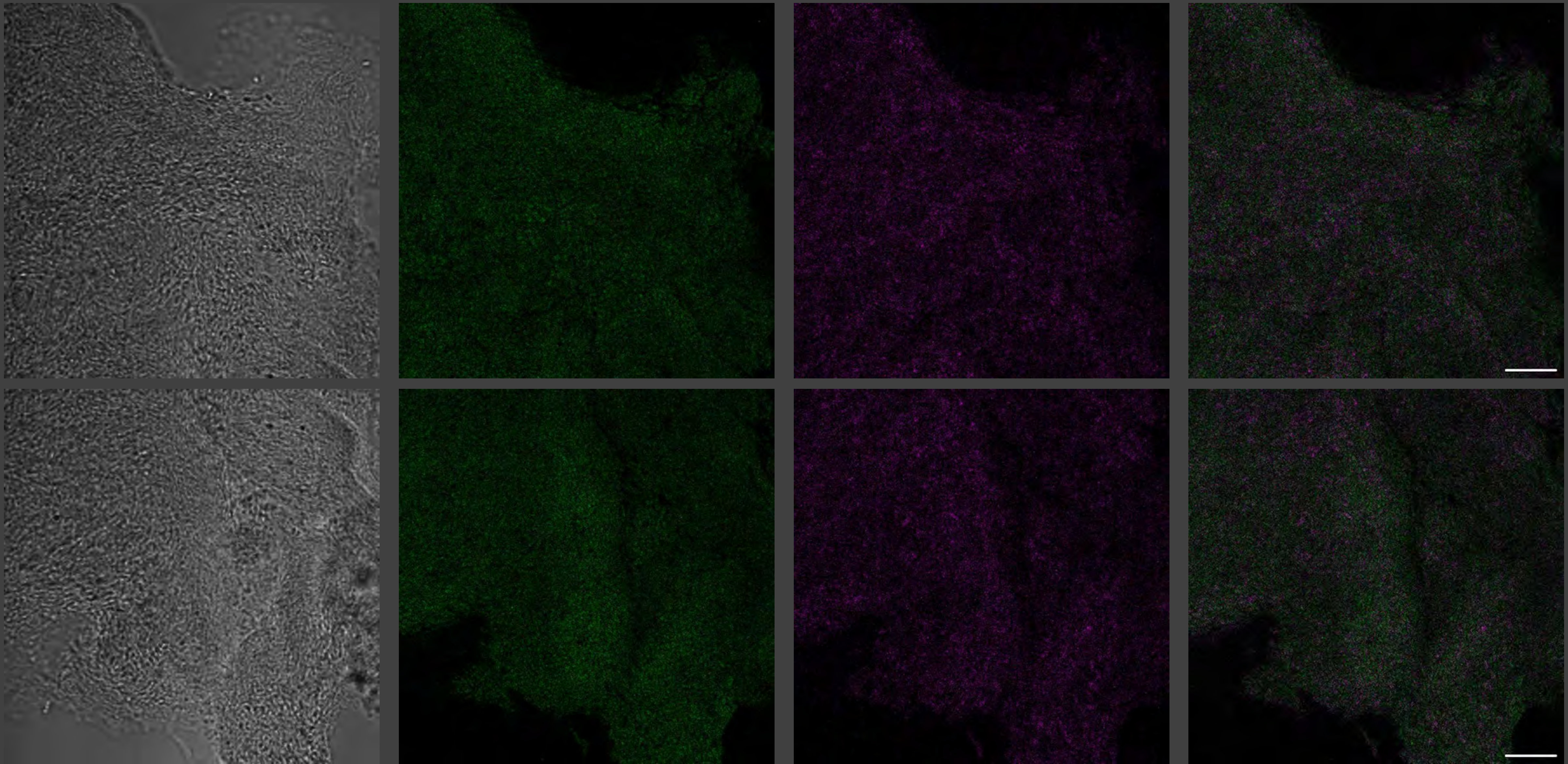

2018-12-18

SY465 P_{comQX}-Ypet (*cam*); P_{skfA}-mTurq (*erm*) middle

Brightfield | YPet | mTurq | Merged

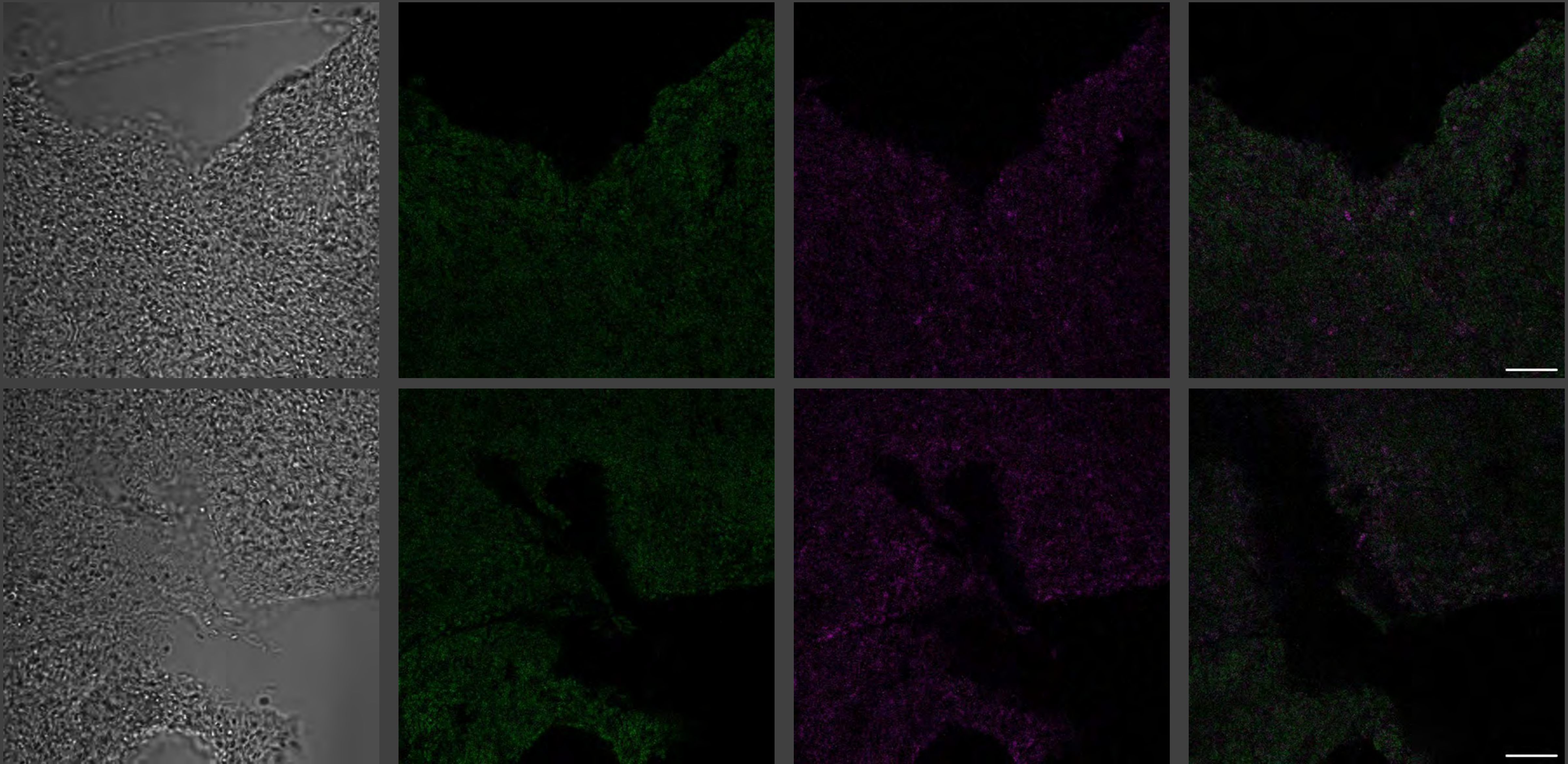

SY465 P_{comQX}-Ypet (*cam*); P_{skfA}-mTurq (*erm*) interior

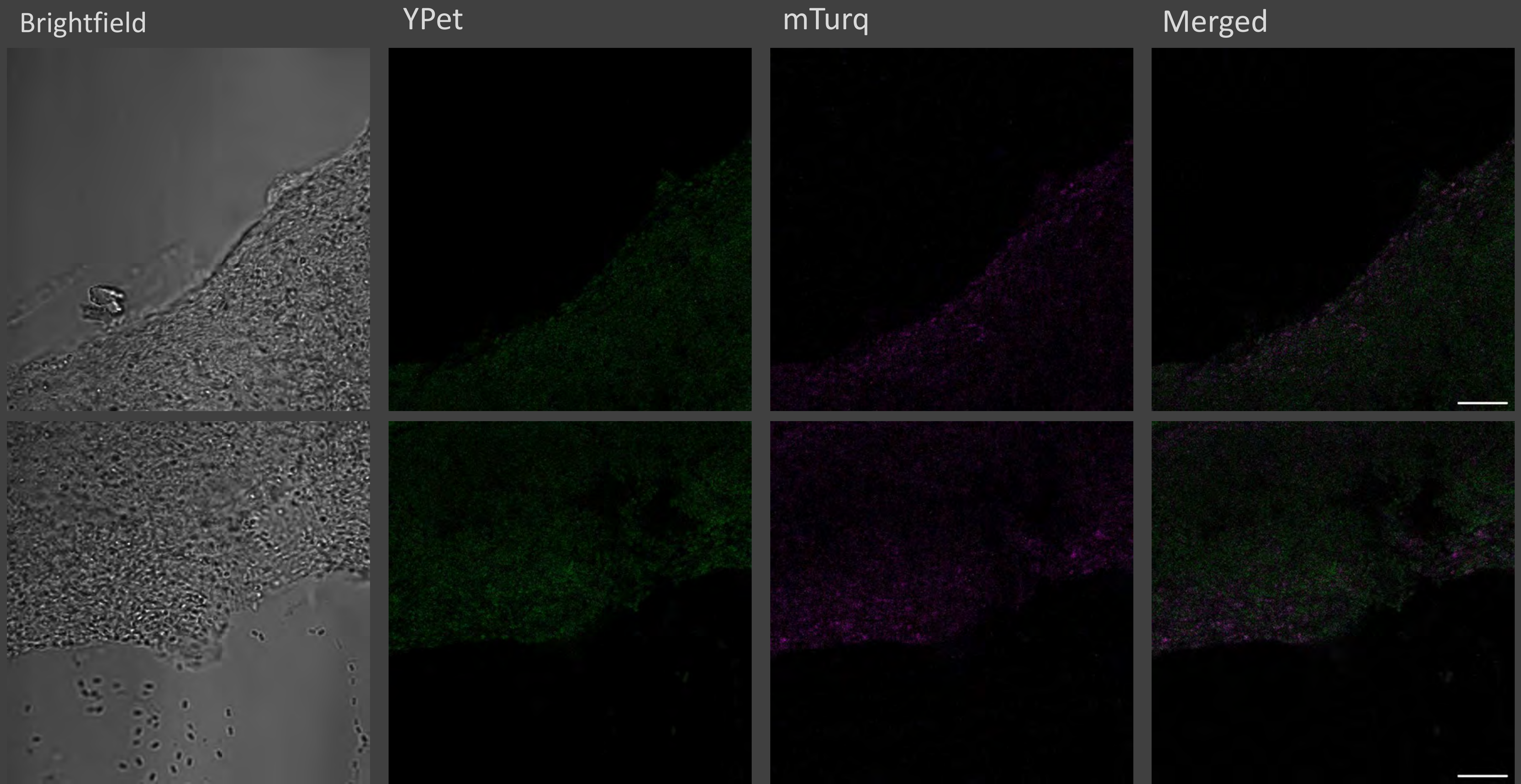

2019-12-18

SY466 P_{comQX}-Ypet (*cam*); P_{aprE}-mTurq (*erm*) periphery

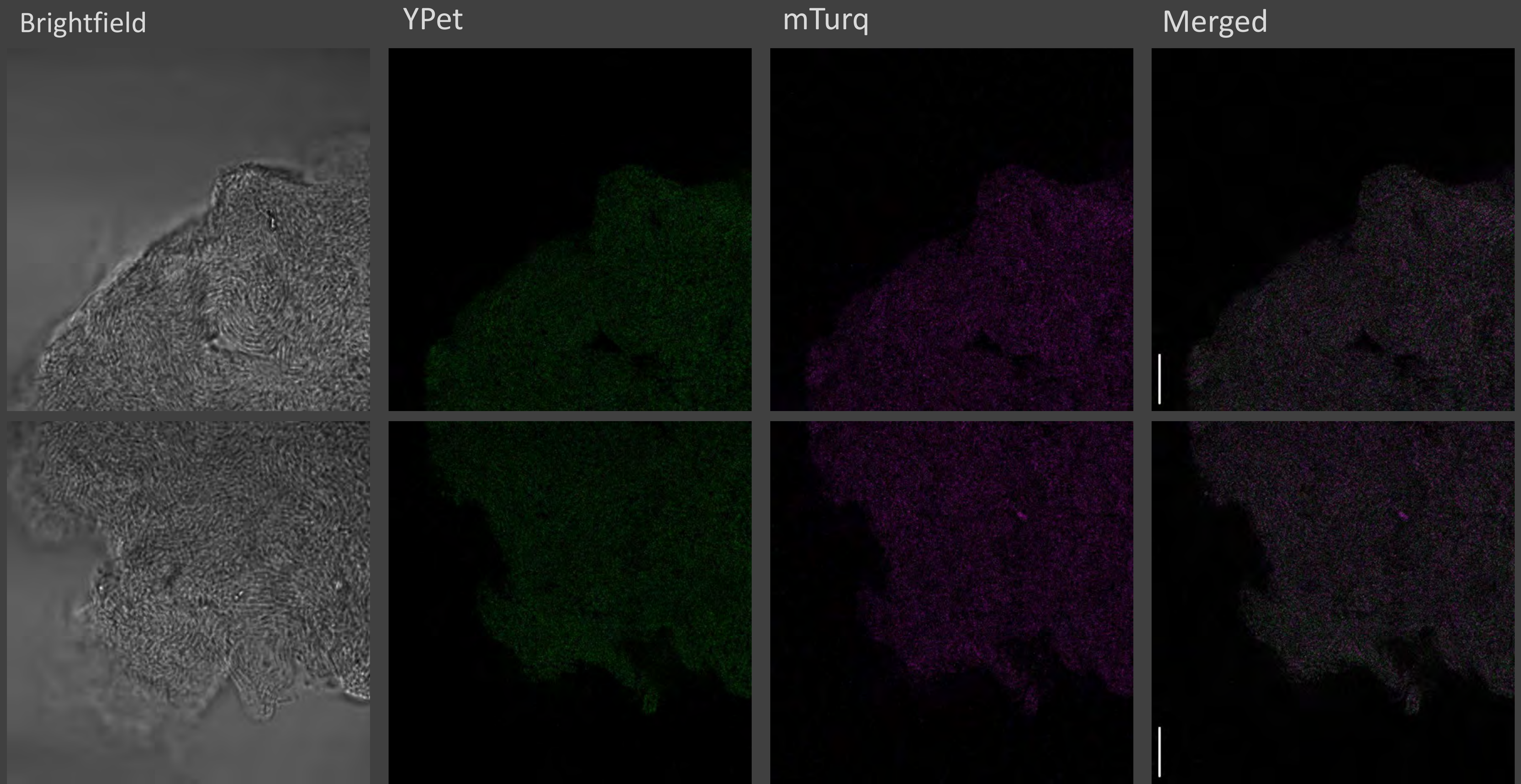

2019-12-18

SY466 P_{comQX}-Ypet (*cam*); P_{aprE}-mTurq (*erm*) middle

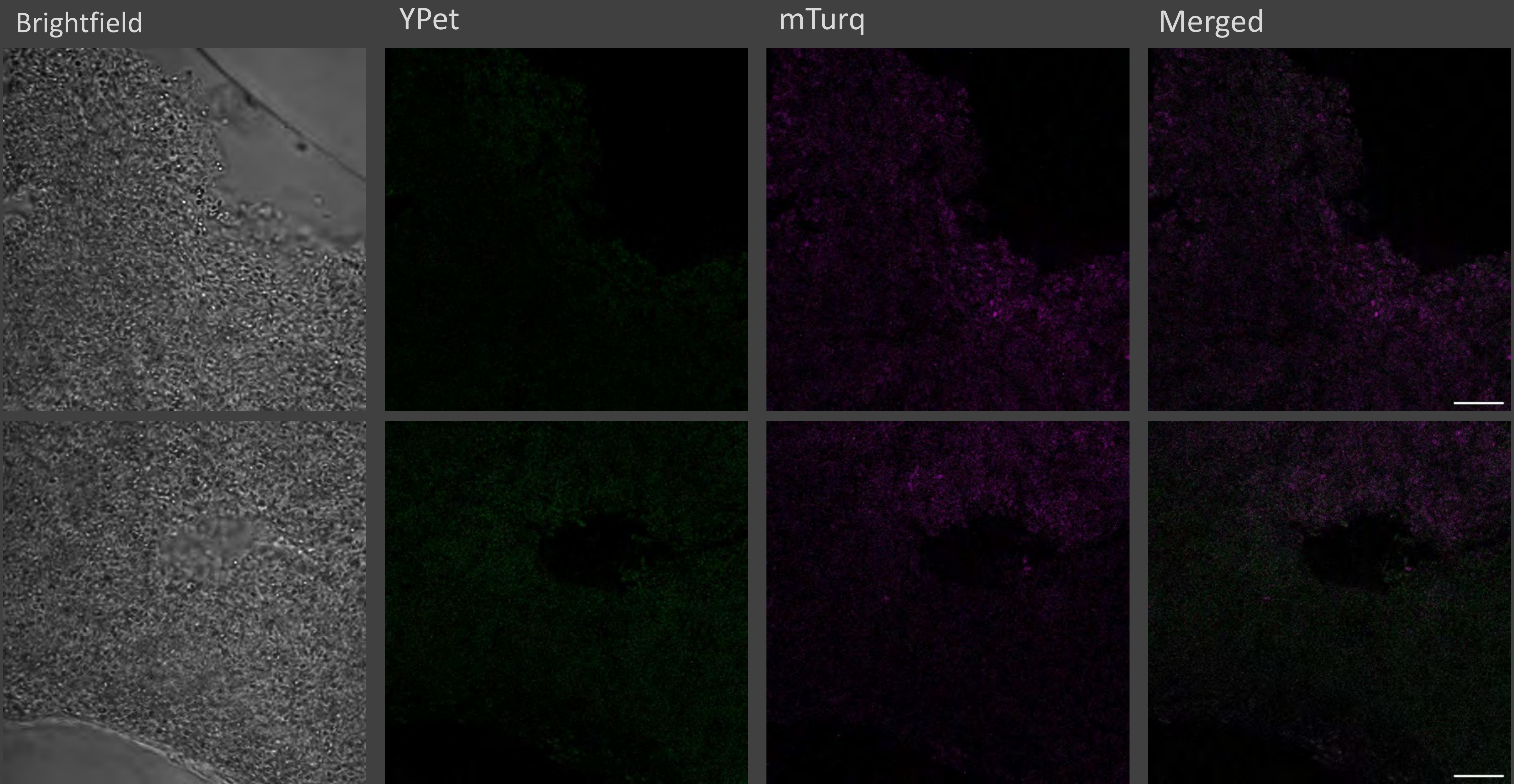

SY466 P_{comQX}-Ypet (*cam*); P_{aprE}-mTurq (*erm*) interior

Brightfield | YPet | mTurq | Merged

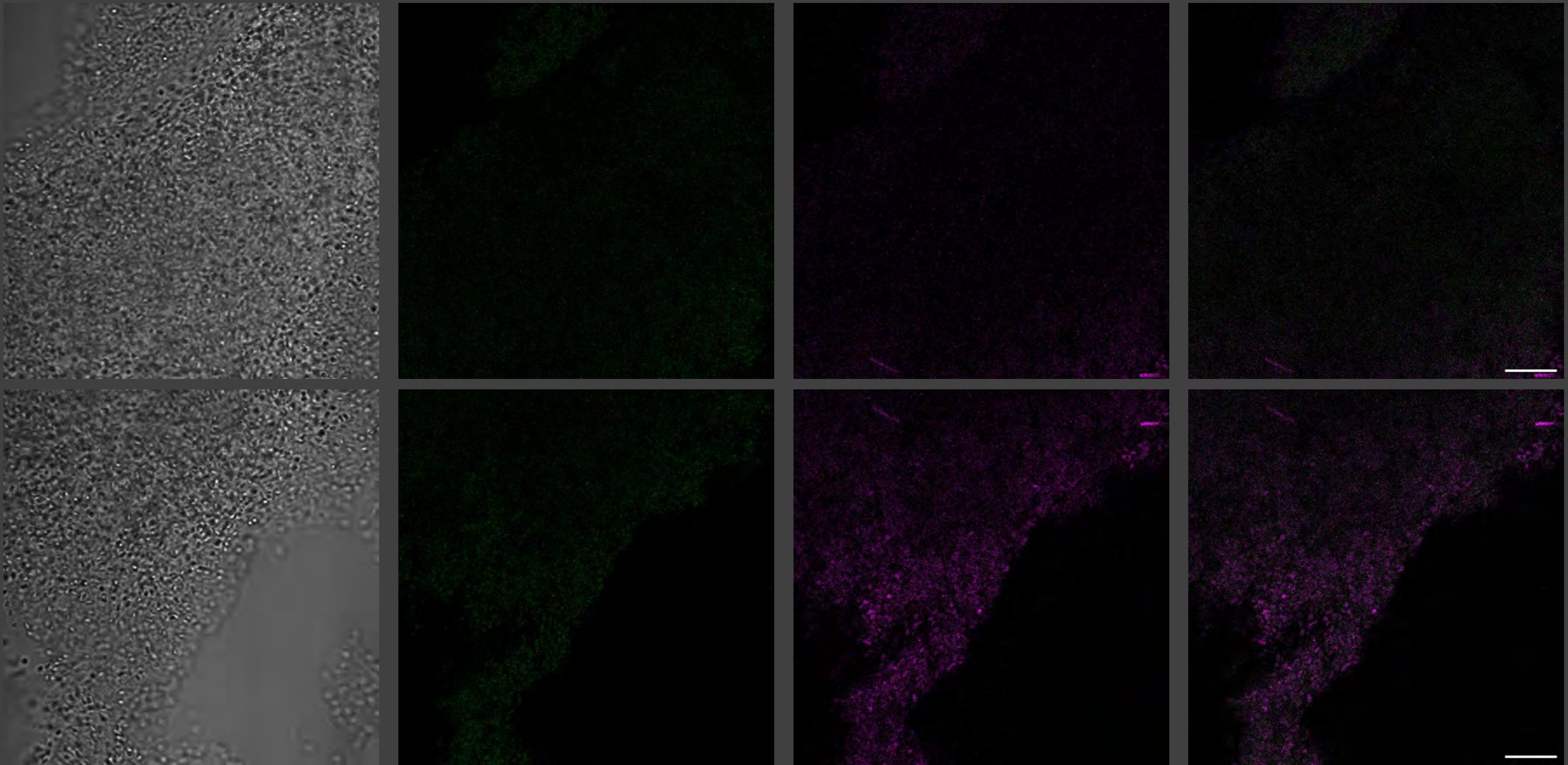

SY313 P_{tapA}-Ypet (*cam*); P_{sdpA}-mTurq (*erm*) periphery

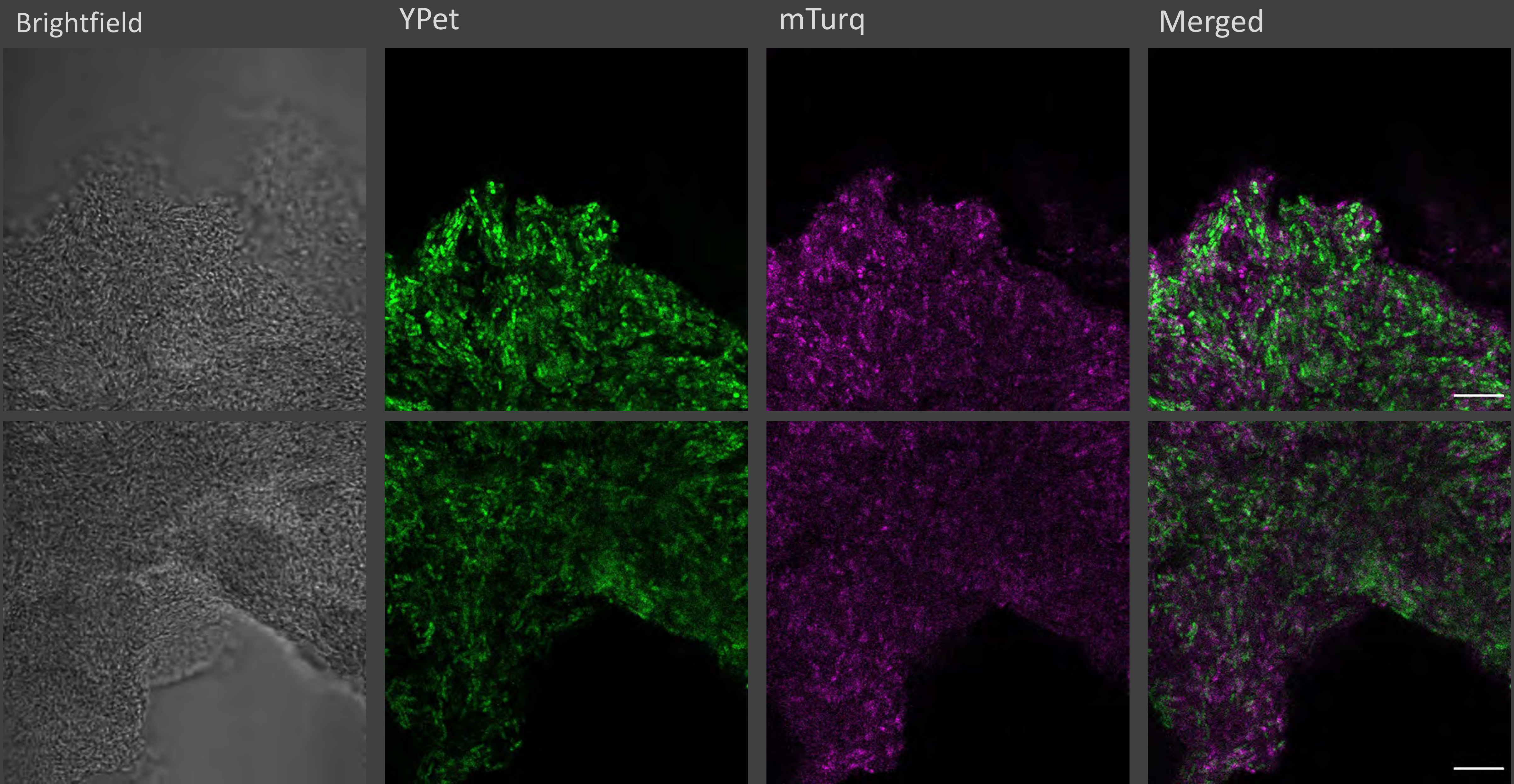

SY313 P_{tapA}-Ypet (*cam*); P_{sdpA}-mTurq (*erm*) middle

Brightfield | YPet | mTurq | Merged

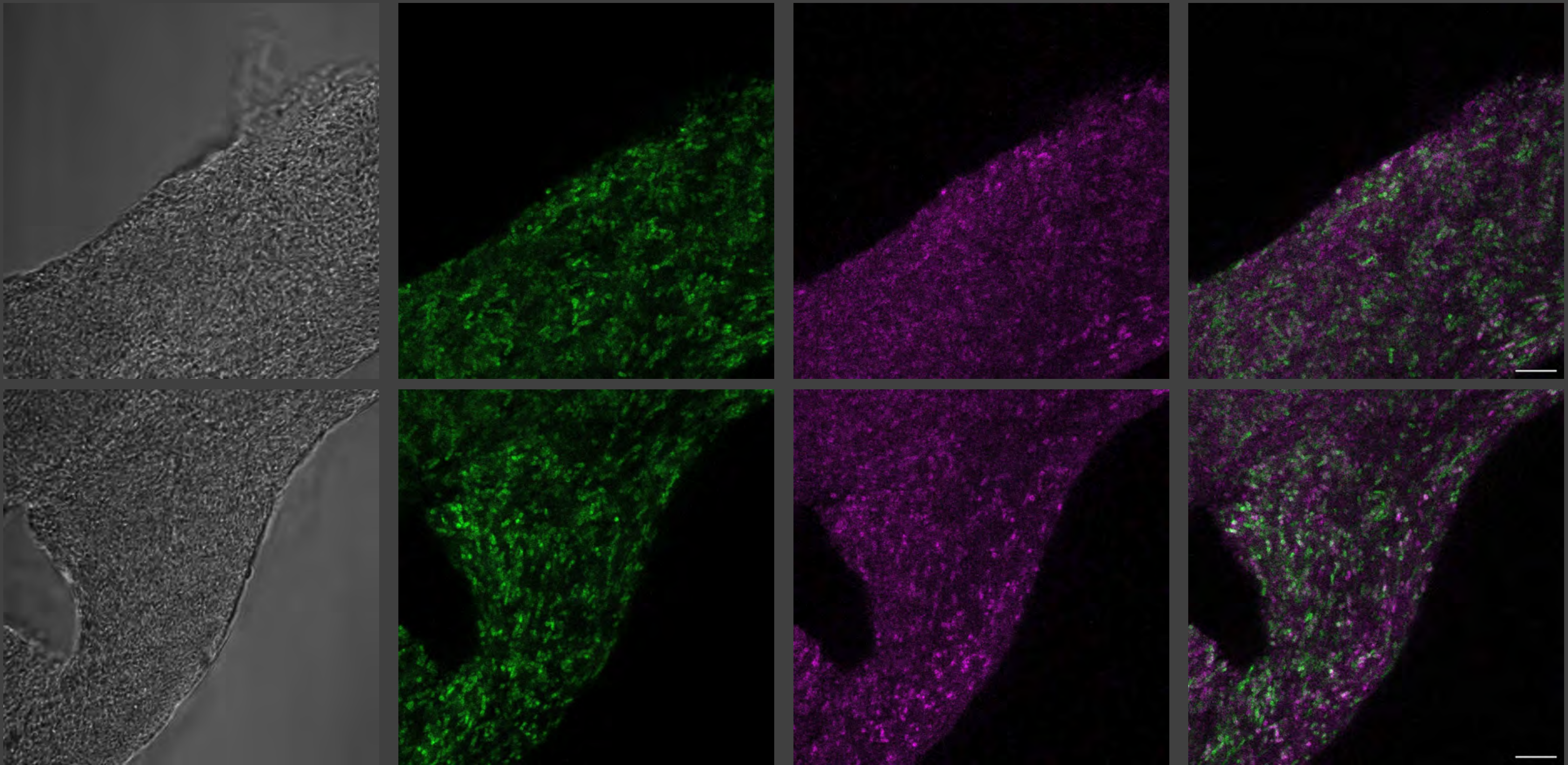

2019-12-30

SY313 $P_{\textit{tapA}}$-Ypet (*cam*); $P_{\textit{sdpA}}$-mTurq (*erm*) interior

Brightfield | YPet | mTurq | Merged

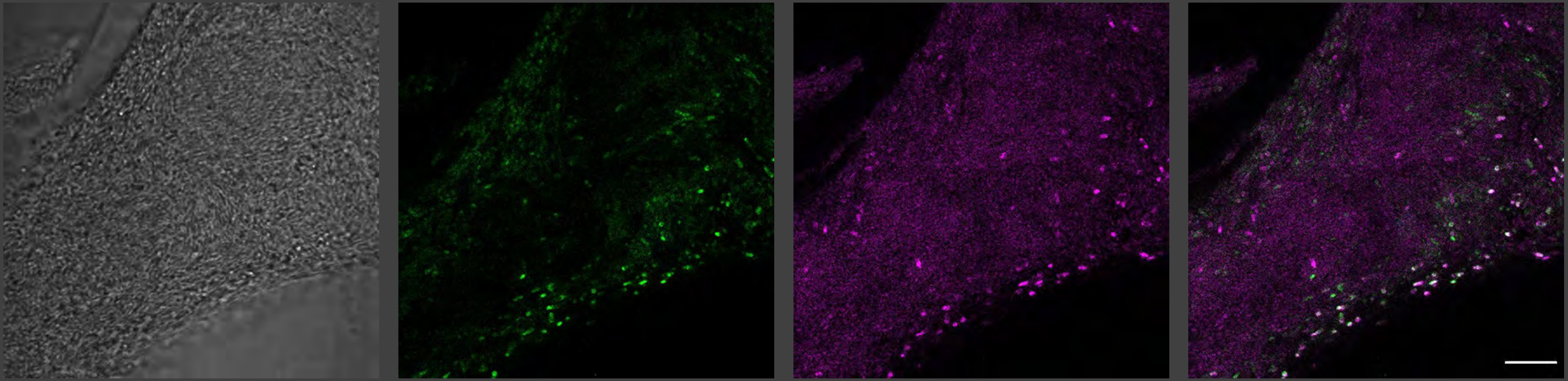

SY329 P_{sspB}-Ypet (*cam*); P_{hag}-mTurq (*erm*) periphery

Brightfield | YPet | mTurq | Merged

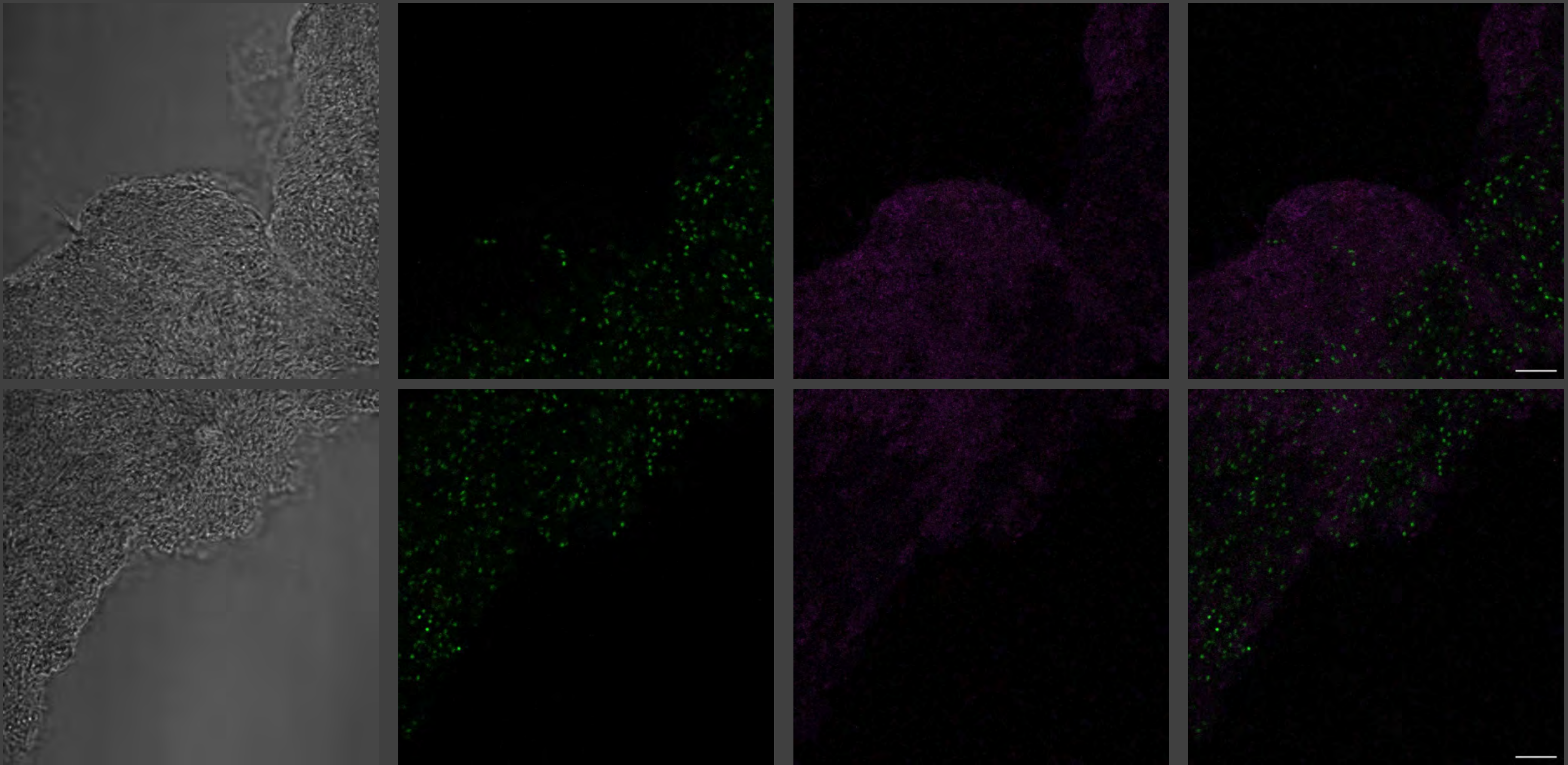

SY329 P_{sspB}-Ypet (*cam*); P_{hag}-mTurq (*erm*) middle

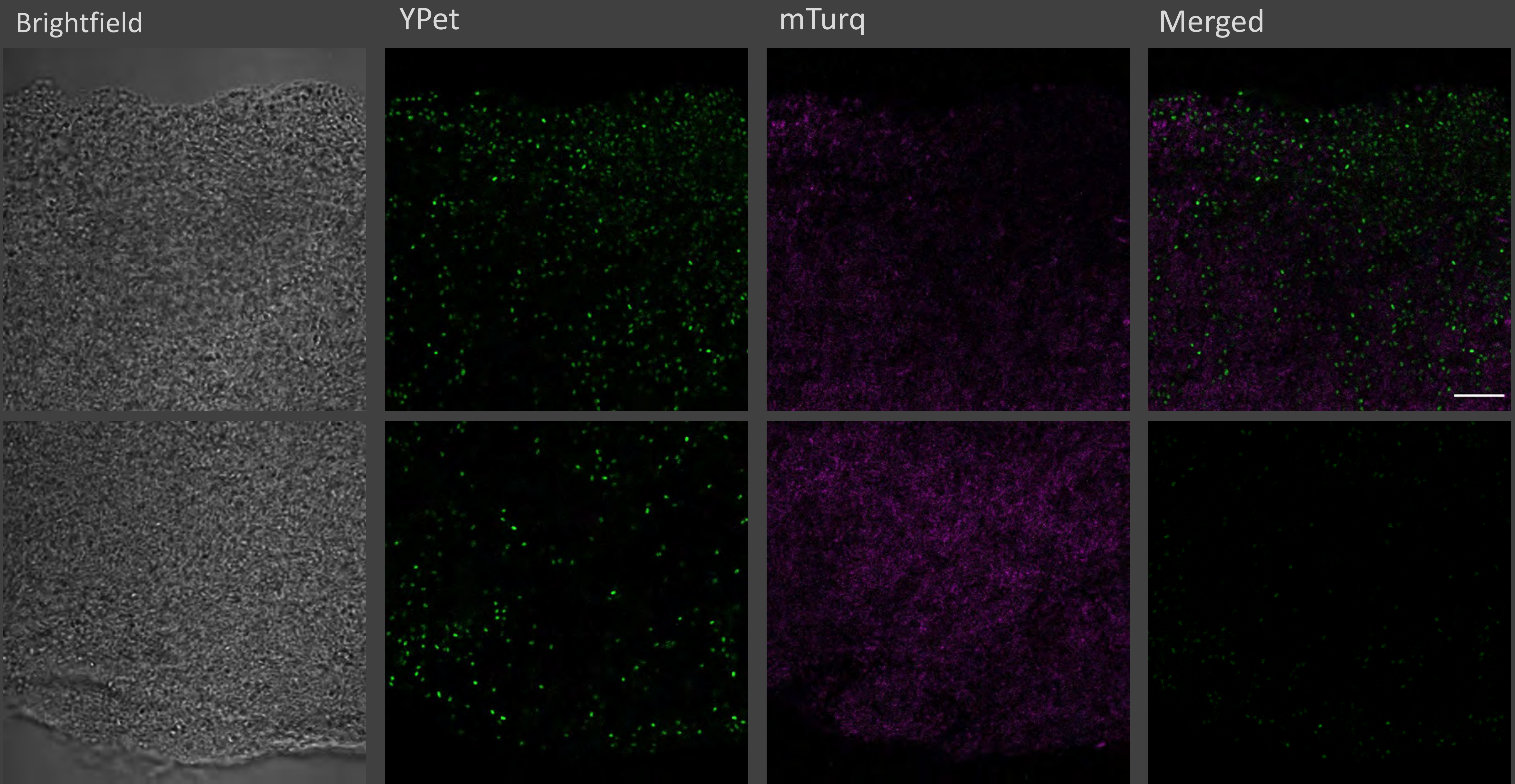

SY329 P_{sspB}-Ypet (*cam*); P_{hag}-mTurq (*erm*) interior

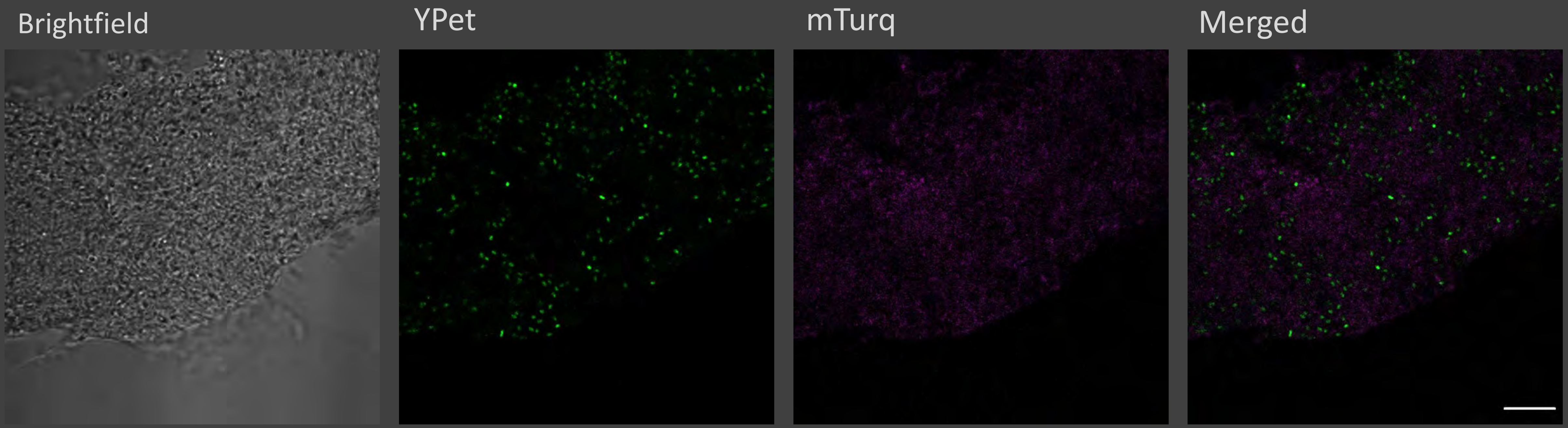

SY330 P_{sspB}-Ypet (*cam*); P_{sdpA}-mTurq (*erm*) periphery

2019-12-31

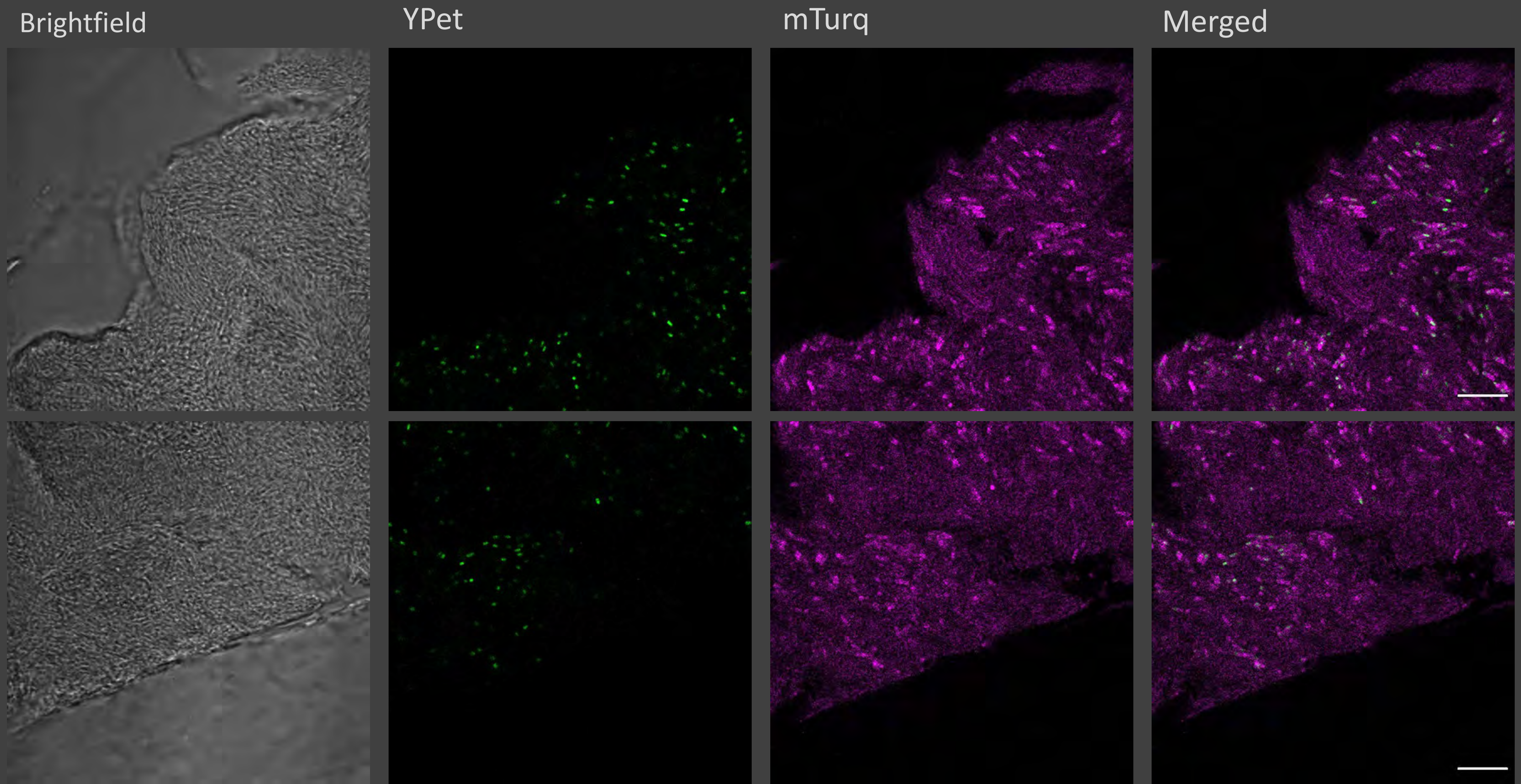

SY330 P_{sspB}-Ypet (*cam*); P_{sdpA}-mTurq (*erm*) middle

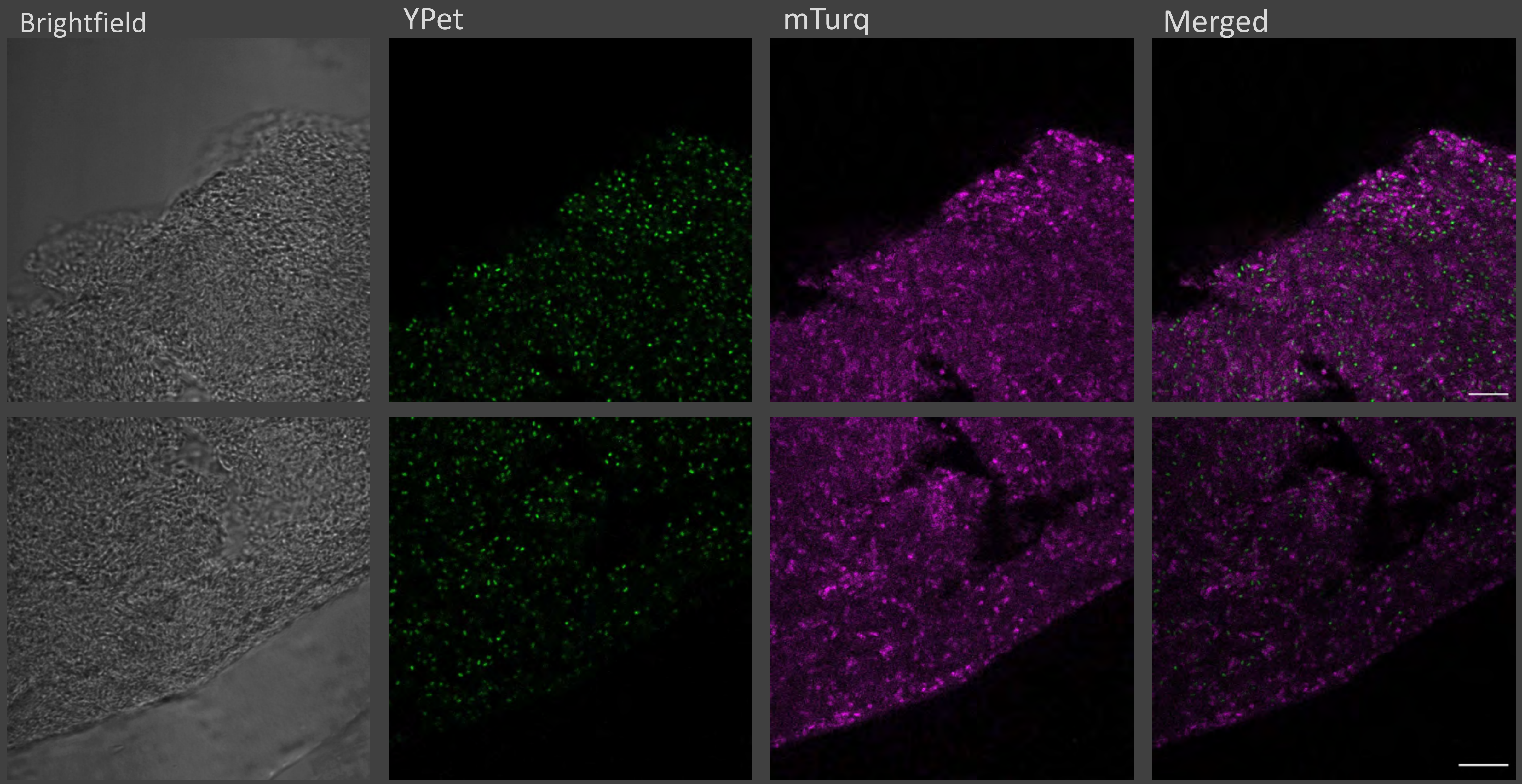

2019-12-31

SY330 P_{sspB}-Ypet (*cam*); P_{sdpA}-mTurq (*erm*) interior

Brightfield | YPet | mTurq | Merged

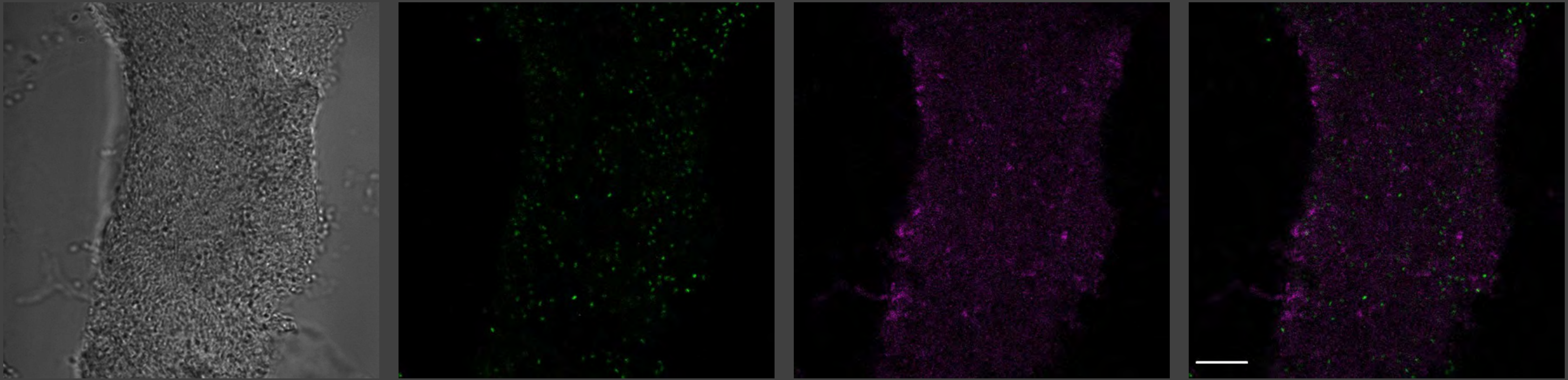

SY340 P_{sspB}-Ypet (*cam*); P_{comQX}-mTurq (*erm*) periphery

2019-12-31

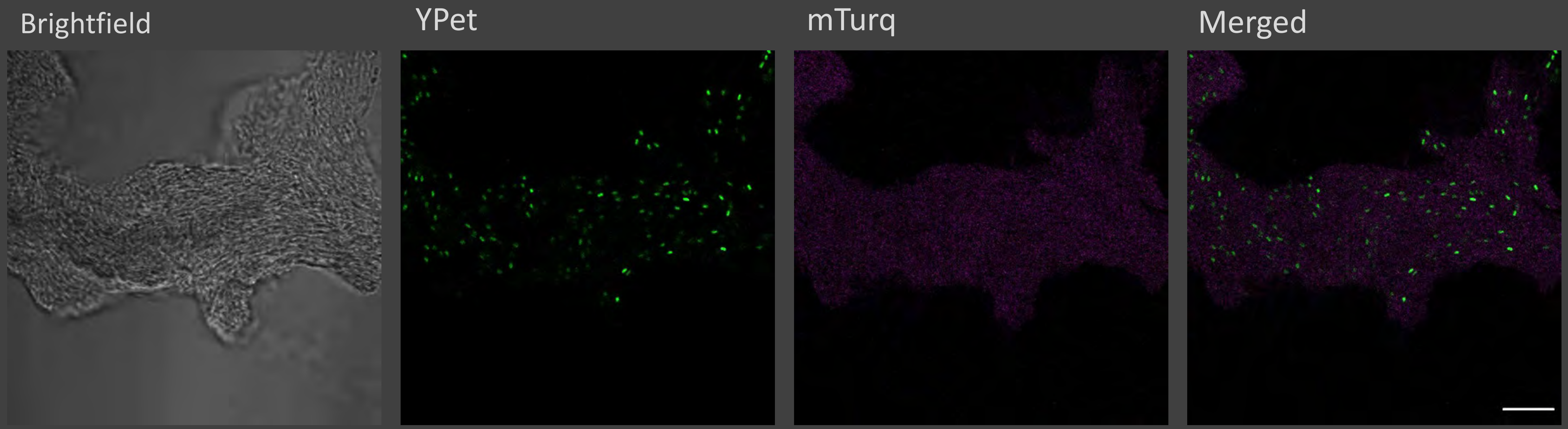

SY340 P_{sspB}-Ypet (*cam*); P_{comQX}-mTurq (*erm*) middle

2019-12-31

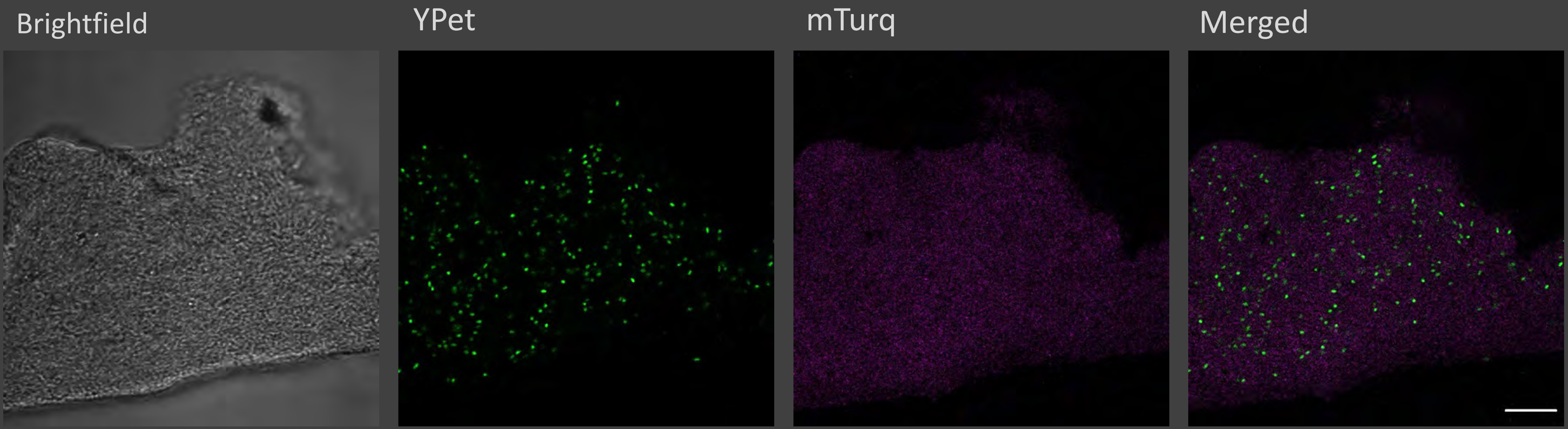

2019-12-31

SY340 P_{sspB}-Ypet (*cam*); P_{comQX}-mTurq (*erm*) interior

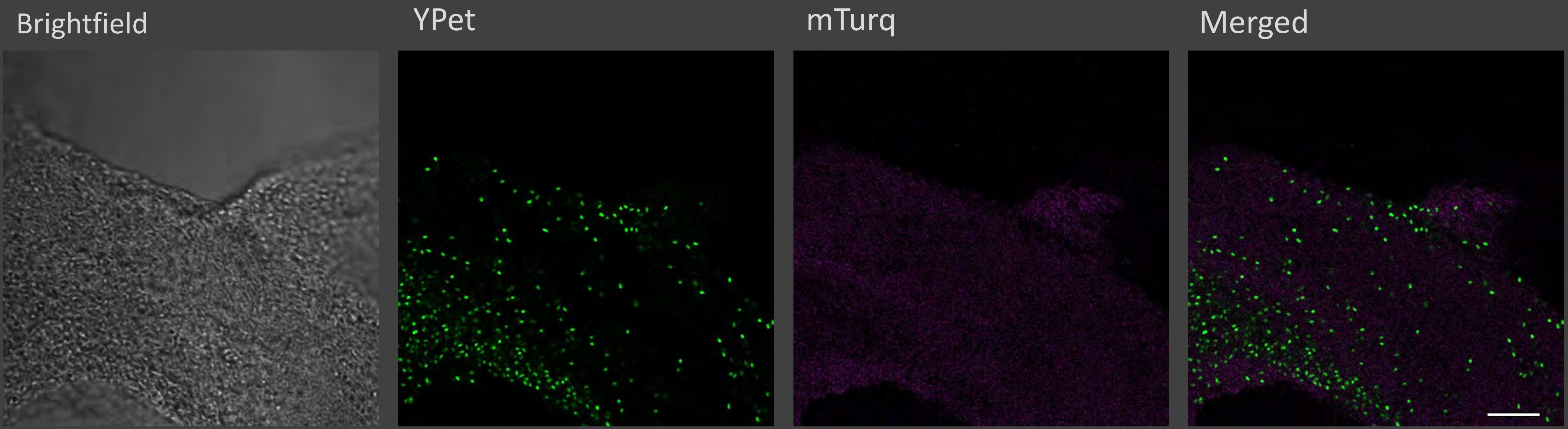

SY345 P_{sspB}-Ypet (*cam*); P_{aprE}-mTurq (*erm*) periphery

2019-12-31

Brightfield | YPet | mTurq | Merged

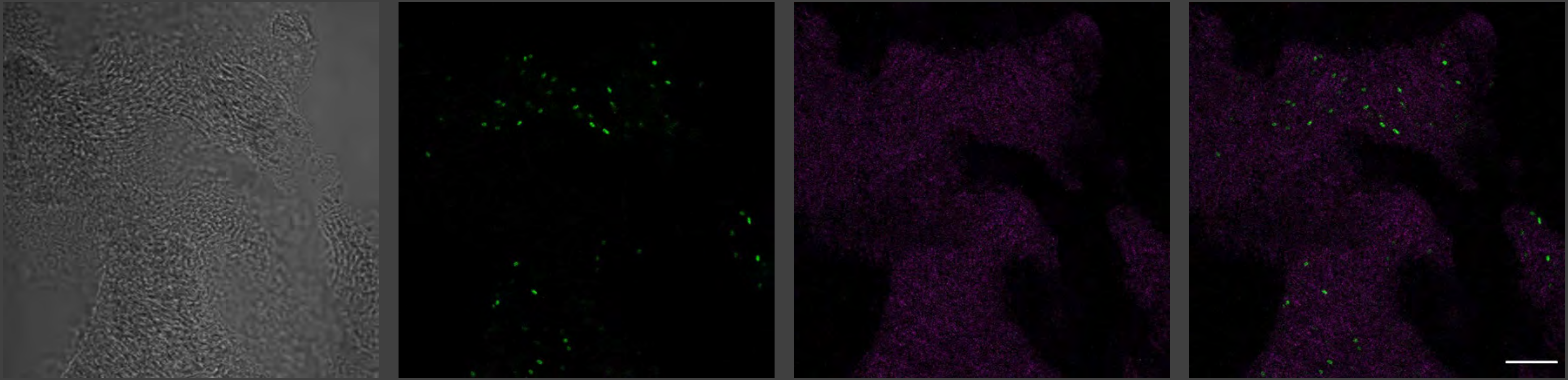

SY345 P_{sspB}-Ypet (*cam*); P_{aprE}-mTurq (*erm*) middle

Brightfield | YPet | mTurq | Merged

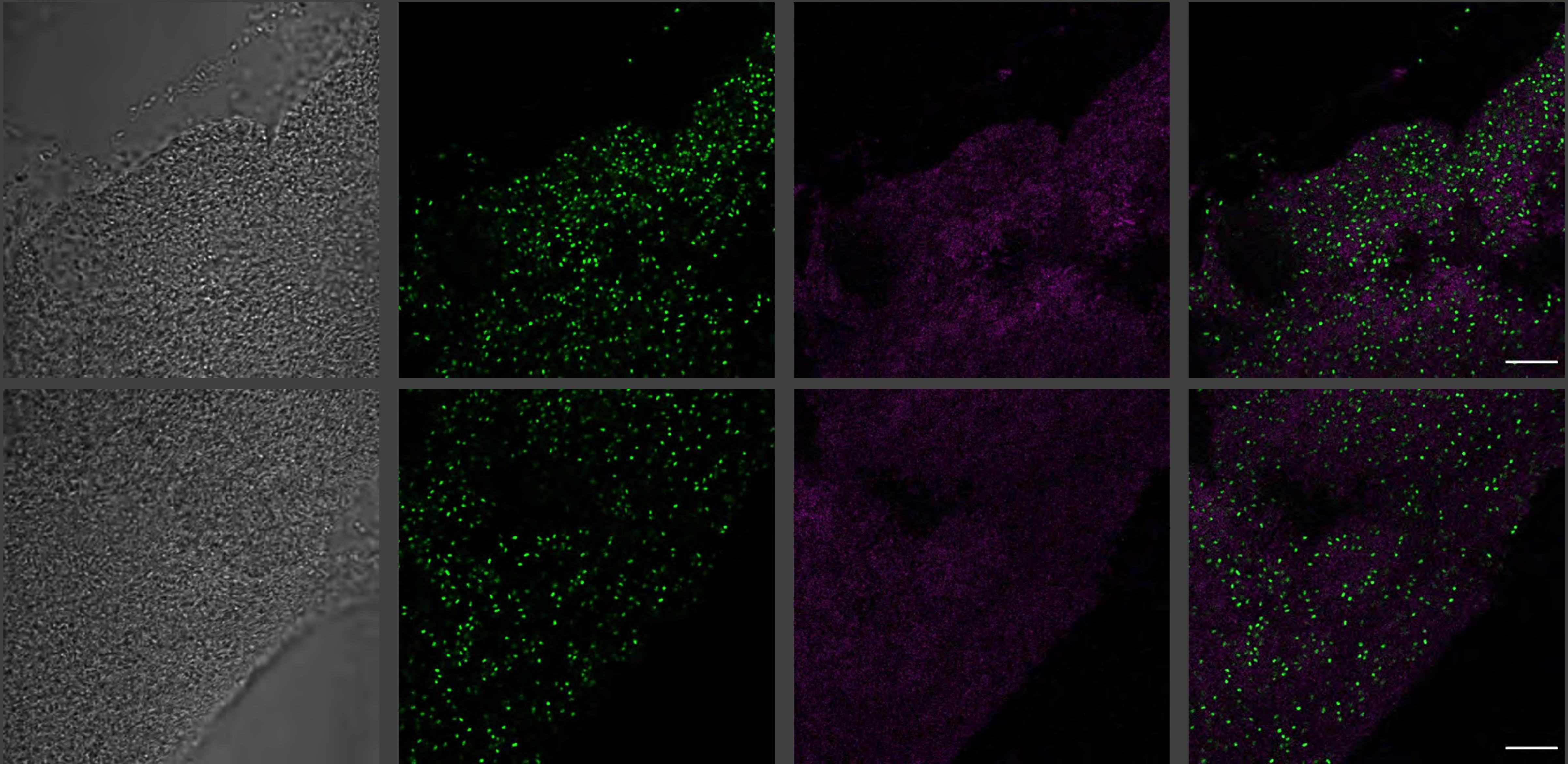

SY345 P_{sspB}-Ypet (*cam*); P_{aprE}-mTurq (*erm*) interior

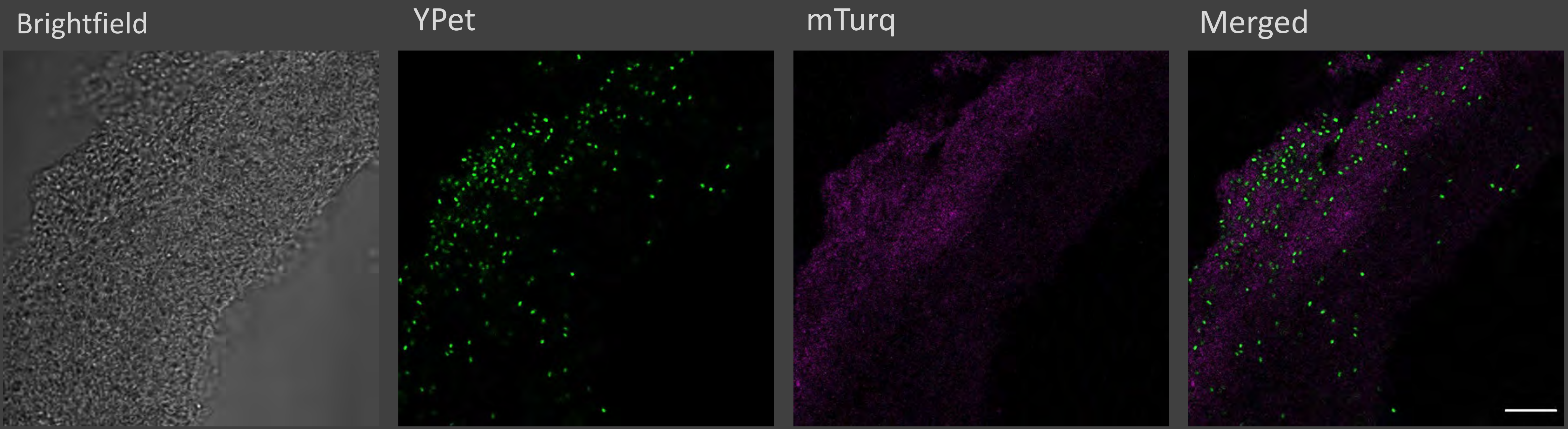

SY350 P_{hag}-Ypet (*cam*); P_{pksC}-mTurq (*erm*) periphery

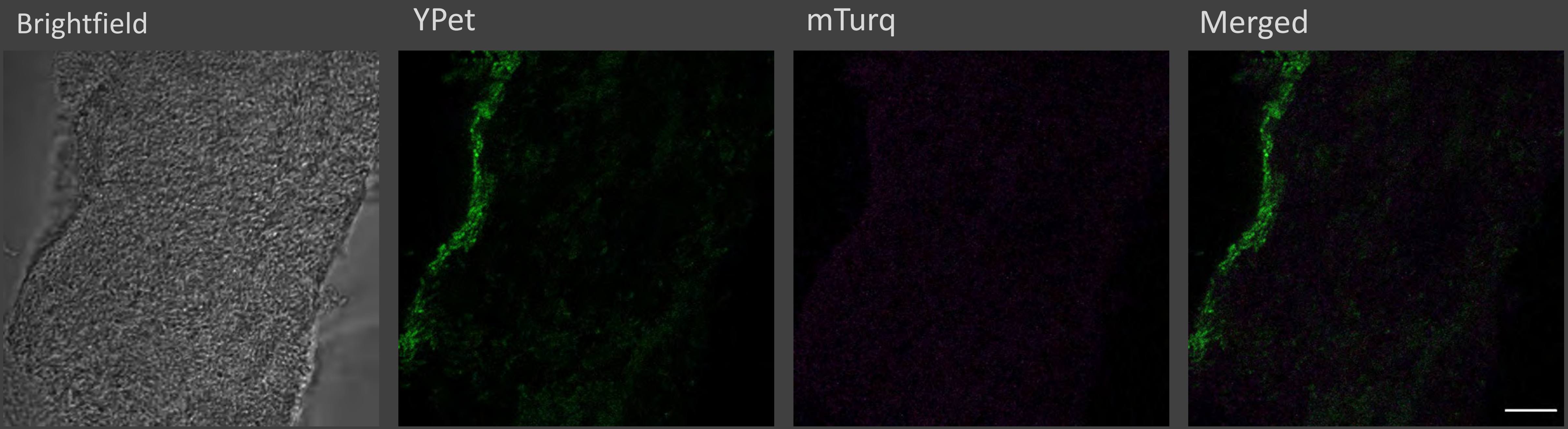

2020-01-09

SY350 P_{hag}-Ypet (*cam*); P_{pksC}-mTurq (*erm*) middle

Brightfield

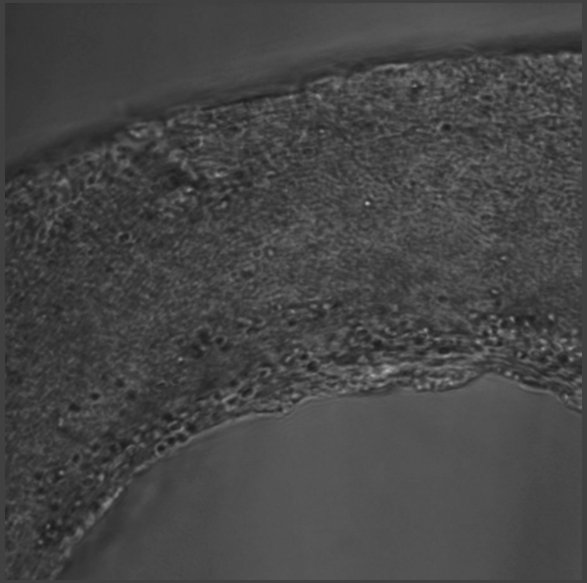

YPet

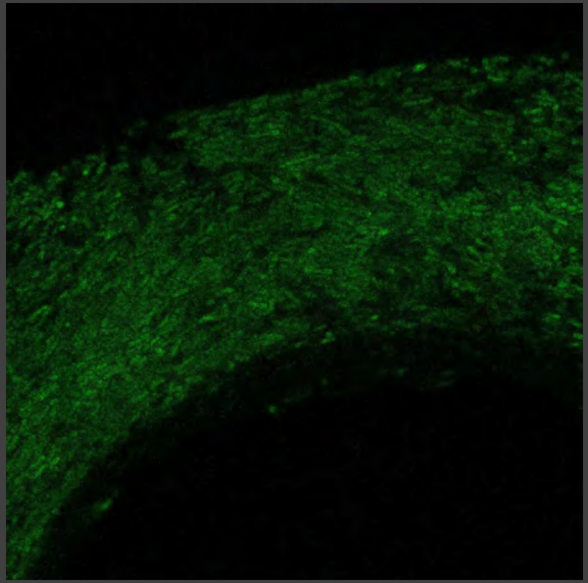

mTurq

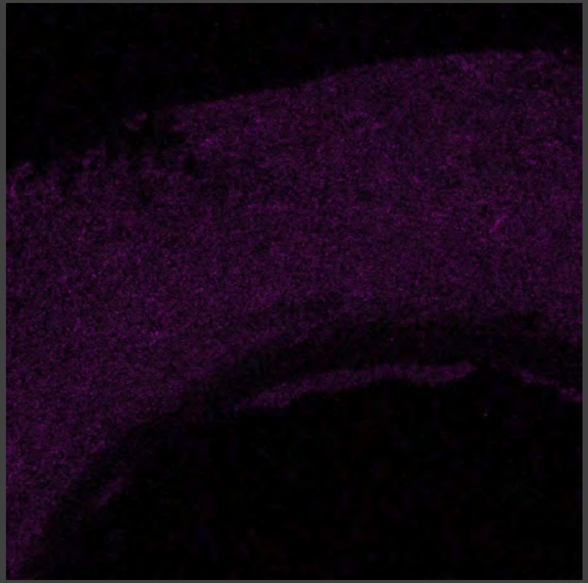

Merged

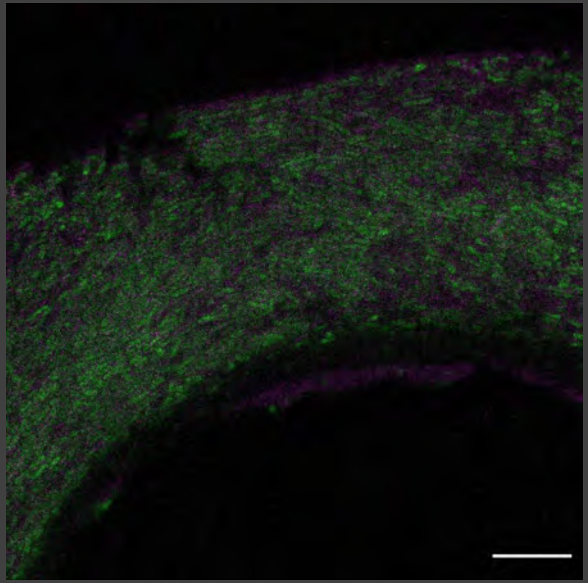

SY350 P_{hag}-Ypet (*cam*); P_{pksC}-mTurq (*erm*) interior

Brightfield YPet mTurq Merged

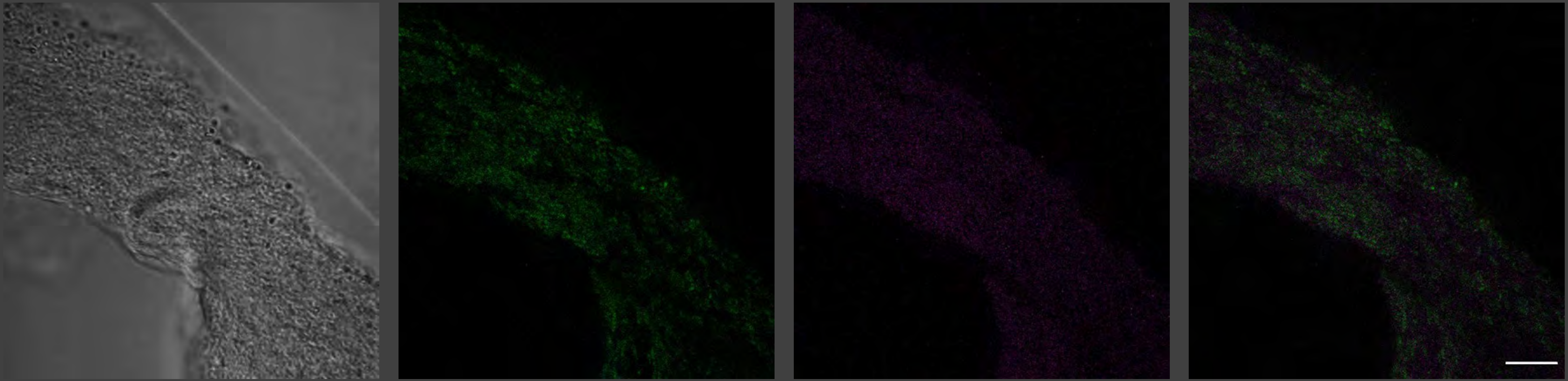

SY351 P_{hag}-Ypet (*cam*); P_{bacA}-mTurq (*erm*) periphery

Brightfield | YPet | mTurq | Merged

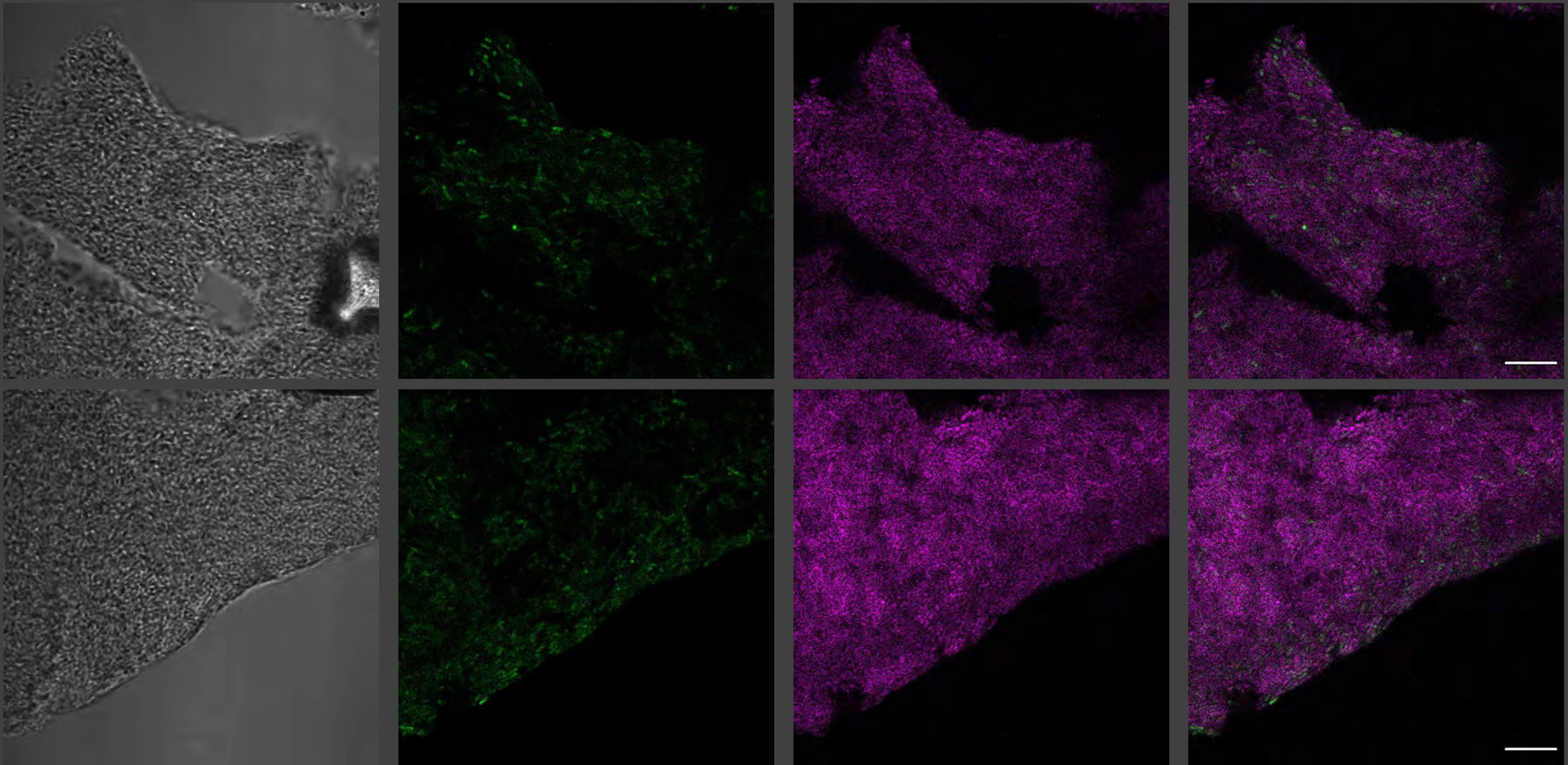

SY351 P_{hag}-Ypet (*cam*); P_{bacA}-mTurq (*erm*) middle

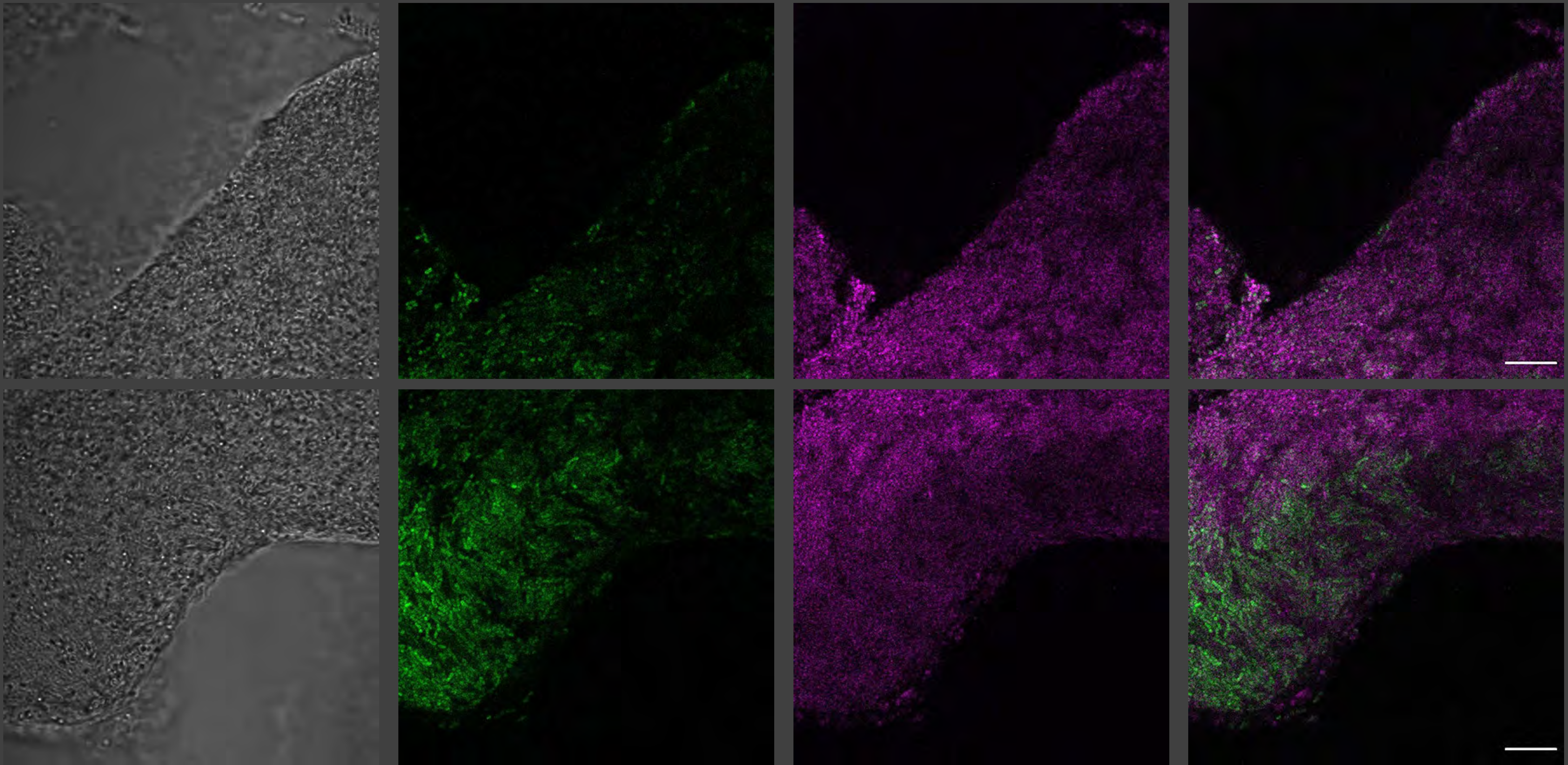

2020-01-09

SY351 P_{hag}-Ypet (*cam*); P_{bacA}-mTurq (*erm*) interior

Brightfield | YPet | mTurq | Merged

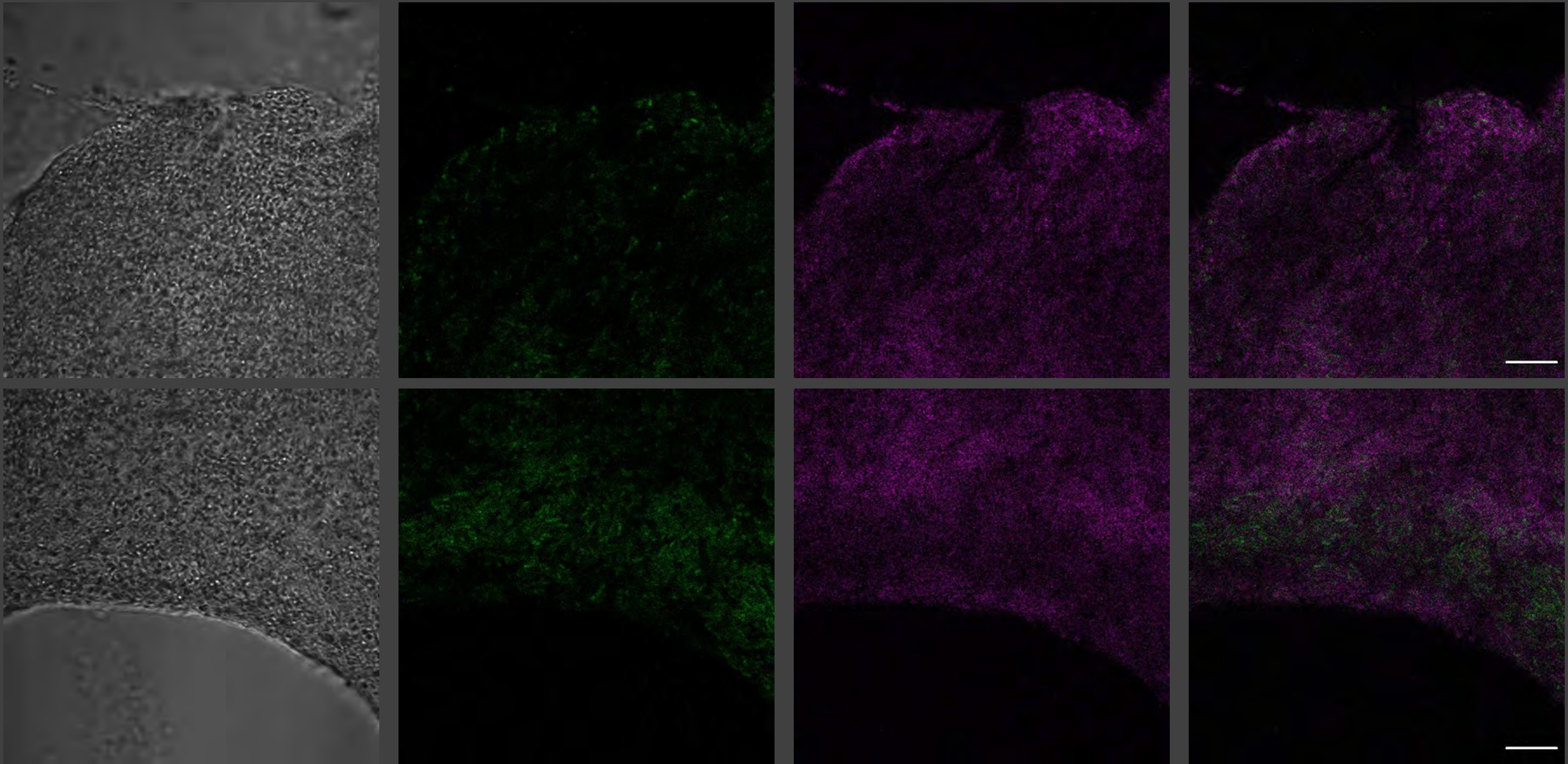

SY365 P_{sdpA}-Ypet (*cam*); P_{pksC}-mTurq (*erm*) periphery

2020-01-09

Brightfield | YPet | mTurq | Merged

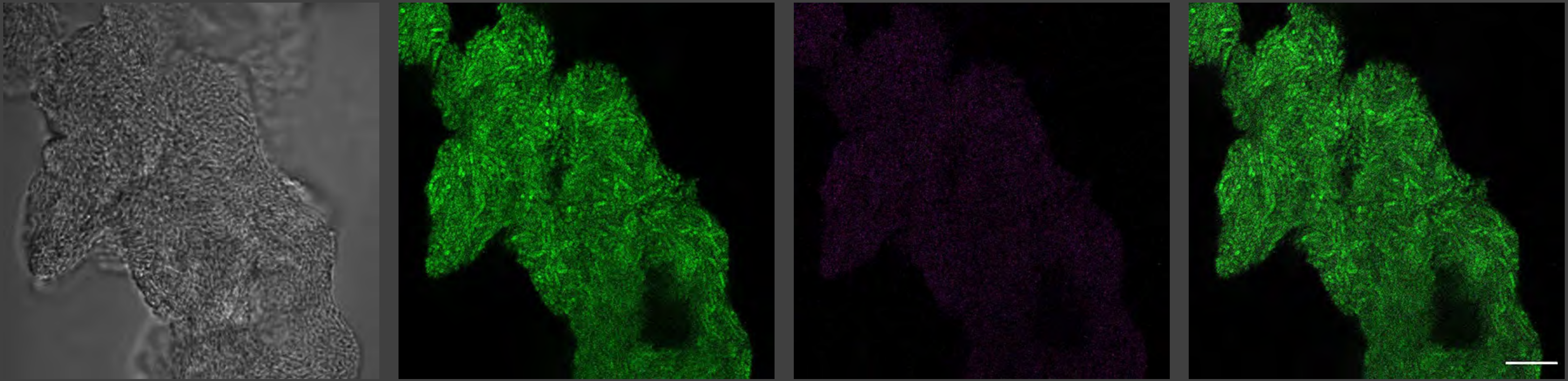

SY365 P_{sdpA}-Ypet (*cam*); P_{pksC}-mTurq (*erm*) middle

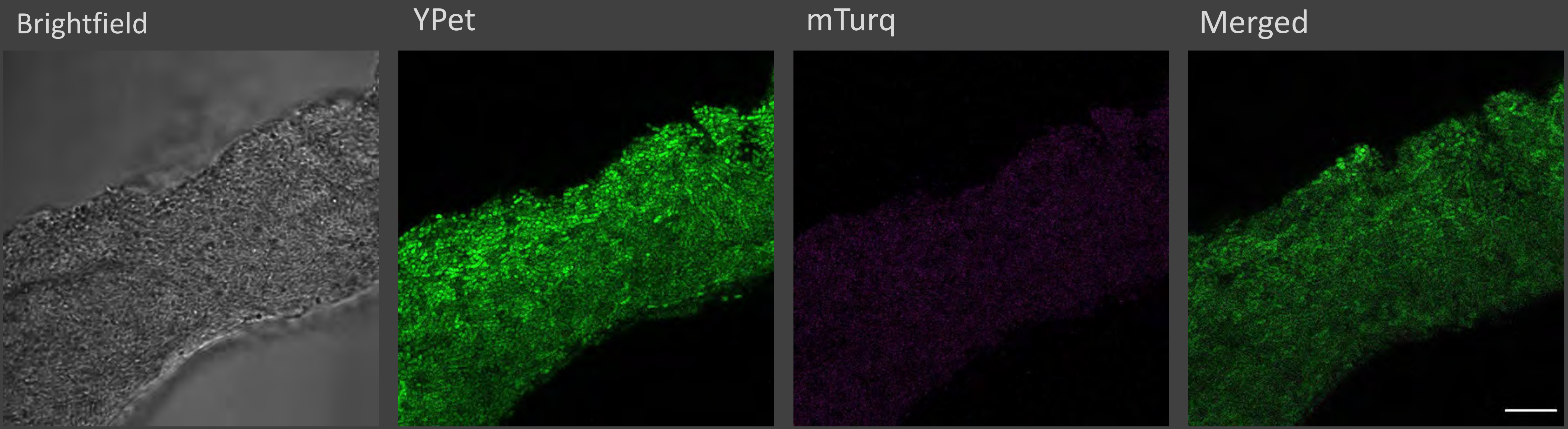

2020-01-09

SY365 P_{sdpA}-Ypet (*cam*); P_{pksC}-mTurq (*erm*) interior

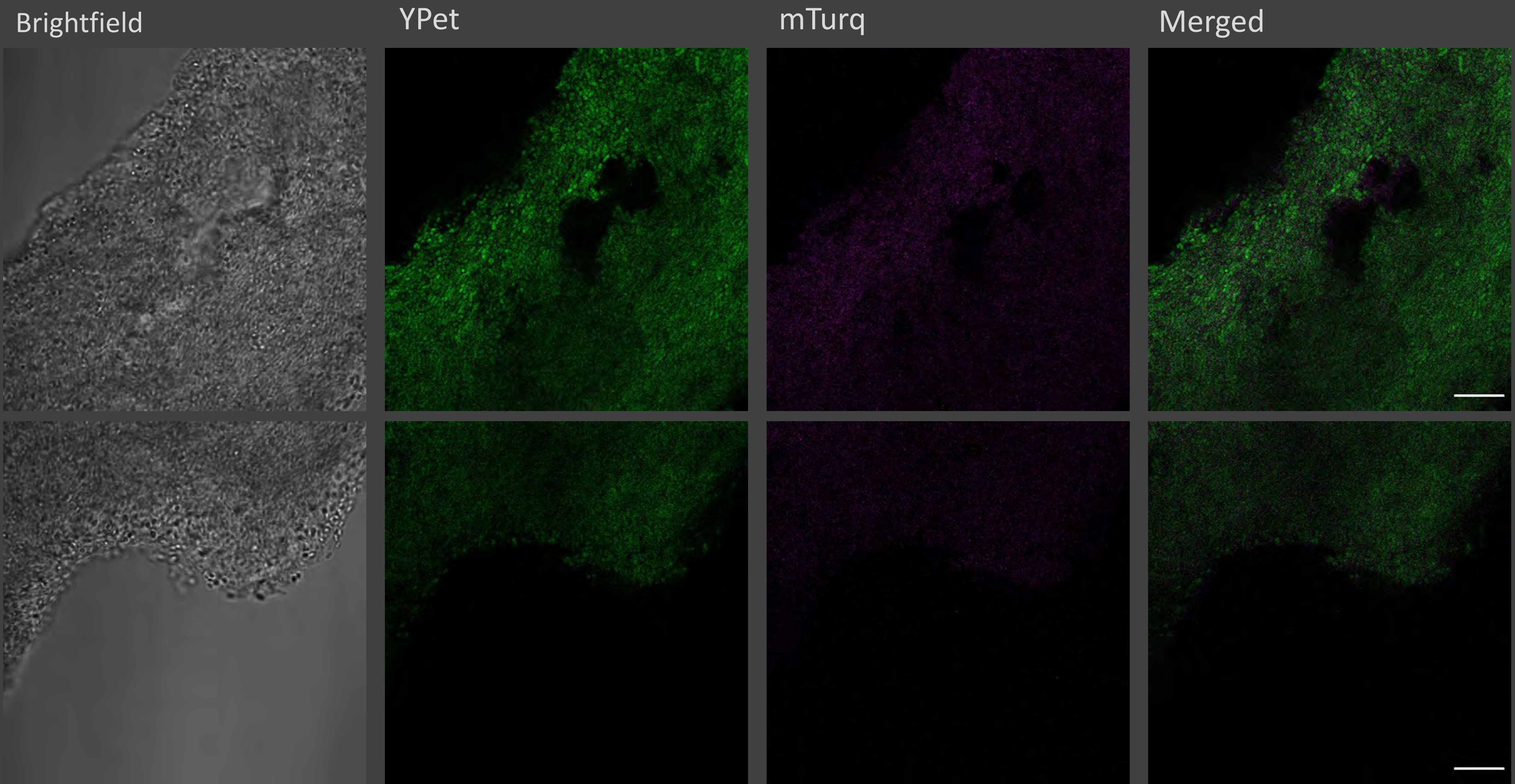

SY375 P_{sdpA}-Ypet (*cam*); P_{skfA}-mTurq (*erm*) periphery

2020-01-18

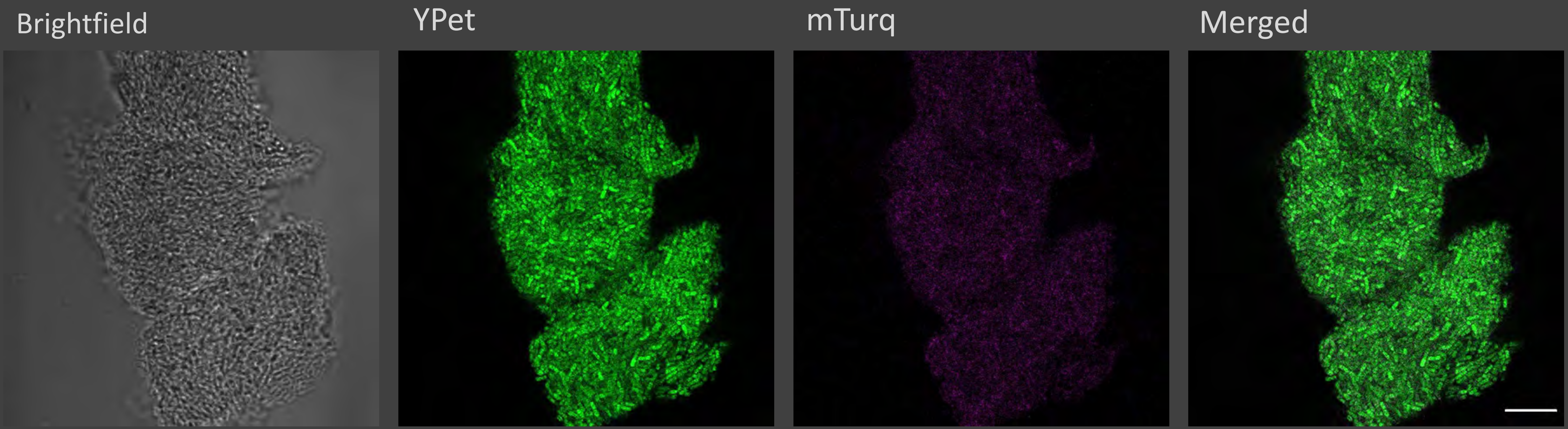

2020-01-18

SY375 P_{sdpA}-Ypet (*cam*); P_{skfA}-mTurq (*erm*) middle

Brightfield | YPet | mTurq | Merged

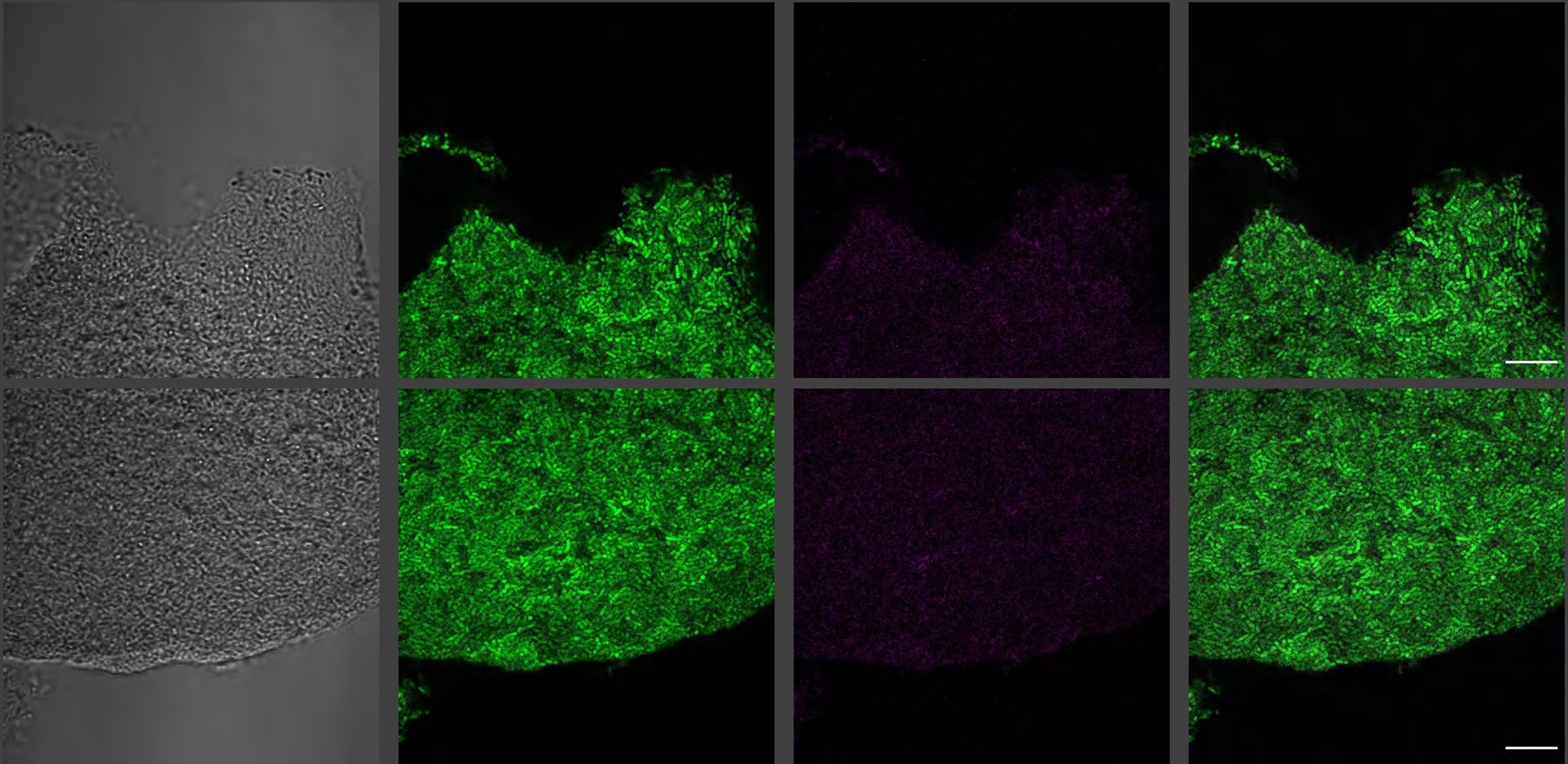

2020-01-18

SY375 P_{sdpA}-Ypet (*cam*); P_{skfA}-mTurq (*erm*) interior

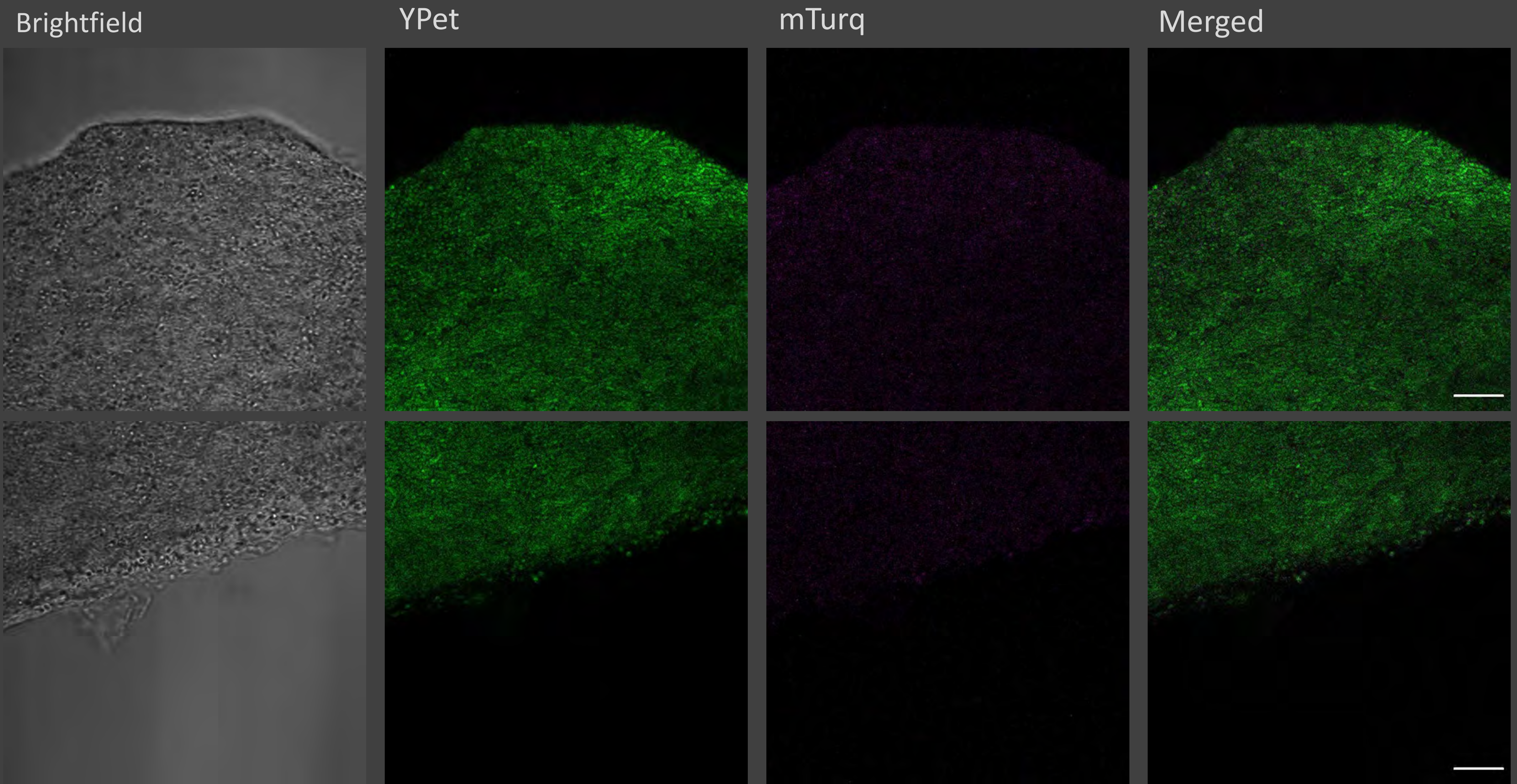

2020-01-18

SY403 P_{pksC}-Ypet (*cam*); P_{aprE}-mTurq (*erm*) periphery

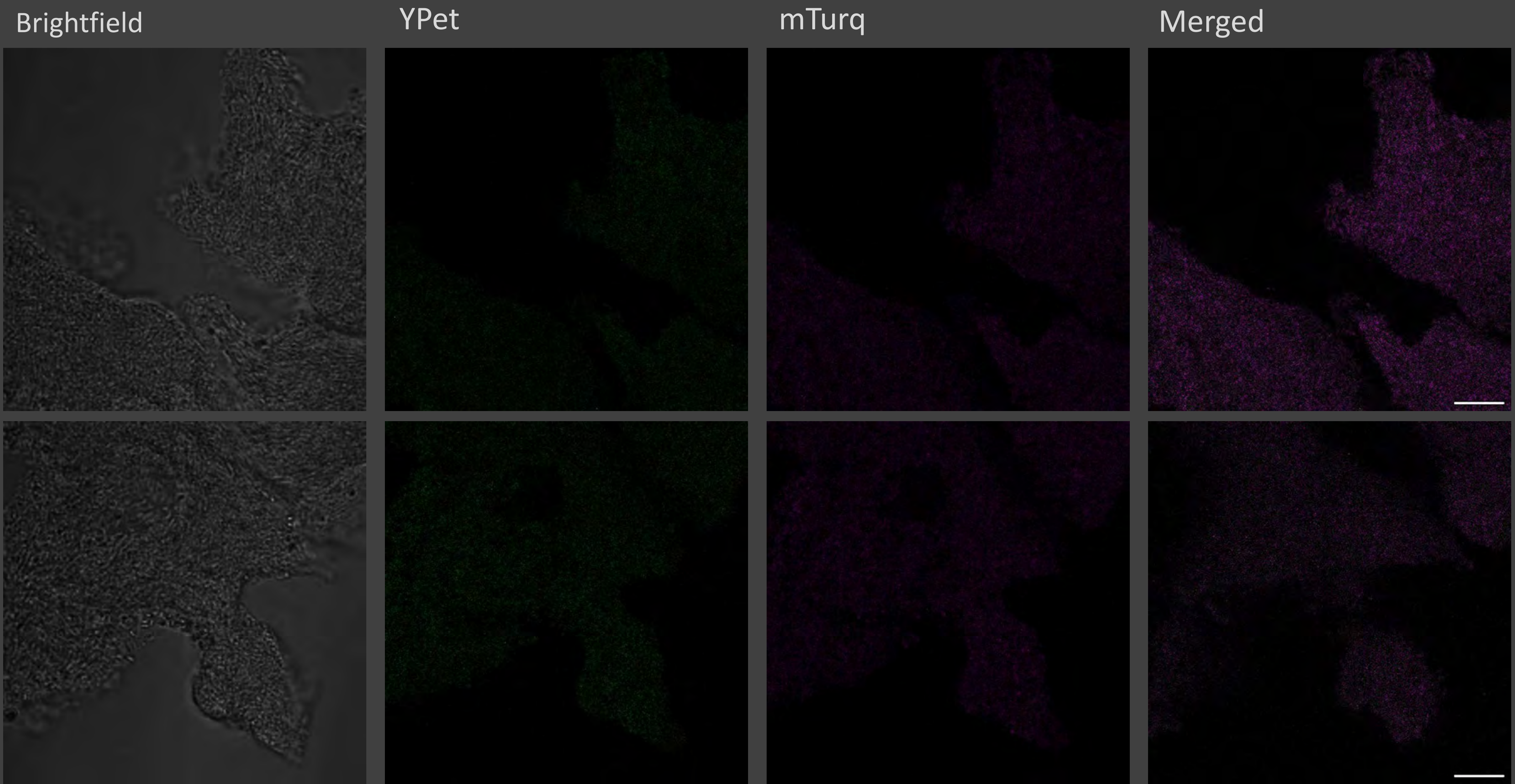

SY403 $P_{\textit{pksC}}$-Ypet (*cam*); $P_{\textit{aprE}}$-mTurq (*erm*) middle

Brightfield

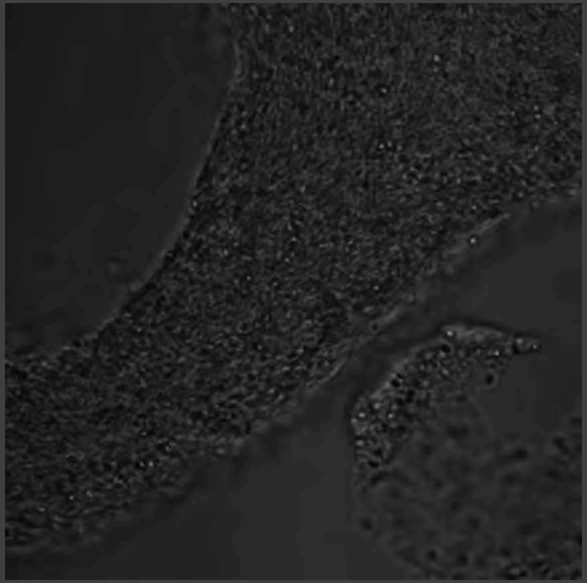

YPet

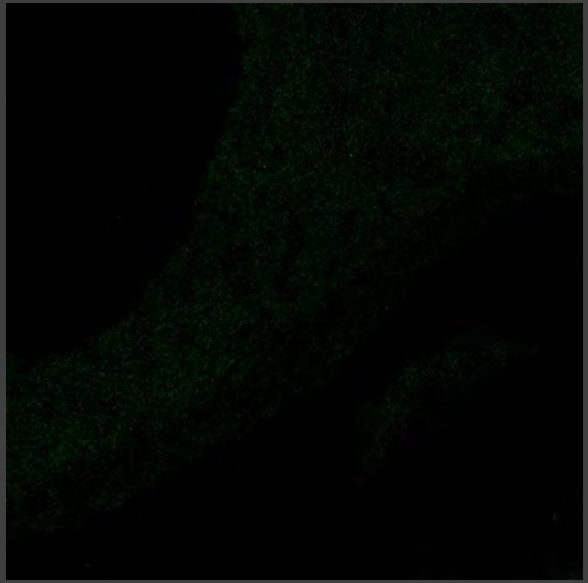

mTurq

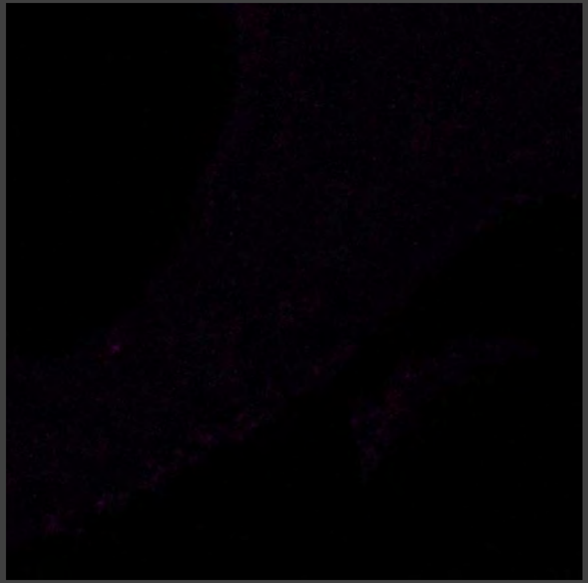

Merged

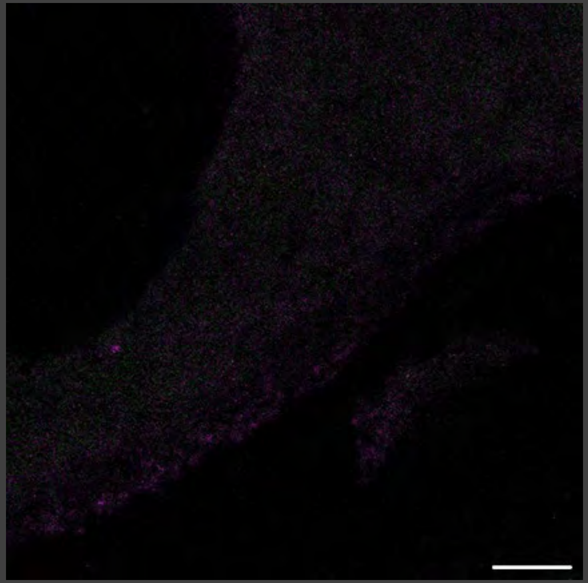

2020-01-18

SY403 P_{pksC}-Ypet (*cam*); P_{aprE}-mTurq (*erm*) interior

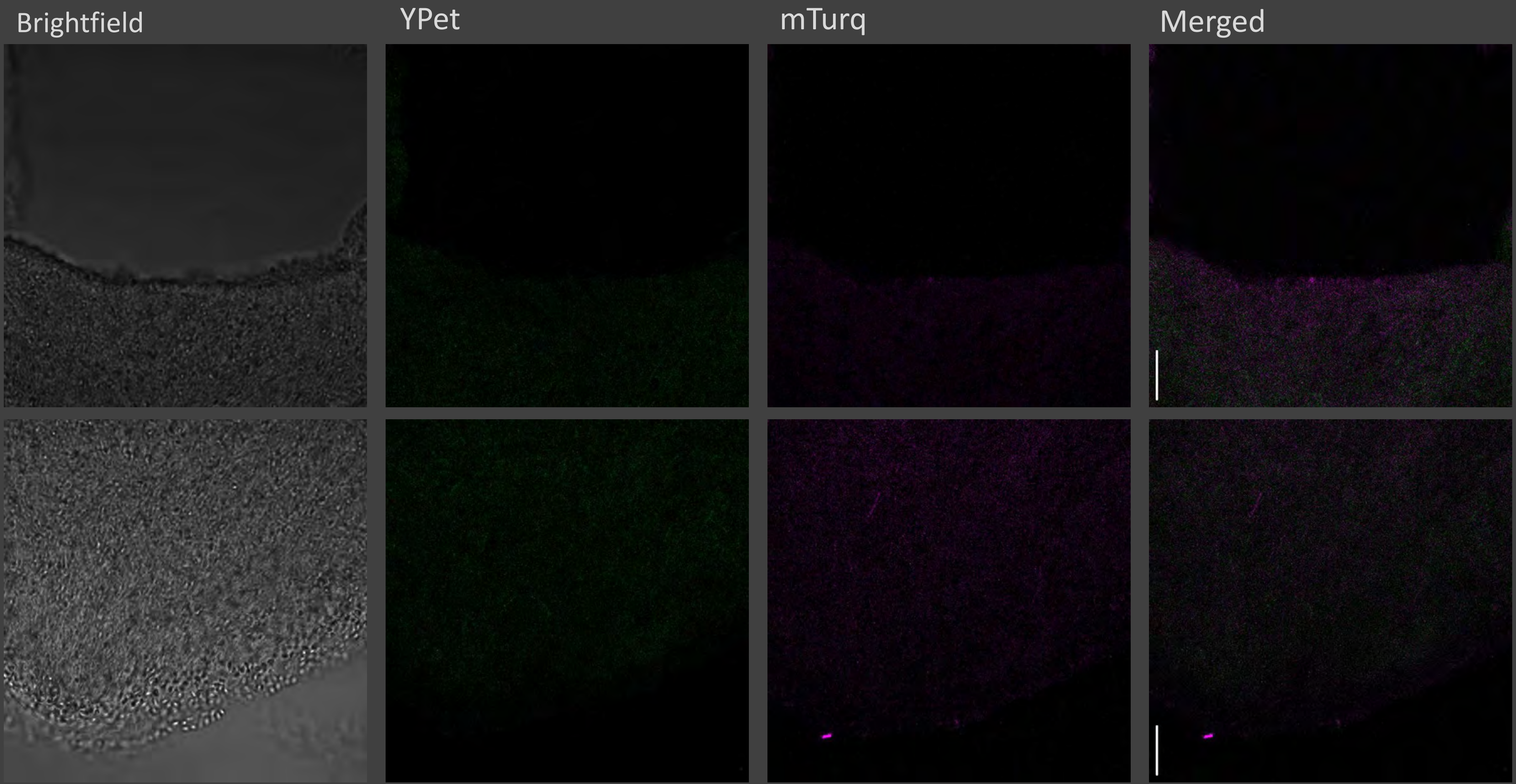

Supplement: FIG S5 — Microscopy images of biofilm thin-sections were taken at two or three fields of view across the biofilm for each strain (see Table S1 for complete list of micrographs collected from which strains) at the approximate positions noted in the biofilm schematic in Fig. S6. Images shown here are single Z-sections of confocal microscopy images that have had their levels linearly adjusted using identical settings (across all panels) in Photoshop to better enable the visualization of the bright pixels in these images. Raw microscopy files can be downloaded as described in the manuscript Data Availability section. [file msystems.00891-22-s0005.pdf]
